# Extreme Hypoxia Causing Brady-Arrythmias During Apnea in Elite Breath-Hold Divers

**DOI:** 10.3389/fphys.2021.712573

**Published:** 2021-12-03

**Authors:** Thomas Kjeld, Anders Brenøe Isbrand, Katrine Linnet, Bo Zerahn, Jens Højberg, Egon Godthaab Hansen, Lars Christian Gormsen, Jacob Bejder, Thomas Krag, John Vissing, Hans Erik Bøtker, Henrik Christian Arendrup

**Affiliations:** ^1^Department of Anesthesiology, Herlev Hospital, University of Copenhagen, Copenhagen, Denmark; ^2^Department of Clinical Physiology and Nuclear Medicine, Herlev Hospital, University of Copenhagen, Copenhagen, Denmark; ^3^Department of Cardiothoracic Anesthesiology, Rigshospitalet, University of Copenhagen, Copenhagen, Denmark; ^4^Department of Clinical Physiology and Nuclear Medicine, Skejby Hospital, Aarhus University, Aarhus, Denmark; ^5^Department of Nutrition, Exercise and Sports (NEXS), University of Copenhagen, Copenhagen, Denmark; ^6^Department of Neurology, Rigshospitalet, University of Copenhagen, Copenhagen, Denmark; ^7^Department of Cardiology, Aarhus University Hospital, Aarhus, Denmark; ^8^Department of Clinical Medicine, Faculty of Medicine, University of Copenhagen, Copenhagen, Denmark

**Keywords:** junctional rhythm, brady-arrythmia, free-diving, invasive blood pressure, hypoxia induced factor-1 (HIF-1), atrioventricular block, apnea and face immersion, bradycardia

## Abstract

**Introduction:** The cardiac electrical conduction system is very sensitive to hypoglycemia and hypoxia, and the consequence may be brady-arrythmias. Weddell seals endure brady-arrythmias during their dives when desaturating to 3.2 kPa and elite breath-hold-divers (BHD), who share metabolic and cardiovascular adaptions including bradycardia with diving mammals, endure similar desaturation during maximum apnea. We hypothesized that hypoxia causes brady-arrythmias during maximum apnea in elite BHD. Hence, this study aimed to define the arterial blood glucose (Glu), peripheral saturation (SAT), heart rhythm (HR), and mean arterial blood pressure (MAP) of elite BHD during maximum apneas.

**Methods:** HR was monitored with Direct-Current-Pads/ECG-lead-II and MAP and Glu from a radial arterial-catheter in nine BHD performing an immersed and head-down maximal static pool apnea after three warm-up apneas. SAT was monitored with a sensor on the neck of the subjects. On a separate day, a 12-lead-ECG-monitored maximum static apnea was repeated dry (*n* = 6).

**Results:** During pool apnea of maximum duration (385 ± 70 s), SAT decreased from 99.6 ± 0.5 to 58.5 ± 5.5% (∼PaO_2_ 4.8 ± 1.5 kPa, *P* < 0.001), while Glu increased from 5.8 ± 0.2 to 6.2 ± 0.2 mmol/l (*P* = 0.009). MAP increased from 103 ± 4 to 155 ± 6 mm Hg (*P* < 0.005). HR decreased to 46 ± 10 from 86 ± 14 beats/minute (*P* < 0.001). HR and MAP were unchanged after 3–4 min of apnea. During dry apnea (378 ± 31 s), HR decreased from 55 ± 4 to 40 ± 3 beats/minute (*P* = 0.031). Atrioventricular dissociation and junctional rhythm were observed both during pool and dry apneas.

**Conclusion:** Our findings contrast with previous studies concluding that Glu decreases during apnea diving. We conclude during maximum apnea in elite BHD that (1) the diving reflex is maximized after 3–4 min, (2) increasing Glu may indicate lactate metabolism in accordance with our previous results, and (3) extreme hypoxia rather than hypoglycemia causes brady-arrythmias in elite BHD similar to diving mammals.

## Introduction

Bradycardia is a well-known consequence of cardiac hypoxia (James et al., 1966; Guilleminault et al., 1983; Kato et al., 1988), but bradycardia is also an underlying oxygen-conserving mechanism in the mammalian diving reflex (Godek and Freeman, 2021). In addition, the mammalian diving reflex includes peripheral vasoconstriction, increased blood pressure, and blood centralized to the brain, lungs, and heart (Foster and Sheel, 2005; Kjeld et al., 2009; Lemaître et al., 2013; Supplementary Table 1). The decreasing heart rate during the apnea dives of animals and humans due to the mammalian diving reflex is vagally mediated, and the His bundle conduction system of the heart is very sensitive to hypoxia and hypoglycemia (James et al., 1966; Senges et al., 1980). Hence, hypoxia or hypoglycemia may inhibit normal conduction. Accordingly, during the dives of Weddell seals, where their partial pressure of oxygen (PaO_2_) decreases to 3.2 kPa, brady-arrythmias including 2^nd^ degree atrioventricular blockade and junctional rhythm can be observed (Qvist et al., 1986; Davis and Kanatous, 1999; Williams et al., 2015), yet the animals stay conscious. In contrast, blackouts or even death may be the consequence of maximum apneas in elite breath-hold divers (BHD), despite a pronounced diving reflex (Foster and Sheel, 2005; Kjeld et al., 2009; Buzzacott and Denoble, 2018) and also metabolic adaptions to hypoxia and hypercapnia similar to diving mammals (Kjeld et al., 2009, 2018). During maximum apneas of elite BHD, we have previously demonstrated that PET-CT-assessed myocardial blood flow increases markedly (by a factor of 2), whereas both PaO_2_ decrease (to 4.3 kPa) and concomitantly circulating lactate levels decrease, indicating lactate metabolism (Kjeld et al., 2021). Lactate metabolism during maximum exercise and apnea can also be demonstrated in high-altitude miners and Sherpas (Ge et al., 1994; Moraga et al., 2019), dwelling at 3,440 to 4,243 m altitudes and having SaO_2_ between 88 and 93% (Jansen et al., 2007). However, in contrast to their lowland relatives and diving seals, the Sherpas have no brady-arrythmias during apnea and hypoxia (Busch et al., 2018). Sherpas are constantly exposed to hypoxia, while BHD and seals are exposed to intermittent hypoxia, and both kinds of exposure may lead to increases in the levels of hypoxia-inducible-factor-1-alpha (HIF-1-alpha), a key transcription factor, that regulates cellular adaptions to hypoxia (Li et al., 2009). Skeletal muscle HIF-1-alpha has been found to be cardioprotective in animal models of intermittent hypoxia (Cai et al., 2003). Seal hearts have low levels of HIF-1-alpha whereas the concentration in their skeletal muscles is high (Johnson et al., 2004, 2005). HIF-1 mediates adaptions to hypoxia by downregulating mitochondrial oxygen consumption (Papandreou et al., 2006), and similarly BHD and diving mammals are characterized by low mitochondrial oxygen consumption in their skeletal muscles (Kjeld et al., 2018). HIF-1-alpha do also interrelate with pancreatic beta-cell function: increased levels of HIF-1-alpha improve insulin secretion (Cheng et al., 2010), and according to Dangmann, insulin is theoretically a key factor under hypoxic conditions; it was also concluded that insulin secretion stops during hypoxia (Dangmann, 2015). Sponsiello et al. confirmed this theory in BHD and found a decrease in blood glucose of ∼11.5% with a concomitant increase in insulin of ∼ 55% after 5 dives to 20 m (Sponsiello et al., 2018). Theoretically, the consequence of further repeated dives, for example, in underwater spearfishing, would be fatal hypoglycemia.

In our previous study of elite BHD, we demonstrated both decreased levels of circulating lactate and significantly increased myocardial blood flow during maximum apnea, the latter by a factor of 2 compared to resting conditions (Danad et al., 2014). Hence, maximum apnea in elite BHD can be compared to maximum aerobic exercise. Miller et al. (2002) demonstrated during aerobic exercise and lactate infusion that glucose was stable due to decreased metabolism. Therefore, assuming that the decreasing lactate during maximum apnea is indicative of lactate metabolization (Kjeld et al., 2021), and if glucose is stable in elite BHD during maximum apnea, then hypoxia may be the key factor causing bradycardia, as the His bundle conduction system of the heart is very sensitive to both hypoxia and hypoglycemia (James et al., 1966; Senges et al., 1980).

The aim of the present study was to determine, in elite BHD during maximum apnea, (1) peripheral saturation, (2) blood glucose, (3) cardiac-monitored brady-arrythmias, and (4) invasive measured blood pressure. (5) The resting levels of skeletal muscle HIF-1-alpha in BHD were compared to matched controls.

## Materials and Methods

Seventeen healthy/non-medicated male non-smoking subjects participated in the study as approved by the Regional Ethics Committee of Copenhagen (H-1-2013-060). All clinical investigations have been conducted according to the principles expressed in the Declaration of Helsinki. Informed consent, written and oral, have been obtained from the participants. Nine subjects were divers (age 43 ± 3 years), and eight judo athletes matched for morphometric variables (age, height, weight, body mass) and whole-body aerobic capacity (VO_2_max, Table 1) were chosen for comparison.

**TABLE 1:** Subject characteristics.

	Divers	Controls
No. subjects	9 males	8 males
Age (years)	39 ± 10	36 ± 11
Height (cm)	184 ± 6	183 ± 4
Weight (kg)	80.4 ± 6.0	79.9 ± 7.3
Body mass index (kg/m^2^)	23.6 ± 1.9	23.6 ± 1.2
Fat mass%	17.7 ± 5.5	15.4 ± 6.2
Fat (kg)	14.2 ± 4.9	12.7 ± 6.0
Maximal oxygen uptake (mL O2/min/kg)	48.6 ± 7.1	47.5 ± 7.1
Hemoglobin (%)	8.9 ± 0.7	8.9 ± 0.8
Static personal best (seconds)	395 ± 48	N/A
Dynamic pool personal best (meters)	171 ± 38	N/A
Dynamic pool no fins personal best (meters)	143 ± 38	N/A

*Basic morphometric data. Values are mean ± SD.*

All free divers ranked among the national top 10, three of the participating free divers ranked among the World top 10, and one was a 2016 outdoor free-diving World champion, while one reached third place at the same Championship (no limit depth competition), and one was a World record holder.

### VO_2_ Max and Dual-Energy X-Ray Absorptiometry Scan

Subjects completed a standardized warm-up followed by an incremental cycling test starting at a workload of 150 W and increasing 25 W every minute until voluntary exhaustion. The highest recorded 30 s average oxygen uptake (VO_2_) during the test was defined as VO_2_max. For recognition of true VO_2_max, three of five criteria had to be met: individual perception of exhaustion, respiratory exchange ratio >1.15, plateau of VO_2_ curve, heart rate approaching age-predicted maximum, and inability to maintain a pedaling frequency above 70 rpm (Table 1).

To describe subjects, an assessment of body composition was determined with a dual-energy X-ray absorptiometry scan (Lunar iDXA; Lunar, Madison, WI, United States) (Table 1).

### Muscle Biopsies

Subjects were instructed to refrain from exercise and apnea before muscle biopsies were taken. Muscle biopsies were obtained in local anesthesia with lidocaine (5%) from the lateral vastus of the femoral muscle a.m. Bergstroem (Gusba et al., 2008). The biopsies were snap-frozen in liquid N_2_ and stored at −80°C. Western blotting was performed as previously described in detail (Krag et al., 2016). Briefly, biopsies were cut on a microtome, the sections were homogenized in sample buffer, and proteins were separated on SDS-page gels and blotted onto a membrane using a Trans-turbo blotter (Bio-Rad, Hercules, CA, United States). The membrane was incubated with antibodies against HIF1-alpha (AF1935, R&D Systems, Minneapolis, MN, United States) and alpha-tubulin (clone 12G10, Developmental Studies Hybridoma Bank, University of Iowa, Iowa City, IA, United States), followed by rabbit anti-goat and rabbit anti-mouse horseradish peroxidase-conjugated antibodies. The membrane was developed using Clarity and visualized on a ChemiDoc MP (Bio-Rad). The muscle specimen of one control participant was not sufficient for analyses.

### Pool Apneas

The nine elite breath-hold divers were instructed to refrain from caffeine intake and to fast for at least 6 h before the pool apneas. Any strenuous physical activity was discouraged for at least 1 day before the experiment.

A 1.1 mm, 20-gauge catheter was inserted in the radial artery of the non-dominant arm connected to a transducer for continuous flow of saline (3 ml/h; Baxter, Uden, Netherlands) for blood pressure monitoring and for collection of blood glucose. Blood glucose analyses were performed immediately after sampling, using an automated self-calibrating blood gas machine (ABL 725, Radiometer, Copenhagen, Denmark) and evaluated for blood gases as well as described previously (Kjeld et al., 2021).

The BHD performed head immersed maximal static apnea after glossopharyngeal insufflation (GPI) (Seccombe et al., 2006) in a 28^°^C, 0.8-m-deep indoor pool after a warm-up of three consecutive apneas to maximize the diving response (Kjeld et al., 2009). They were instructed to make a sign with their index finger, just before terminating apnea, so blood glucose sampling could be made just before breathing.

Arterial blood pressures and heart rates were recorded concomitantly using a Lifepack ^®^ 20 monitor. To diminish artifacts from water, heart rhythm (HR) was monitored with electrodes for direct current therapy, and hence only one lead was recording (∼ lead II): electrodes were placed at the sternum and under the left arm in an antero-lateral position. All subjects had normal ECG at rest. Peripheral saturation was monitored with a Covidien ^®^ saturation sensor placed at the neck of the subjects similarly to avoid artifacts from water, but also to ensure valid peripheral saturation, during max apnea and concomitant peripheral vasoconstriction.

### Dry Apneas

On a separate day, six of the BHD with the longest self-reported apneas completed a dry apnea after GPI during 12-lead ECG monitoring and after a warm-up of three consecutive apneas to maximize the diving response (Kjeld et al., 2009).

### Statistical Analysis

Variables are presented as mean ± standard error of the mean (SEM). Data were analyzed by Sigma-Plot ^®^ using one-way repeated-measures ANOVA. The Holm-Sidaks method *post hoc* was used to evaluate differences between the collected data during rest, apnea, and recovery. A *P*-value < 0.05 was considered statistically significant.

## Results

**FIGURE 1:**
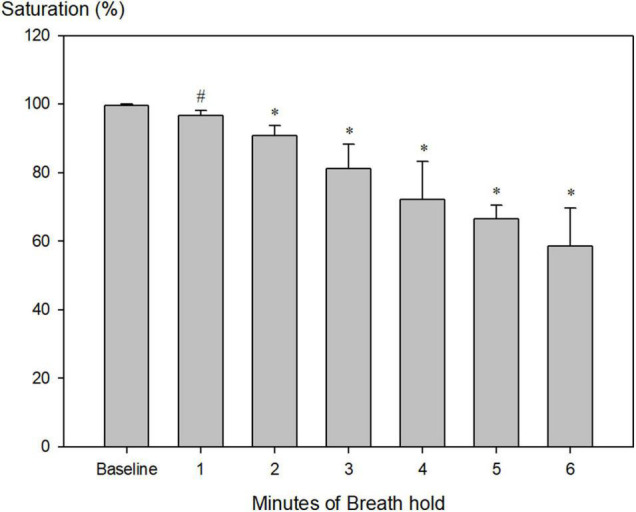
Peripheral measured saturation (sensor placed at neck, *n* = 9) during pool apnea decreased from 99.6 ± 0.5% every minute until termination of breath hold to 58.5 ± 5.5% (#*P* = 0.004, *P < 0.001 compared to baseline).

At the end of maximum pool apnea (314 ± 64 s), peripheral saturation decreased from 99.6 ± 0.5 to 58.5 ± 5.5% (Figure 1; Table 2, *P* < 0.001), while blood glucose increased from 5.8 ± 0.2 at rest to 6.2 ± 0.2 mmol/l just before the end of apnea (Table 2, *P* = 0.009).

**TABLE 2:** Peripheral saturation, blood glucose, heart rate, and mean arterial blood pressure during pool apnea.

	Rest	End BH	After BH
Mean arterial pressure/mmHg	103 ± 11	148 ± 15*	104 ± 31
Heart Rate/beats min^–1^	86 ± 14	46 ± 10*	64 ± 9*
Peripheral Saturation/%	99.6 ± 0.5	58.5 ± 5.5*	N/A
Blood glucose mmol/l	5.8 ± 0.2	6.2 ± 0.2 #	N/A

Values are means ± SD; *P < 0.001 vs. rest. #P = 0.009 vs. rest. BH = breath hold.

**FIGURE 2:**
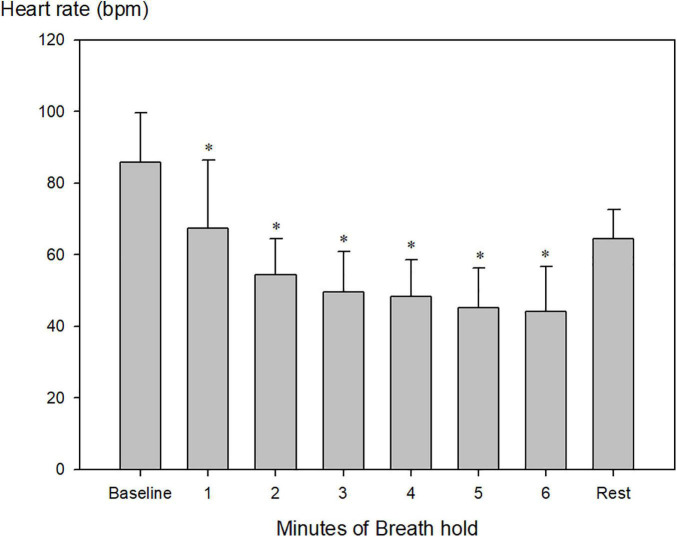
Heart rate during maximum pool apnea. Heart rate decreased from 86 ± 14 beats per minute (bpm) to 46 ± 10 bpm after the first 4 min of apnea compared to baseline and stabilized until termination of breath hold (*P < 0.001 compared to baseline, *n* = 9).

**FIGURE 3:**
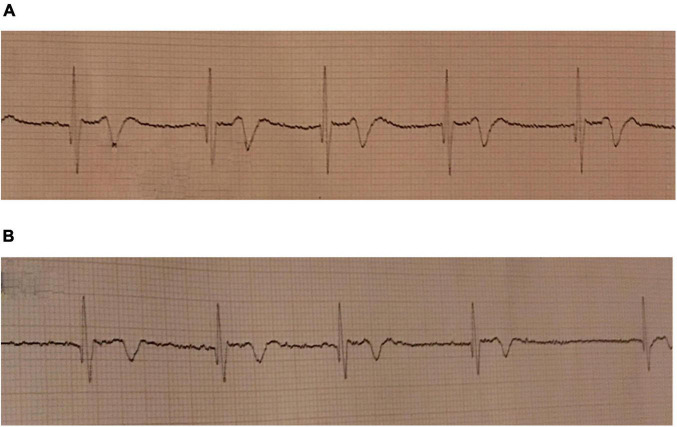
ECG during maximum pool apnea in two subjects. **(A)** Nodal rhythm during maximum dry apnea. Recorded at 25 mm/s. **(B)** Atrioventricular dissociation during maximum pool apnea. Recorded at 25 mm/s.

Heart rate decreased from 86 ± 14 beats per minute (bpm) to 46 ± 10 bpm and remained constant after 4 min (Figure 2; Table 2, *P* < 0.001). Just before the end of apnea, junctional rhythm (*n* = 4), 2^nd^ degree atrioventricular blockade (*n* = 1) and sinus bradycardia (*n* = 3) was observed (Figures 3A,B and Supplementary Datasheet 1).

**FIGURE 4:**
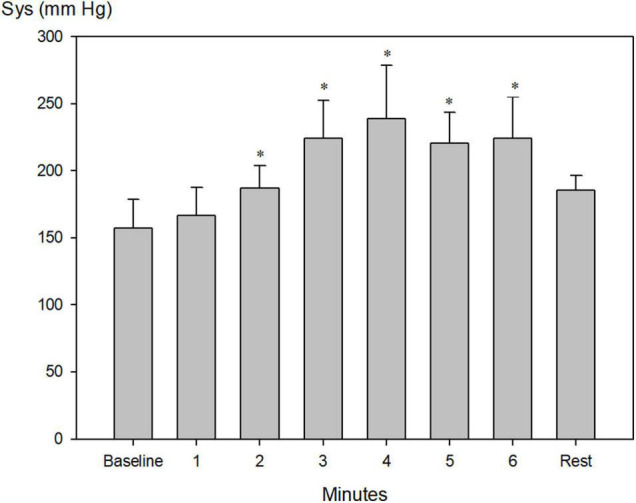
Invasively measured systolic blood pressure (Sys) during pool apnea. Systolic blood pressure increased every minute from 157 ± 7 before apnea (baseline) to a maximum of 239 ± 15 mm Hg after 4 min of apnea (*P < 0.001 compared to baseline, *n* = 9).

**FIGURE 5:**
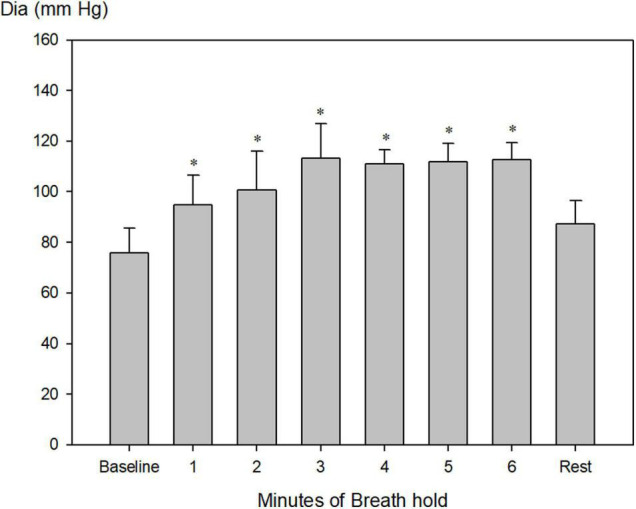
Invasively measured diastolic blood pressure (Dia) during pool apnea. Diastolic blood pressure increased every minute from 76 ± 3 to a maximum of 113 ± 5 mm Hg after 3 min of apnea compared to rest and remained constant hereafter until termination of breath hold (*P < 0.001 compared to baseline, *n* = 9).

Systolic blood pressure increased from 157 ± 7 to a maximum of 239 ± 15 mm Hg after 4 min of apnea (Figure 4, *P* < 0.001), whereas diastolic blood pressure increased from 76 ± 3 mmHg to a maximum of 113 ± 5 mm Hg after 3 min of apnea (Figure 5, *P* < 0.001).

**FIGURE 6:**
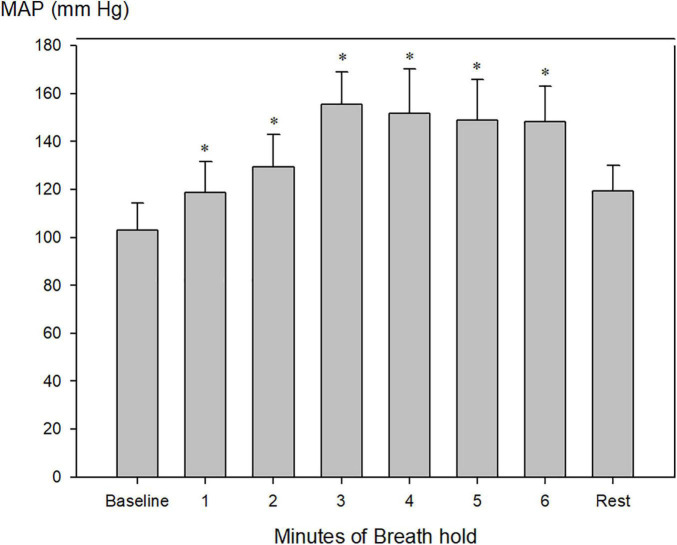
Invasively measured mean arterial blood pressure (MAP) during pool apnea. After 2 min of breath hold mean arterial blood pressure increased every minute compared to rest from 103 ± 4 to a maximum of 155 ± 6 after 3 min of apnea (*P < 0.001 compared to baseline, *n* = 9).

Mean arterial blood pressure increased from 103 ± 4 to a maximum of 155 ± 6 after 3 min of apnea (Figure 6; Table 2, *P* < 0.001).

**FIGURE 7:**
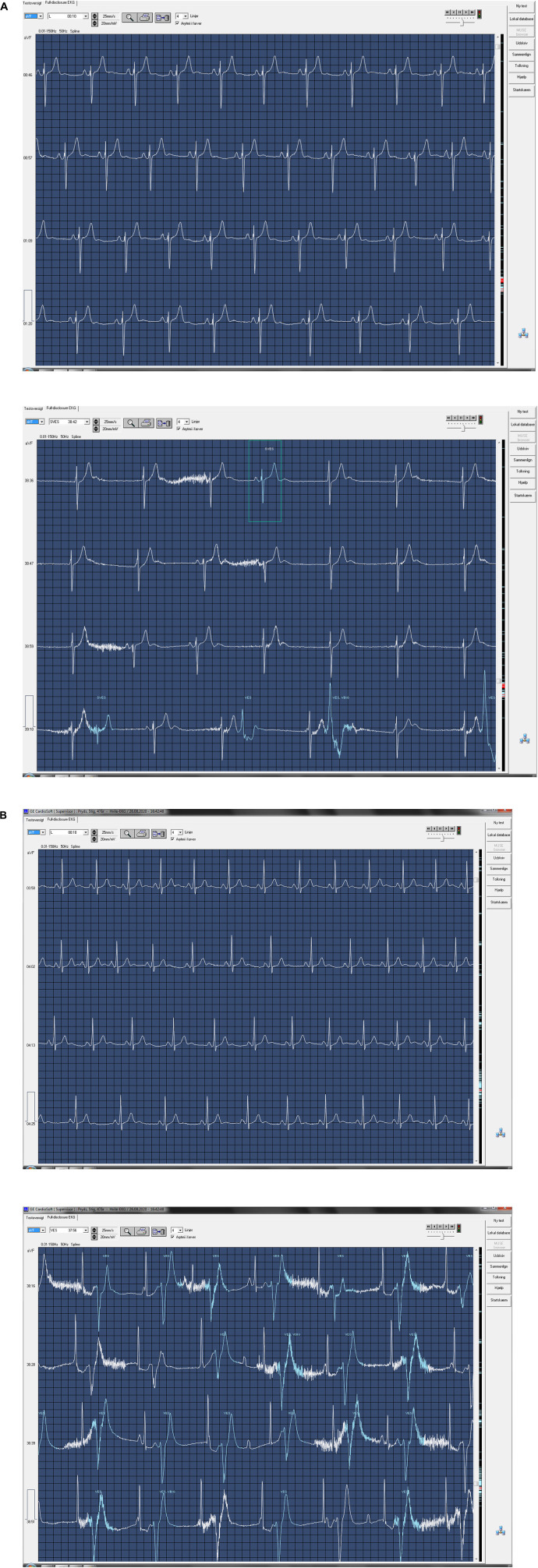
ECG at rest and ECG during maximum dry apnea in two subjects. **(A)** Sinus rhythm at rest (top) and nodal rhythm during maximum dry apnea (bottom). Recorded at 25 mm/s. Continues on next page. **(B)** Sinus rhythm at rest (top) and second-degree atrioventricular dissociation during maximum dry apnea (bottom). Recorded at 25 mm/s.

During dry apnea of maximum duration (378 ± 31 s), heart rate decreased from 55 ± 4 to 40 ± 3 bpm (*P* = 0.031). Sinus bradycardia (*n* = 3), 2^nd^ degree atrioventricular dissociation (*n* = 1), and junctional rhythm (*n* = 2) were observed (Figures 7A,B and Supplementary Datasheet 2). No ST-segment changes were detected.

All BHD remained conscious during both maximum pool and dry static apneas.

**FIGURE 8:**
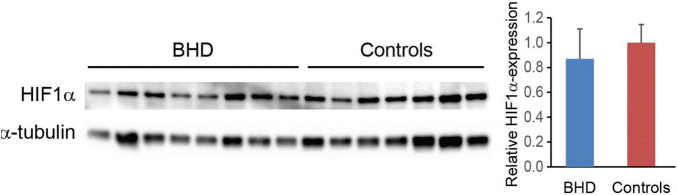
Hypoxia-inducible-factor-1-alpha (HIF1α) expression normalized to α-tubulin shows no difference between breath-hold divers (BHD, *n* = 8) and controls subjects (*n* = 6).

Muscle biopsy analyses revealed no differences in HIF-1-alpha between BHD (relative value 0.87 ± 0.09, *n* = 9) and controls (relative value 1.00 ± 0.06, *n* = 7): *P* = 0.121 (Figure 8).

## Discussion

The main and novel findings of our study are:

After a warm-up of three consecutive apneas and at end of maximum pool apnea (314 ± 64 s) after GPI, the elite BHD (1) tolerated peripheral saturation as low as 58.5 ± 5.5% (∼PaO_2_ 4.8 ± 1.5 kPa), (2) had stable or increasing blood glucose, (3) increased in MAP to a maximum after 3 min of apnea without subsequent changing, (4) decreased in heart rate to a minimum of 46 ± 10 bpm and stabilized after 4 min until termination of apnea, (5) junctional rhythm, 2^nd^ degree atrioventricular blockade, and sinus bradycardia were observed concomitantly by 1-lead ECG during maximum pool apnea, and (6) during maximum dry apnea, both junctional rhythm and 2^nd^ atrioventricular dissociations were confirmed with 12-lead ECG. To our knowledge, these combined and detailed hemodynamic observations, and detailed arrythmias in particular have never been demonstrated earlier in elite BHD during static apneas: Although Ferrigno et al. (1997) and Lindholm et al. (2006) reported similar decreases in heart rate and increases in MAP in pressure chamber apnea in two divers and static apnea in competitive divers, respectively, our findings add further information by blood glucose monitoring and continuous ECG recording and analysis. Lemaître et al. reported of junctional rhythm during constant weight 70-m-deep dives (141 s duration, Lemaître et al., 2013), whereas 2^nd^ degree atrioventricular dissociation has not been reported earlier.

### Hypoxia as a Cause of Brady-Arrythmias

Multiple factors determine the heart rate and cardiac conduction including sympathetic and parasympathetic activity, but also hydrostatic pressure on myocardial cells, baroreceptors, blood gases, cardiovascular hormones, and pulmonary stretch receptors or pressure changes in lung volume may modify heart rate and cardiac contractility in BHD and diving mammals (Williams et al., 2015). We demonstrated that GPI before apnea is not followed by an initial increase in heart rate, as otherwise expected due to pulmonary stretching in BHD (Heusser et al., 2010; Pinsky, 2018).

Cellular hypoxia along the His bundle, the area of the cardiac conduction system known to be very sensitive to hypoxia, may elicit atrioventricular dissociation (James et al., 1966), and in patients with myocardial infarction during sleep apnea with desaturation below PaO_2_ of 85%, sinus arrest and 2^nd^ degree atrioventricular have been demonstrated (Guilleminault et al., 1983; Galatius-Jensen et al., 1994). Sleeping adult seals also experience sleep apnea, but do not increase production of reactive oxygen species (ROS) nor suffer systemic or local oxidative damage in contrast to terrestrial animals with sleep apnea (Vazquez-Medina et al., 2012). Adult seals, compared to pups and juvenile seals, also have an increased relative capacity for mitochondrial respiration (Chicco et al., 2014). Adult diving mammals also have resistance to release of ROS during dives to tolerate ischemia (Vazquez-Medina et al., 2006, 2012). These findings supports that the ability to tolerate hypoxia is an adaption. Similarly, BHD who can endure apnea for more than 4 min have a smaller increase in ischemia-modified albumin (IMA), a marker of the release of ROS and a measure of resistance to hypoxemia, compared to BHD who endure less than 4 min of apnea (Joulia et al., 2015). However, both young and adult seals have been demonstrated to have atrioventricular dissociation during dives (Murdaugh et al., 1961; Williams et al., 2015). In line with this, the hearts of adult seals are also sensitive to localized ischemia (Elsner et al., 1985). Our study of elite BHD during apnea of at least 4 min and a decrease in PaO_2_ of ∼ 4.3 kPa demonstrated 2^nd^ degree atrioventricular block, junctional rhythm, and sinus bradycardia similar to diving (juvenile as well as adult) mammals (Williams et al., 2015), and without any association between apnea duration. The consequence of the brady-arrythmias endured by the subjects in our study would, in the general non-diving population if followed by syncope, be treatment with a pacemaker (Brignole et al., 2013).

### Cardiac Metabolism During Maximum Apnea

In contrast to BHD and diving mammals, resistance toward brady-arrythmias has been demonstrated in high-altitude Sherpas during high-altitude apnea, whereas a comparable group of lowlanders had junctional rhythm, 3^rd^ degree atrioventricular block, and sinus pause (Busch et al., 2018). These Sherpas demonstrate decreasing lactate levels during maximum exercise, indicative of lactate metabolization (Ge et al., 1994). Similarly, we have demonstrated that elite BHD decrease circulating lactate levels during maximum apnea (Kjeld et al., 2021), indicative of a similar cardiac metabolism as found in adult harbor seals (Phoca Vitulina), who possess the highest cardiac lactate dehydrogenase activity, compared to terrestrial animals (Fuson et al., 2003). In the present study, we demonstrate stable blood glucose during maximum apnea in the same population of elite BHD (+1) as described in our previous study (Kjeld et al., 2021). The stable blood glucose in our study during maximum apnea with PaO_2_ of ∼ 4.8 kPa may underline that lactate is metabolized, when compared to the similar findings of Miller et al. (2002) demonstrating lactate metabolization during exercise, following decreasing blood glucose metabolization: the pancreatic insulin producing beta-cells are sensitive to moderate hypoxia, causing decreasing insulin production (Sato et al., 2014), and because pancreatic blood supply (from the splenic artery) has been demonstrated to remain stable during apnea (Bakovíc et al., 2003), hypoxia seems to be the main factor affecting the stalled pancreatic insulin production, and hence increasing or more likely stabilizing blood glucose (may be secondarily to peripheral vasoconstriction) during apnea as in our study. Our results contrast with Sponsiello et al. (2018) but are in accordance with Ghiani et al. (2016), who found stable or increasing blood glucose after 30-m-deep apnea diving. Hence, despite the obvious similarities in Sherpa lactate metabolism compared to both seals and BHD, only seals and BHD have atrioventricular dissociation and junctional rhythm during apnea (Murdaugh et al., 1961; Williams et al., 2015). We therefore suggest that metabolism is not a factor causing brady-arrythmias in BHD and diving mammals during apnea, and that the Sherpa resistance towards brady-arrythmia during apnea relies on an adaption to chronic exposure toward hypoxia, whereas the intermittent exposure toward extreme hypoxia of BHD and diving mammals does not protect against brady-arrythmias.

### Hypoxia-Inducible-Factor-1-Alpha

Intermittent hypoxia can upregulate HIF-1 and in turn iNOS gene expression in cardiomyocytes, and since skeletal muscle HIF-1-alpha has been found to be cardioprotective in animal models of intermittent hypoxia (Cai et al., 2003), we assumed that skeletal muscle HIF-1-alpha would be higher in BHD as compared to controls. Our study revealed no differences in skeletal muscle HIF-1-alpha between BHD and controls, and hence remote cardioprotection from skeletal muscle HIF-1-alpha could not be demonstrated.

### Blood Pressure During Hypoxia and Hypercapnia

Tissue oxygen delivery from Hb depends on 2,3 diphosphoglycerate, pH, and CO_2_ (Mairbaurl and Weber, 2012). Partial arterial CO_2_ (PaCO_2_) increases during apnea (Kjeld et al., 2009), and in our latest study of elite BHD increased to 6.7 kPa, in which we also demonstrated that maximum apnea increases myocardial blood flow markedly, whereas cardiac output decreases (Kjeld et al., 2021). In the present study, the blood pressure during maximum apnea in BHD increased to levels comparable to maximum exercise (Caselli et al., 2016): DBP, SYS, and MAP reached a maximum and remained constant after 3 min, and this may depict maximum vasoconstriction as part of the diving response (Ferrigno et al., 1997; Foster and Sheel, 2005). As PaCO_2_ increases and cardiac output decreases during maximum apnea in BHD (Kjeld et al., 2009, 2021), the consequence could be decreases in MAP. However, MAP remained constant in the present study, and we suggest that despite increases in PaCO_2_, which also may vasodilate peripherally, the vasoconstriction caused by the diving reflex of the elite BHD overrules the potential peripheral vasodilatation (caused by increases in PaCO_2_) and decreases in cardiac output, and the consequence is a constant MAP. Of note, HR decreased to a constant level after 4 min of apnea. Hence, we conclude that in elite BHD during maximum apnea and after GPI, the diving response is maximized after 3–4 min and remains constant during apnea.

### Summary

In summary, we found that elite BHD similar to diving mammals have brady-arrythmias during maximum apnea enduring hypoxia of PaO_2_ of 4.8 kPa, but stable or increasing blood sugar. Our results contrast with previous studies, and the stable blood glucose in this study (1) underlines the possibility of lactate metabolization during maximum apnea in elite BHD as we have sought to determine previously and (2) indicates that hypoxia rather than hypoglycemia is a factor causing brady-arrythmias during maximum apnea in elite BHD. Furthermore, we conclude that the diving reflex is maximized after 3–4 min of apnea, and extreme hypoxia is the key factor causing brady-arrythmias in elite BHD similar to diving mammals.

### Perspectives

A high reliance on cardiac lactate metabolism similar to diving mammals is also part of the high-altitude adaption of the Himalayan plateau Pika (*Ochotona curzoniae*) (Li et al., 2015). The Pika, as compared to mice at sea level, also have higher levels of hypoxia-inducible-factor-1-alpha (HIF-1-alpha). In contrast, seal hearts have low levels of HIF-1-alpha (Johnson et al., 2004, 2005), and since the hearts of seals are also sensitive to localized ischemia (Elsner et al., 1985), it may be the low levels of cardiac HIF-1-alpha that explain why seals have brady-arrythmias during apnea, whereas high-altitude Sherpas, genetically adapted to high levels of HIF-1-alpha do not (Suzuki et al., 2003): HIF-1 has been demonstrated to mediate adaptions to hypoxia by downregulating mitochondrial oxygen consumption (Papandreou et al., 2006). Similarly, BHD and diving mammals have been demonstrated to have low mitochondrial oxygen consumption in their skeletal muscle (Kjeld et al., 2018). We suggest that BHD may have low levels of cardiac HIF-1-alpha similar to adult seals, also explaining the similar brady-arrythmias during apnea and hypoxia.

Future studies may reveal the content of cardiac HIF-1-alpha in BHD. Furthermore, it is speculated that the understanding of these mechanisms may help in developing new treatments of severe clinical conditions caused by extreme hypoxia, e.g., cardiac surgery, organ transplantation, and in the post-resuscitation care setting. Elite sprint athletes, fighter pilots, and mountain climbers may benefit from apnea training.

## Data Availability Statement

Data collected in this study are all saved encrypted at hospital servers.

## Ethics Statement

The studies involving human participants were reviewed and approved by Ethical Committee of Copenhagen, Denmark. The participants provided their oral and written informed consent to participate in this study.

## Author Contributions

TKj conceived the study and was in charge of the overall direction and planning of the presented ideas and developed the hypothesis. EH, BZ, LG, TKr, JV, and HA carried out the experiments. TKj, EH, AI, KL, TKr, BZ, LG, and HA contributed to sample preparation. All authors provided feedback, assisted in the analysis, critically revised the manuscript, approved the final revised version, and contributed to the interpretation and analysis of the results according to their specific specialized areas.

## Conflict of Interest

TKj was voluntary (unpaid) board member of the Danish National Diving Federation. The remaining authors declare that the research was conducted in the absence of any commercial or financial relationships that could be construed as a potential conflict of interest.

## Publisher’s Note

All claims expressed in this article are solely those of the authors and do not necessarily represent those of their affiliated organizations, or those of the publisher, the editors and the reviewers. Any product that may be evaluated in this article, or claim that may be made by its manufacturer, is not guaranteed or endorsed by the publisher.
