# Supplementary figures and images for: Extreme Hypoxia Causing Brady-Arrythmias During Apnea in Elite Breath-Hold Divers (part 1 of 2)
Source: Front Physiol. 2021 Dec 3;12:712573. doi: 10.3389/fphys.2021.712573 (PMC8678416; doi:10.3389/fphys.2021.712573)

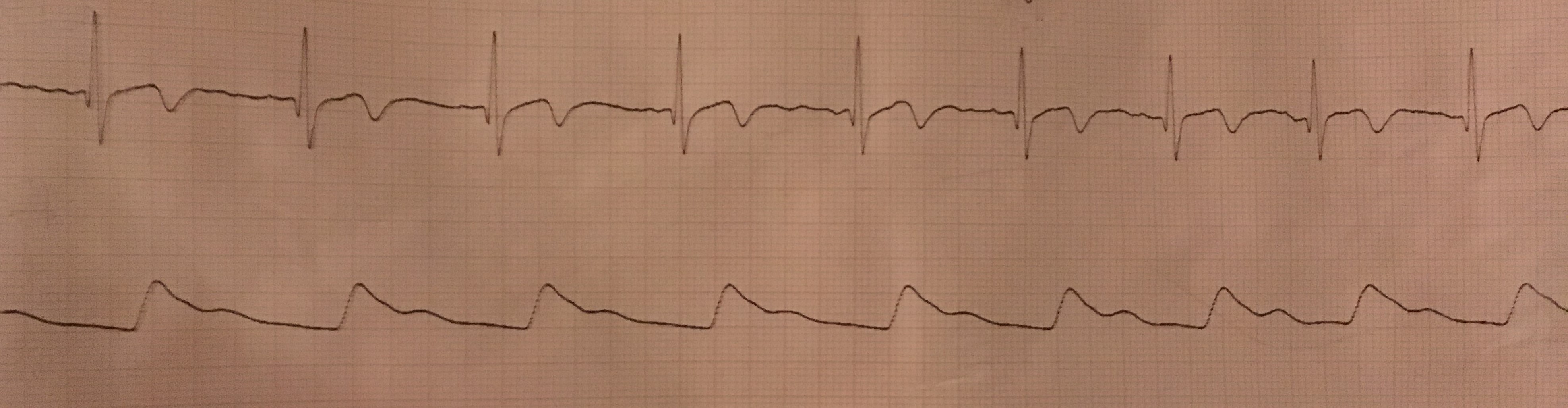

Supplement: Supplementary file 1 [file Data_Sheet_1.zip › ECG recorded at 25 mm pr s during pool apnea/Subject 1 image 1.JPG]

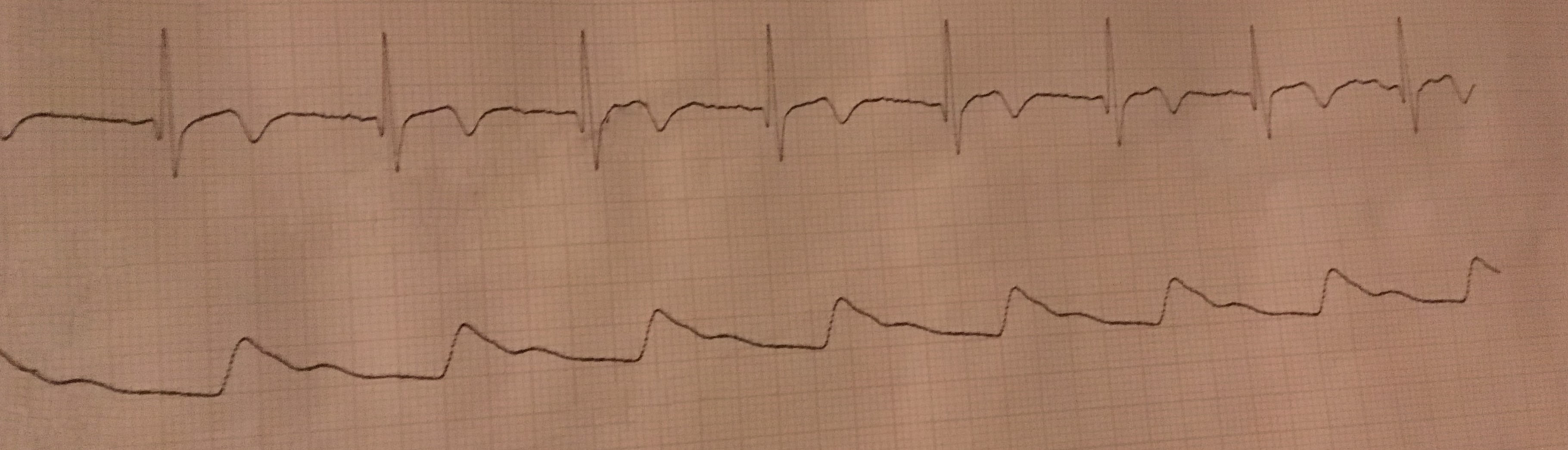

Supplement: Supplementary file 1 [file Data_Sheet_1.zip › ECG recorded at 25 mm pr s during pool apnea/Subject 1 image 2.JPG]

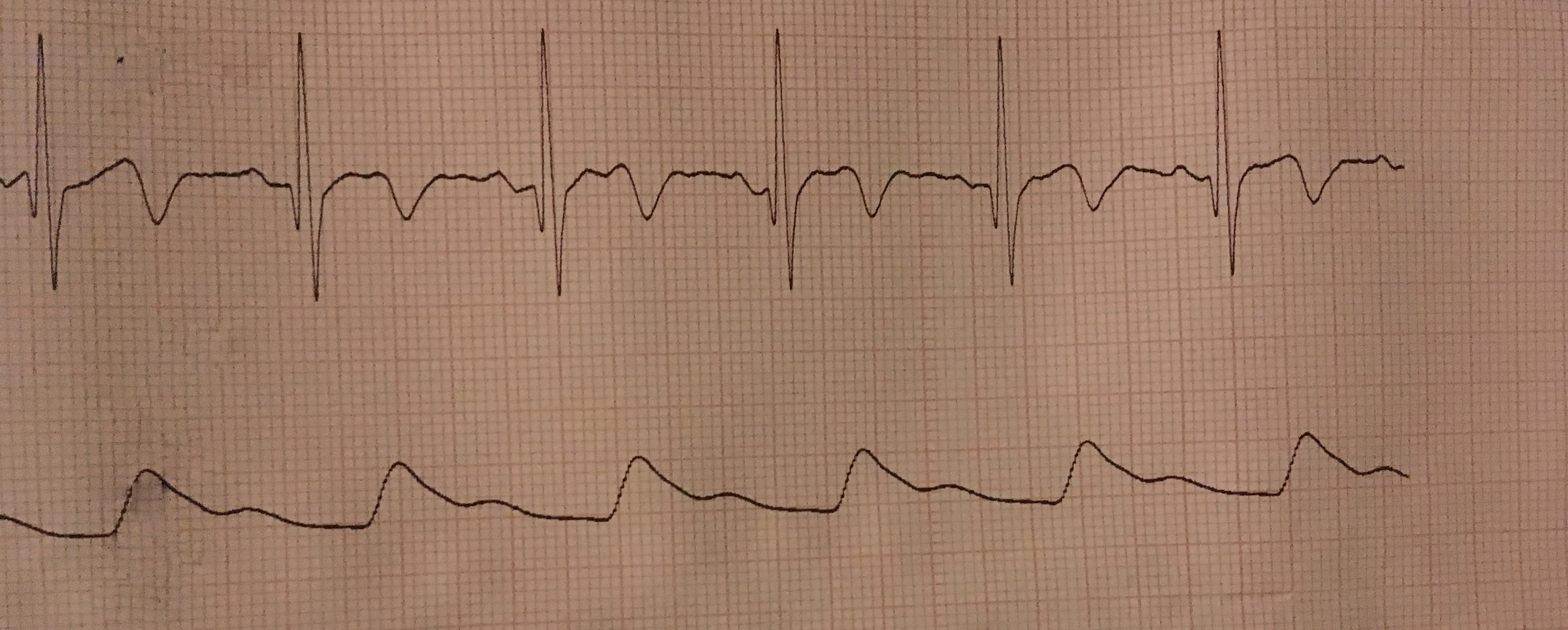

Supplement: Supplementary file 1 [file Data_Sheet_1.zip › ECG recorded at 25 mm pr s during pool apnea/Subject 1 image 3.JPG]

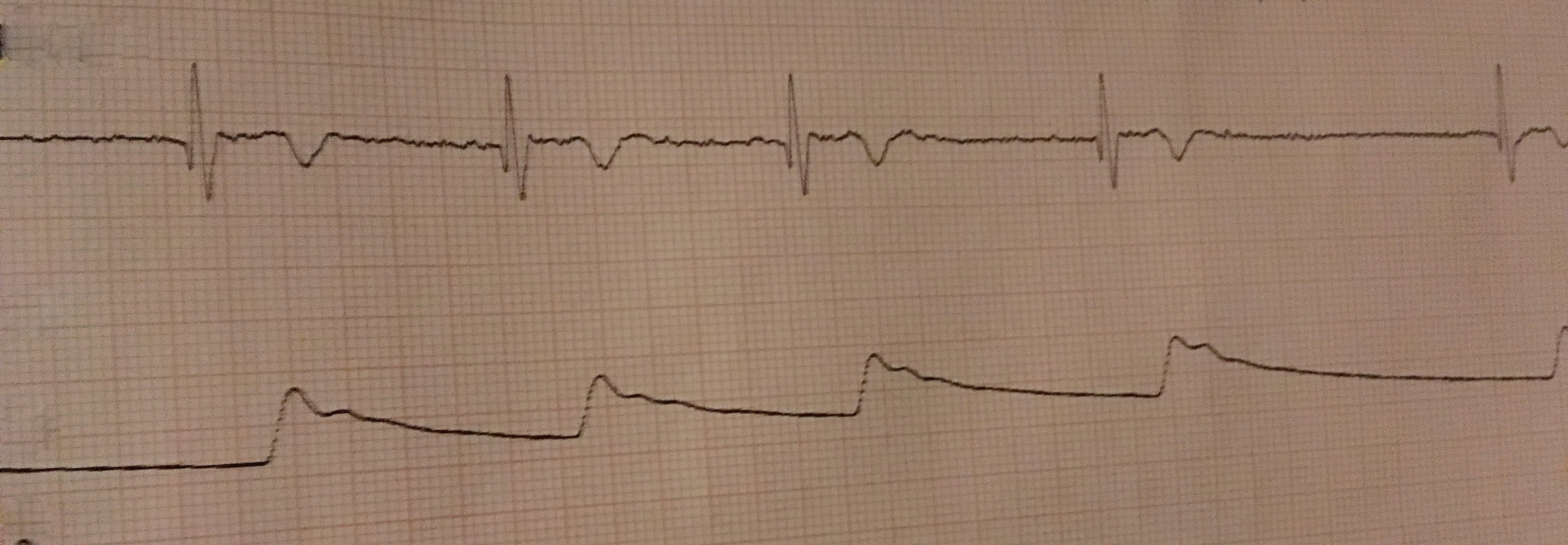

Supplement: Supplementary file 1 [file Data_Sheet_1.zip › ECG recorded at 25 mm pr s during pool apnea/Subject 2 image 1.JPG]

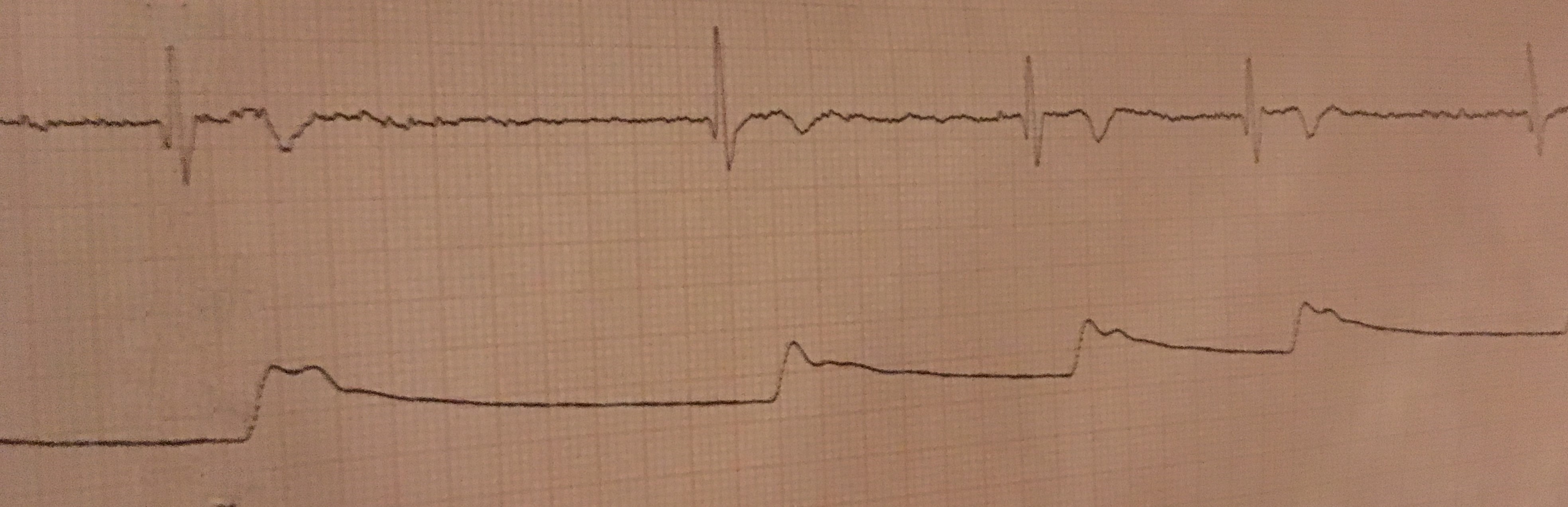

Supplement: Supplementary file 1 [file Data_Sheet_1.zip › ECG recorded at 25 mm pr s during pool apnea/Subject 2 image 2.JPG]

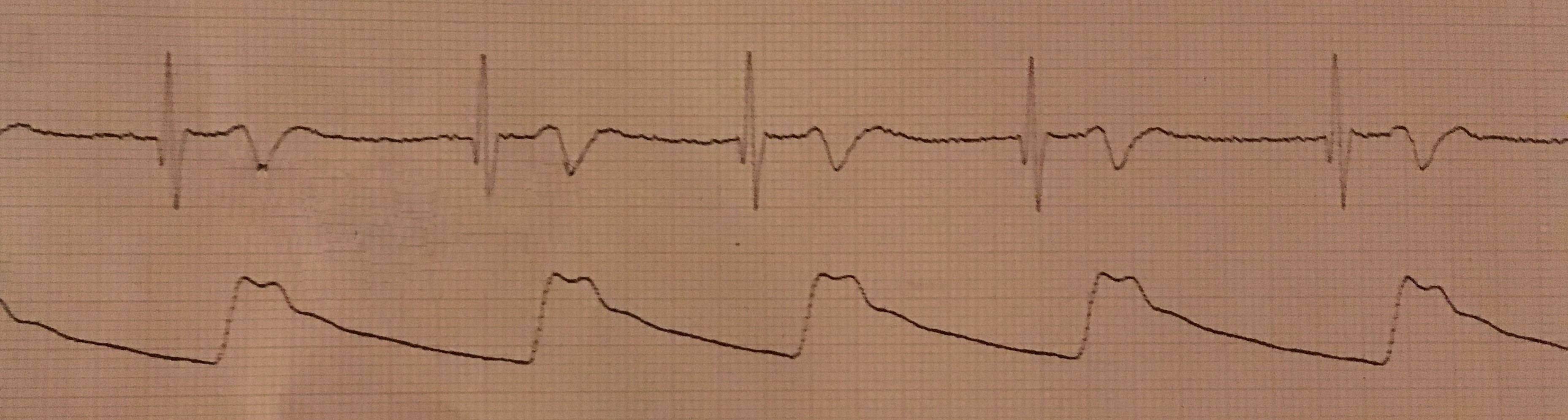

Supplement: Supplementary file 1 [file Data_Sheet_1.zip › ECG recorded at 25 mm pr s during pool apnea/Subject 2 image 3.JPG]

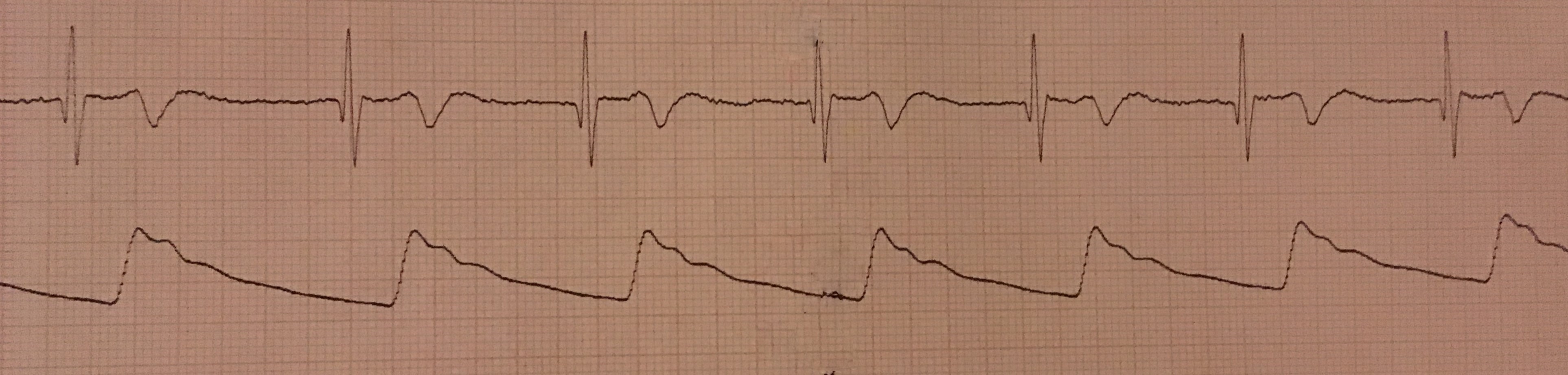

Supplement: Supplementary file 1 [file Data_Sheet_1.zip › ECG recorded at 25 mm pr s during pool apnea/Subject 2 image 4.JPG]

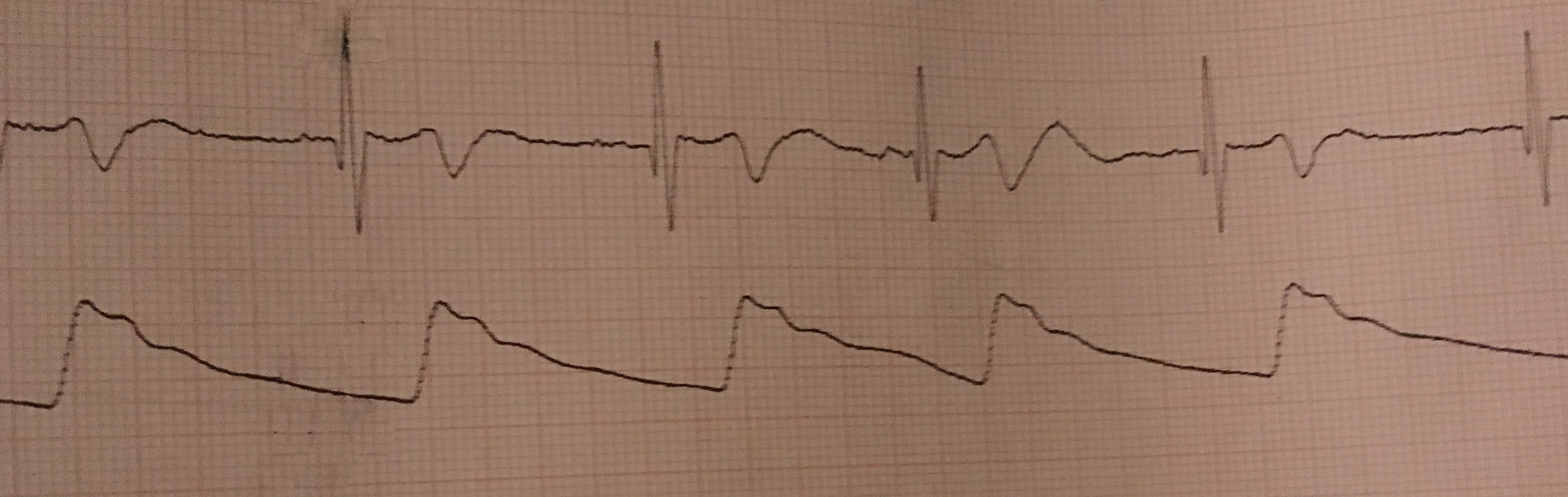

Supplement: Supplementary file 1 [file Data_Sheet_1.zip › ECG recorded at 25 mm pr s during pool apnea/Subject 2 image 5.JPG]

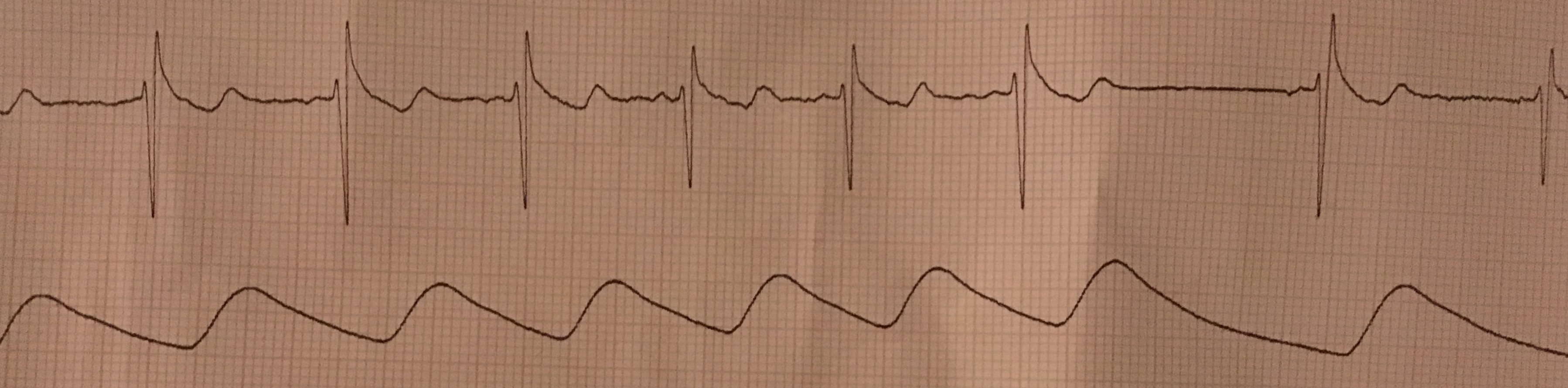

Supplement: Supplementary file 1 [file Data_Sheet_1.zip › ECG recorded at 25 mm pr s during pool apnea/Subject 3 image 1.JPG]

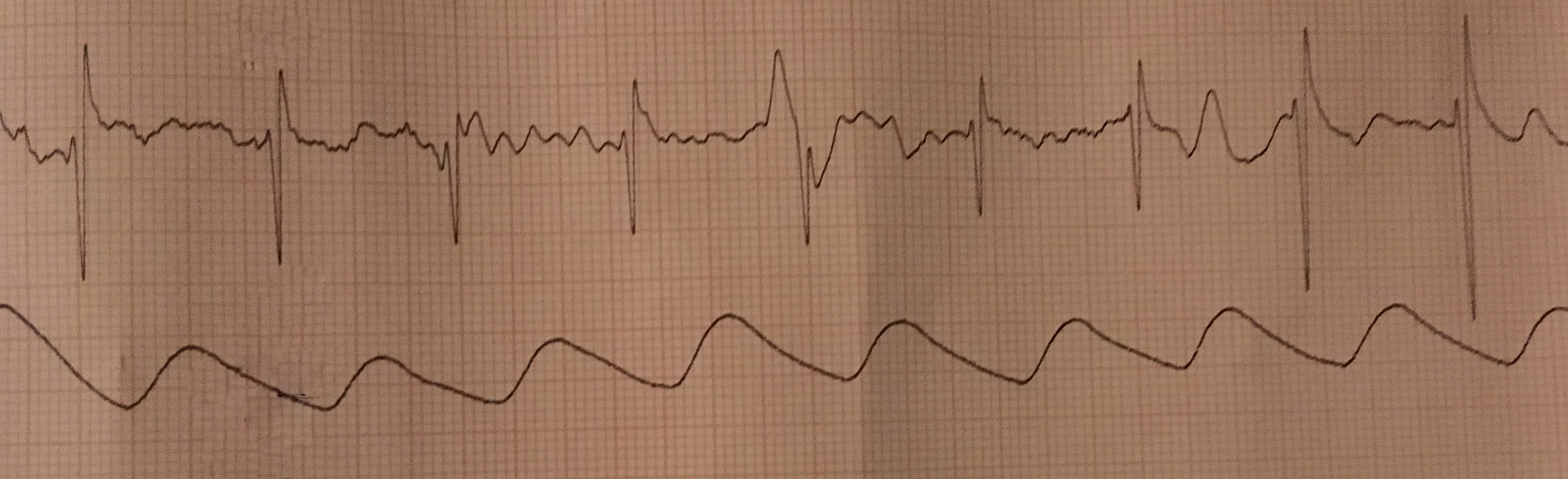

Supplement: Supplementary file 1 [file Data_Sheet_1.zip › ECG recorded at 25 mm pr s during pool apnea/Subject 3 image 2.JPG]

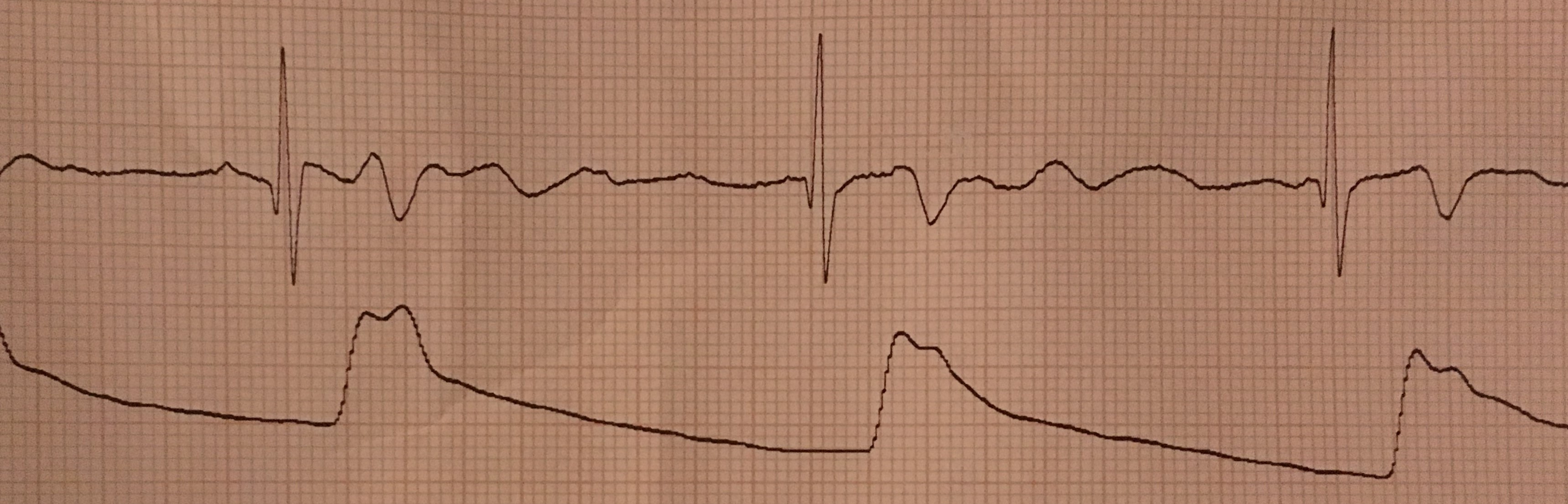

Supplement: Supplementary file 1 [file Data_Sheet_1.zip › ECG recorded at 25 mm pr s during pool apnea/Subject 4 image 1.JPG]

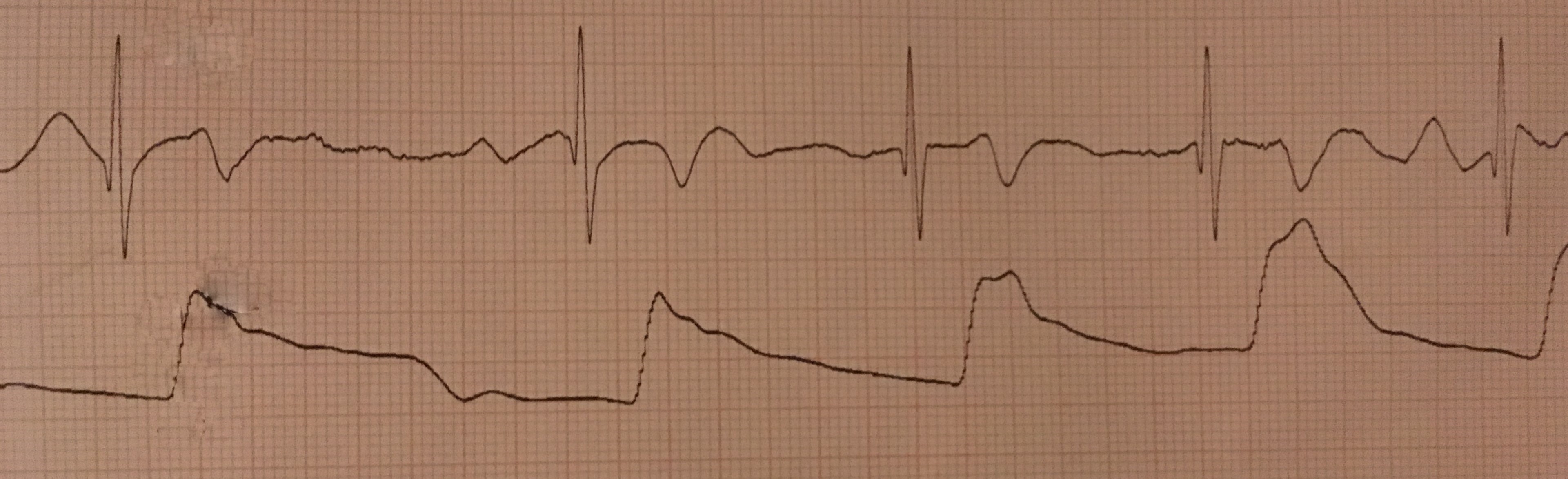

Supplement: Supplementary file 1 [file Data_Sheet_1.zip › ECG recorded at 25 mm pr s during pool apnea/Subject 4 image 2.JPG]

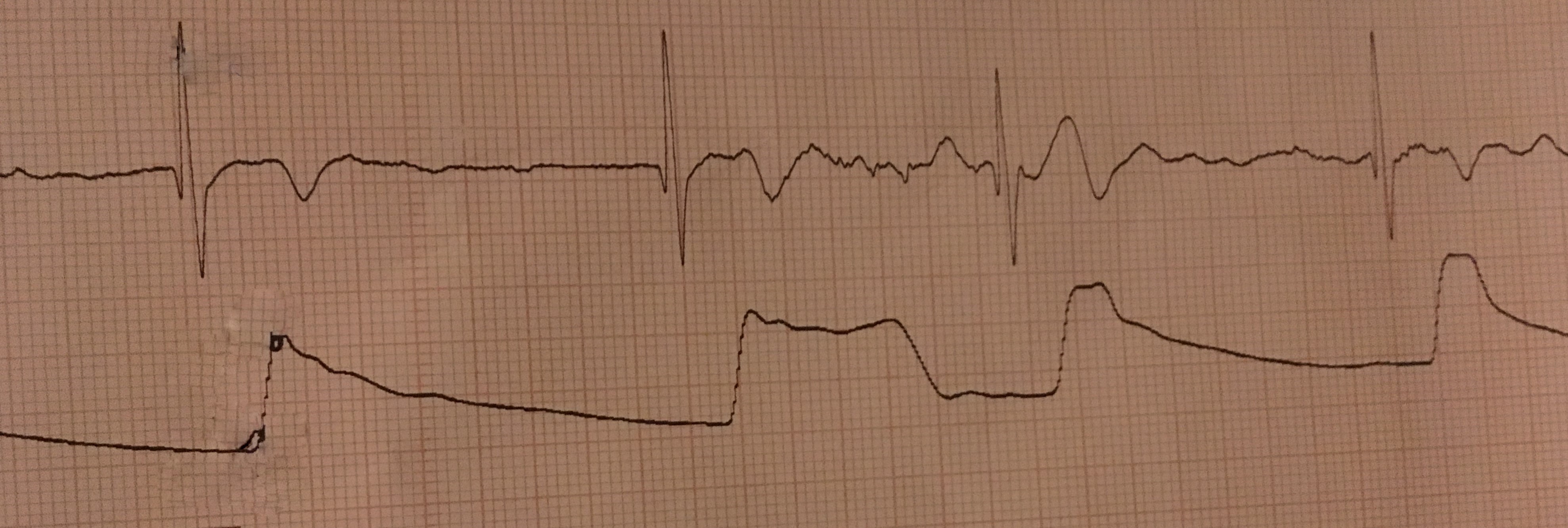

Supplement: Supplementary file 1 [file Data_Sheet_1.zip › ECG recorded at 25 mm pr s during pool apnea/Subject 4 image 3.JPG]

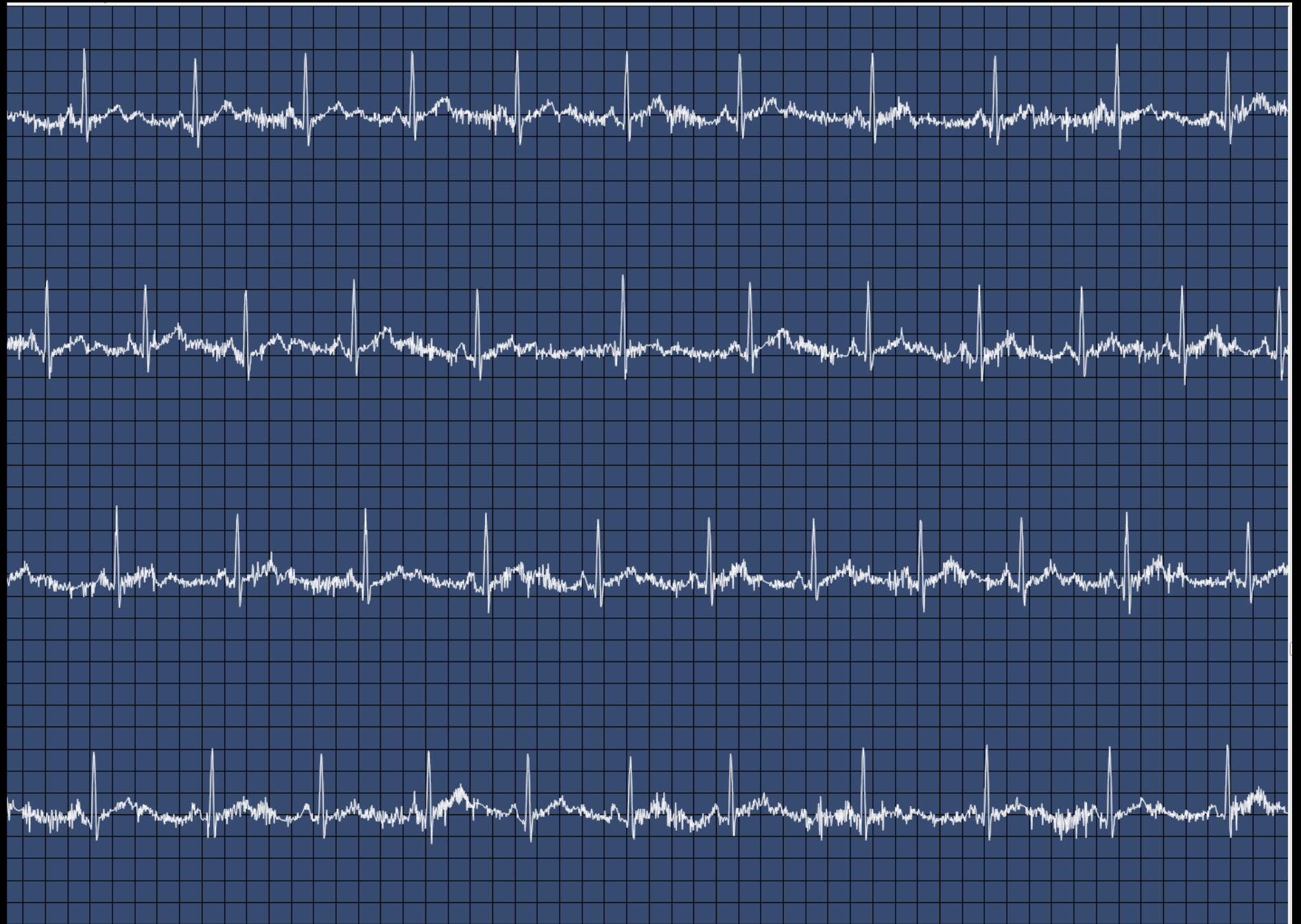

Supplement: Supplementary file 2 [file Data_Sheet_2.zip › EKG blindede/Subject 1 rest + max apnoea/1 max apnoea V5.pdf]

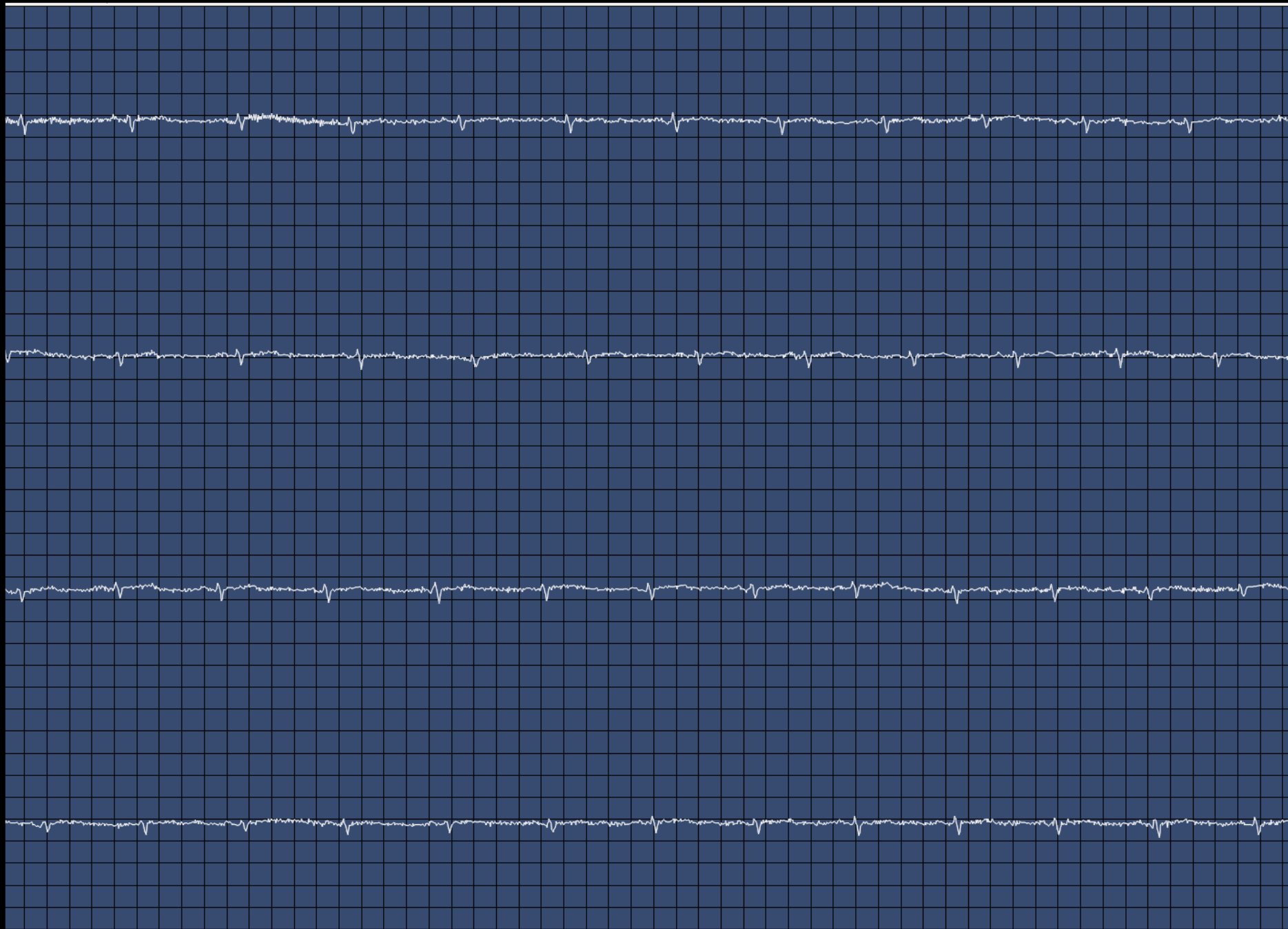

Supplement: Supplementary file 2 [file Data_Sheet_2.zip › EKG blindede/Subject 1 rest + max apnoea/1 rest I.pdf]

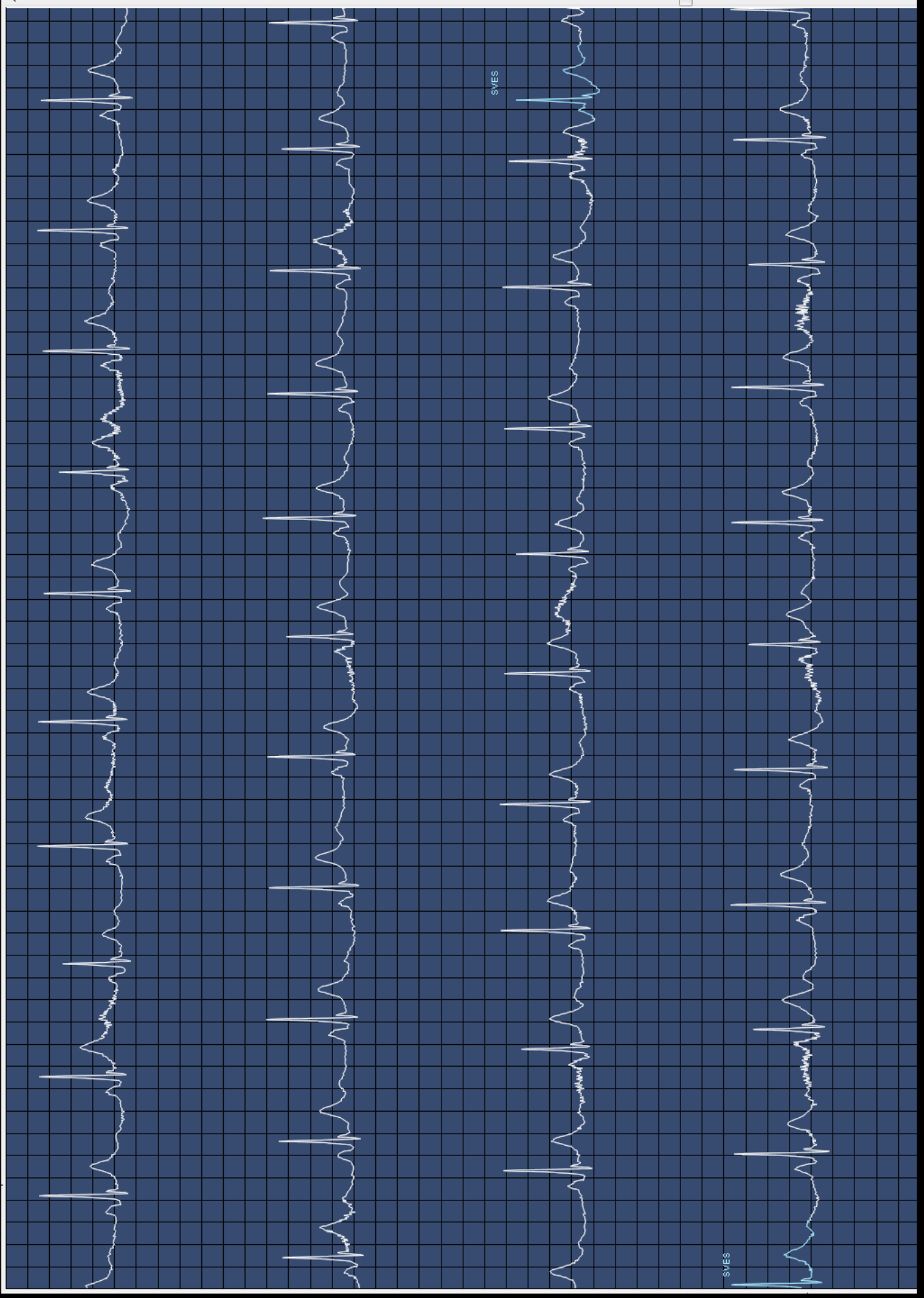

Supplement: Supplementary file 2 [file Data_Sheet_2.zip › EKG blindede/Subject 2 rest + max apnoea/2 max apnoea aVF.pdf]

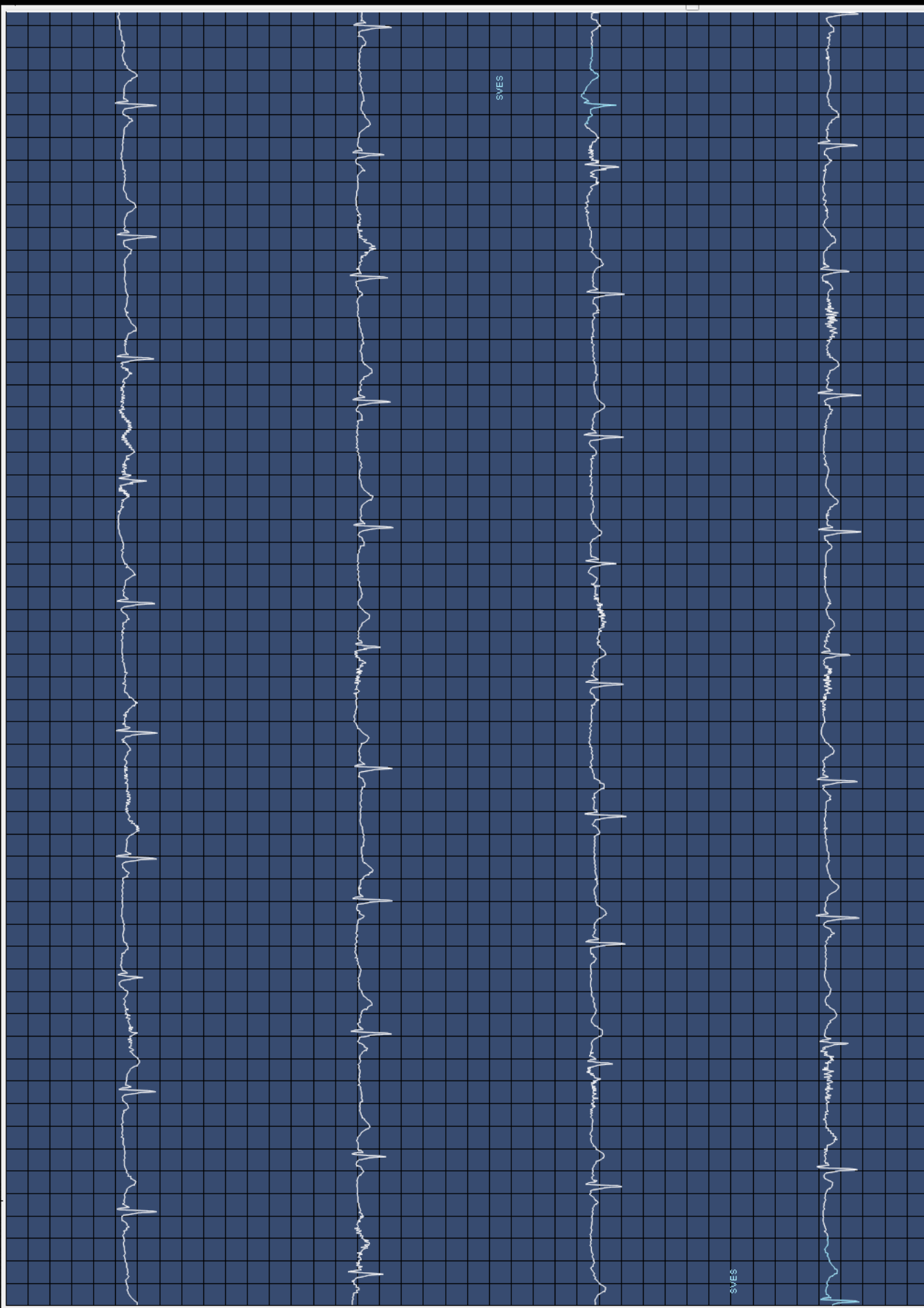

Supplement: Supplementary file 2 [file Data_Sheet_2.zip › EKG blindede/Subject 2 rest + max apnoea/2 max apnoea aVL.pdf]

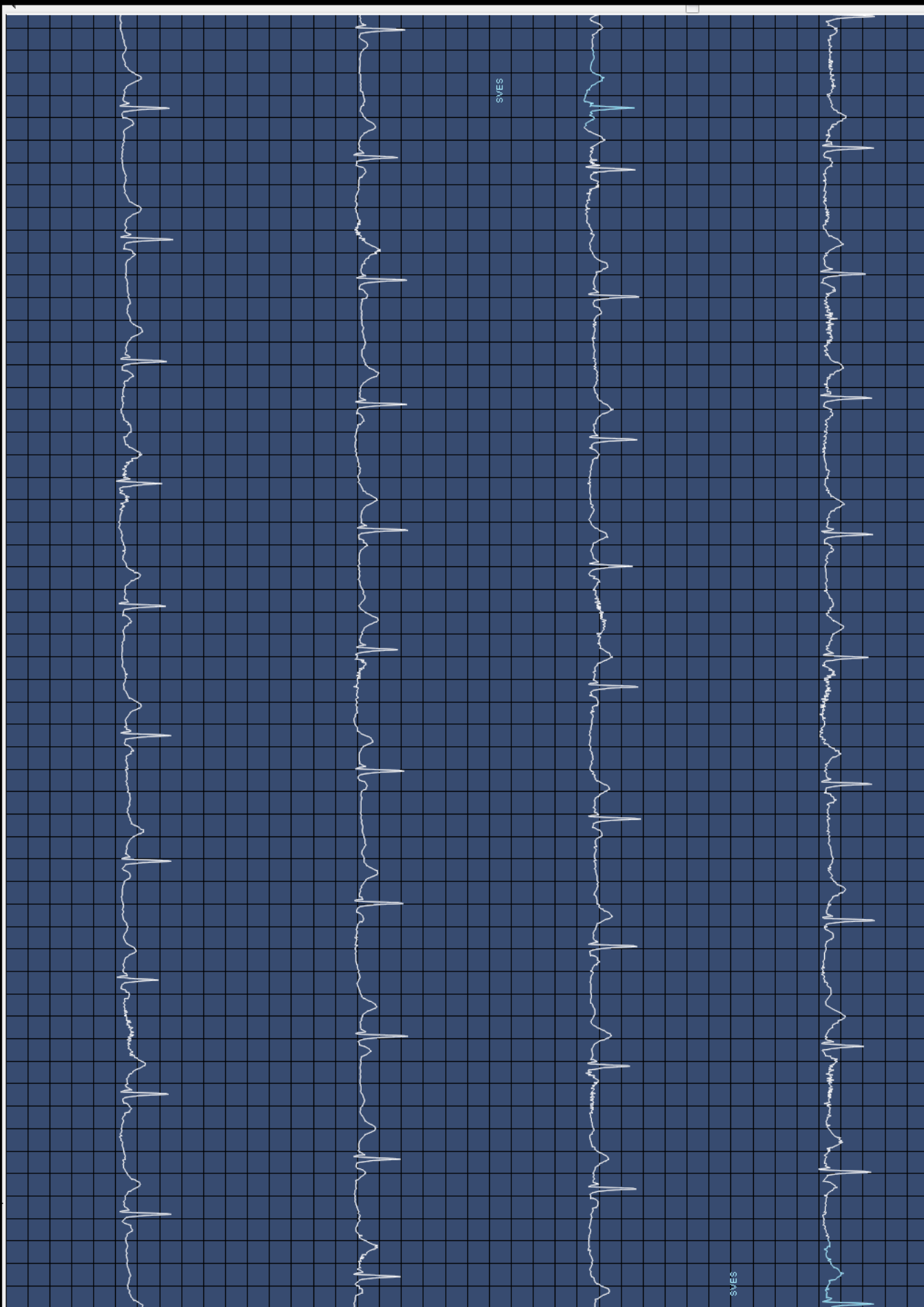

Supplement: Supplementary file 2 [file Data_Sheet_2.zip › EKG blindede/Subject 2 rest + max apnoea/2 max apnoea aVR.pdf]

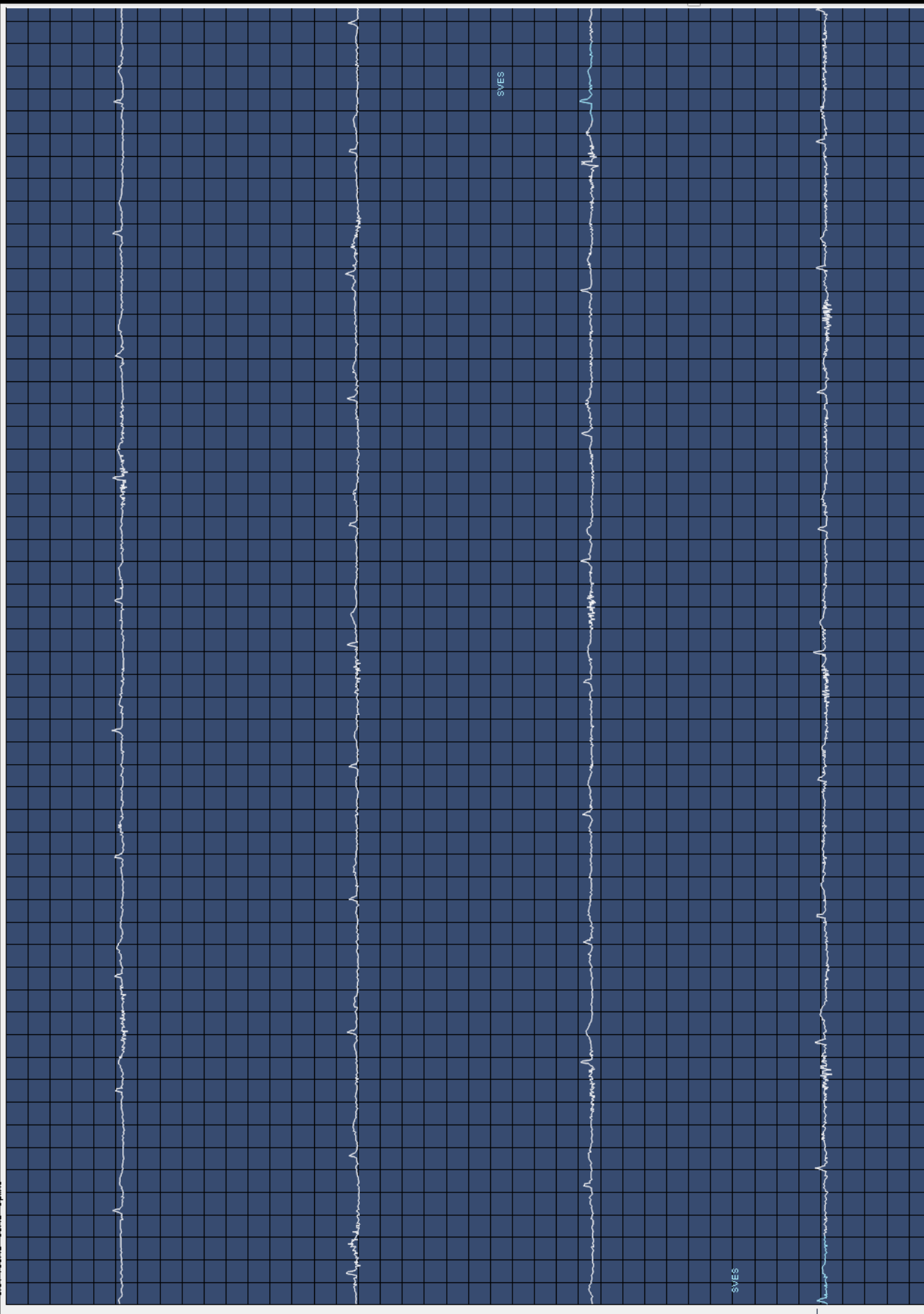

SVES

SVES

Supplement: Supplementary file 2 [file Data_Sheet_2.zip › EKG blindede/Subject 2 rest + max apnoea/2 max apnoea I.pdf]

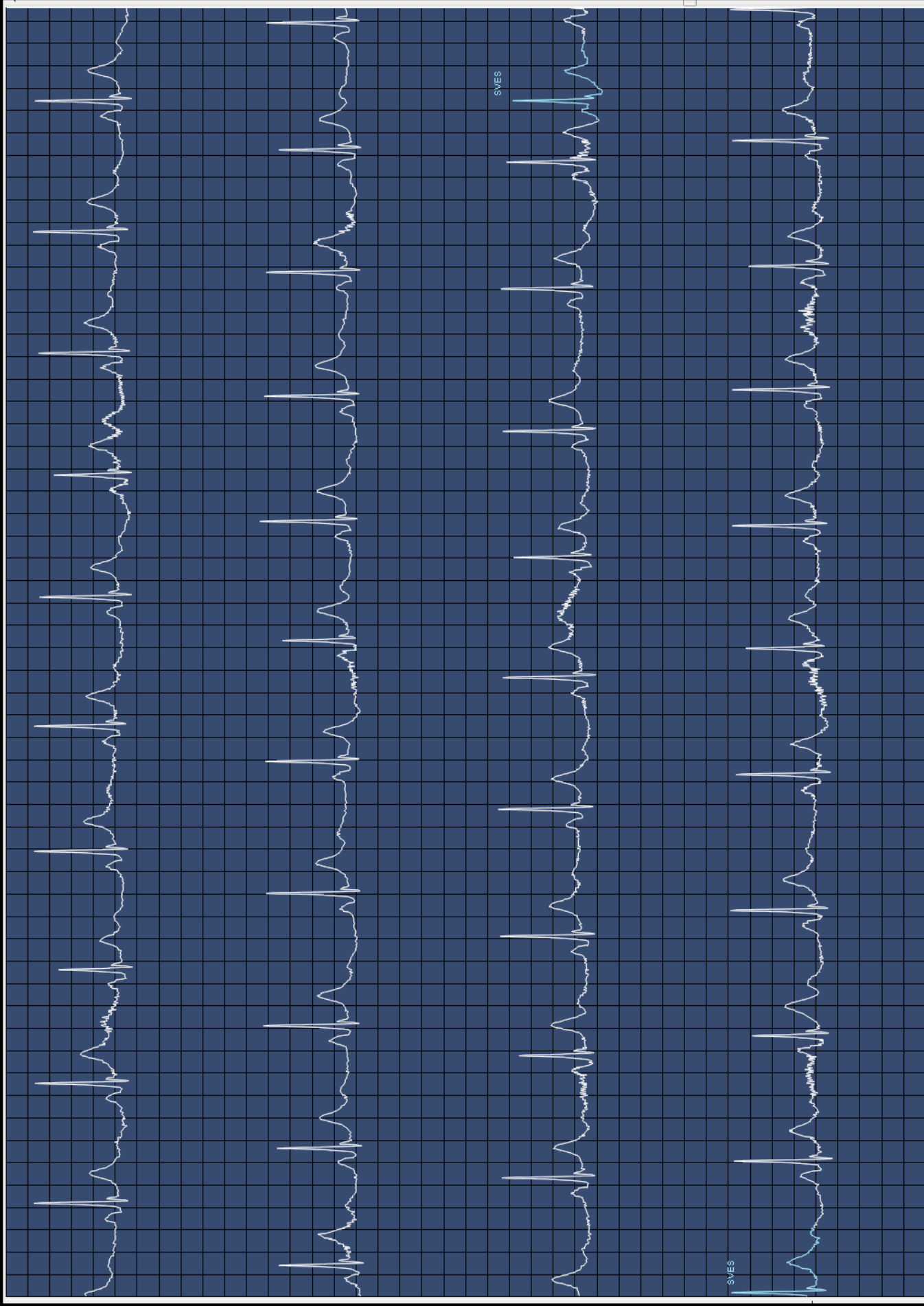

Supplement: Supplementary file 2 [file Data_Sheet_2.zip › EKG blindede/Subject 2 rest + max apnoea/2 max apnoea II.pdf]

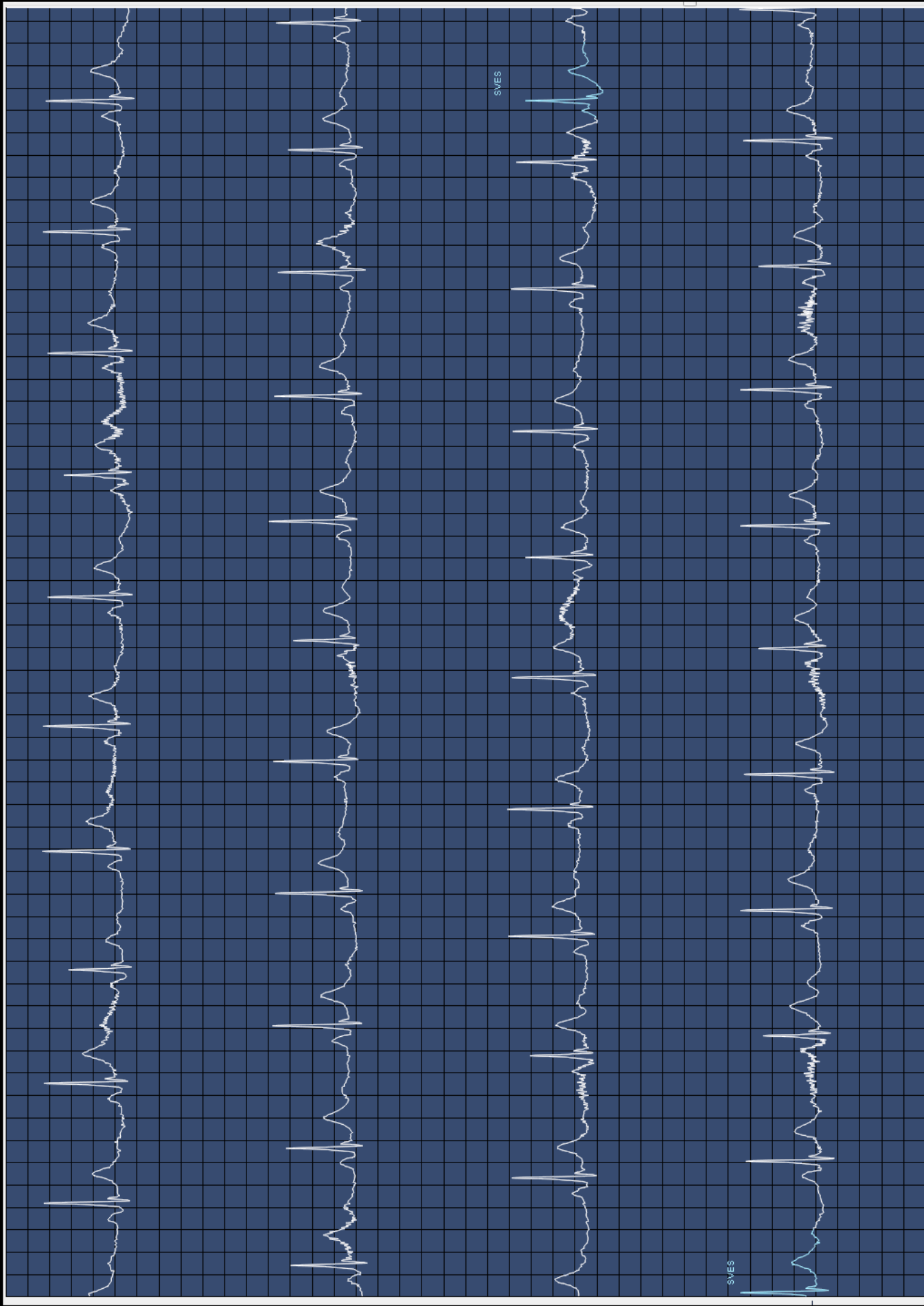

Supplement: Supplementary file 2 [file Data_Sheet_2.zip › EKG blindede/Subject 2 rest + max apnoea/2 max apnoea III.pdf]

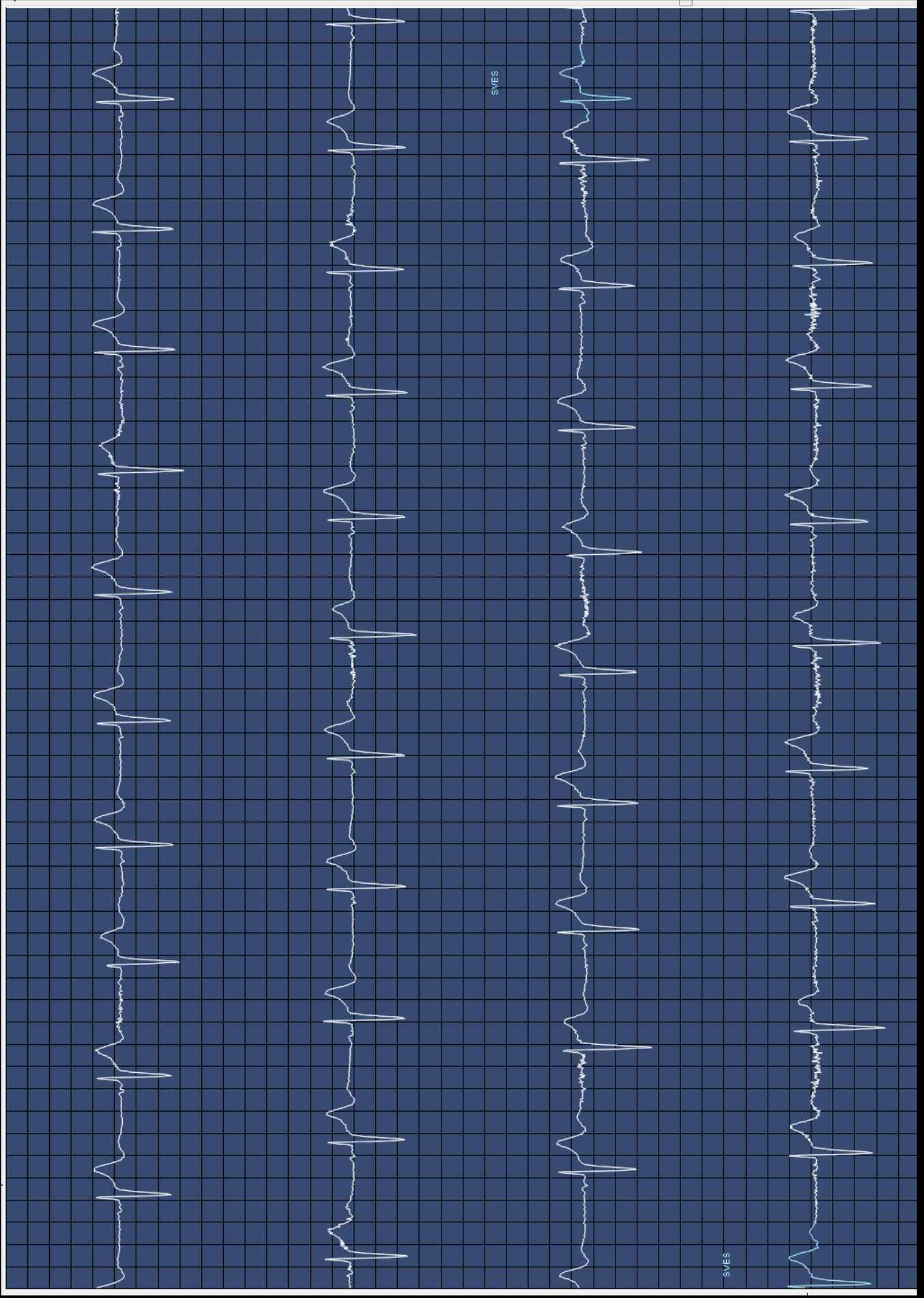

Supplement: Supplementary file 2 [file Data_Sheet_2.zip › EKG blindede/Subject 2 rest + max apnoea/2 max apnoea V1.pdf]

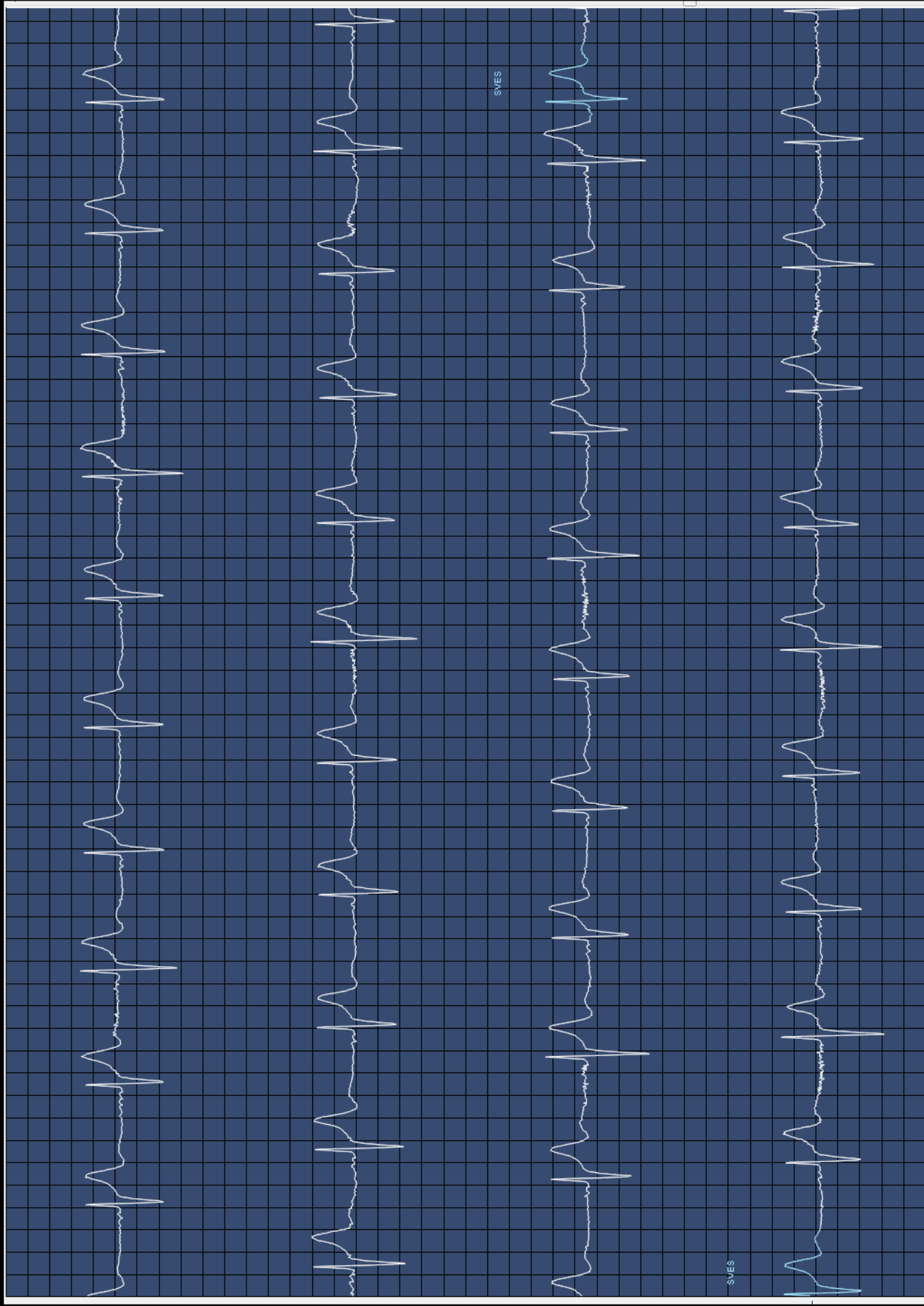

Supplement: Supplementary file 2 [file Data_Sheet_2.zip › EKG blindede/Subject 2 rest + max apnoea/2 max apnoea V2.pdf]

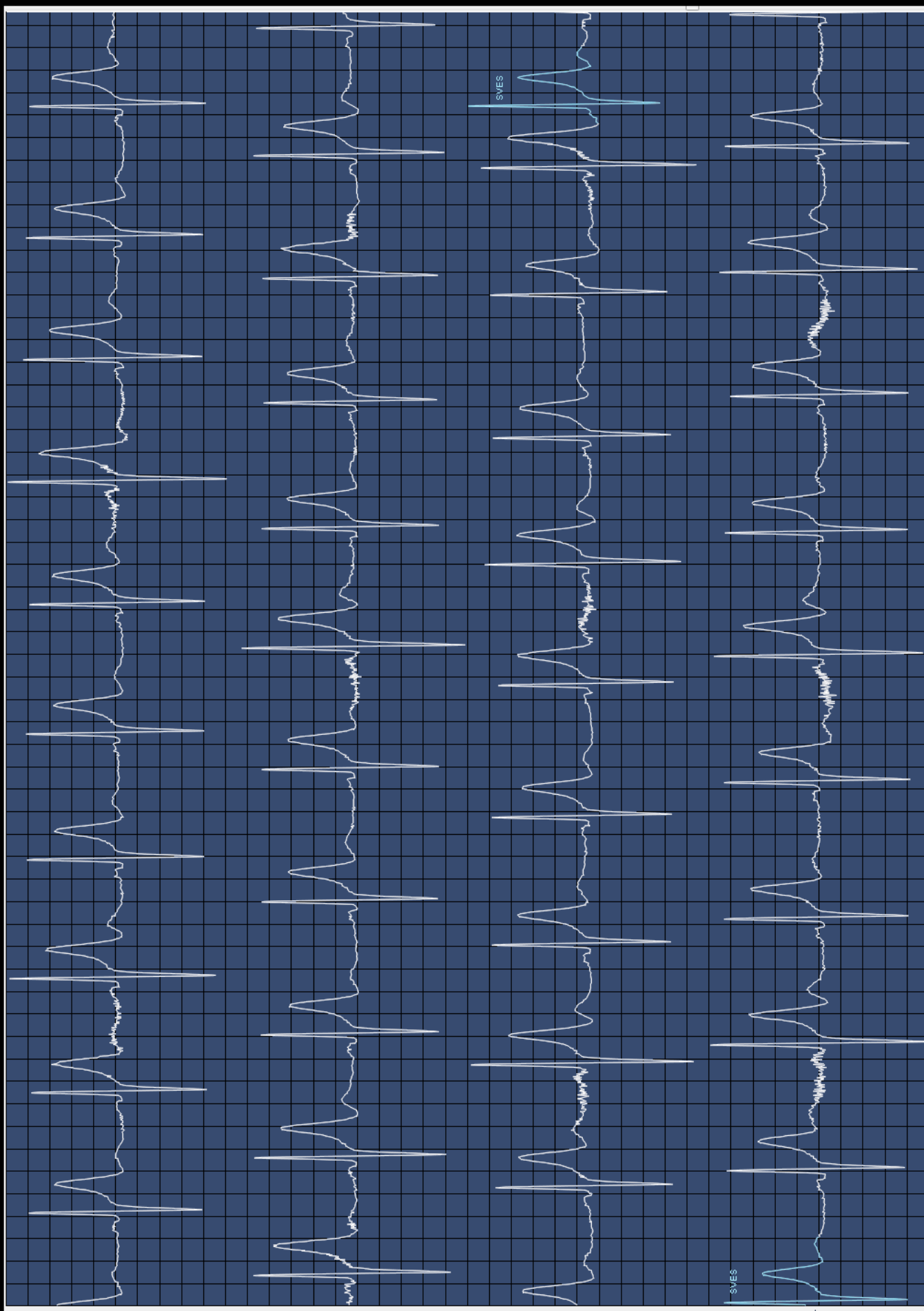

Supplement: Supplementary file 2 [file Data_Sheet_2.zip › EKG blindede/Subject 2 rest + max apnoea/2 max apnoea V3.pdf]

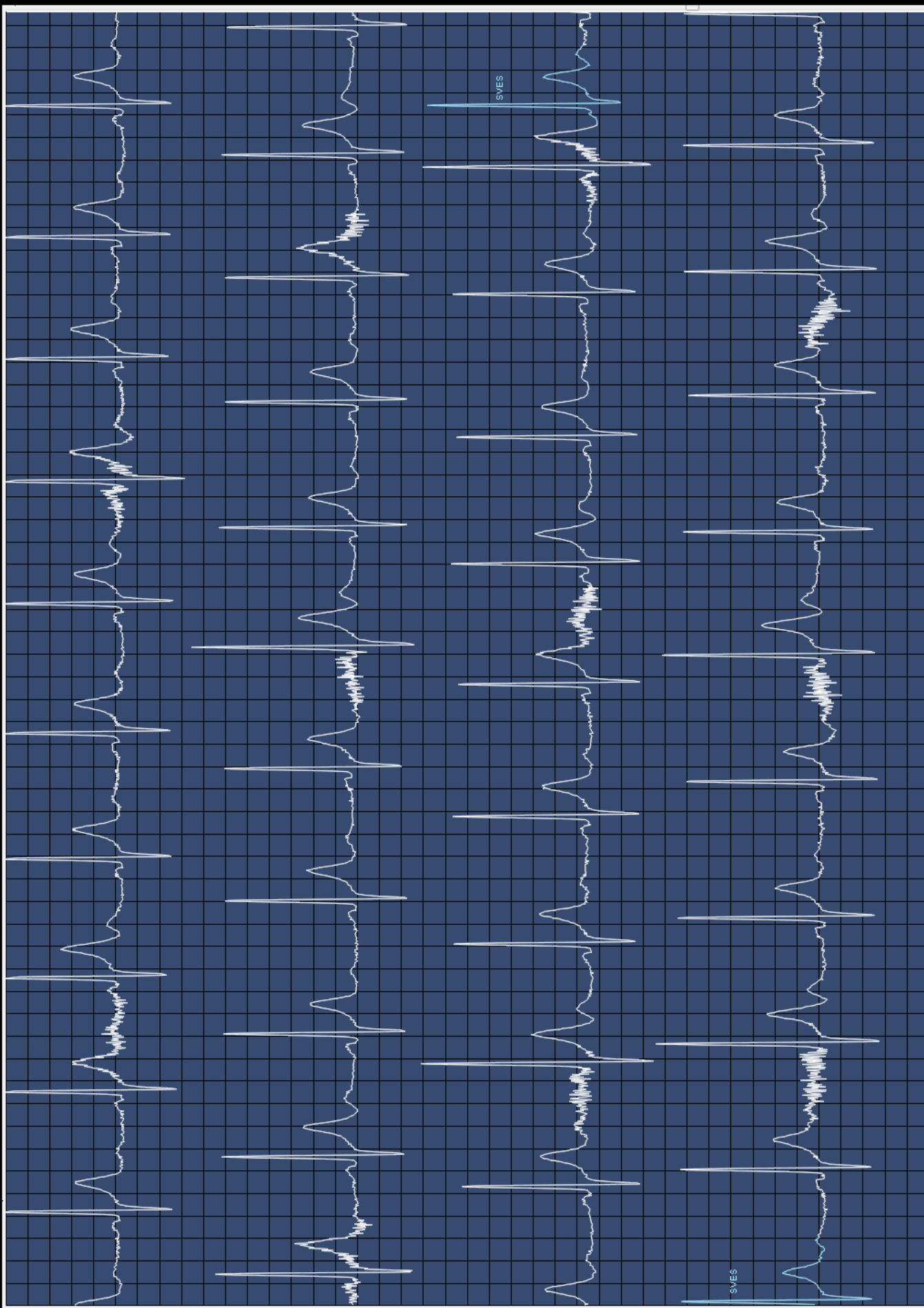

Supplement: Supplementary file 2 [file Data_Sheet_2.zip › EKG blindede/Subject 2 rest + max apnoea/2 max apnoea V4.pdf]

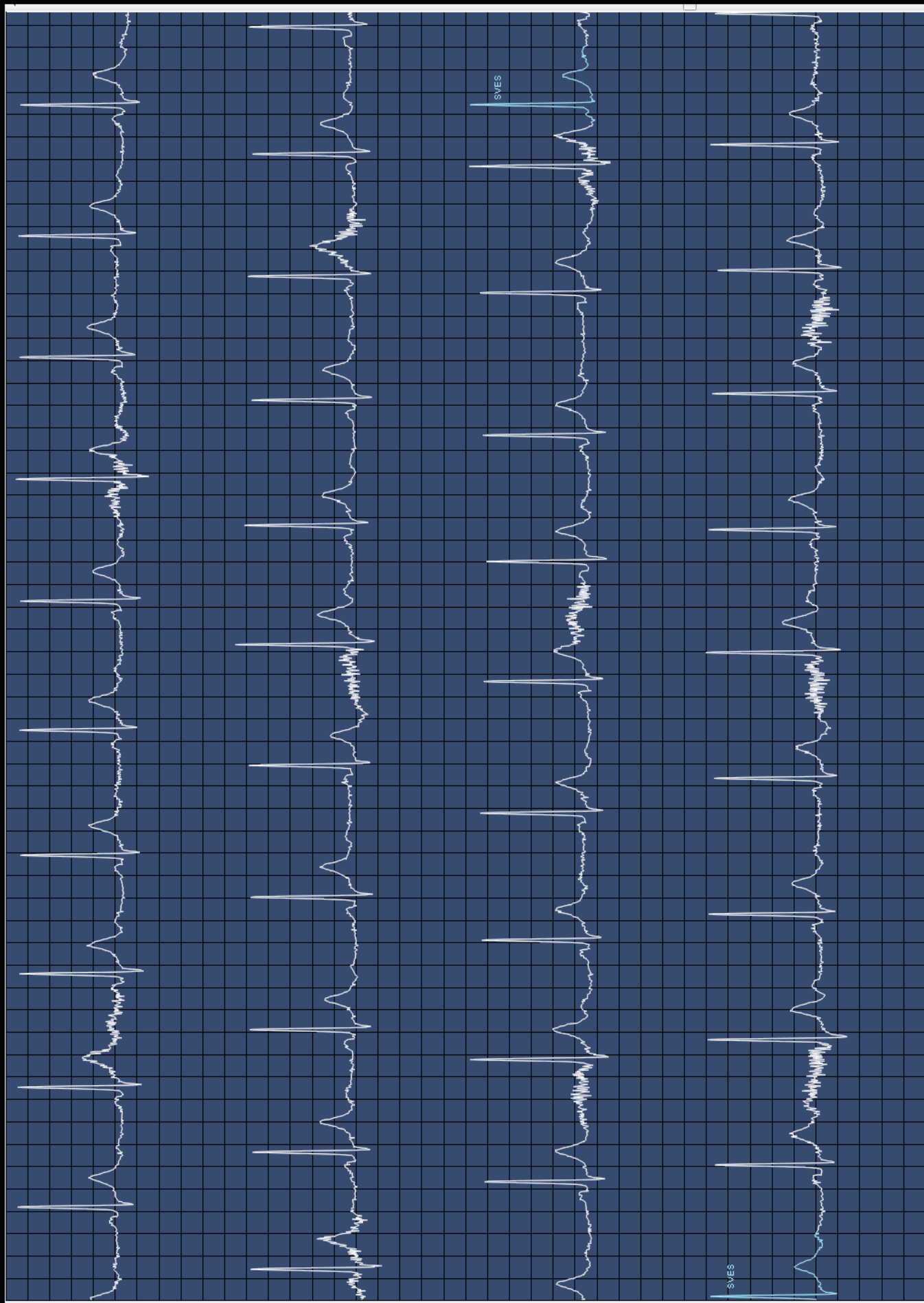

Supplement: Supplementary file 2 [file Data_Sheet_2.zip › EKG blindede/Subject 2 rest + max apnoea/2 max apnoea V5.pdf]

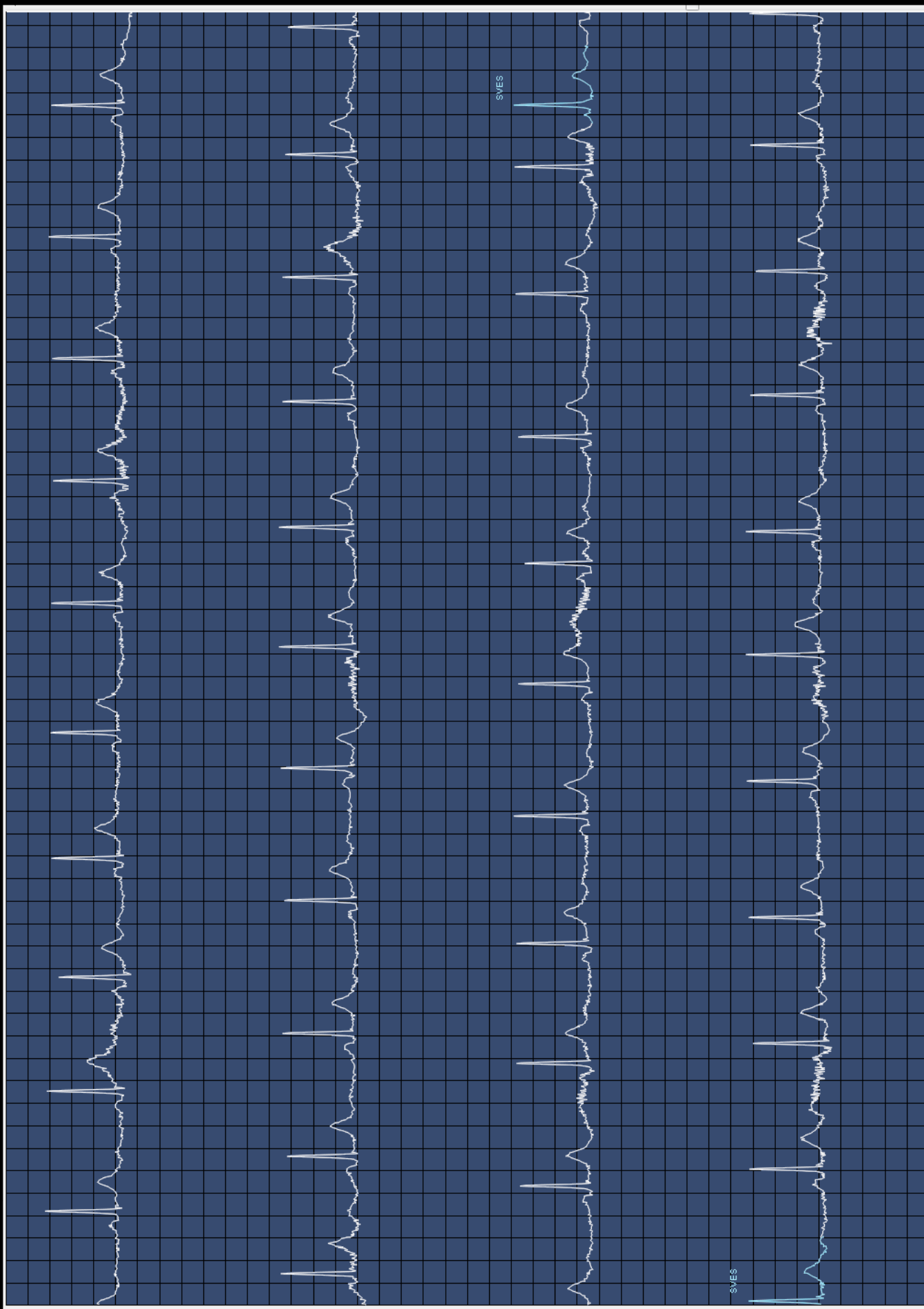

Supplement: Supplementary file 2 [file Data_Sheet_2.zip › EKG blindede/Subject 2 rest + max apnoea/2 max apnoea V6.pdf]

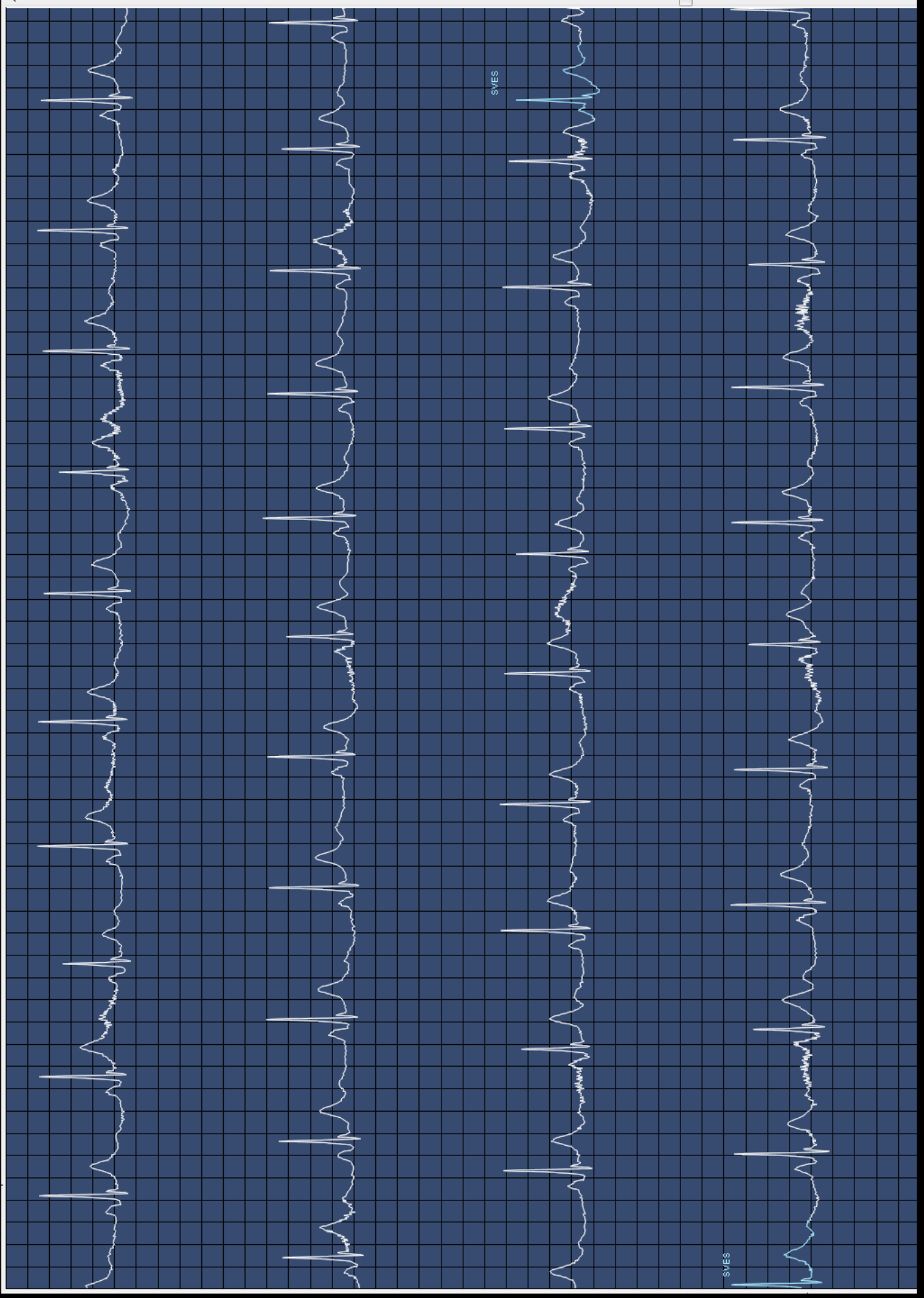

Supplement: Supplementary file 2 [file Data_Sheet_2.zip › EKG blindede/Subject 2 rest + max apnoea/2 rest aVF.pdf]

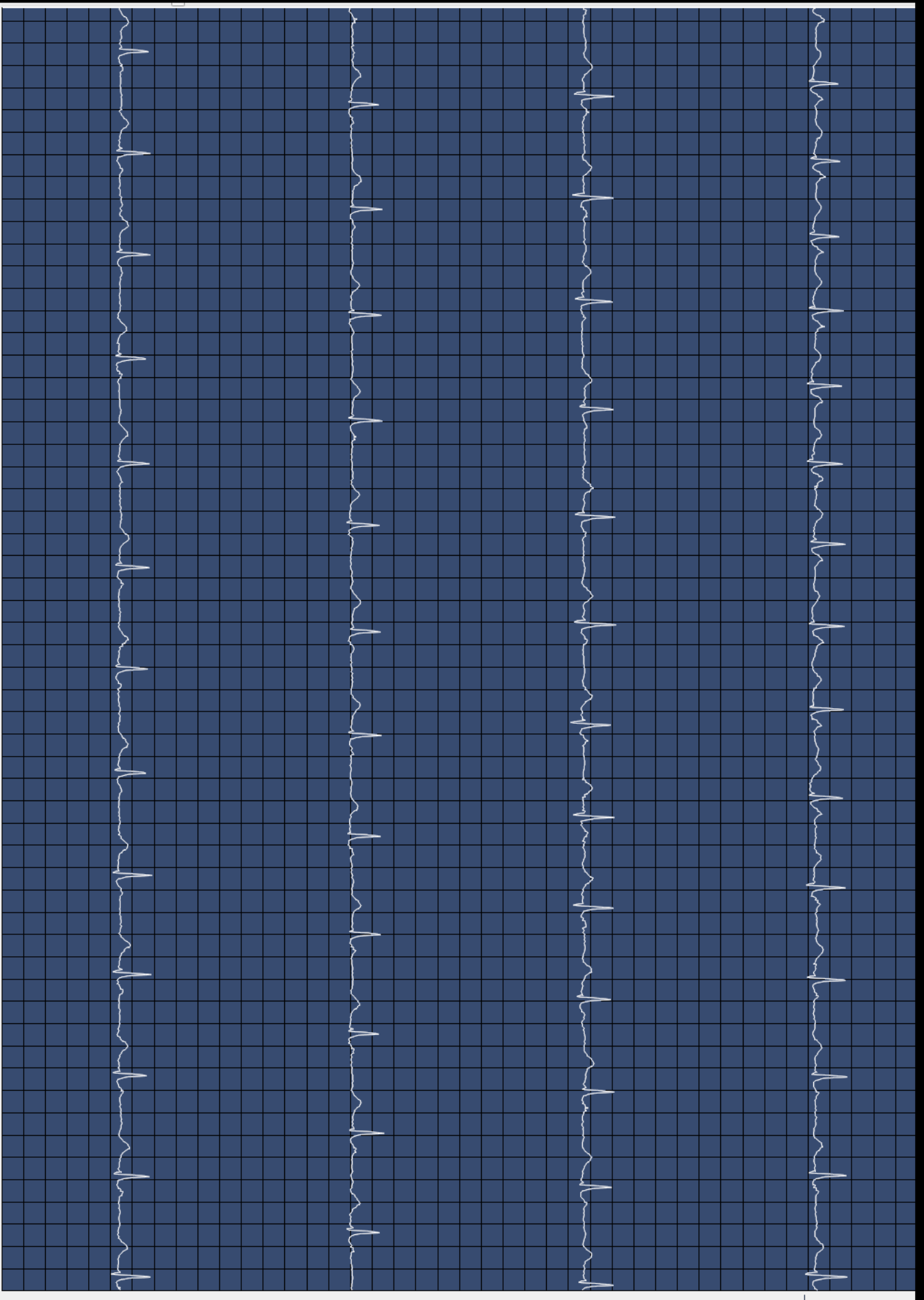

Supplement: Supplementary file 2 [file Data_Sheet_2.zip › EKG blindede/Subject 2 rest + max apnoea/2 rest aVL.pdf]

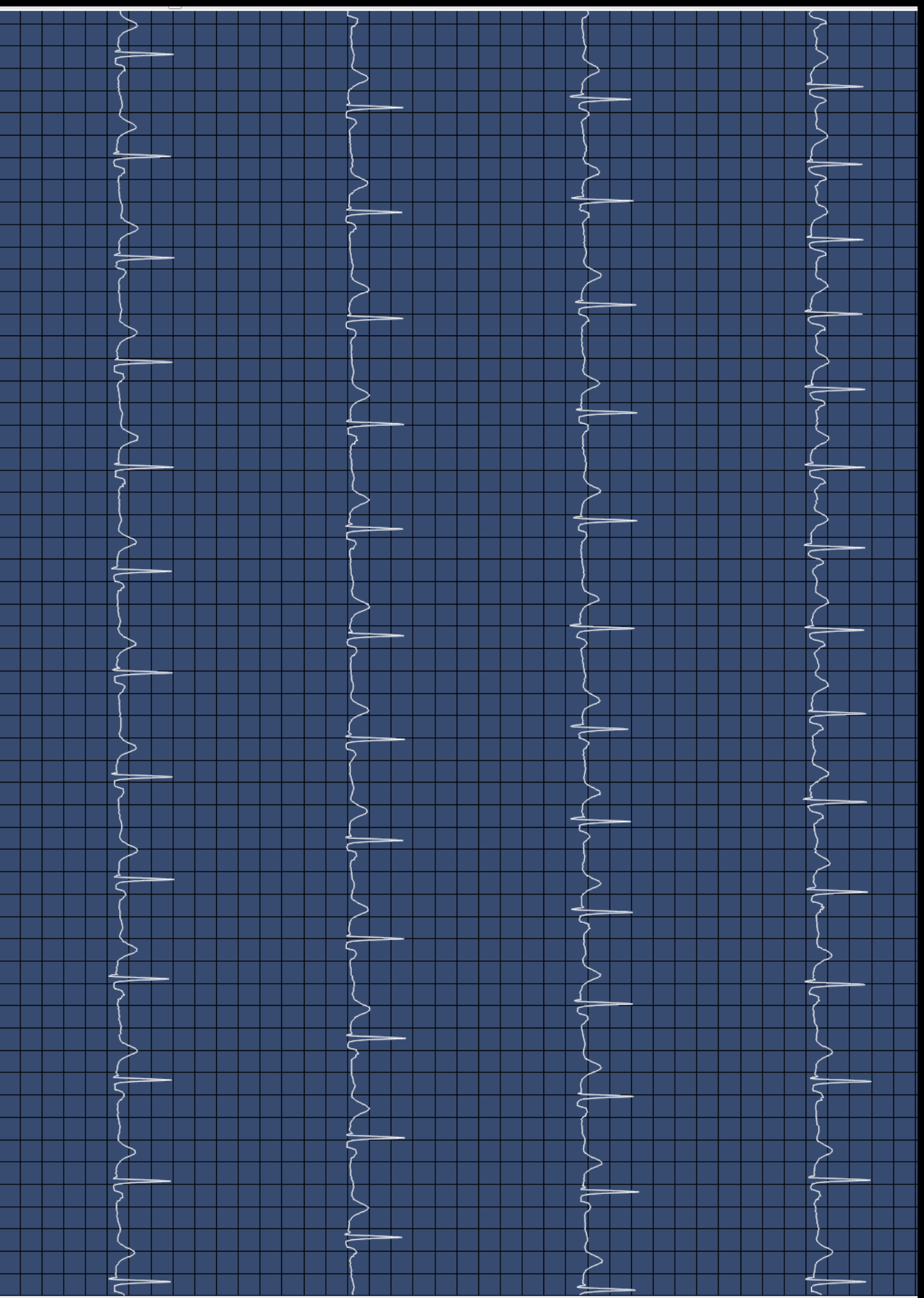

Supplement: Supplementary file 2 [file Data_Sheet_2.zip › EKG blindede/Subject 2 rest + max apnoea/2 rest aVR.pdf]

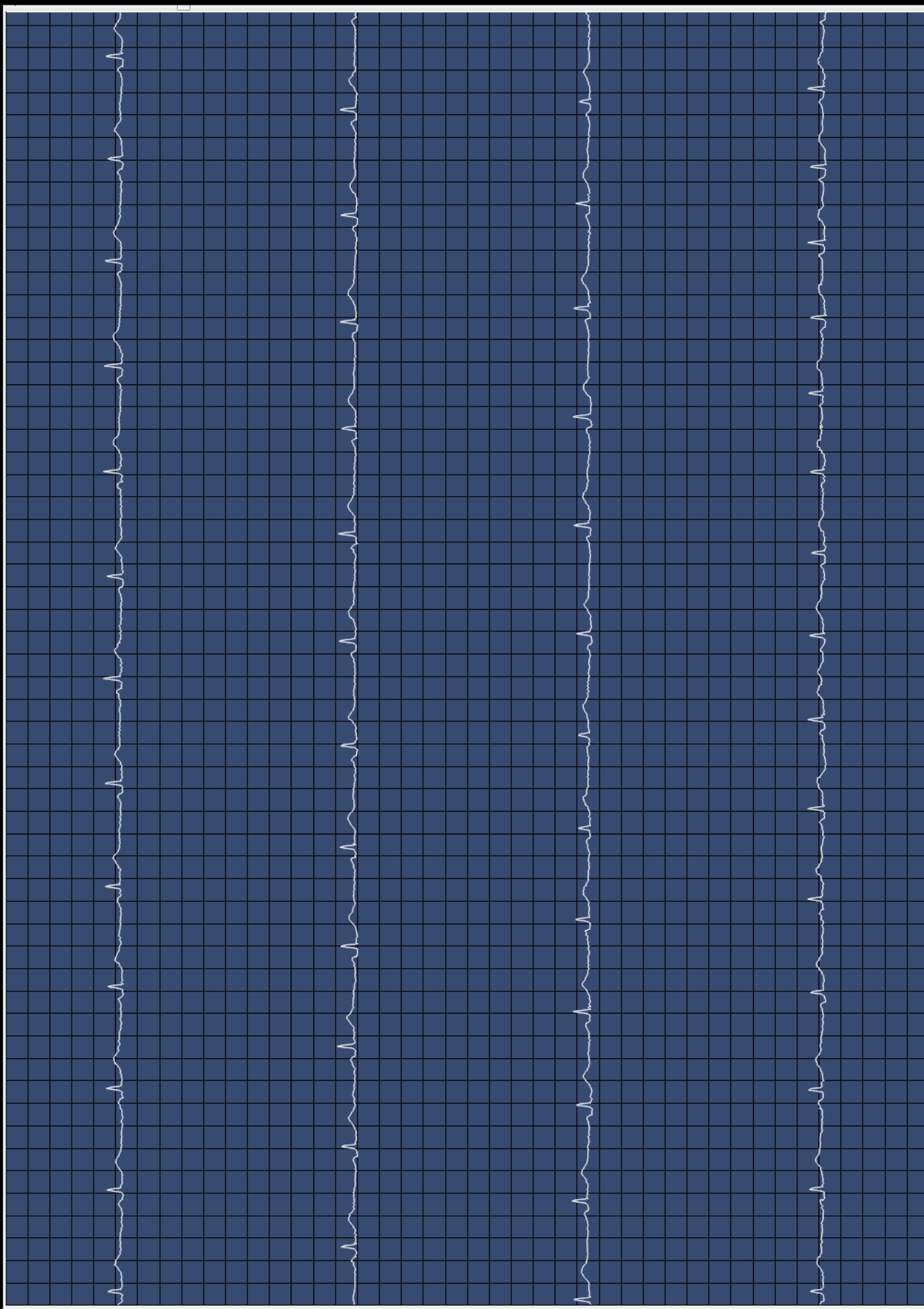

Supplement: Supplementary file 2 [file Data_Sheet_2.zip › EKG blindede/Subject 2 rest + max apnoea/2 rest I.pdf]

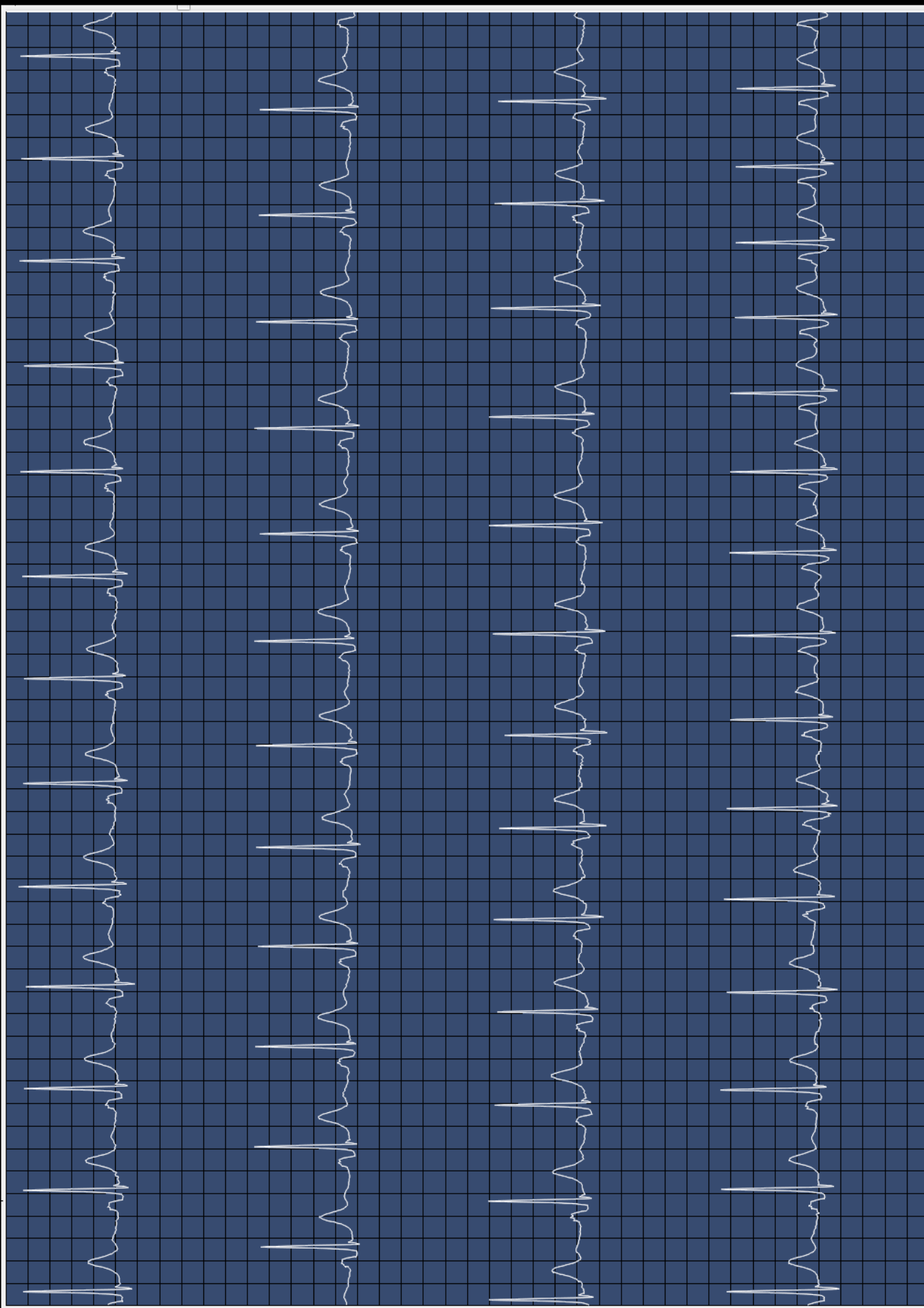

Supplement: Supplementary file 2 [file Data_Sheet_2.zip › EKG blindede/Subject 2 rest + max apnoea/2 rest II.pdf]

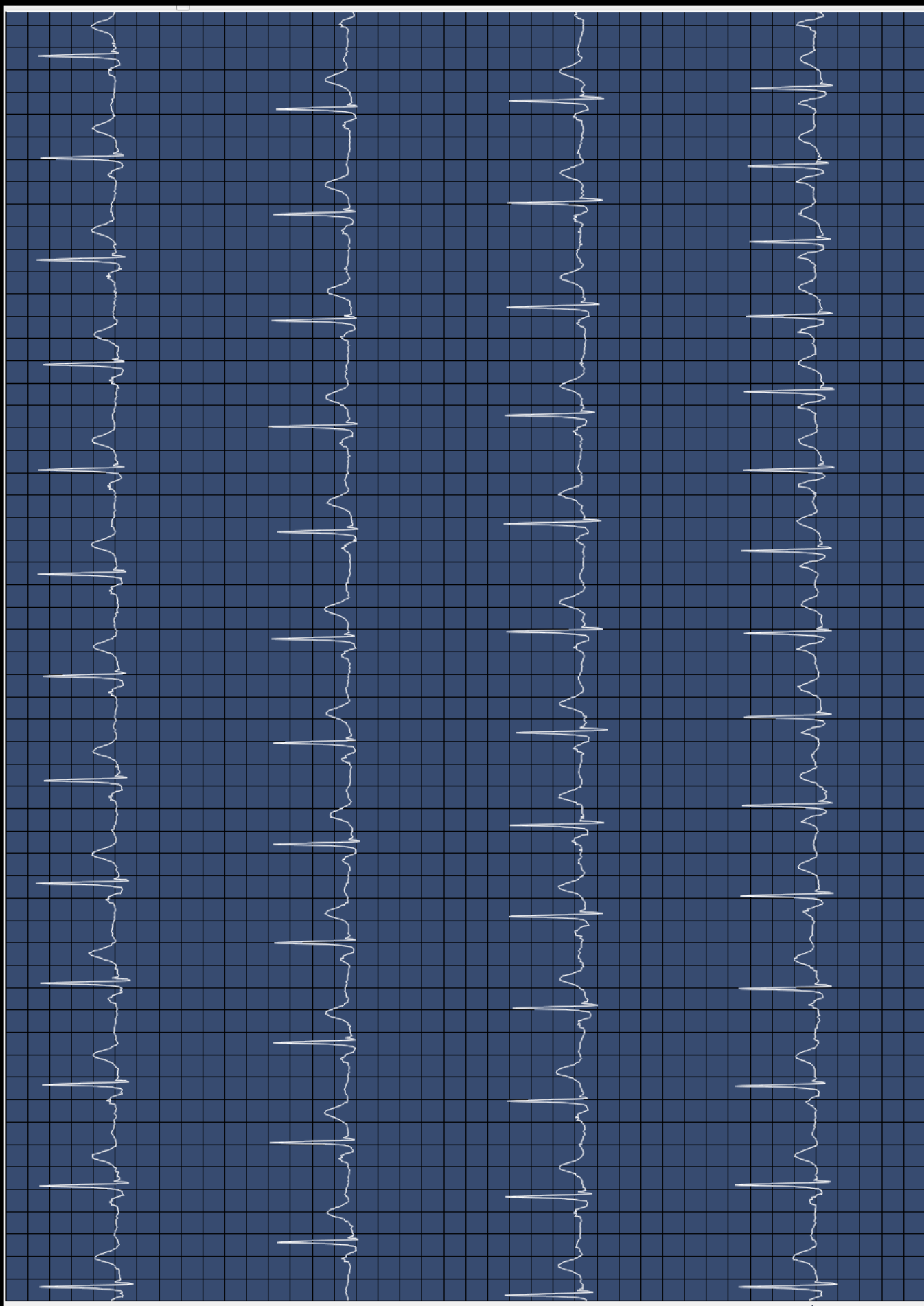

Supplement: Supplementary file 2 [file Data_Sheet_2.zip › EKG blindede/Subject 2 rest + max apnoea/2 rest III.pdf]

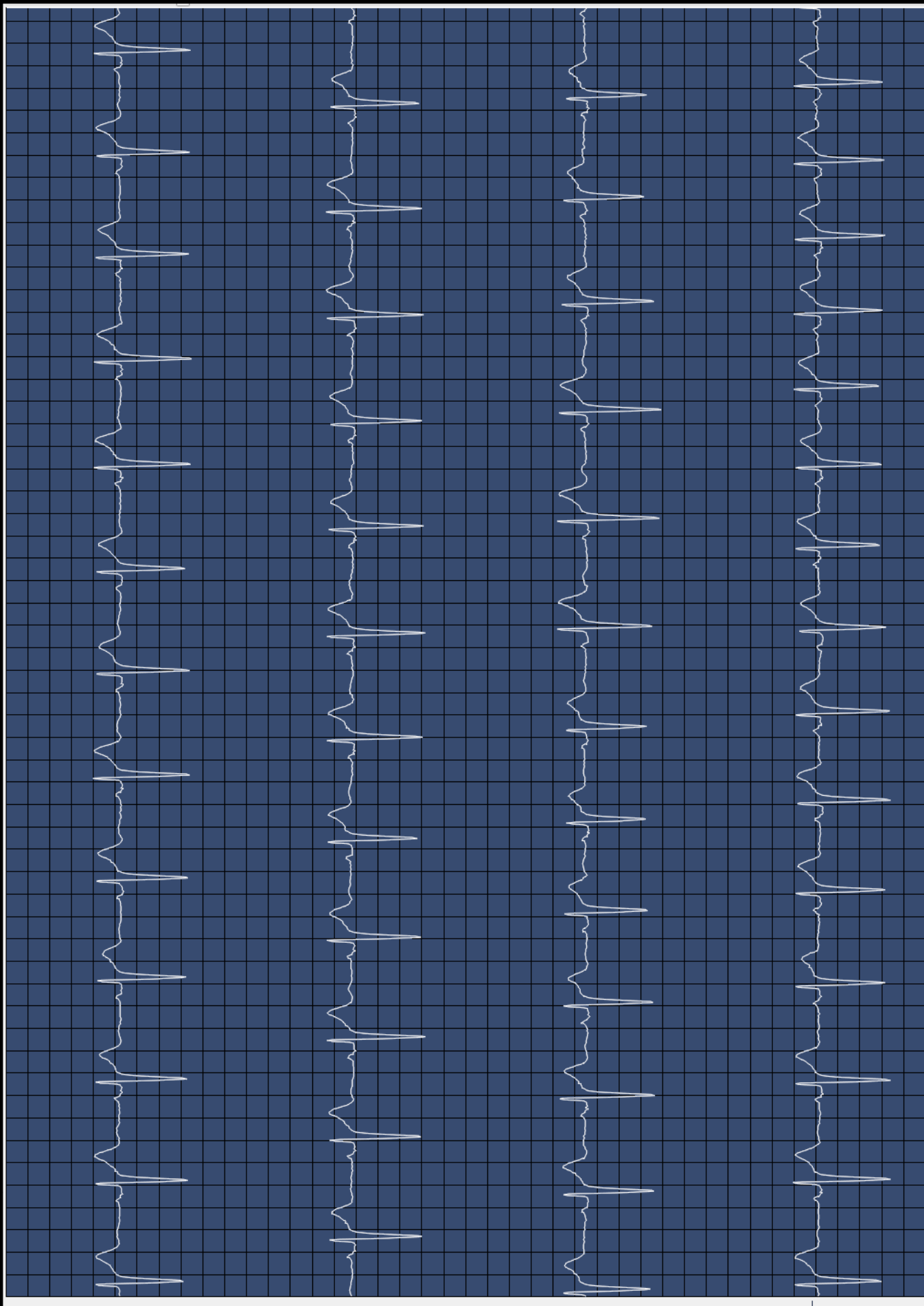

Supplement: Supplementary file 2 [file Data_Sheet_2.zip › EKG blindede/Subject 2 rest + max apnoea/2 rest V1.pdf]

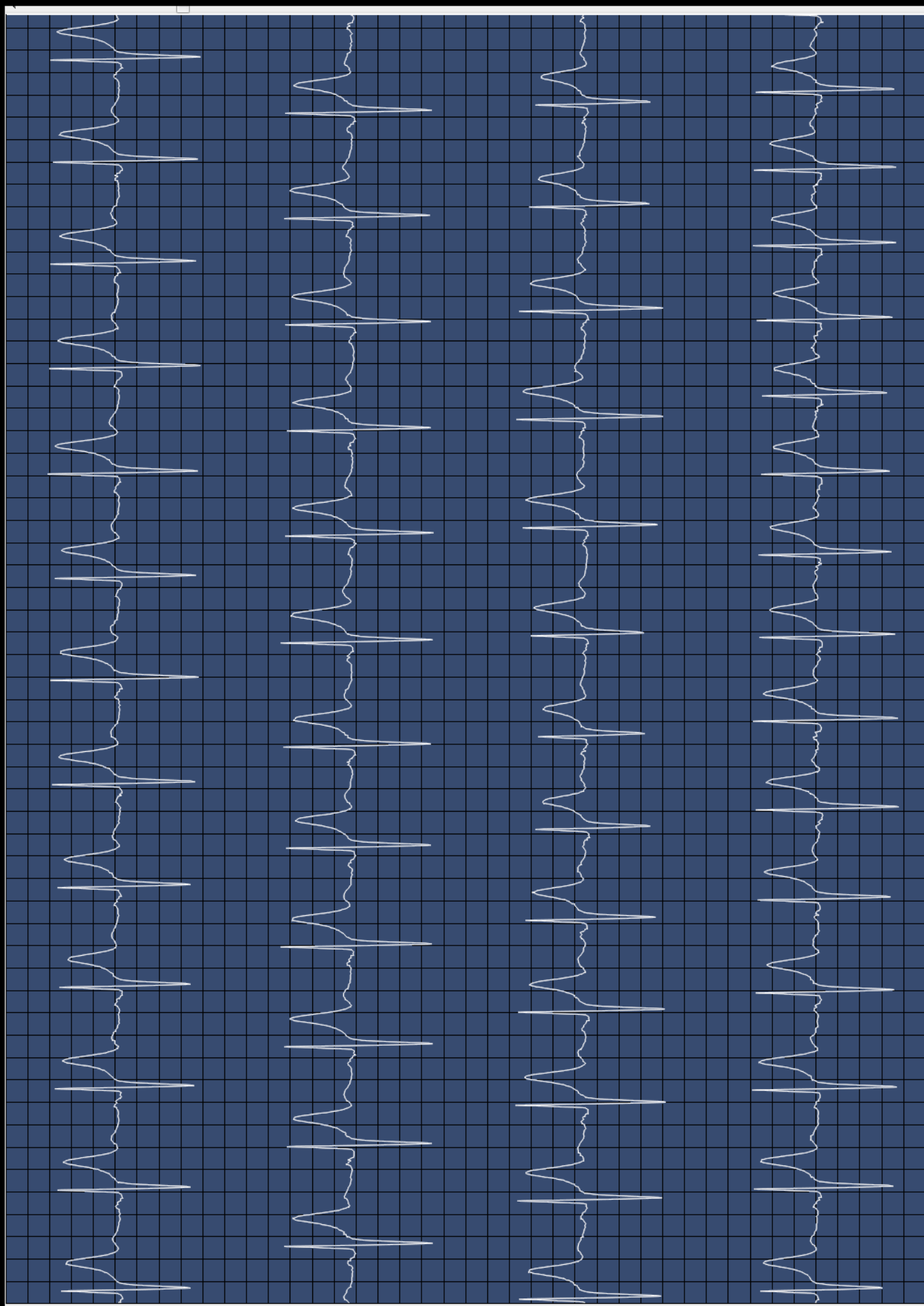

Supplement: Supplementary file 2 [file Data_Sheet_2.zip › EKG blindede/Subject 2 rest + max apnoea/2 rest V2.pdf]

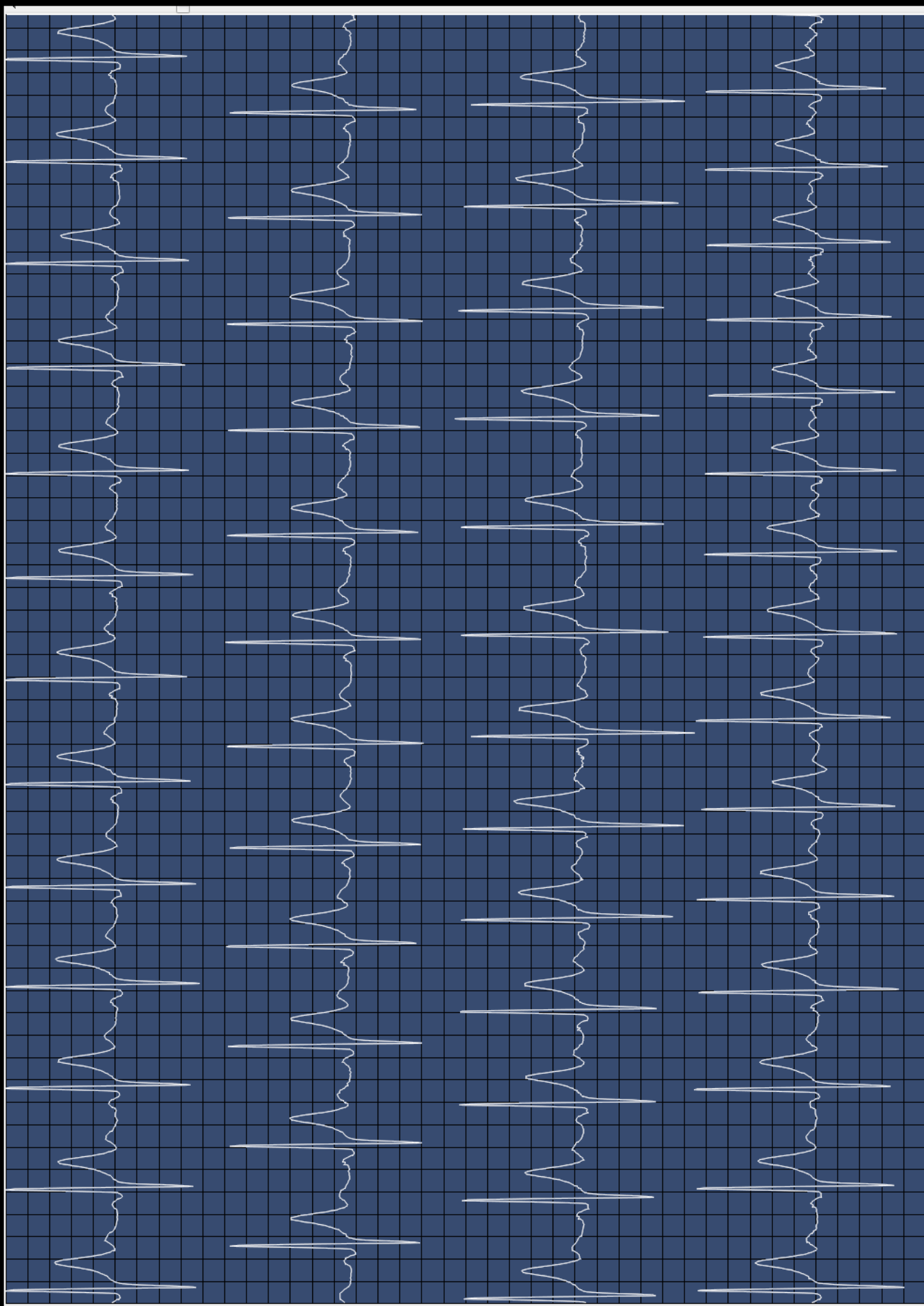

Supplement: Supplementary file 2 [file Data_Sheet_2.zip › EKG blindede/Subject 2 rest + max apnoea/2 rest V3.pdf]

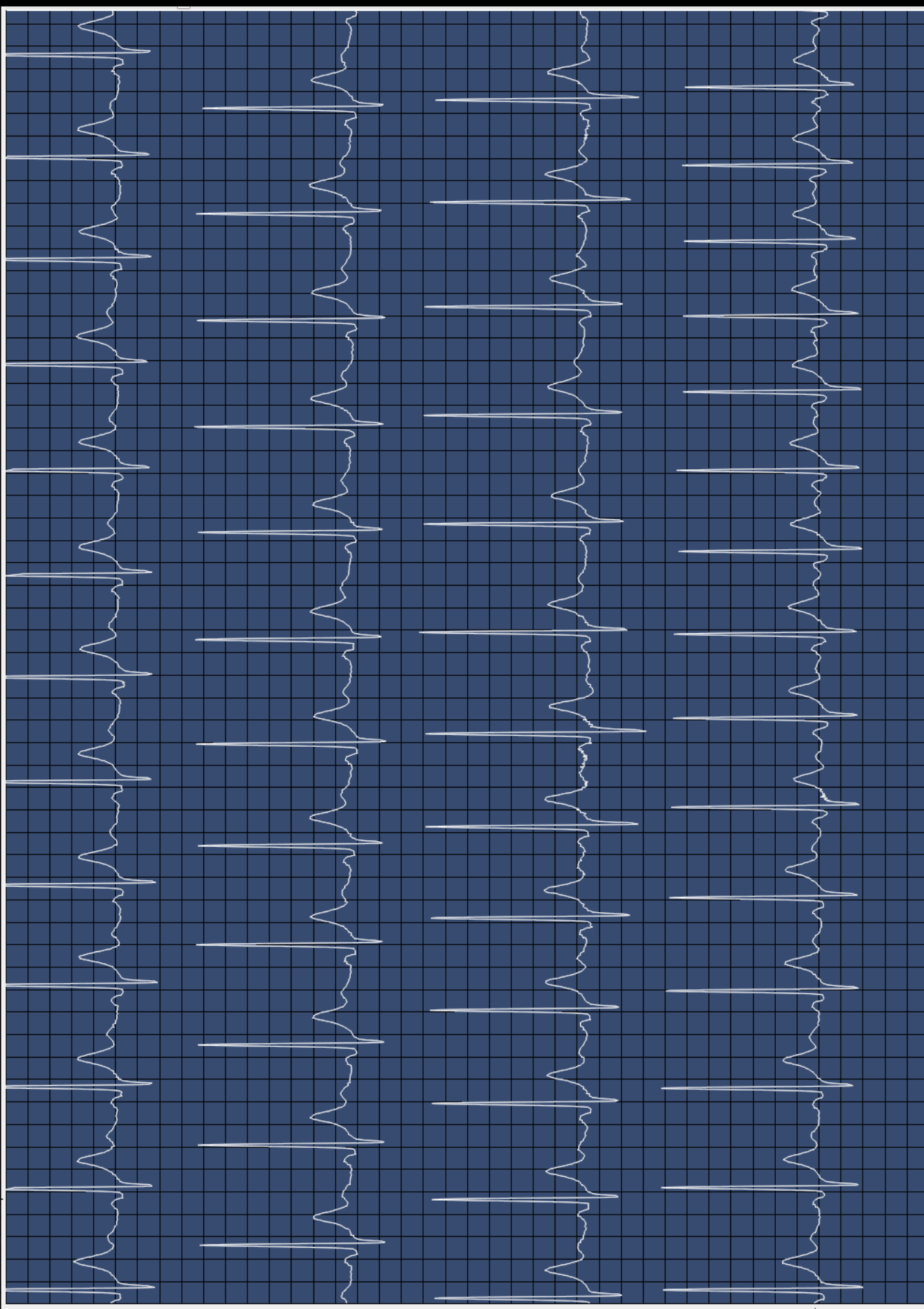

Supplement: Supplementary file 2 [file Data_Sheet_2.zip › EKG blindede/Subject 2 rest + max apnoea/2 rest V4.pdf]

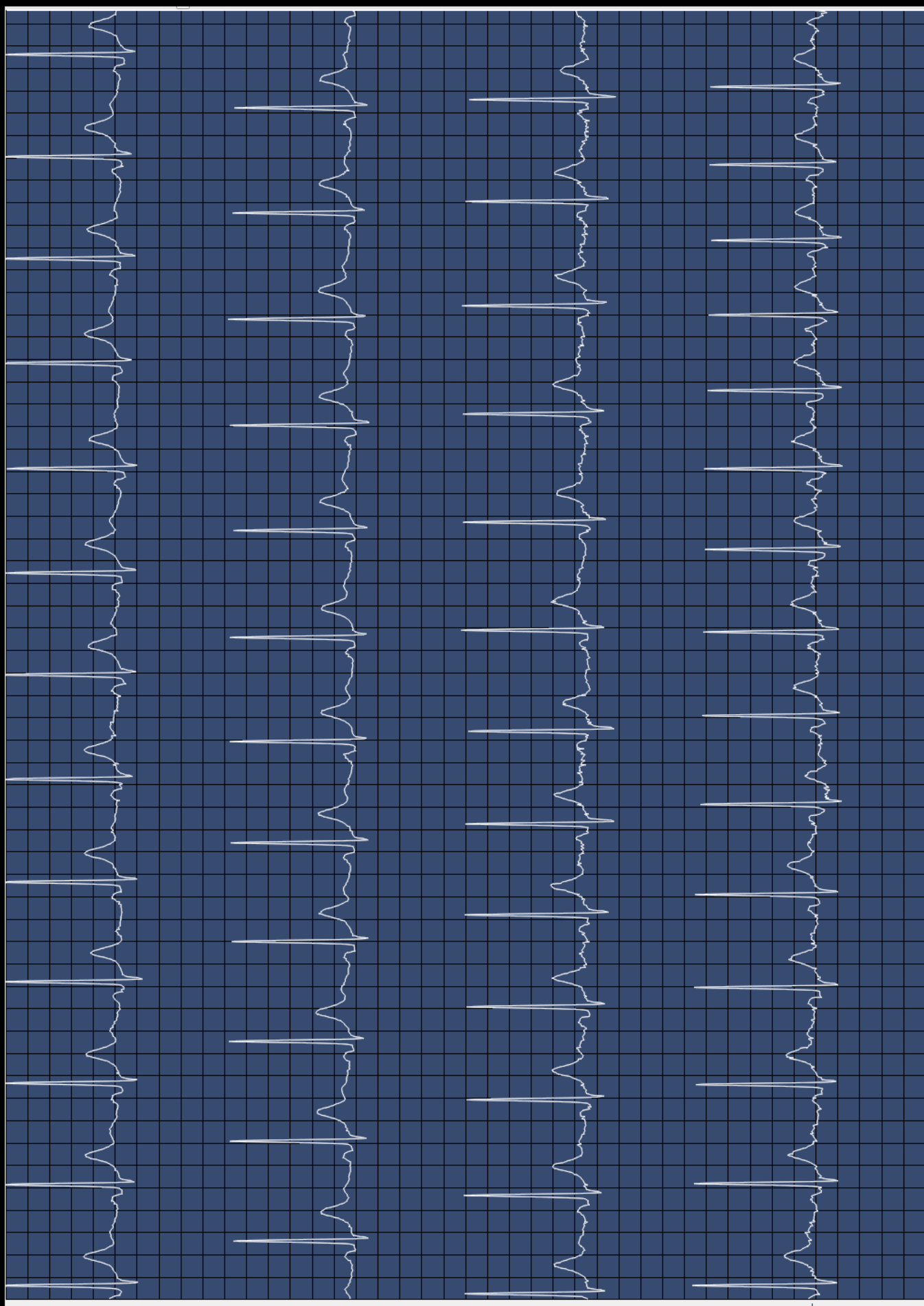

Supplement: Supplementary file 2 [file Data_Sheet_2.zip › EKG blindede/Subject 2 rest + max apnoea/2 rest V5.pdf]

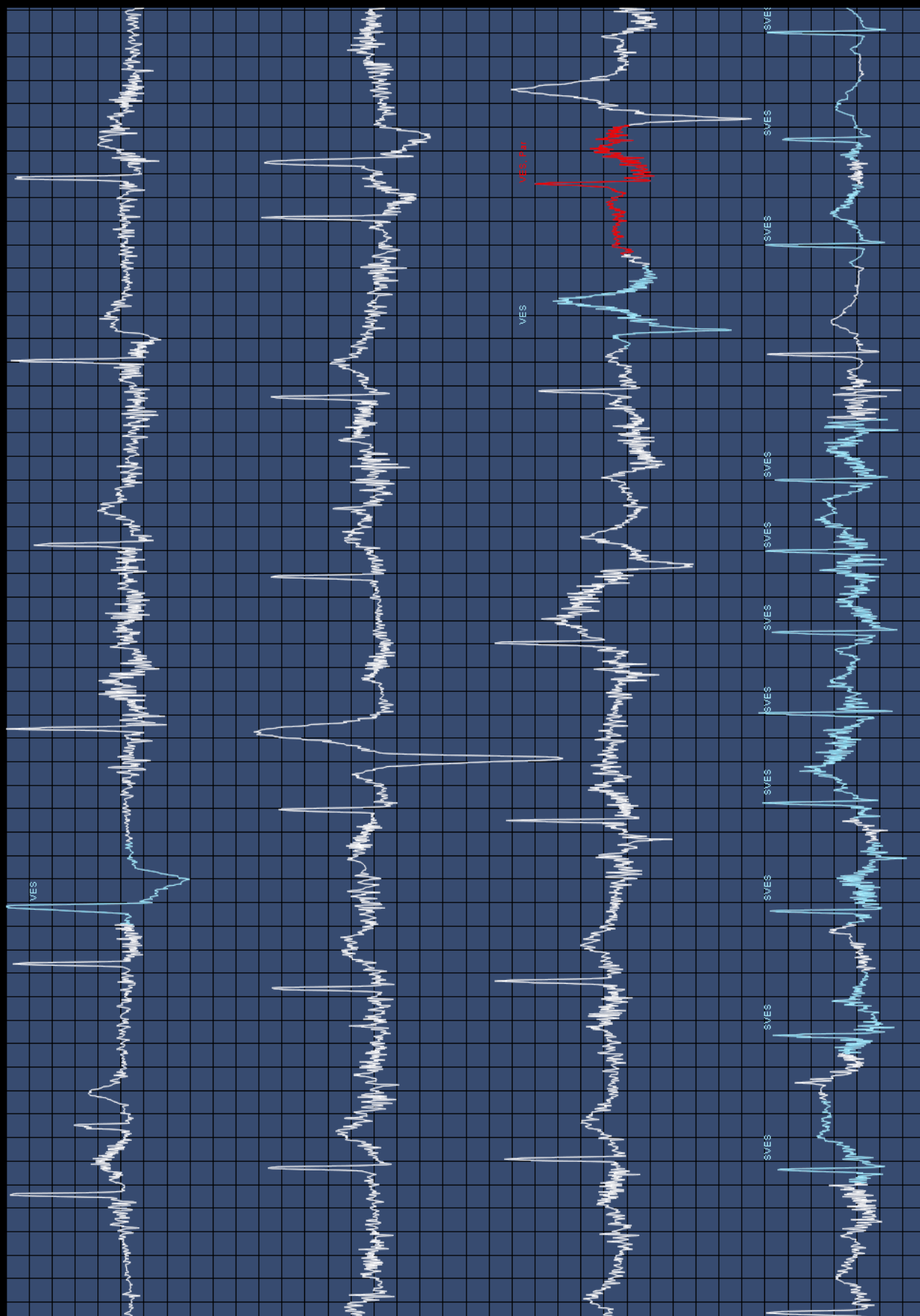

Supplement: Supplementary file 2 [file Data_Sheet_2.zip › EKG blindede/Subject 3 rest + max apnoea/3 max apnoea aVF no 2.pdf]

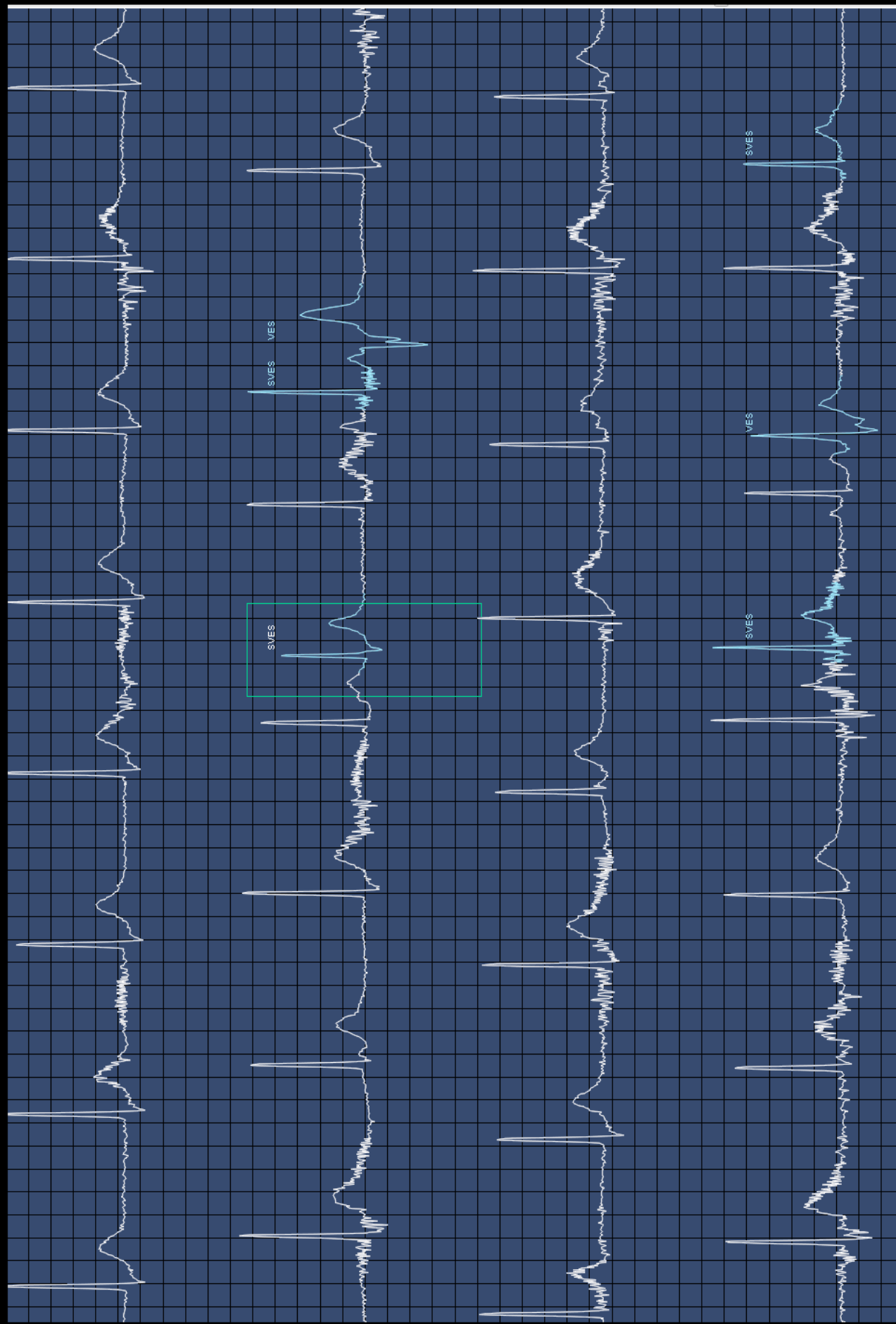

Supplement: Supplementary file 2 [file Data_Sheet_2.zip › EKG blindede/Subject 3 rest + max apnoea/3 max apnoea aVF.pdf]

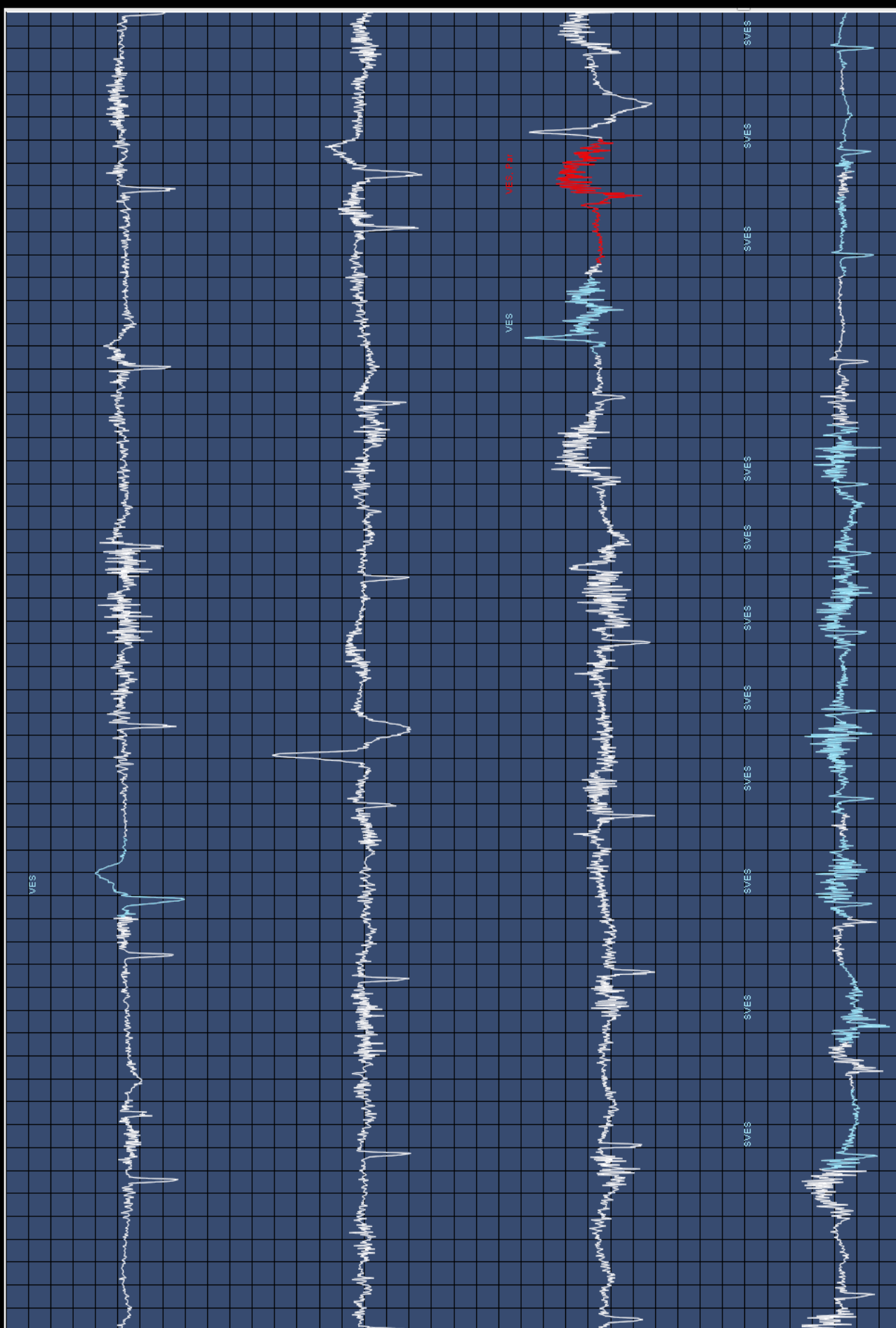

Supplement: Supplementary file 2 [file Data_Sheet_2.zip › EKG blindede/Subject 3 rest + max apnoea/3 max apnoea aVL no 2.pdf]

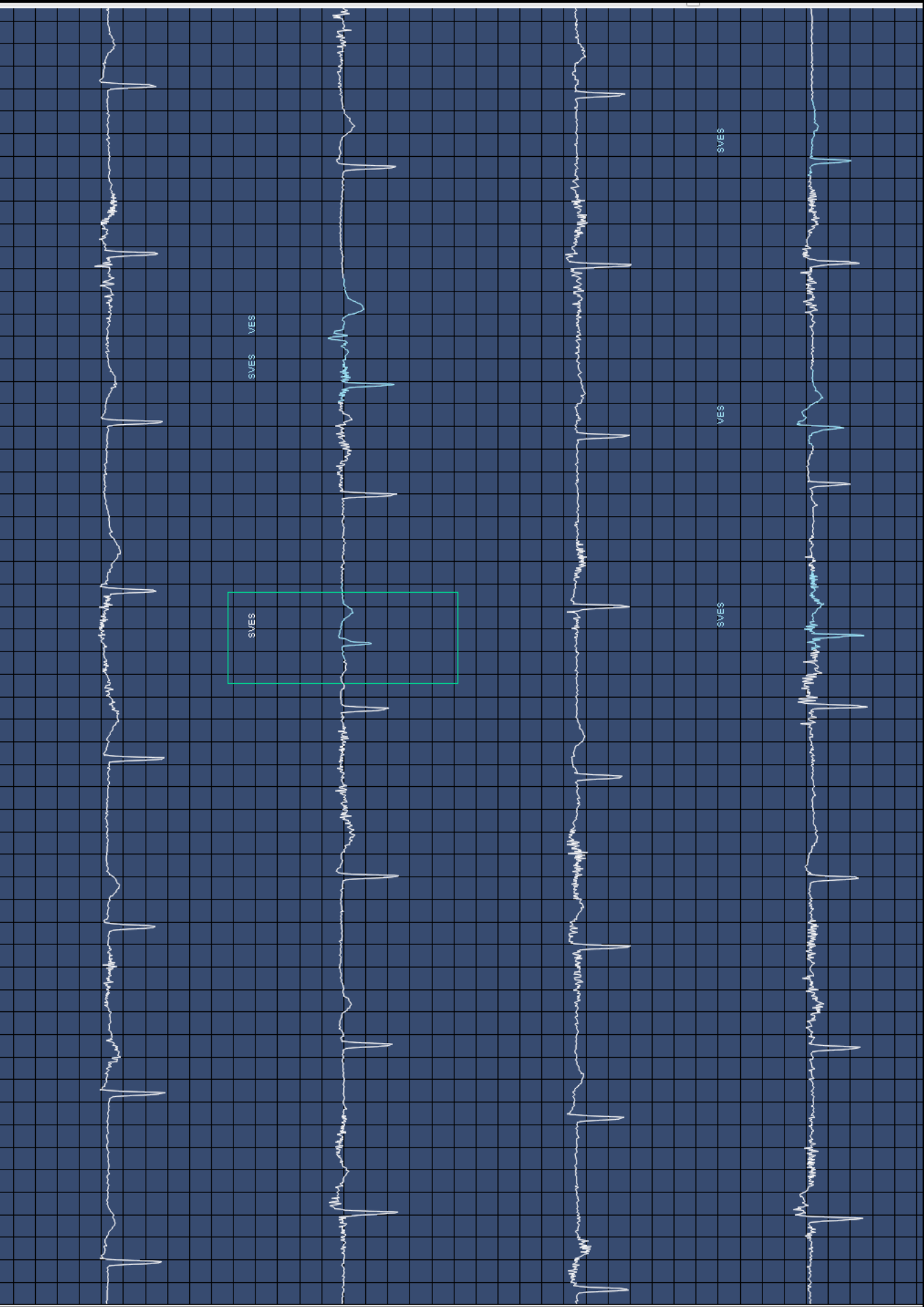

Supplement: Supplementary file 2 [file Data_Sheet_2.zip › EKG blindede/Subject 3 rest + max apnoea/3 max apnoea aVL.pdf]

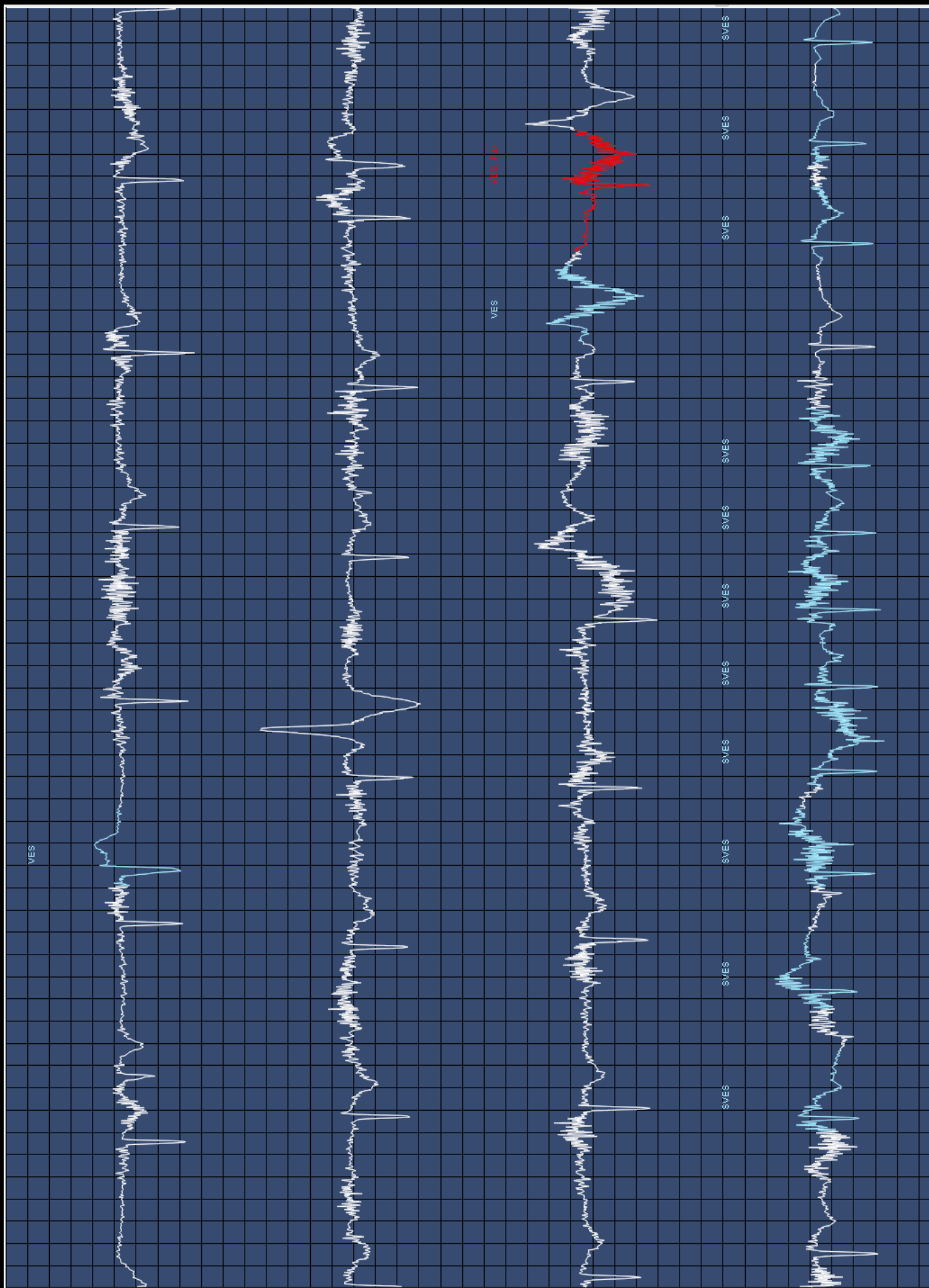

Supplement: Supplementary file 2 [file Data_Sheet_2.zip › EKG blindede/Subject 3 rest + max apnoea/3 max apnoea aVR no 2.pdf]

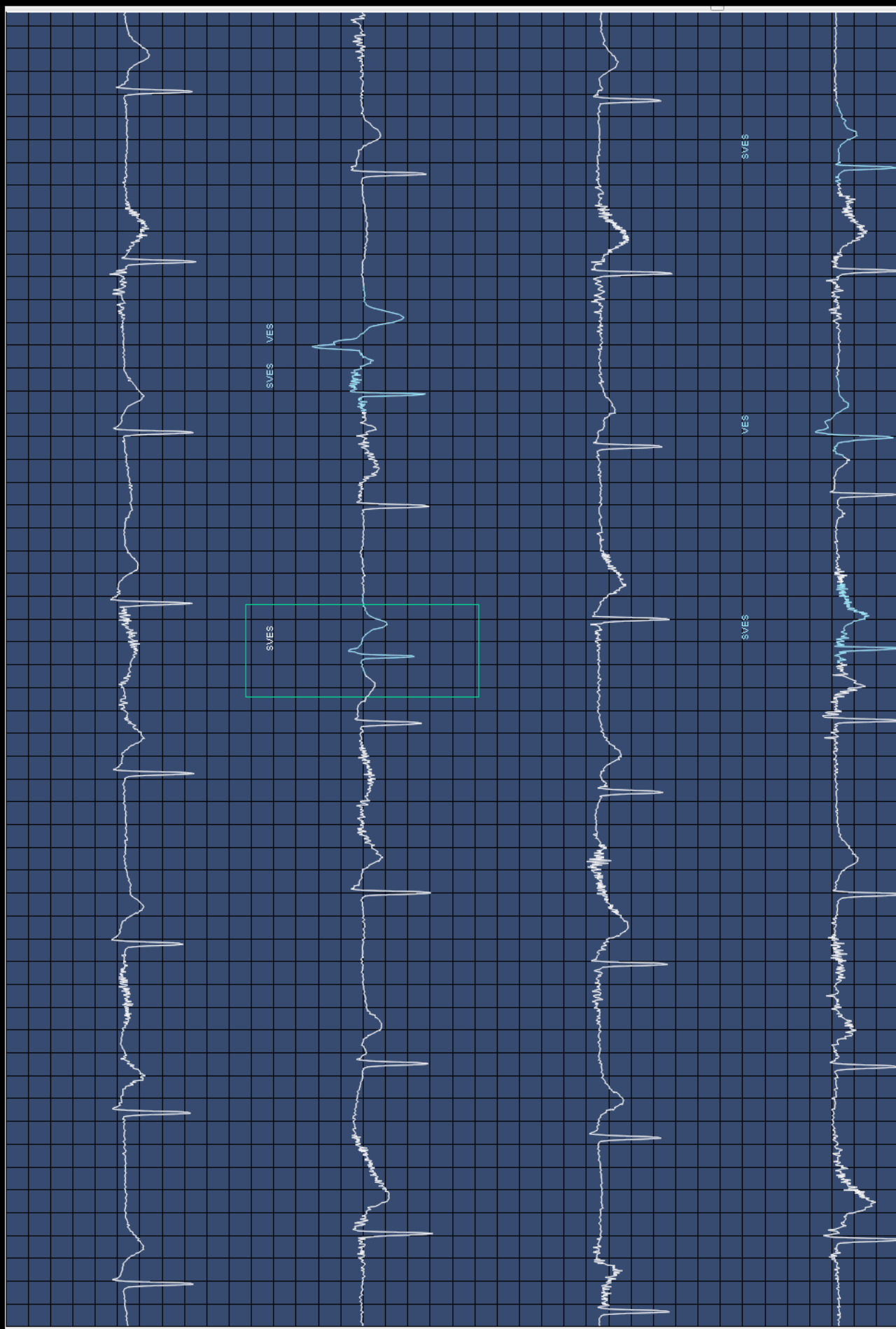

Supplement: Supplementary file 2 [file Data_Sheet_2.zip › EKG blindede/Subject 3 rest + max apnoea/3 max apnoea aVR.pdf]

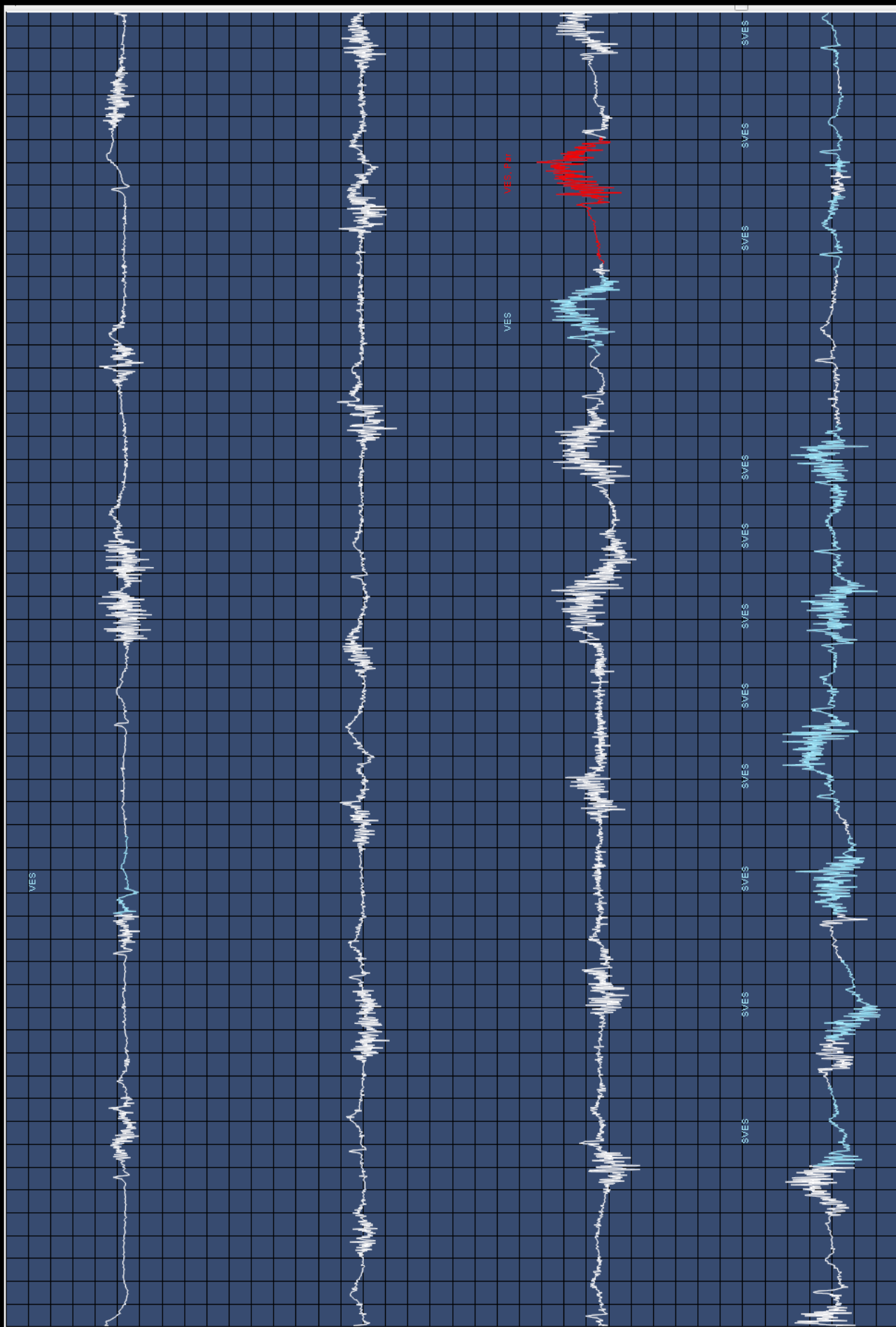

Supplement: Supplementary file 2 [file Data_Sheet_2.zip › EKG blindede/Subject 3 rest + max apnoea/3 max apnoea I no 2.pdf]

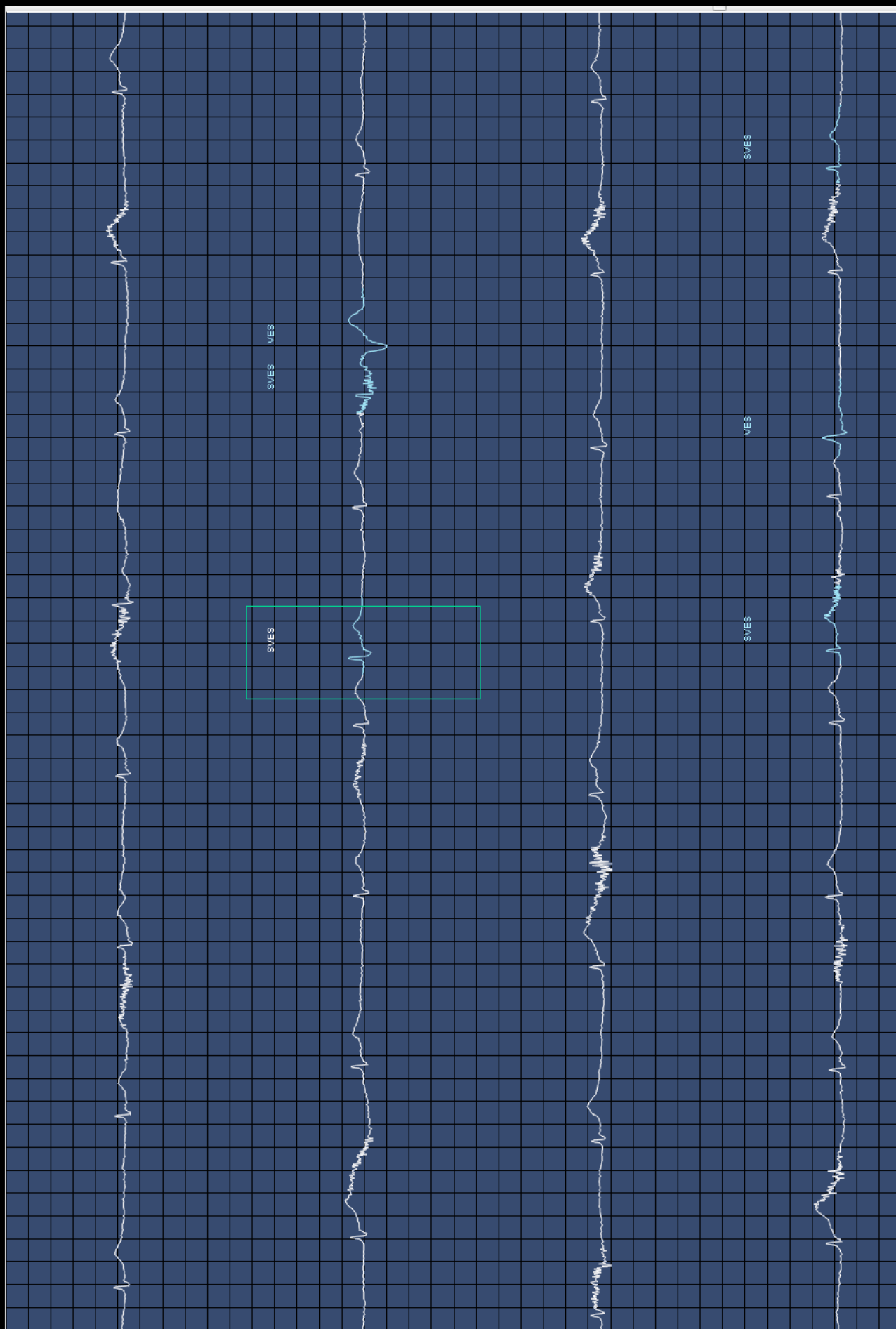

Supplement: Supplementary file 2 [file Data_Sheet_2.zip › EKG blindede/Subject 3 rest + max apnoea/3 max apnoea I.pdf]

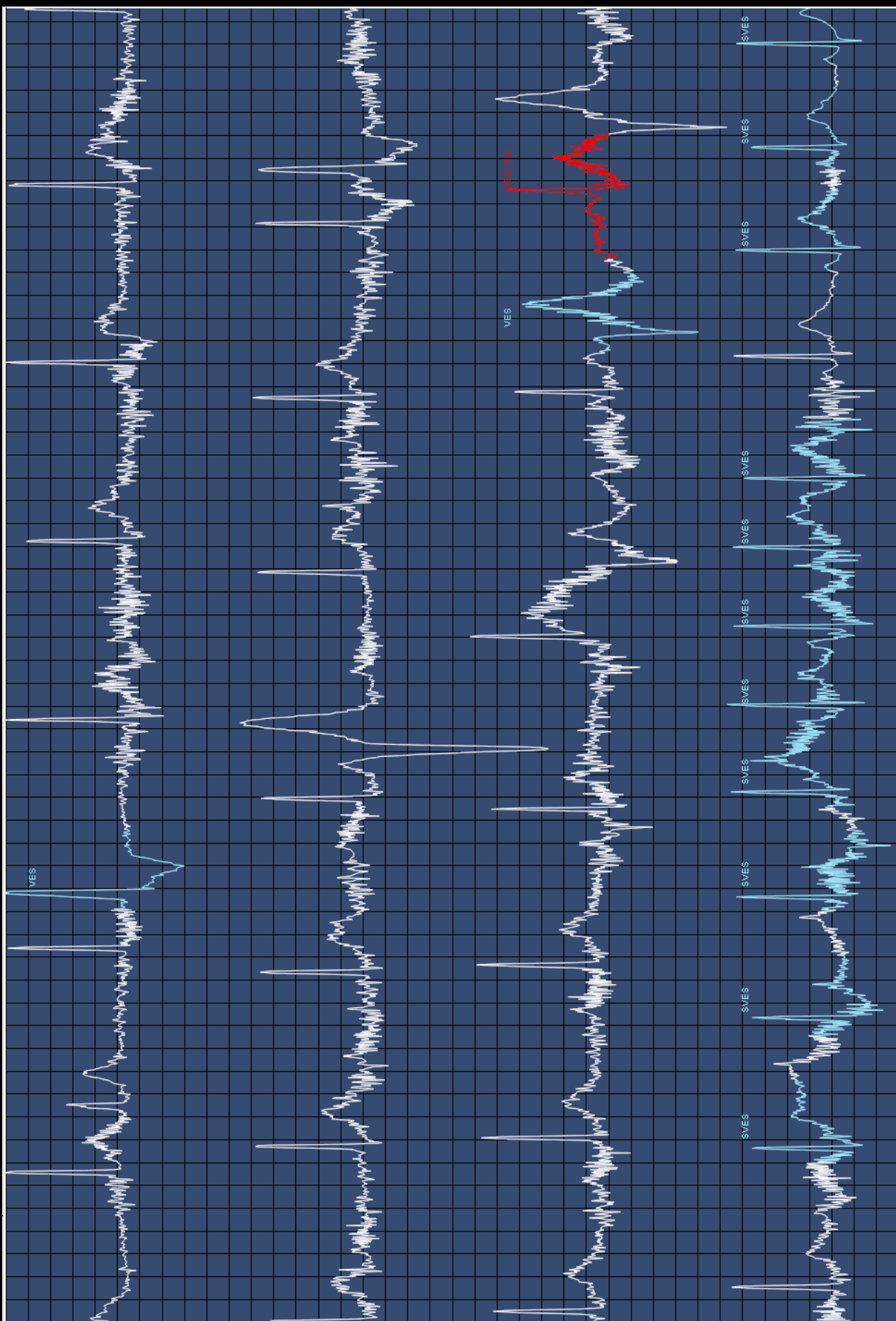

Supplement: Supplementary file 2 [file Data_Sheet_2.zip › EKG blindede/Subject 3 rest + max apnoea/3 max apnoea II no 2.pdf]

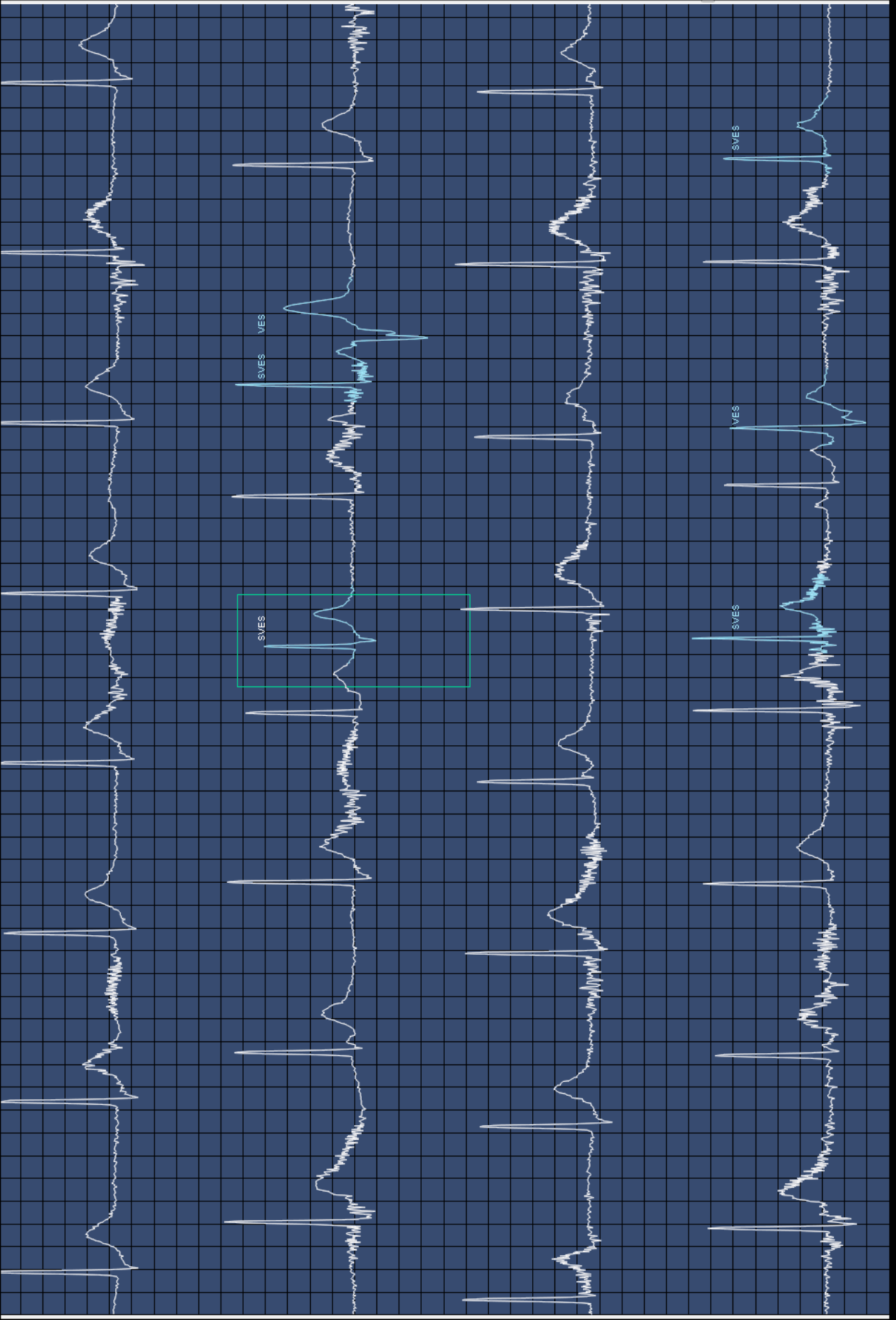

Supplement: Supplementary file 2 [file Data_Sheet_2.zip › EKG blindede/Subject 3 rest + max apnoea/3 max apnoea II.pdf]

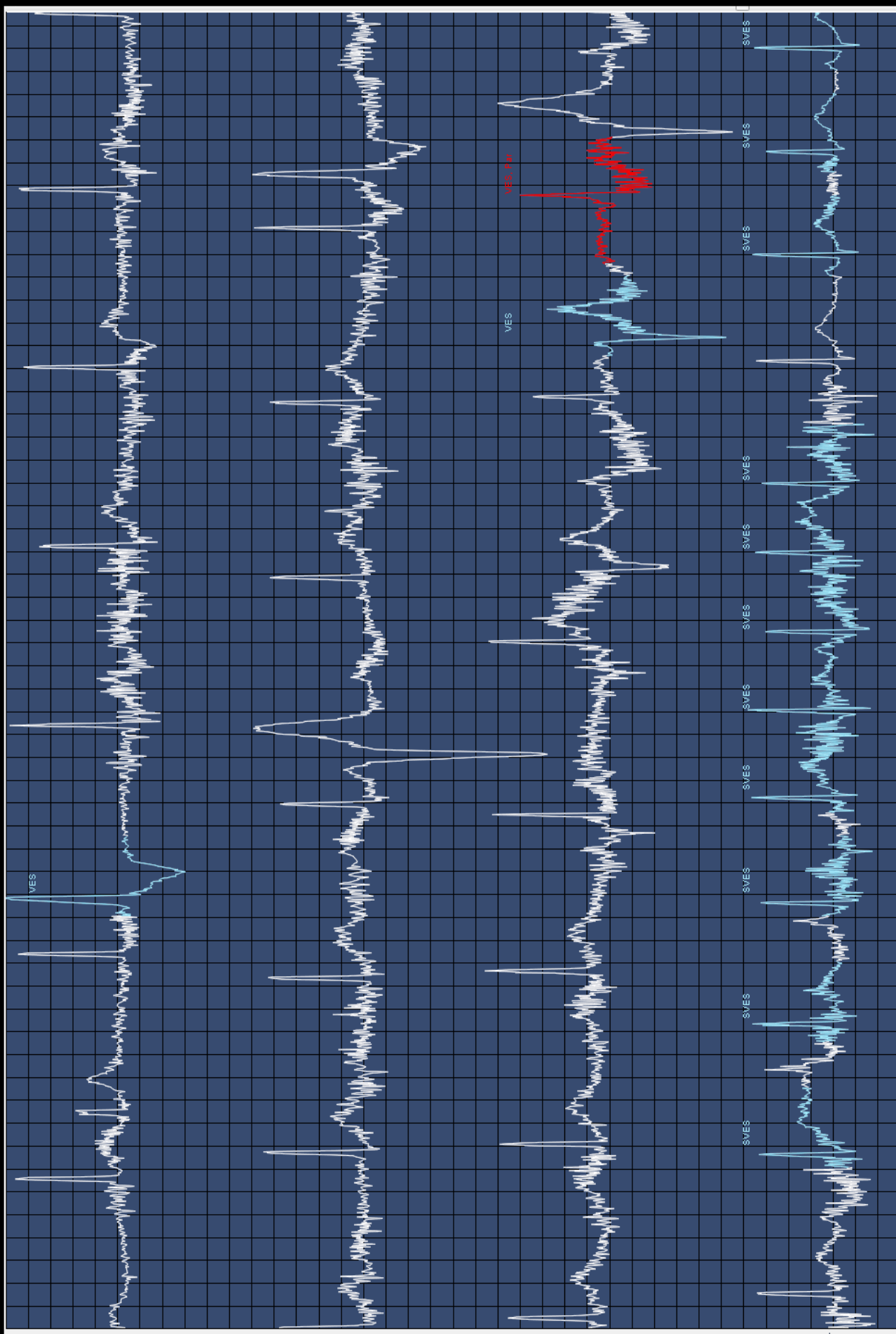

Supplement: Supplementary file 2 [file Data_Sheet_2.zip › EKG blindede/Subject 3 rest + max apnoea/3 max apnoea III no 2.pdf]

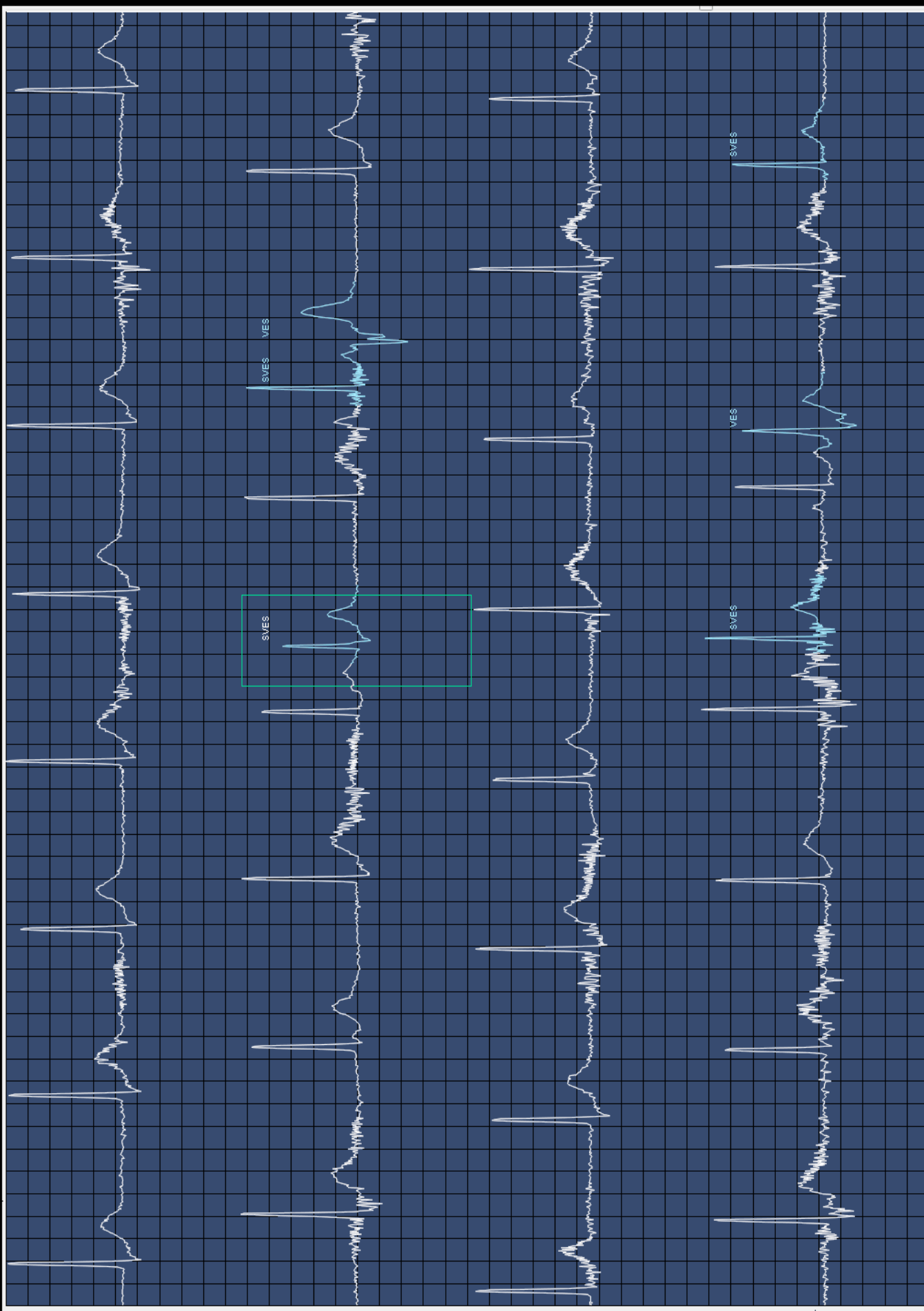

Supplement: Supplementary file 2 [file Data_Sheet_2.zip › EKG blindede/Subject 3 rest + max apnoea/3 max apnoea III.pdf]

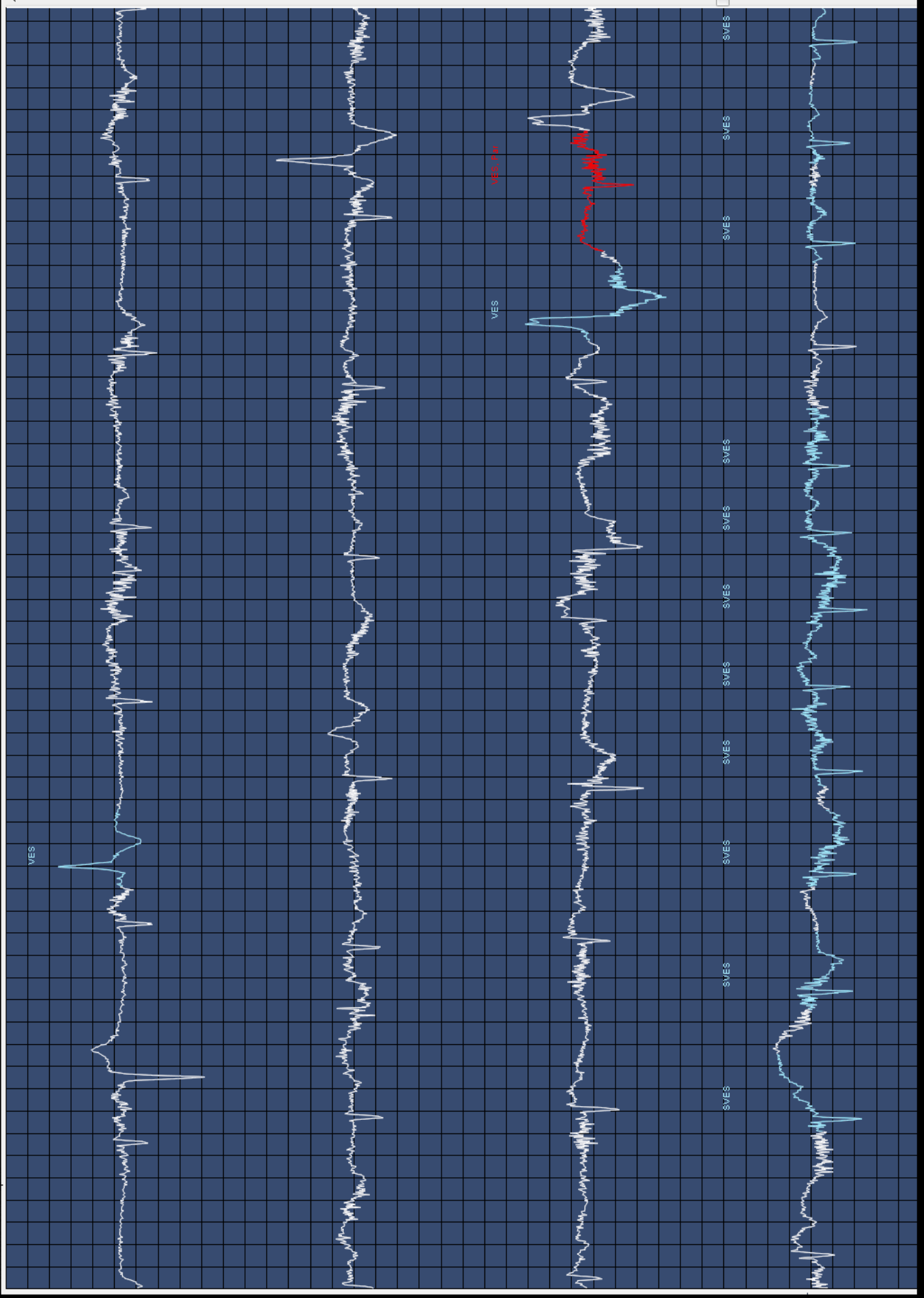

Supplement: Supplementary file 2 [file Data_Sheet_2.zip › EKG blindede/Subject 3 rest + max apnoea/3 max apnoea V1 no 2.pdf]

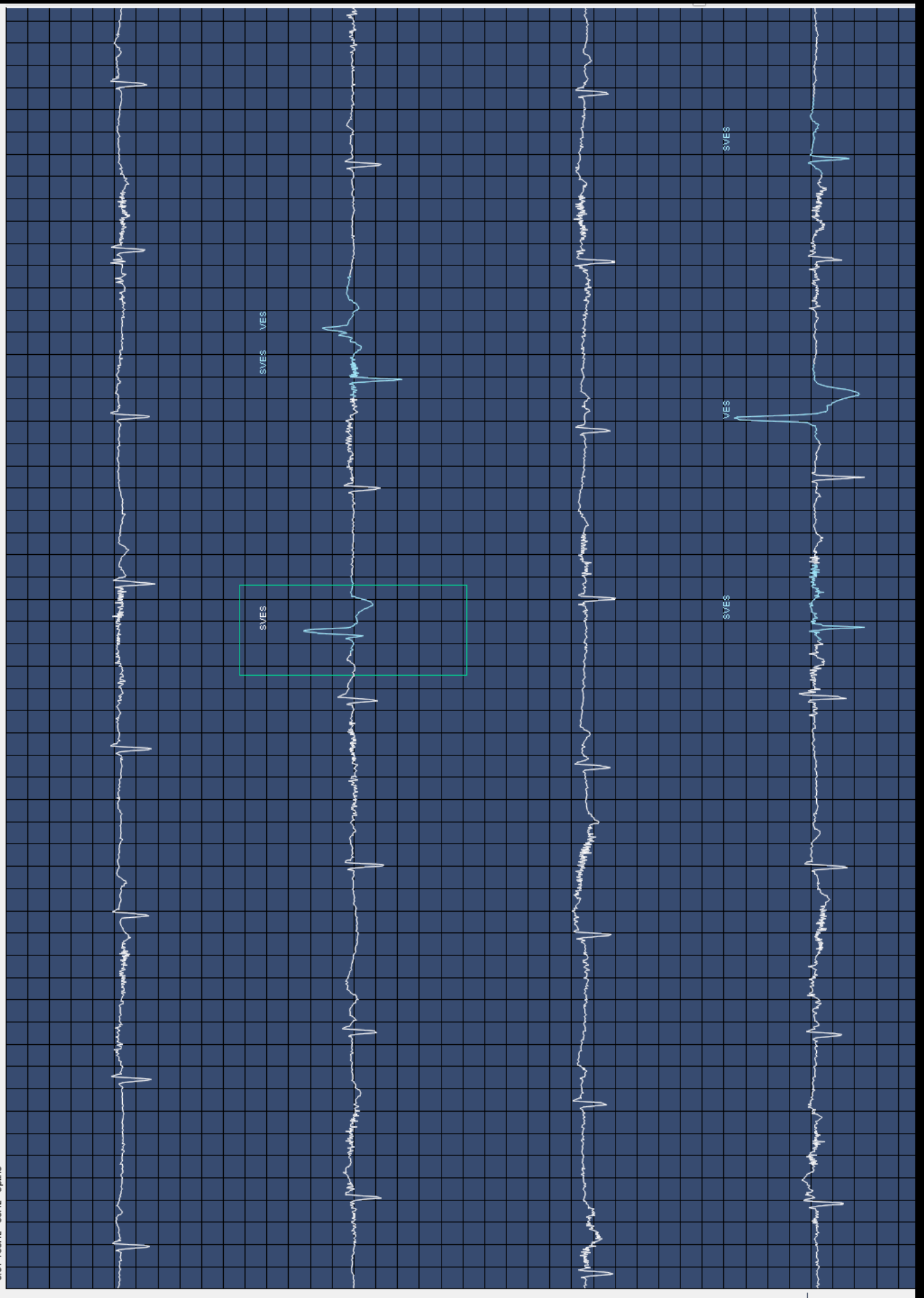

Supplement: Supplementary file 2 [file Data_Sheet_2.zip › EKG blindede/Subject 3 rest + max apnoea/3 max apnoea V1.pdf]

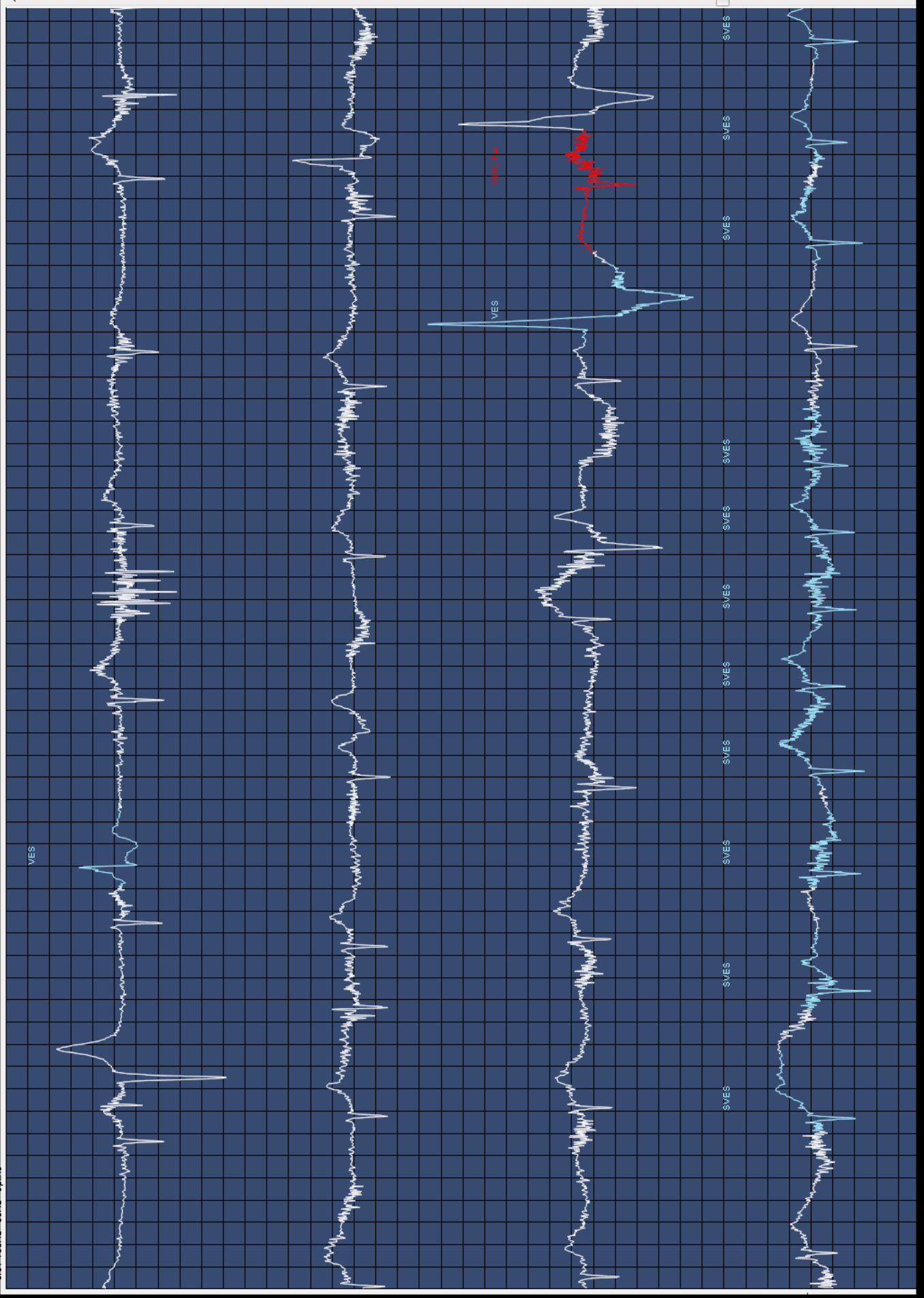

Supplement: Supplementary file 2 [file Data_Sheet_2.zip › EKG blindede/Subject 3 rest + max apnoea/3 max apnoea V2 no 2.pdf]

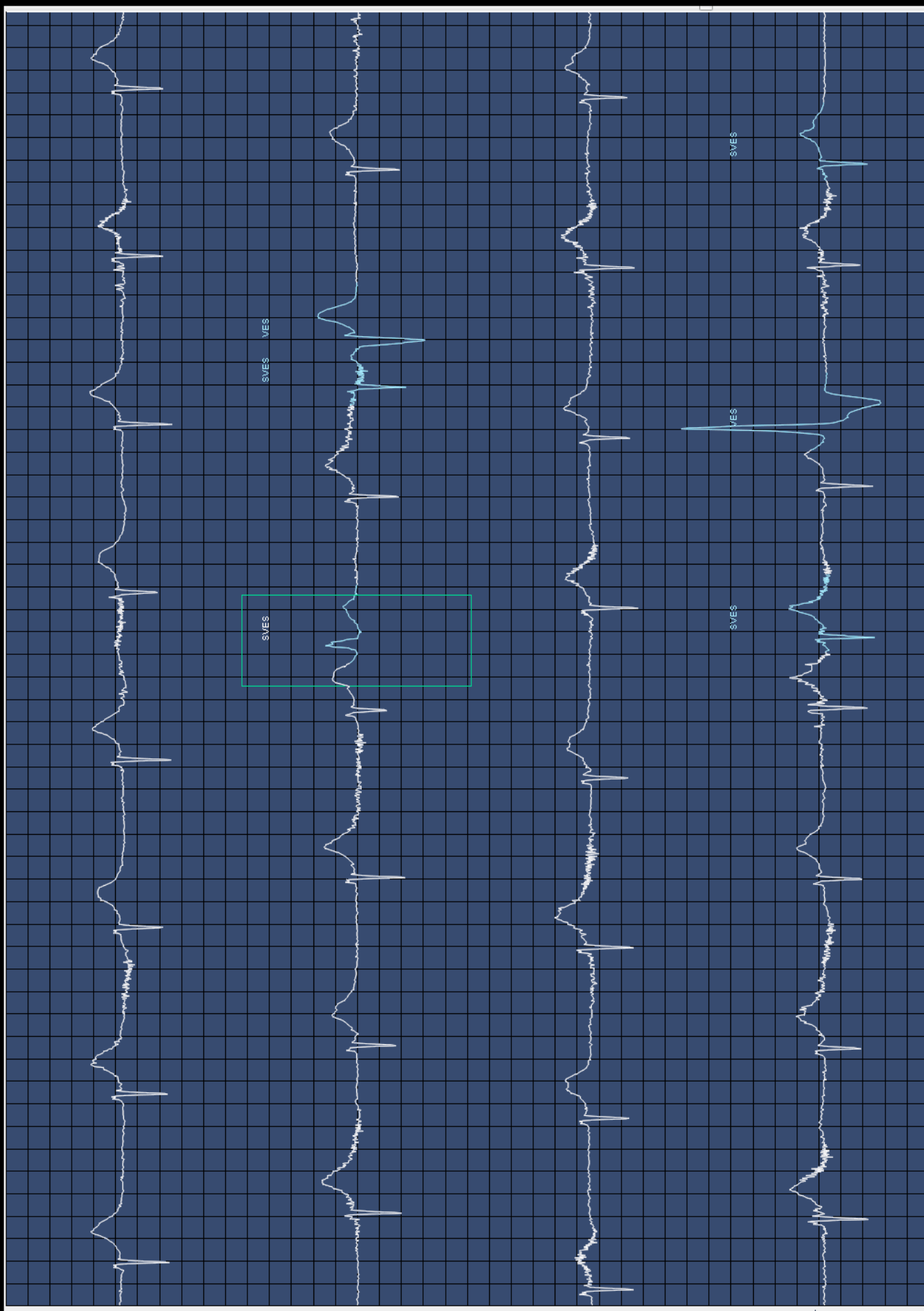

Supplement: Supplementary file 2 [file Data_Sheet_2.zip › EKG blindede/Subject 3 rest + max apnoea/3 max apnoea V2.pdf]

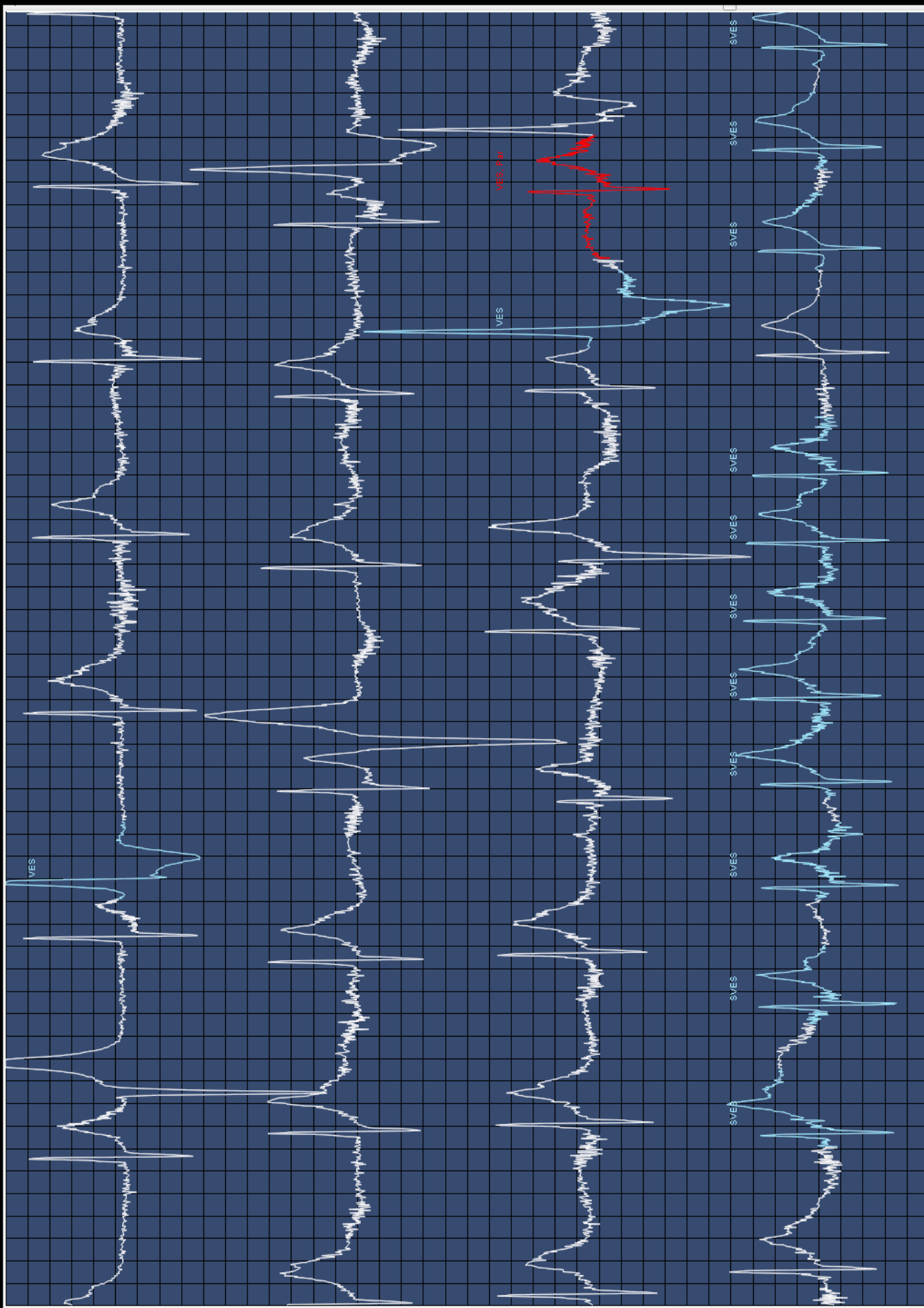

Supplement: Supplementary file 2 [file Data_Sheet_2.zip › EKG blindede/Subject 3 rest + max apnoea/3 max apnoea V3 no 2.pdf]

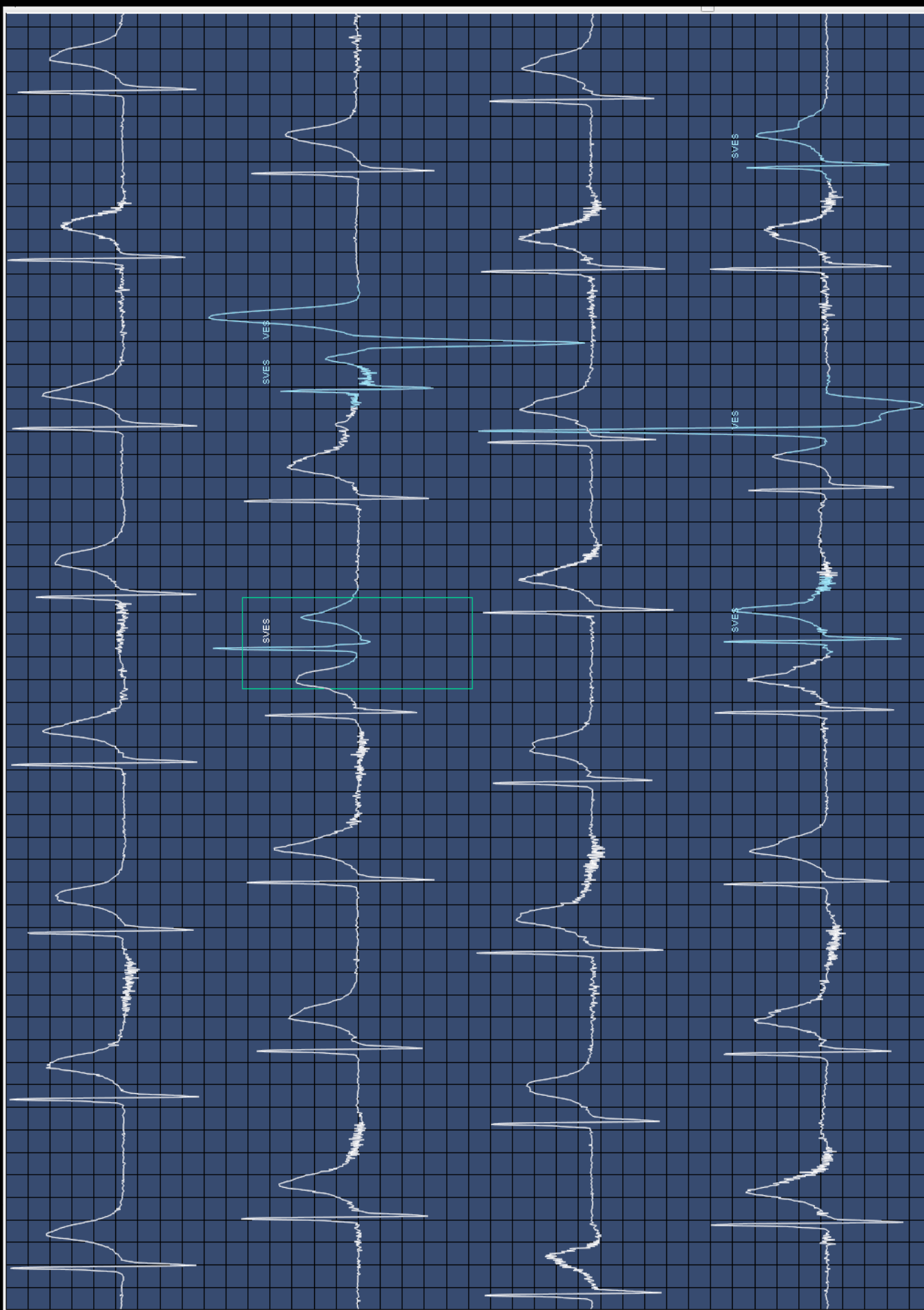

Supplement: Supplementary file 2 [file Data_Sheet_2.zip › EKG blindede/Subject 3 rest + max apnoea/3 max apnoea V3.pdf]

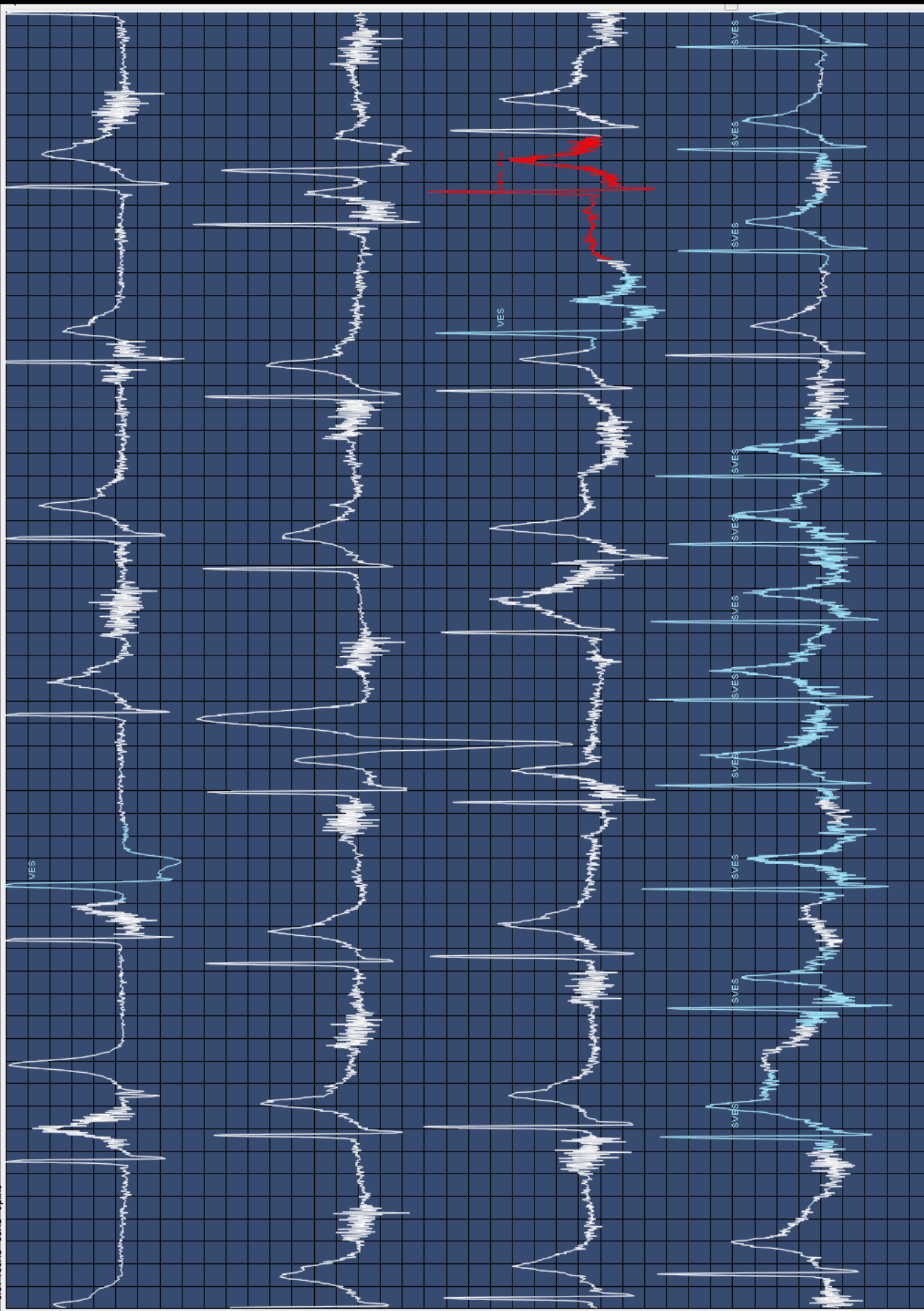

Supplement: Supplementary file 2 [file Data_Sheet_2.zip › EKG blindede/Subject 3 rest + max apnoea/3 max apnoea V4 no 2.pdf]

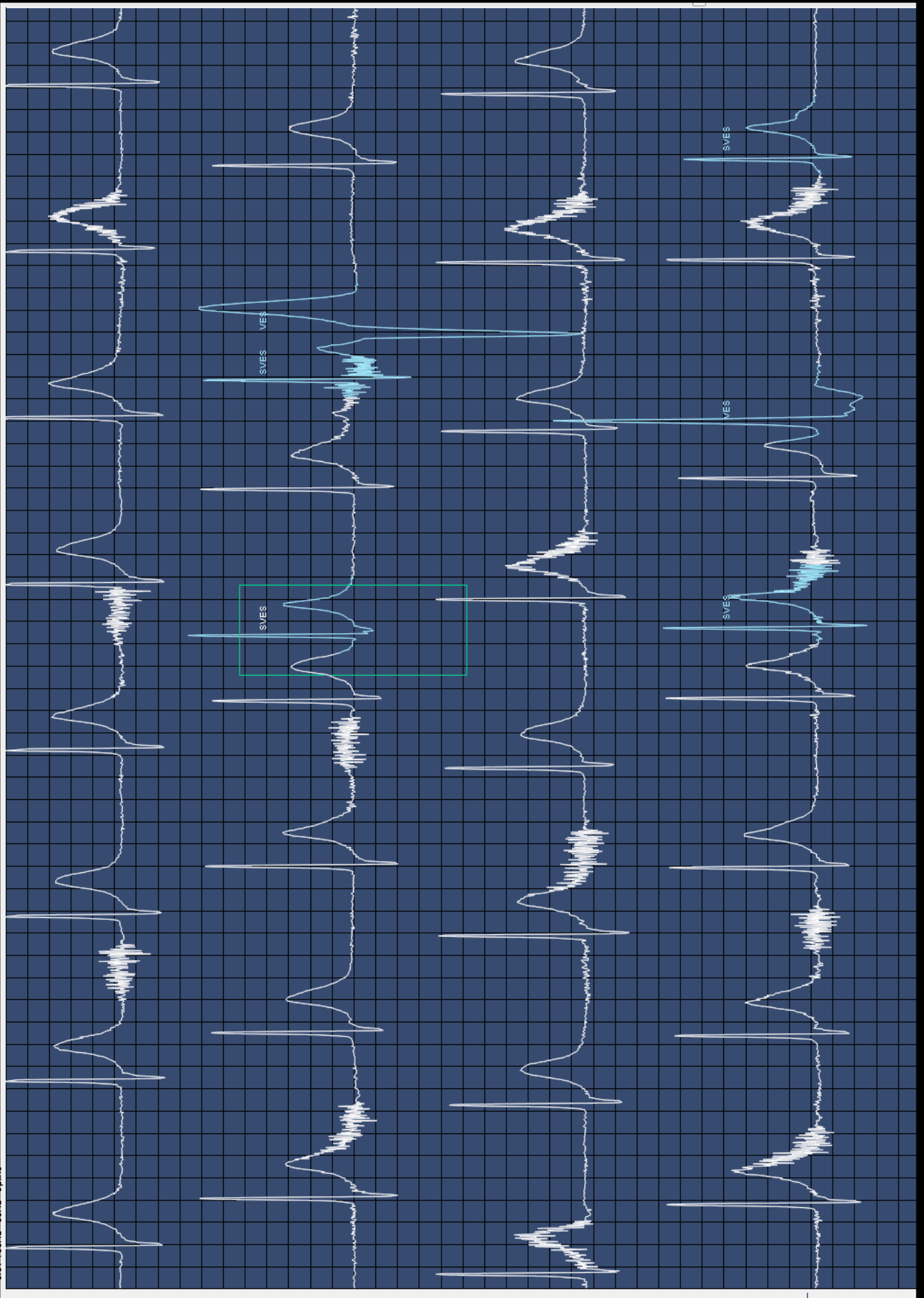

Supplement: Supplementary file 2 [file Data_Sheet_2.zip › EKG blindede/Subject 3 rest + max apnoea/3 max apnoea V4.pdf]

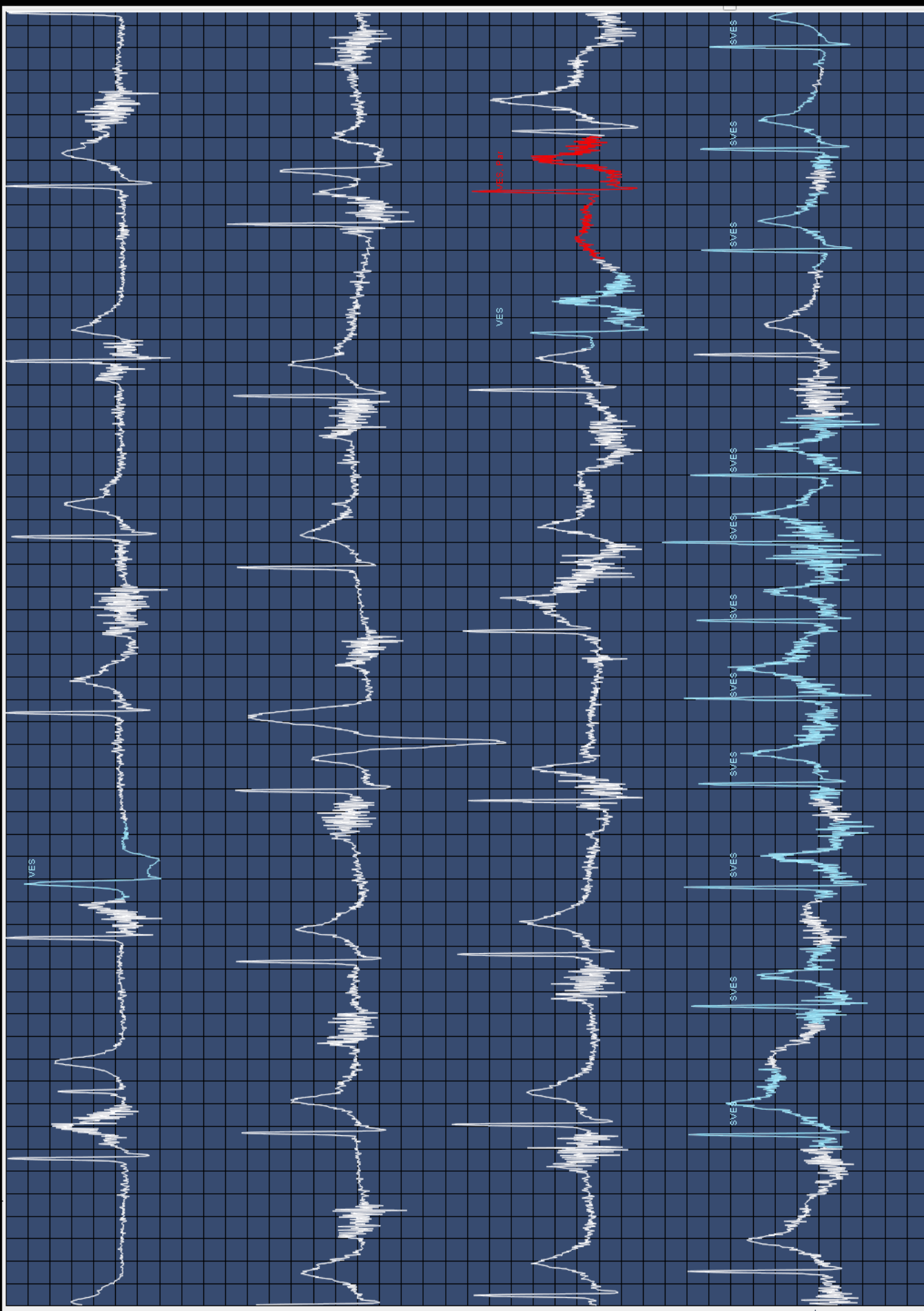

Supplement: Supplementary file 2 [file Data_Sheet_2.zip › EKG blindede/Subject 3 rest + max apnoea/3 max apnoea V5 no 2.pdf]

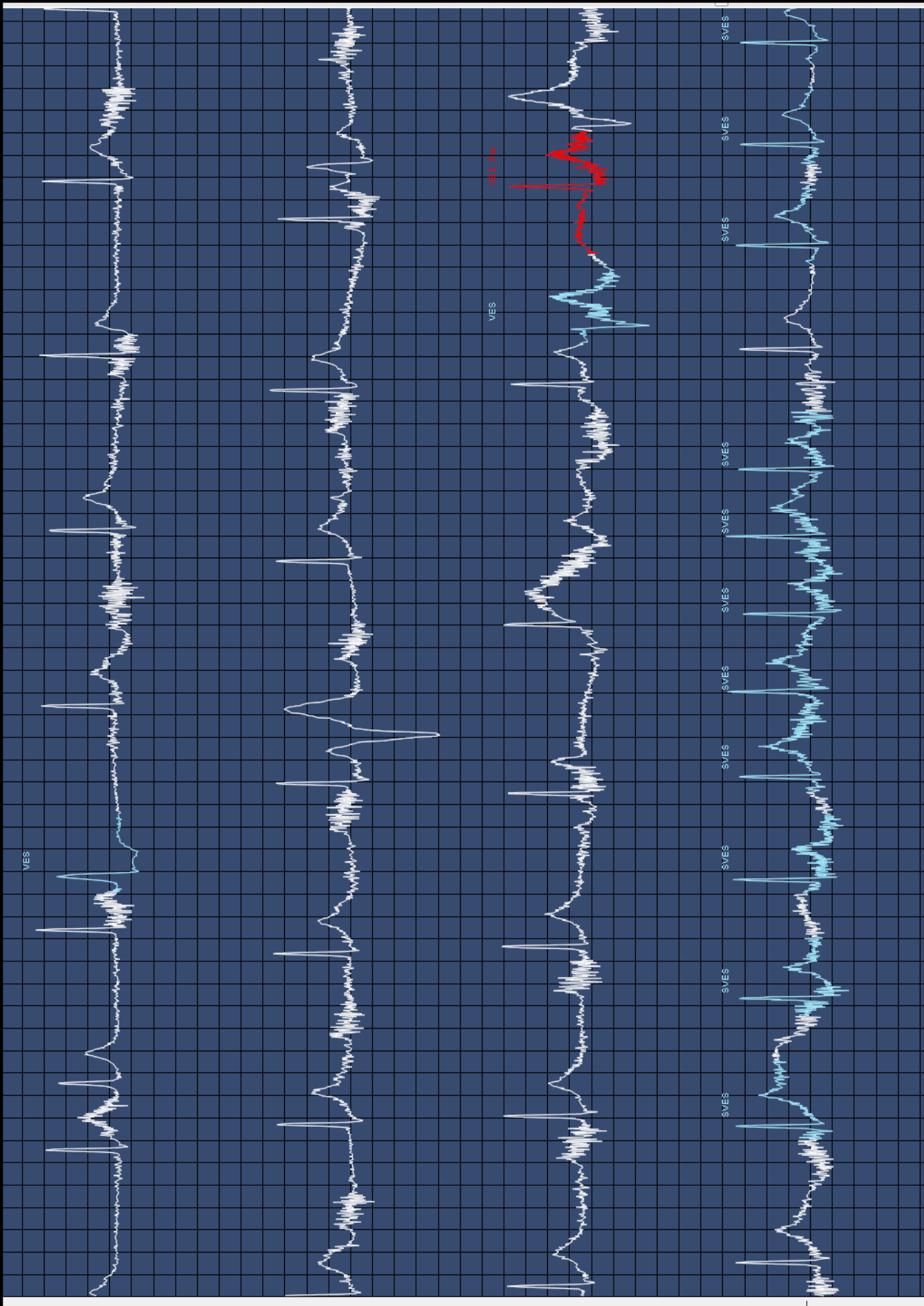

Supplement: Supplementary file 2 [file Data_Sheet_2.zip › EKG blindede/Subject 3 rest + max apnoea/3 max apnoea V6 no 2.pdf]

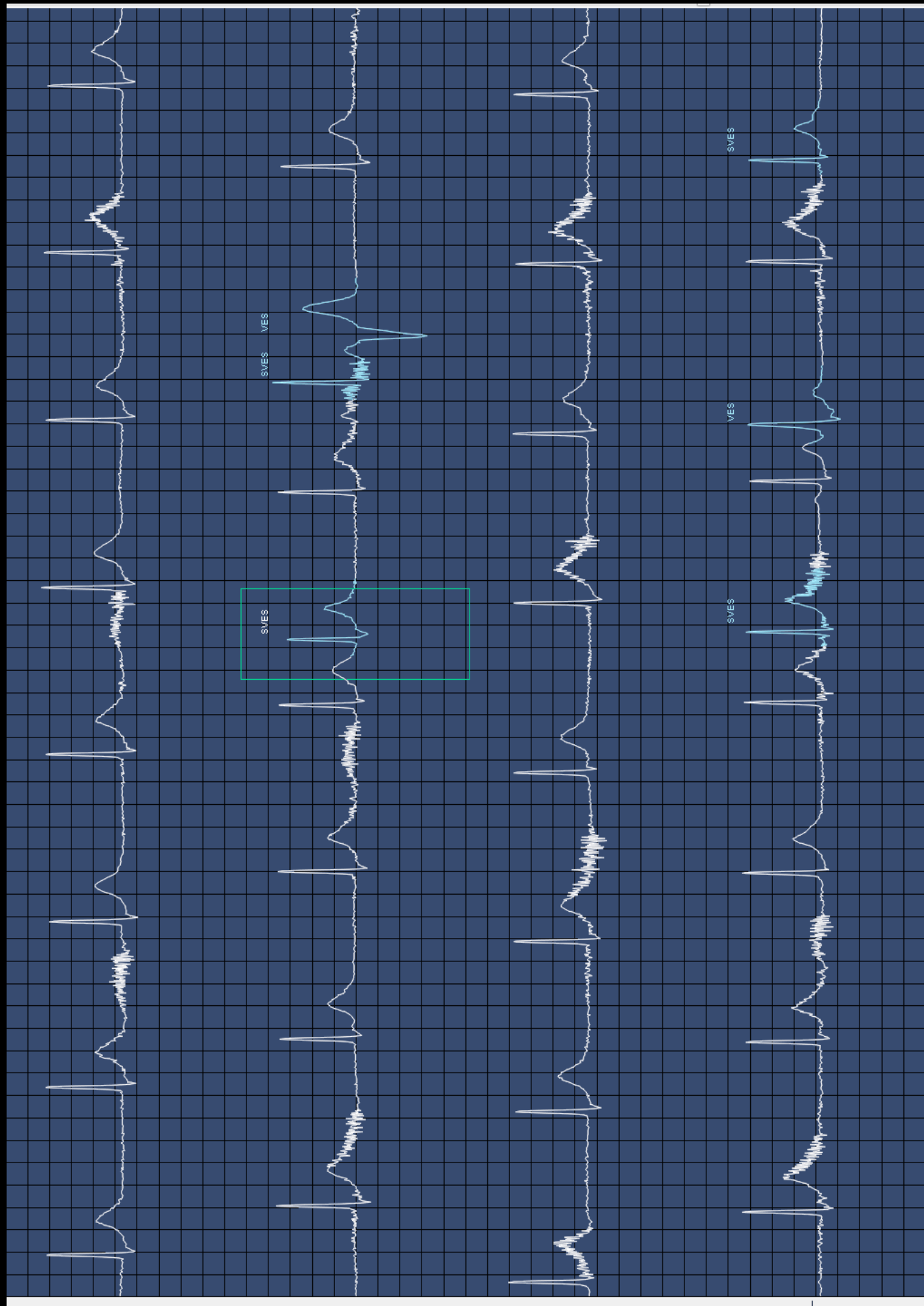

Supplement: Supplementary file 2 [file Data_Sheet_2.zip › EKG blindede/Subject 3 rest + max apnoea/3 max apnoea V6.pdf]

Testoversigt Full-disclosure EKG

aVR L 00:09 25mm/s 20mm/mV 4 Linjer Aritmi i farver

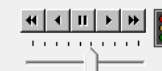

0.01-150Hz 50Hz Spline

aVR

29:11

29:23

29:34

29:46

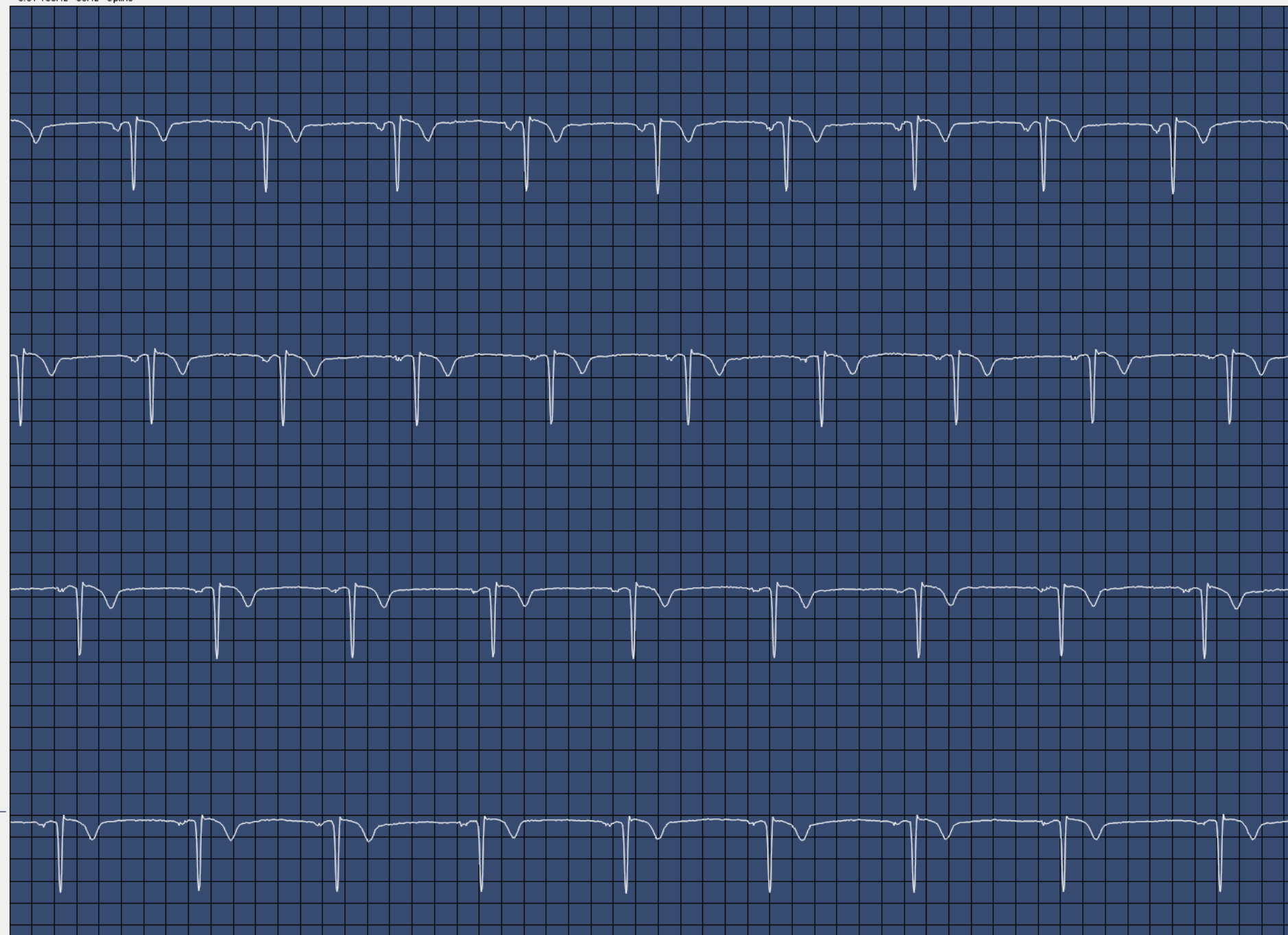

Supplement: Supplementary file 2 [file Data_Sheet_2.zip › EKG blindede/Subject 3 rest + max apnoea/3 rest aVR.pdf]

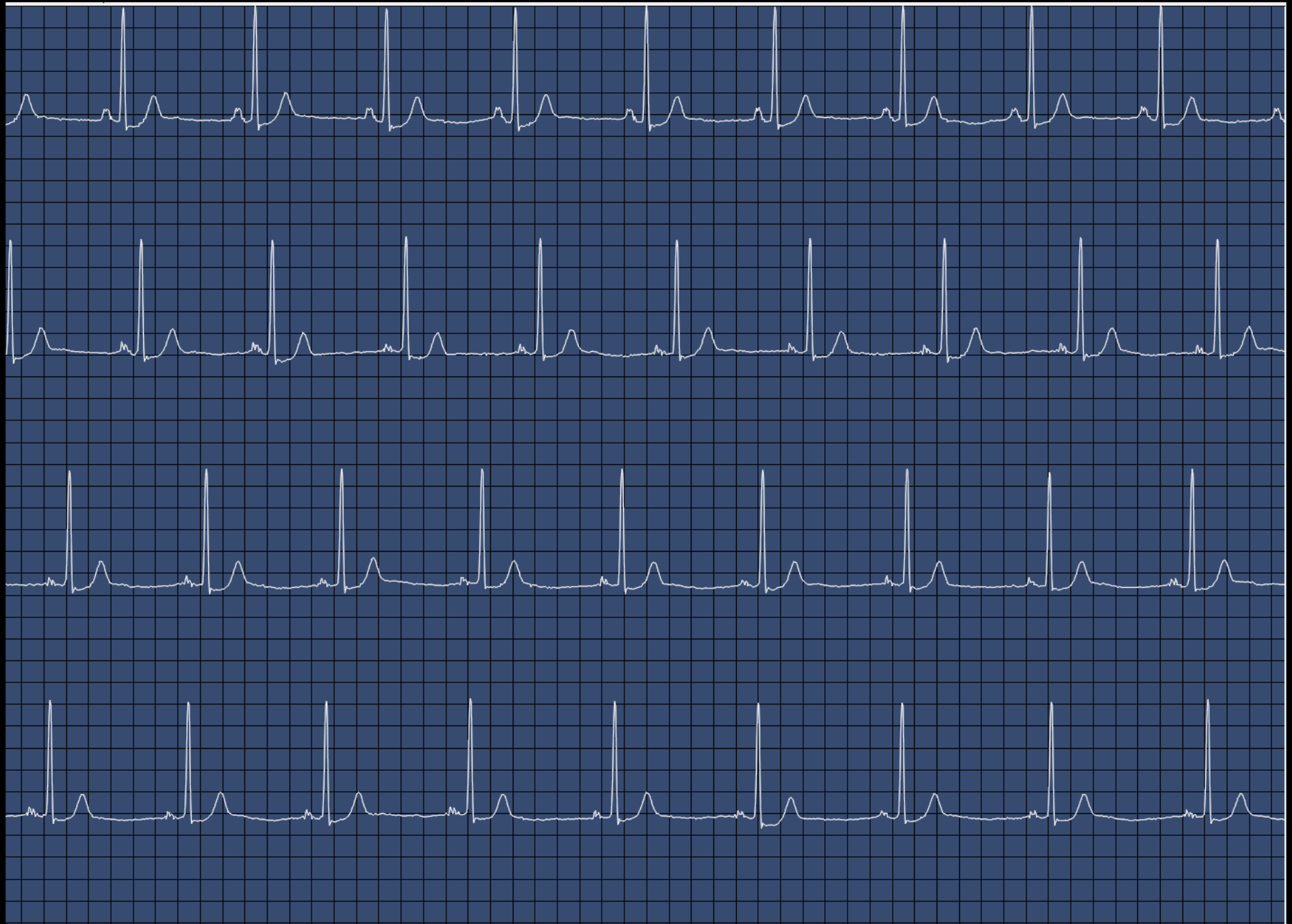

Supplement: Supplementary file 2 [file Data_Sheet_2.zip › EKG blindede/Subject 3 rest + max apnoea/3 rest III.pdf]

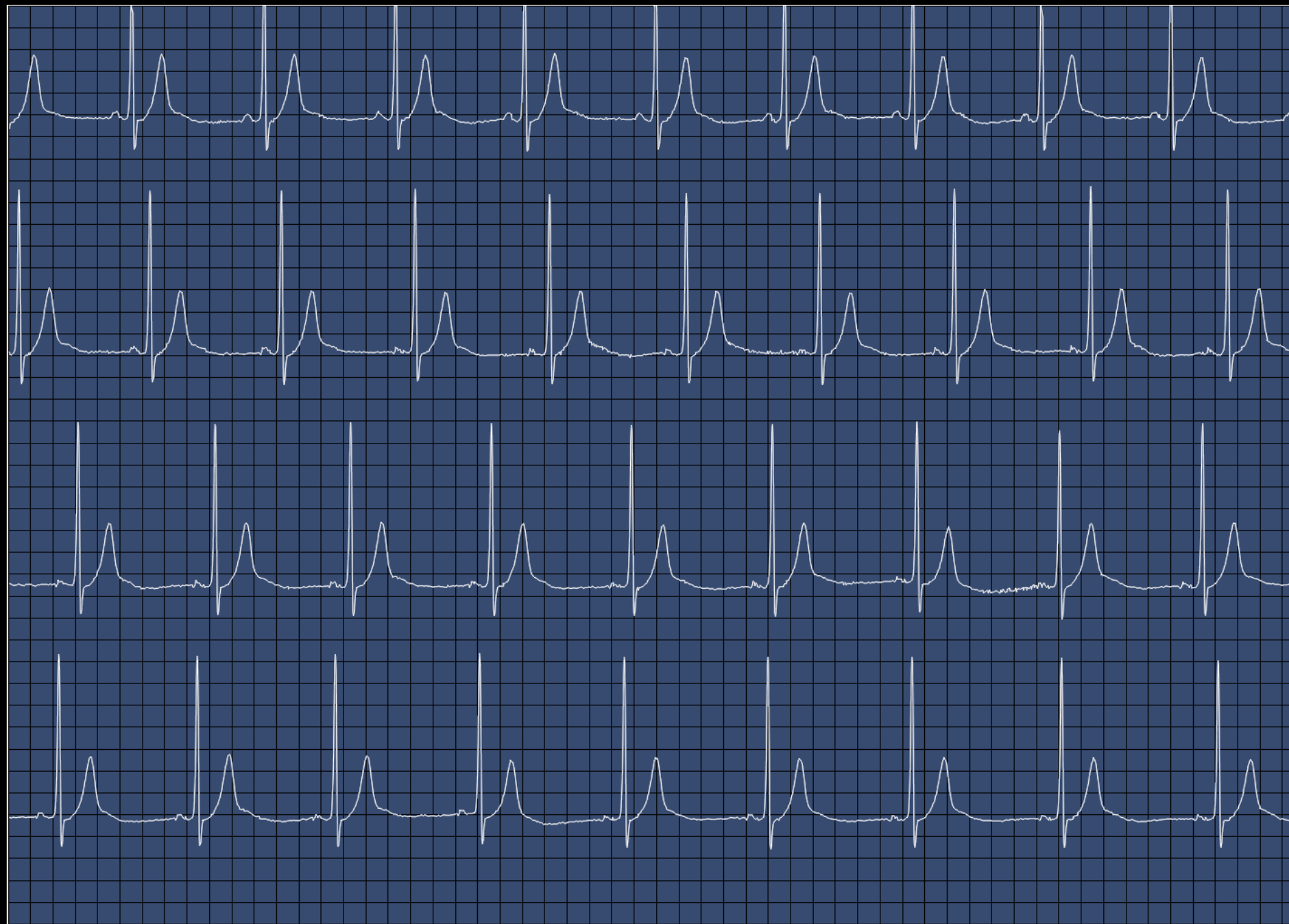

Supplement: Supplementary file 2 [file Data_Sheet_2.zip › EKG blindede/Subject 3 rest + max apnoea/3 rest V4.pdf]

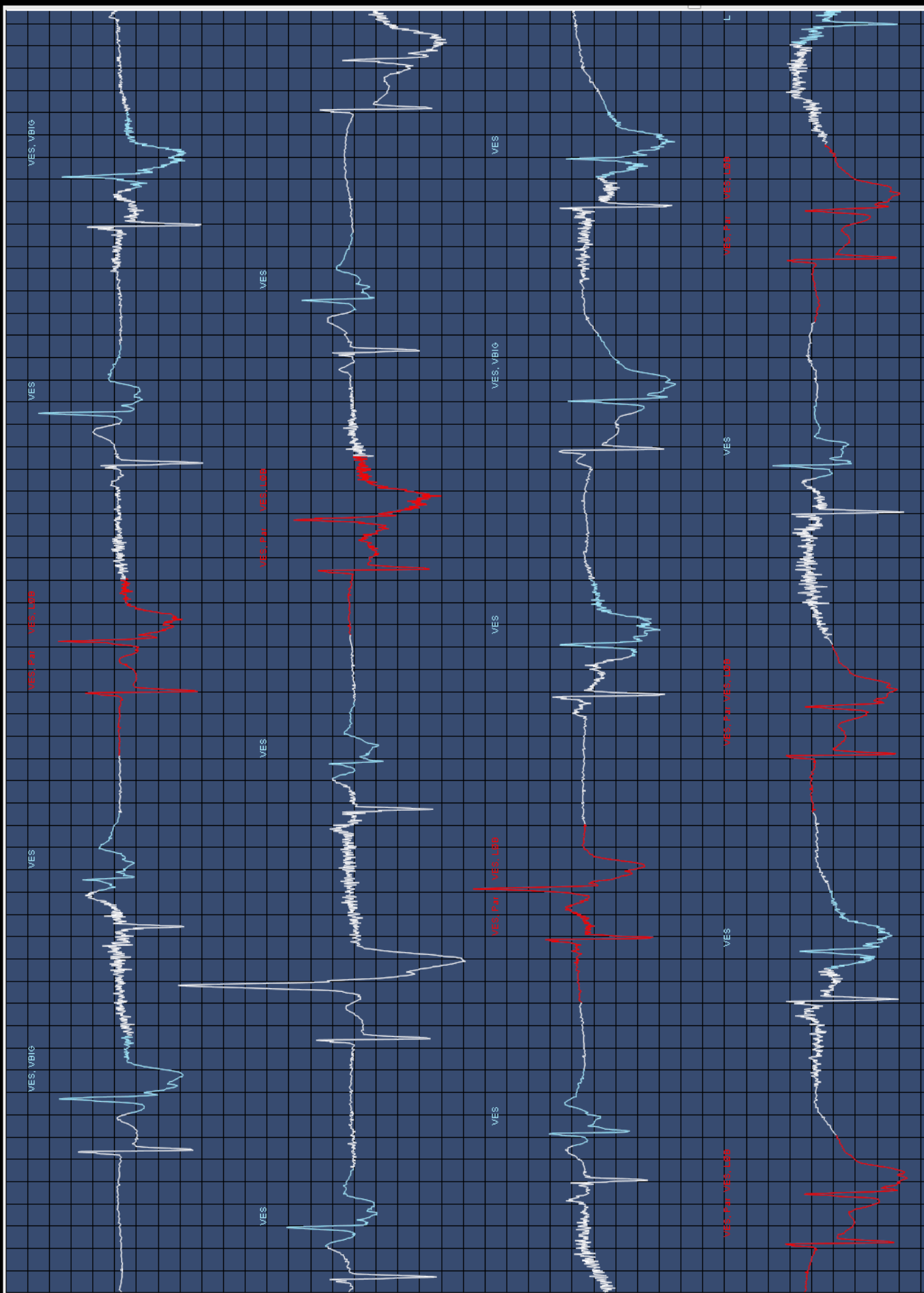

Supplement: Supplementary file 2 [file Data_Sheet_2.zip › EKG blindede/Subject 4 rest + max apnoea/4 max apnoea aVF no 2.pdf]

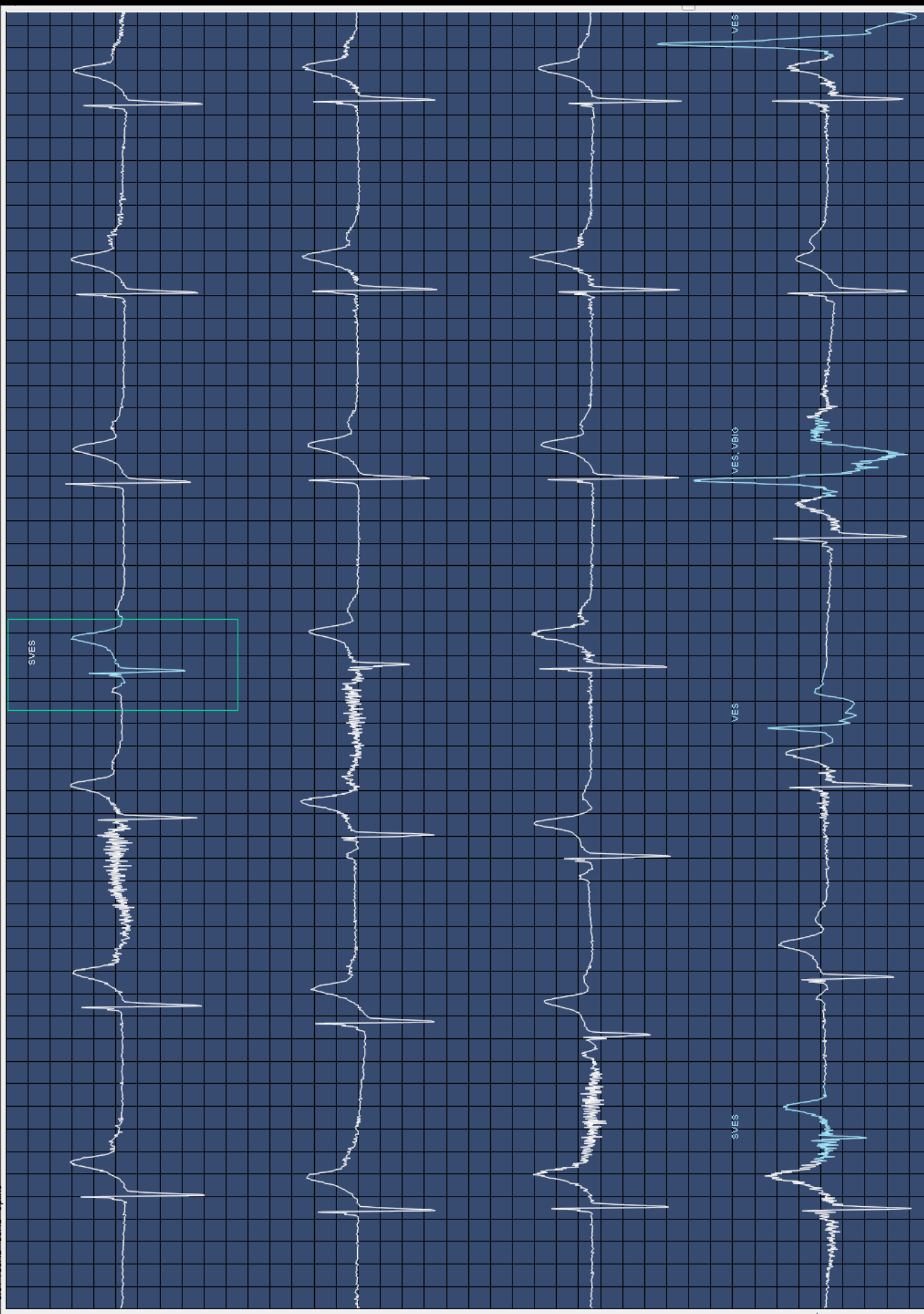

Supplement: Supplementary file 2 [file Data_Sheet_2.zip › EKG blindede/Subject 4 rest + max apnoea/4 max apnoea aVF.pdf]

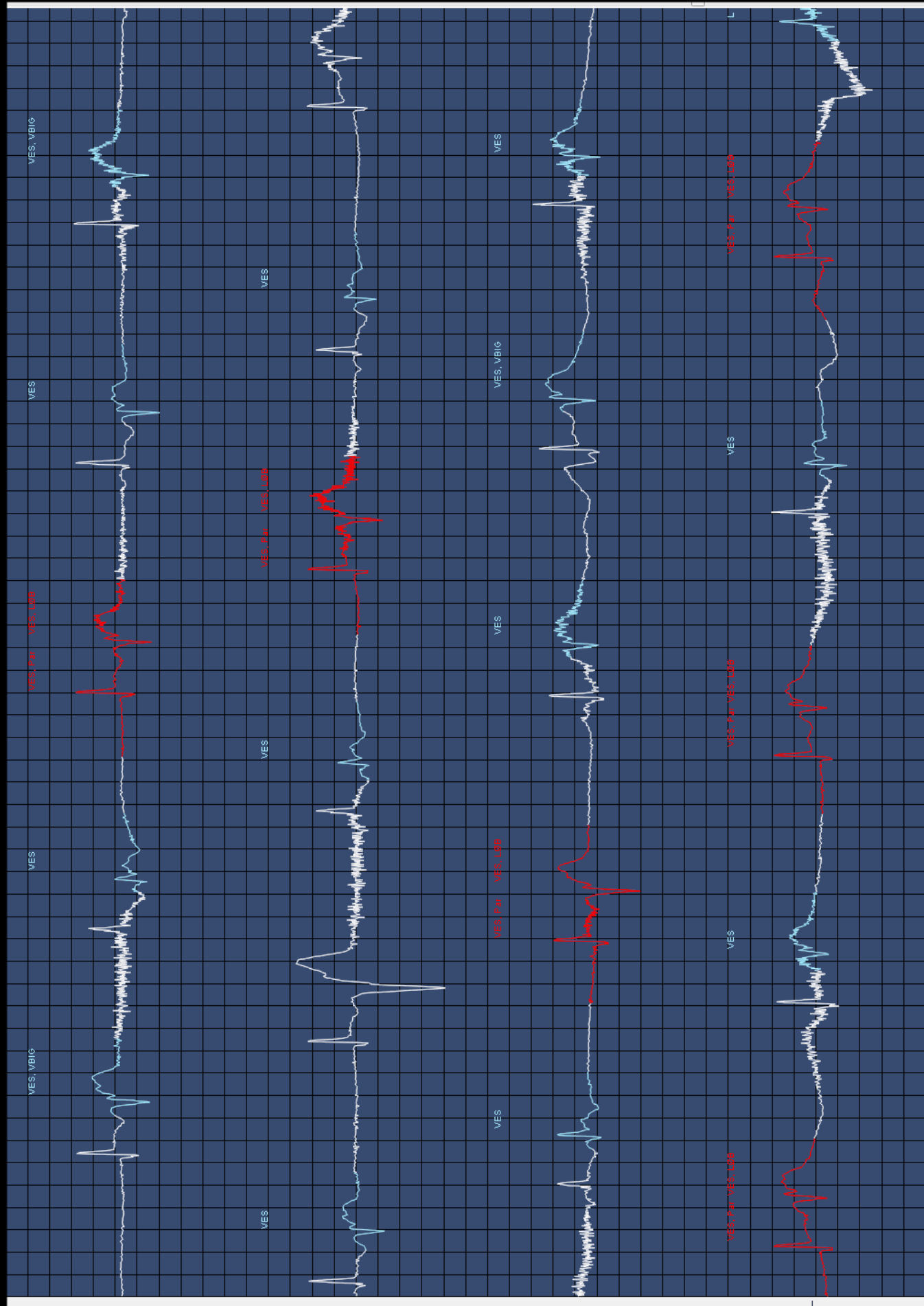

Supplement: Supplementary file 2 [file Data_Sheet_2.zip › EKG blindede/Subject 4 rest + max apnoea/4 max apnoea aVL no 2.pdf]

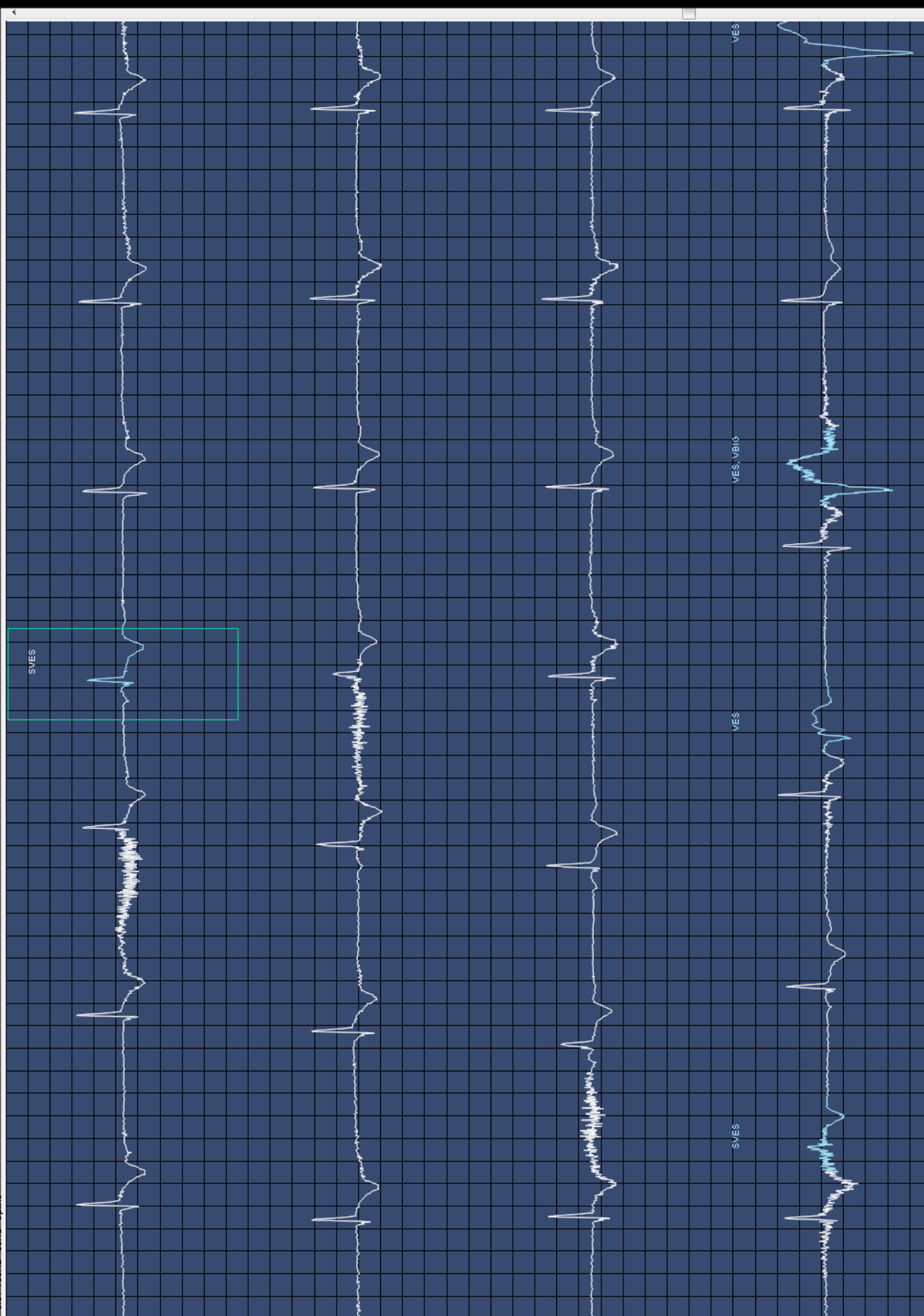

Supplement: Supplementary file 2 [file Data_Sheet_2.zip › EKG blindede/Subject 4 rest + max apnoea/4 max apnoea aVL.pdf]

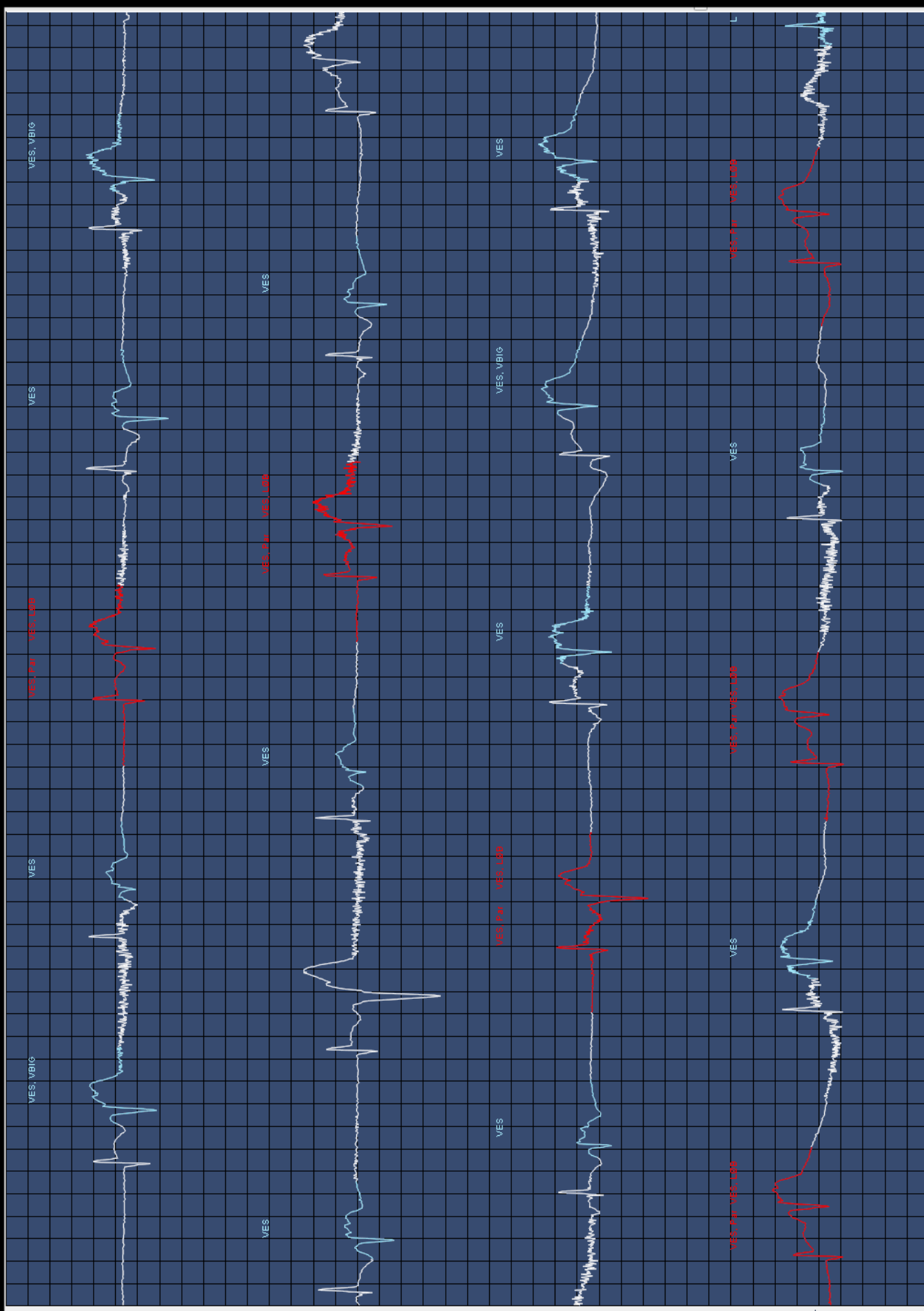

Supplement: Supplementary file 2 [file Data_Sheet_2.zip › EKG blindede/Subject 4 rest + max apnoea/4 max apnoea aVR no 2.pdf]

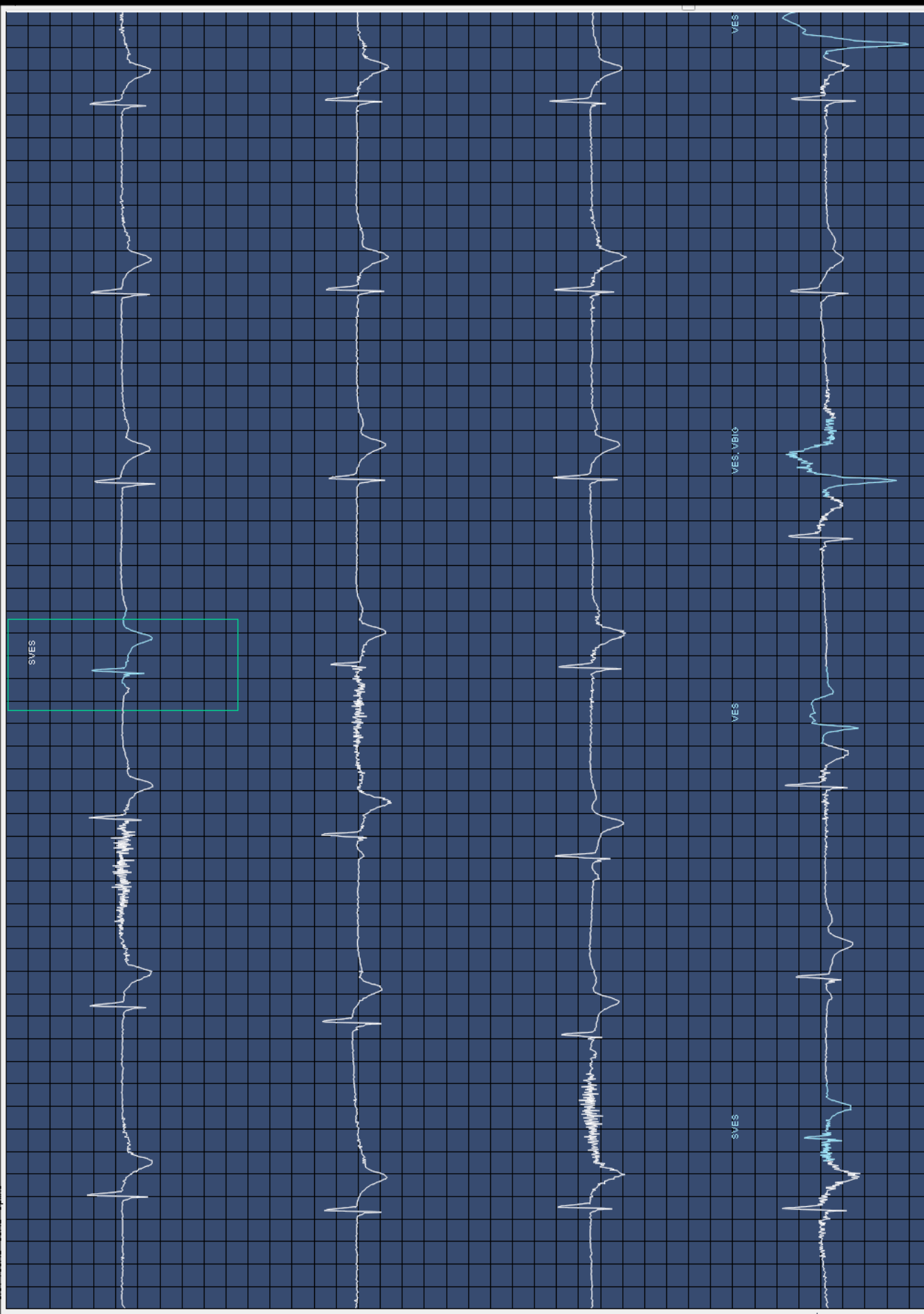

Supplement: Supplementary file 2 [file Data_Sheet_2.zip › EKG blindede/Subject 4 rest + max apnoea/4 max apnoea aVR.pdf]

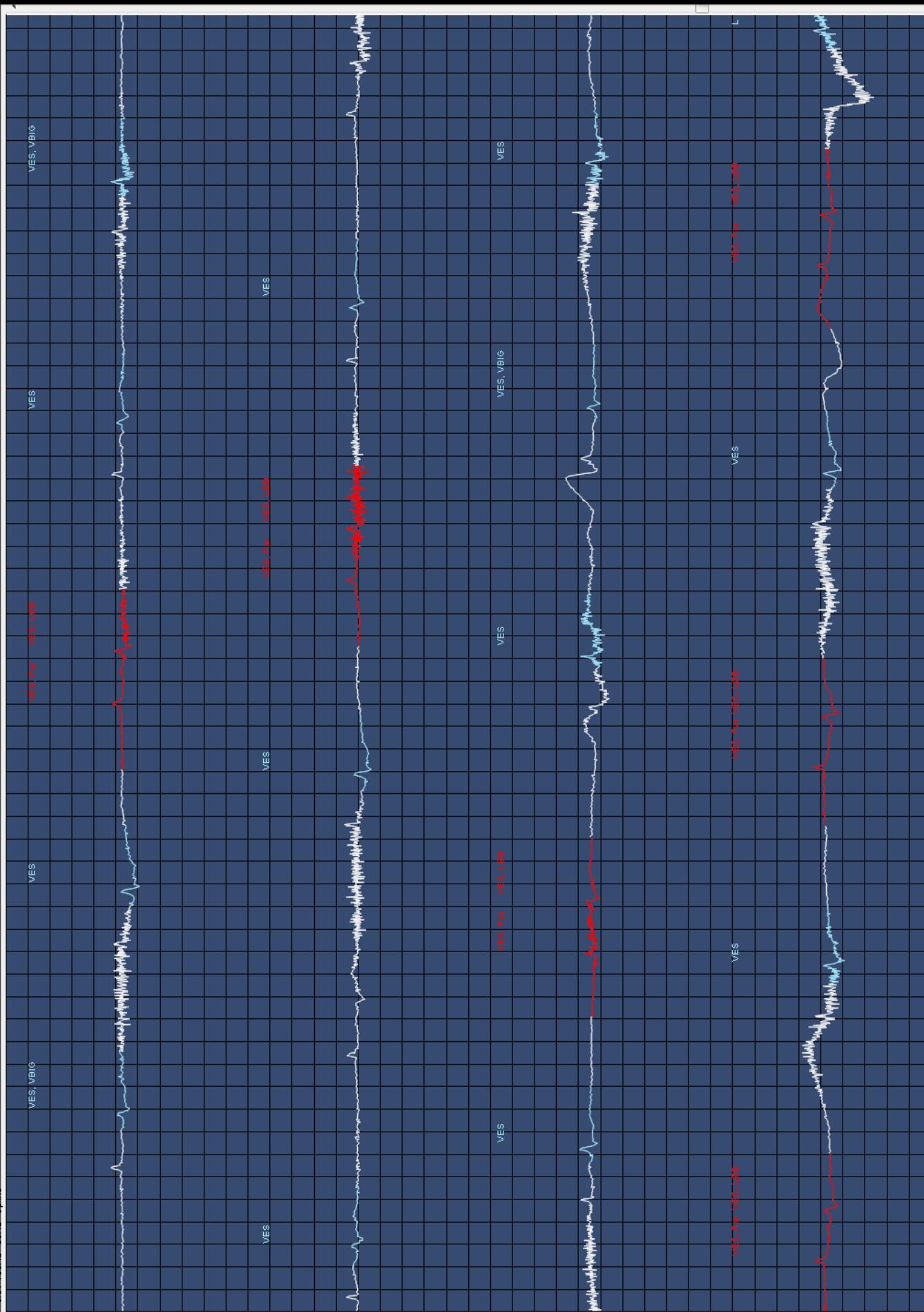

Supplement: Supplementary file 2 [file Data_Sheet_2.zip › EKG blindede/Subject 4 rest + max apnoea/4 max apnoea I no 2.pdf]

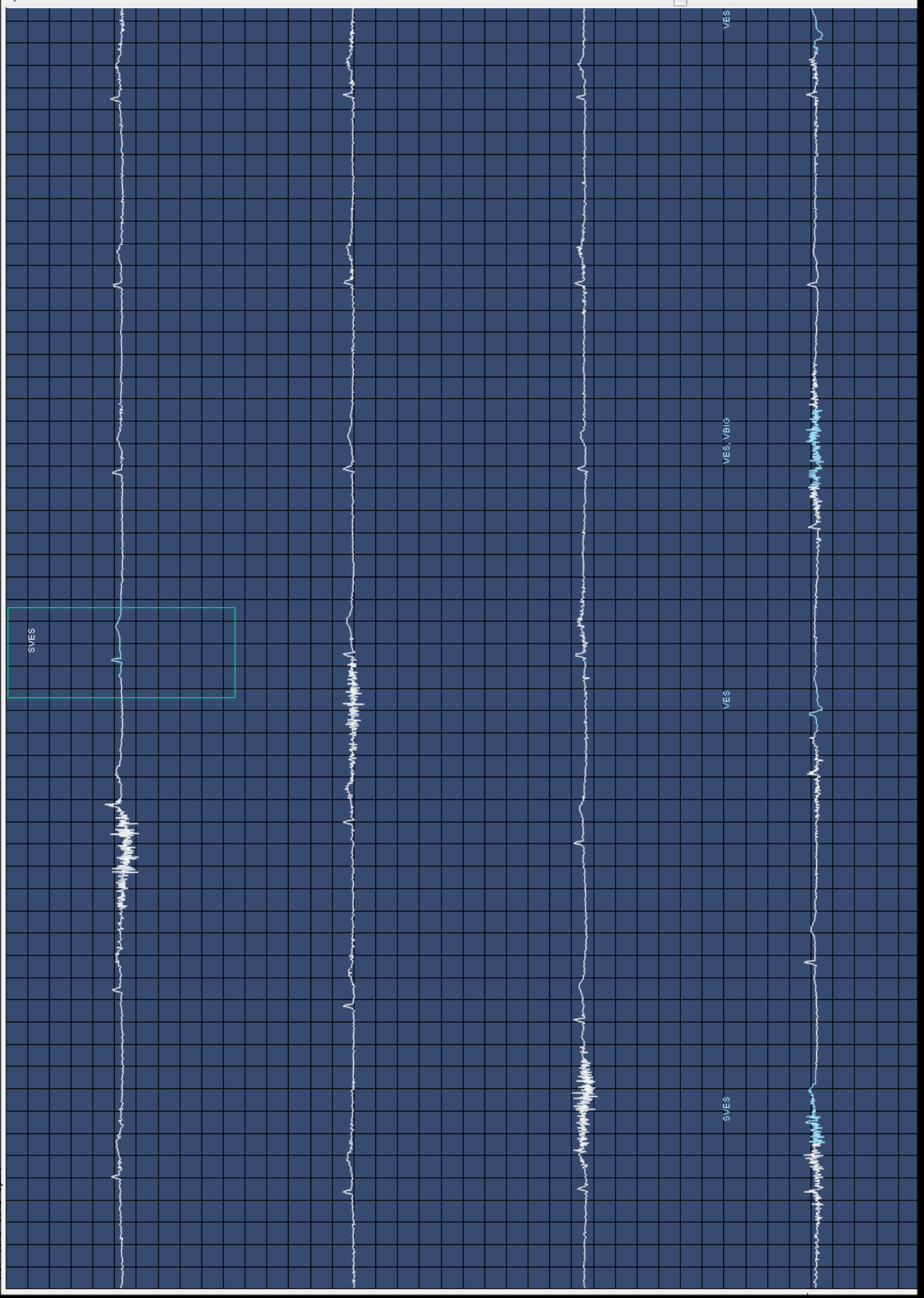

Supplement: Supplementary file 2 [file Data_Sheet_2.zip › EKG blindede/Subject 4 rest + max apnoea/4 max apnoea I.pdf]

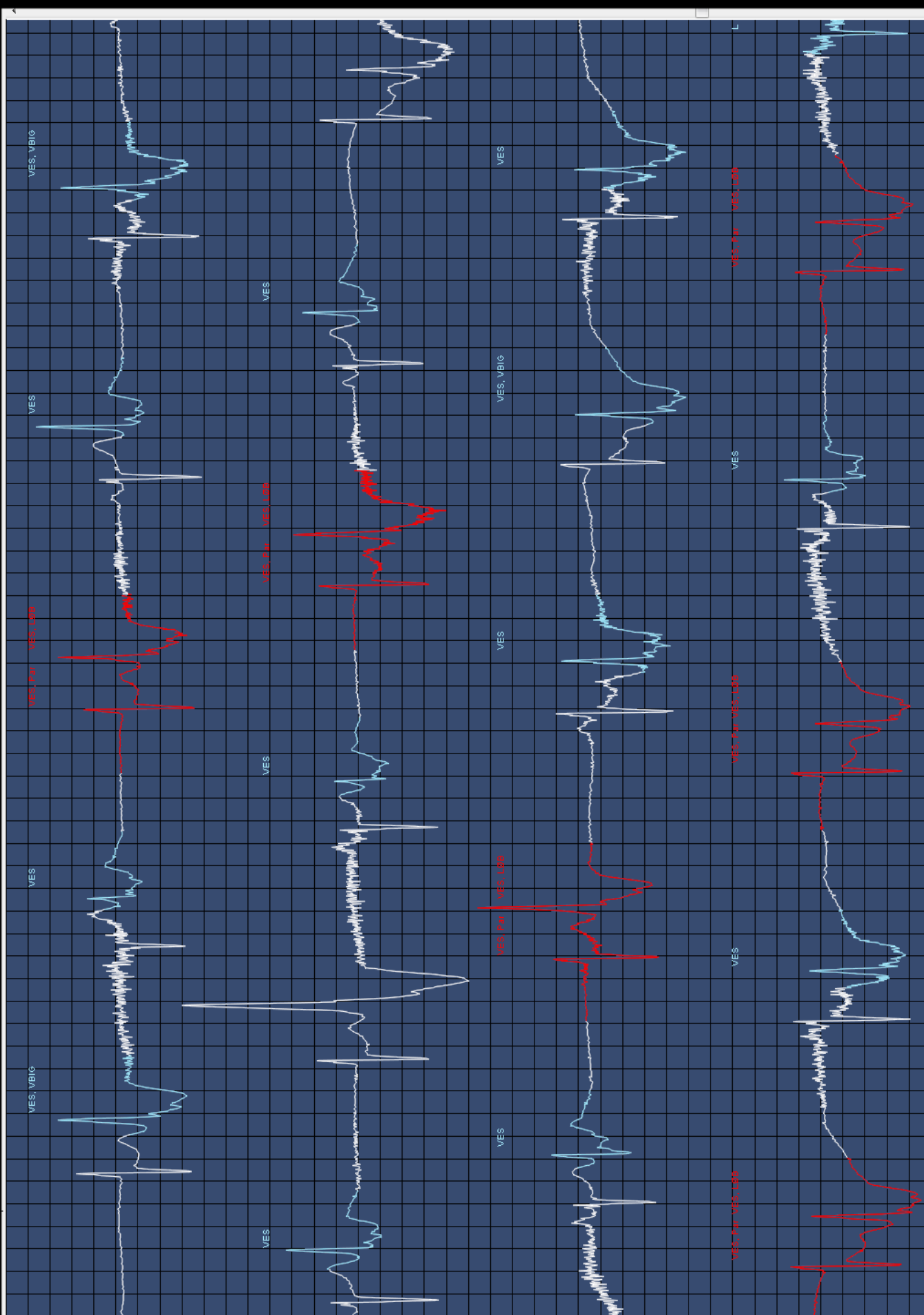

Supplement: Supplementary file 2 [file Data_Sheet_2.zip › EKG blindede/Subject 4 rest + max apnoea/4 max apnoea II no 2.pdf]

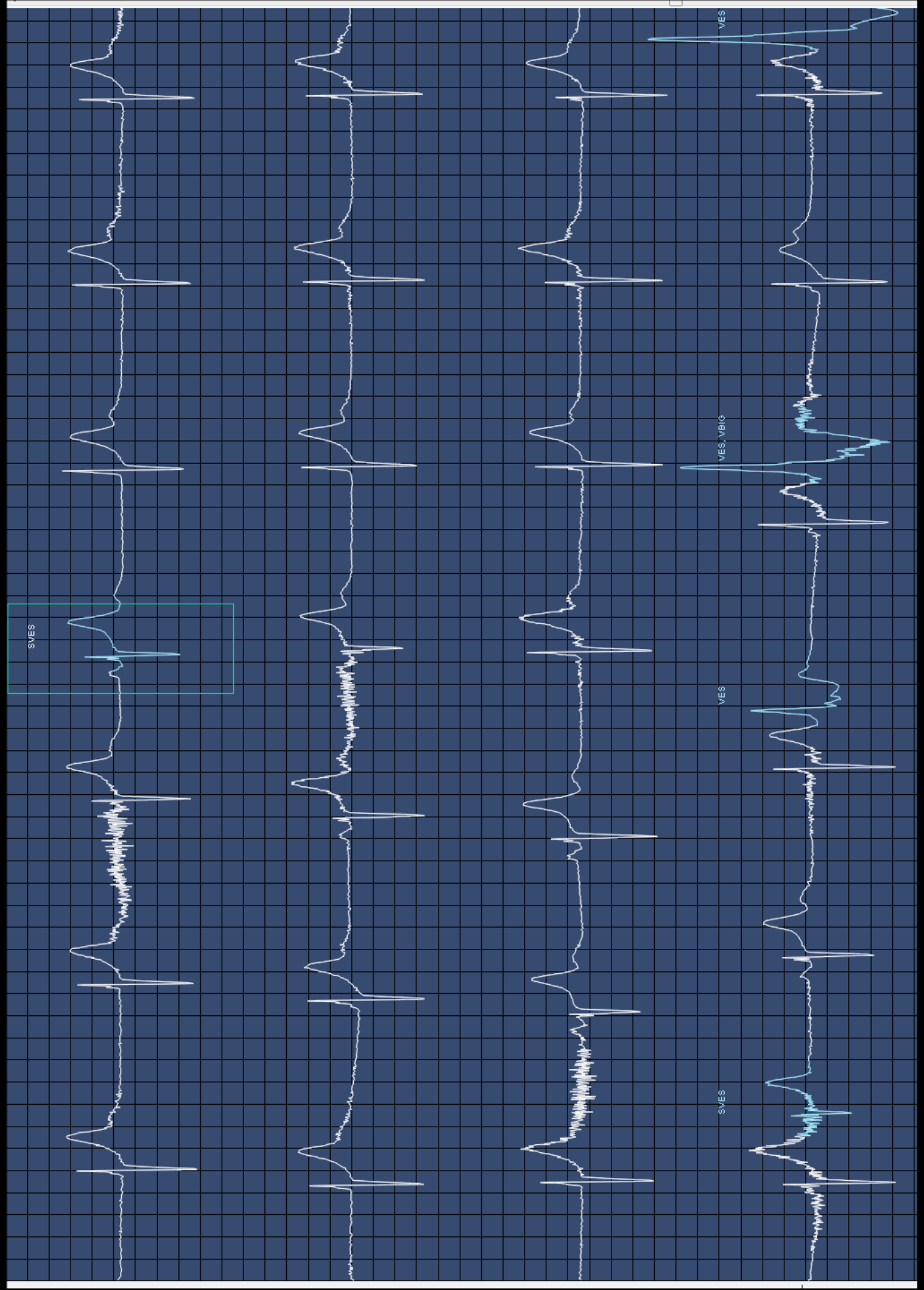

Supplement: Supplementary file 2 [file Data_Sheet_2.zip › EKG blindede/Subject 4 rest + max apnoea/4 max apnoea II.pdf]

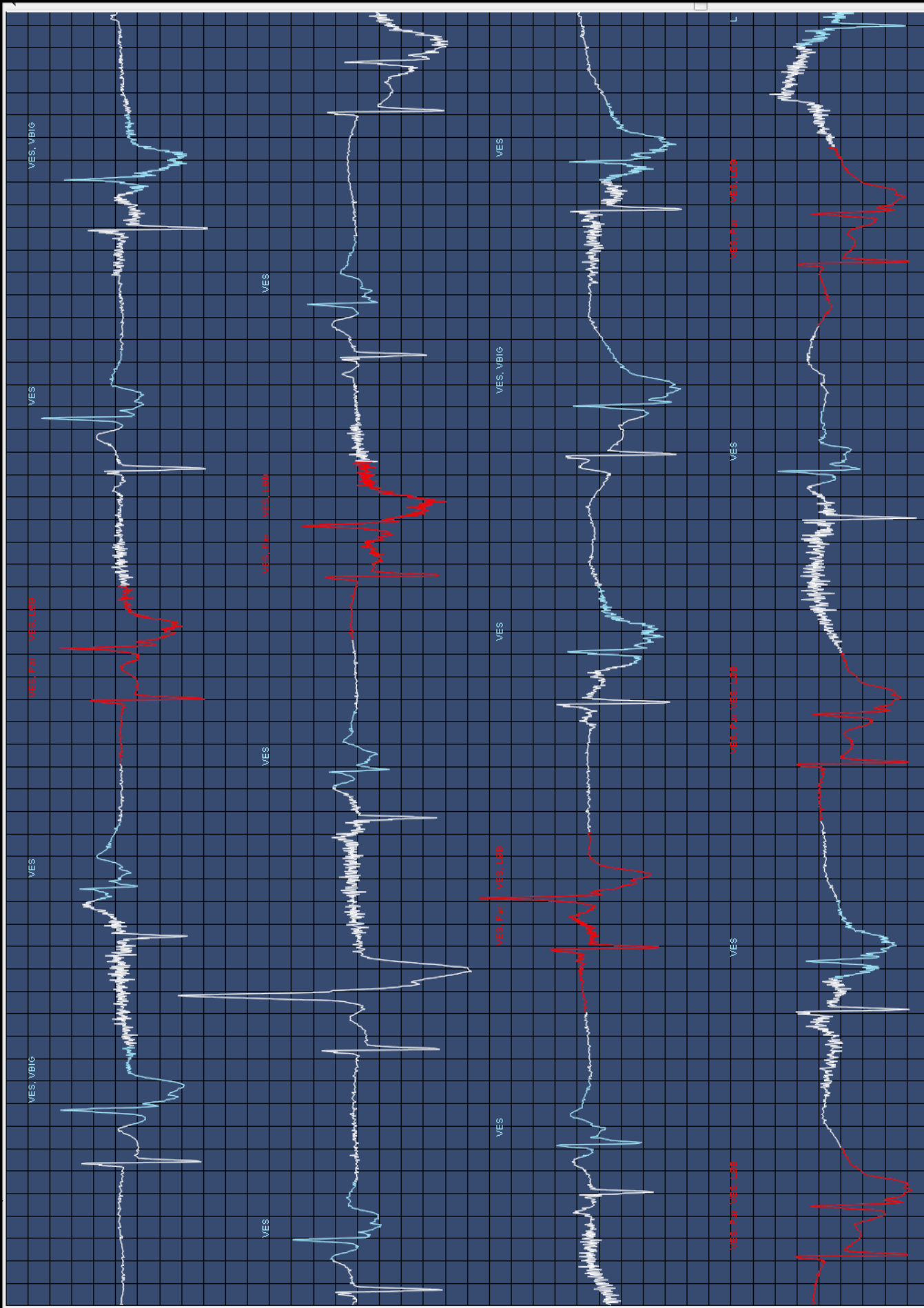

Supplement: Supplementary file 2 [file Data_Sheet_2.zip › EKG blindede/Subject 4 rest + max apnoea/4 max apnoea III no 2.pdf]

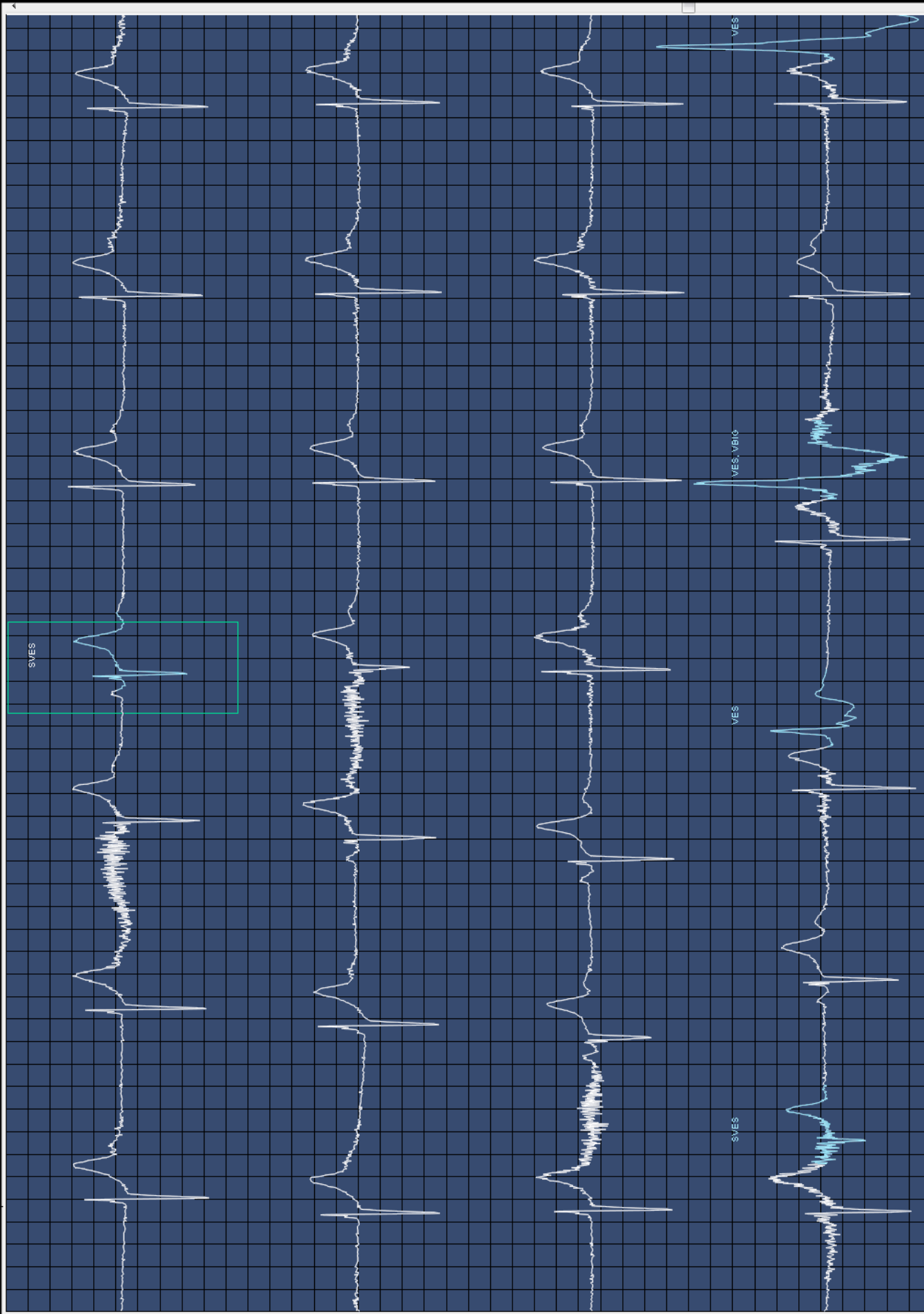

Supplement: Supplementary file 2 [file Data_Sheet_2.zip › EKG blindede/Subject 4 rest + max apnoea/4 max apnoea III.pdf]

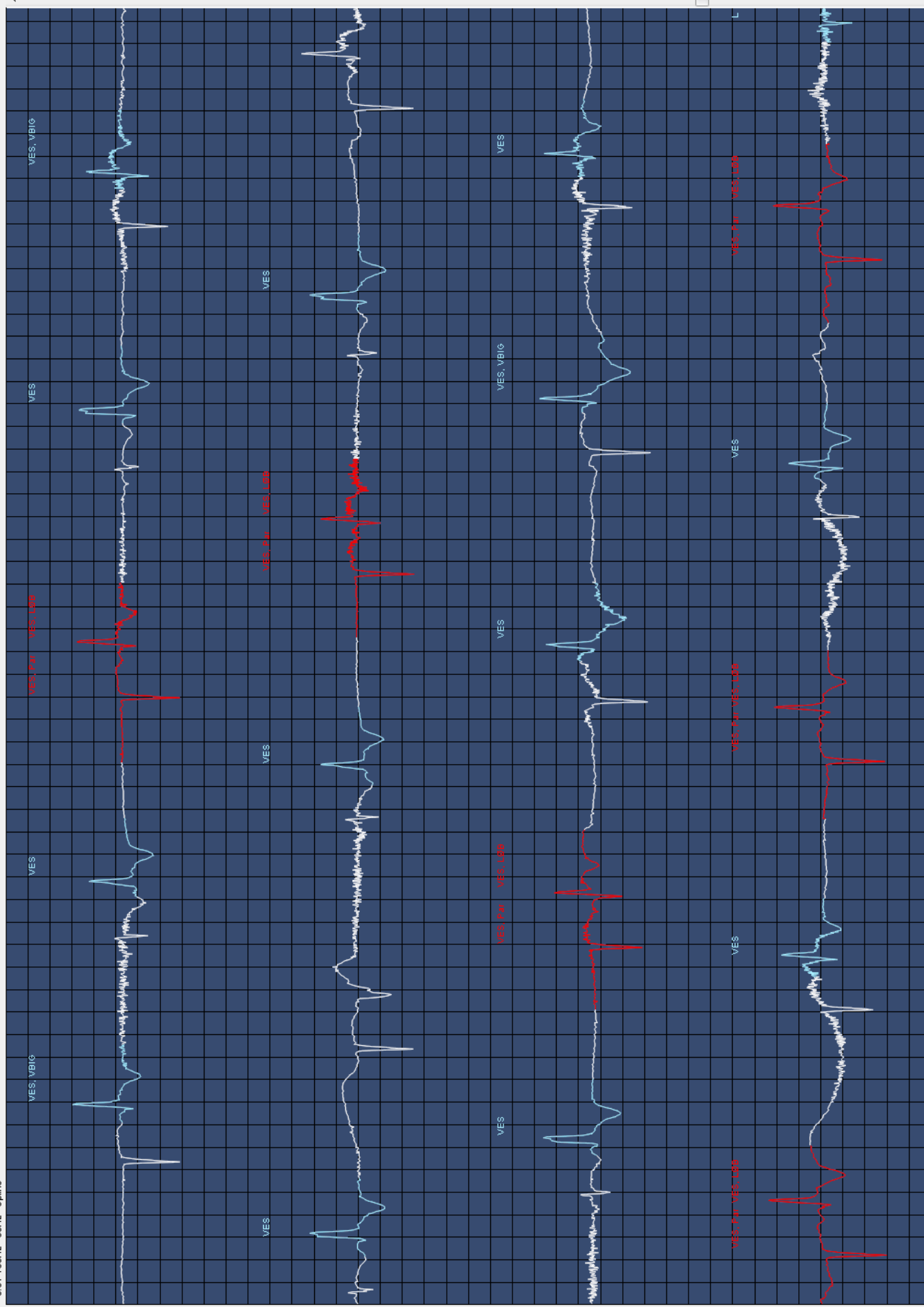

Supplement: Supplementary file 2 [file Data_Sheet_2.zip › EKG blindede/Subject 4 rest + max apnoea/4 max apnoea V1 no 2.pdf]

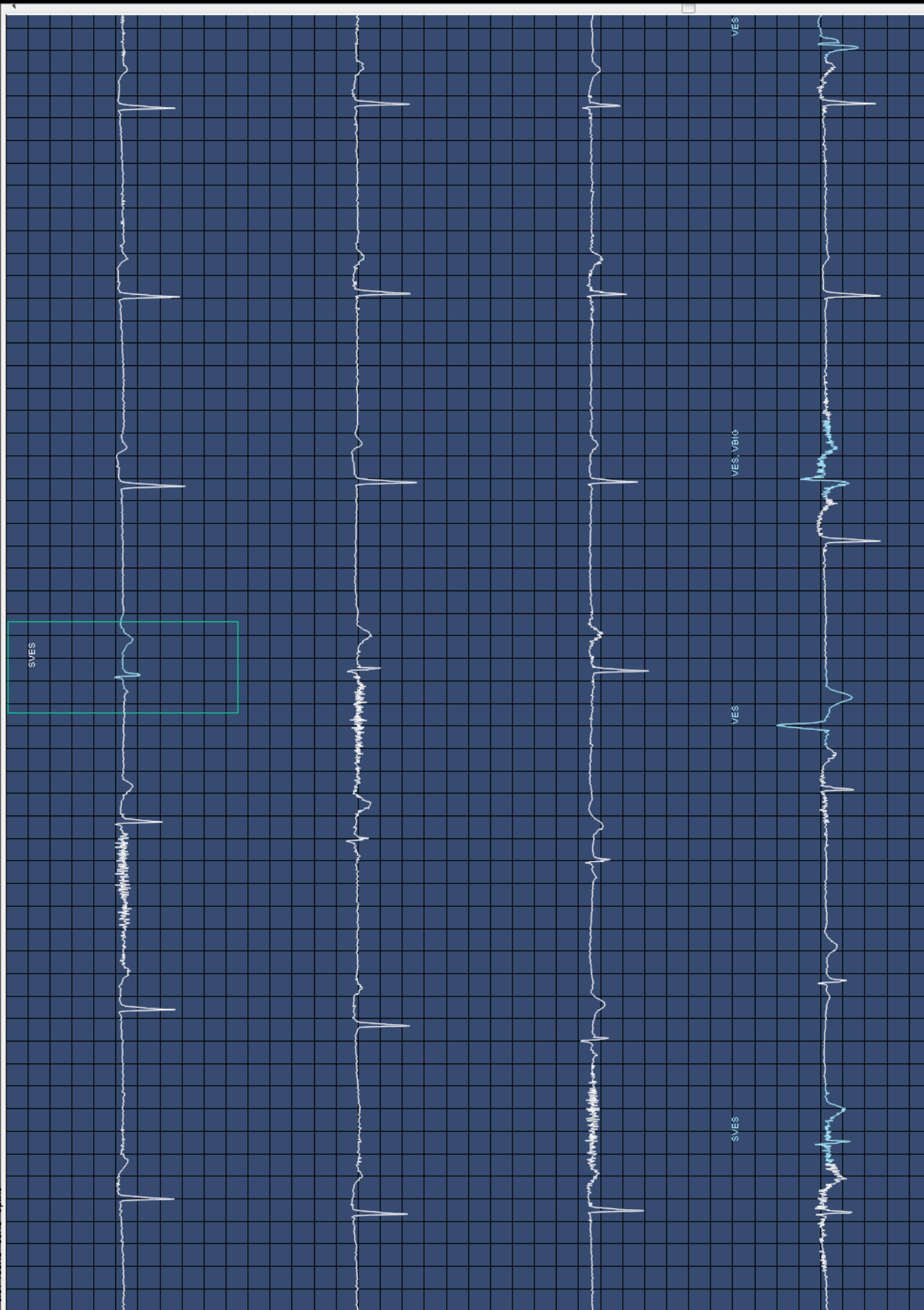

Supplement: Supplementary file 2 [file Data_Sheet_2.zip › EKG blindede/Subject 4 rest + max apnoea/4 max apnoea V1.pdf]

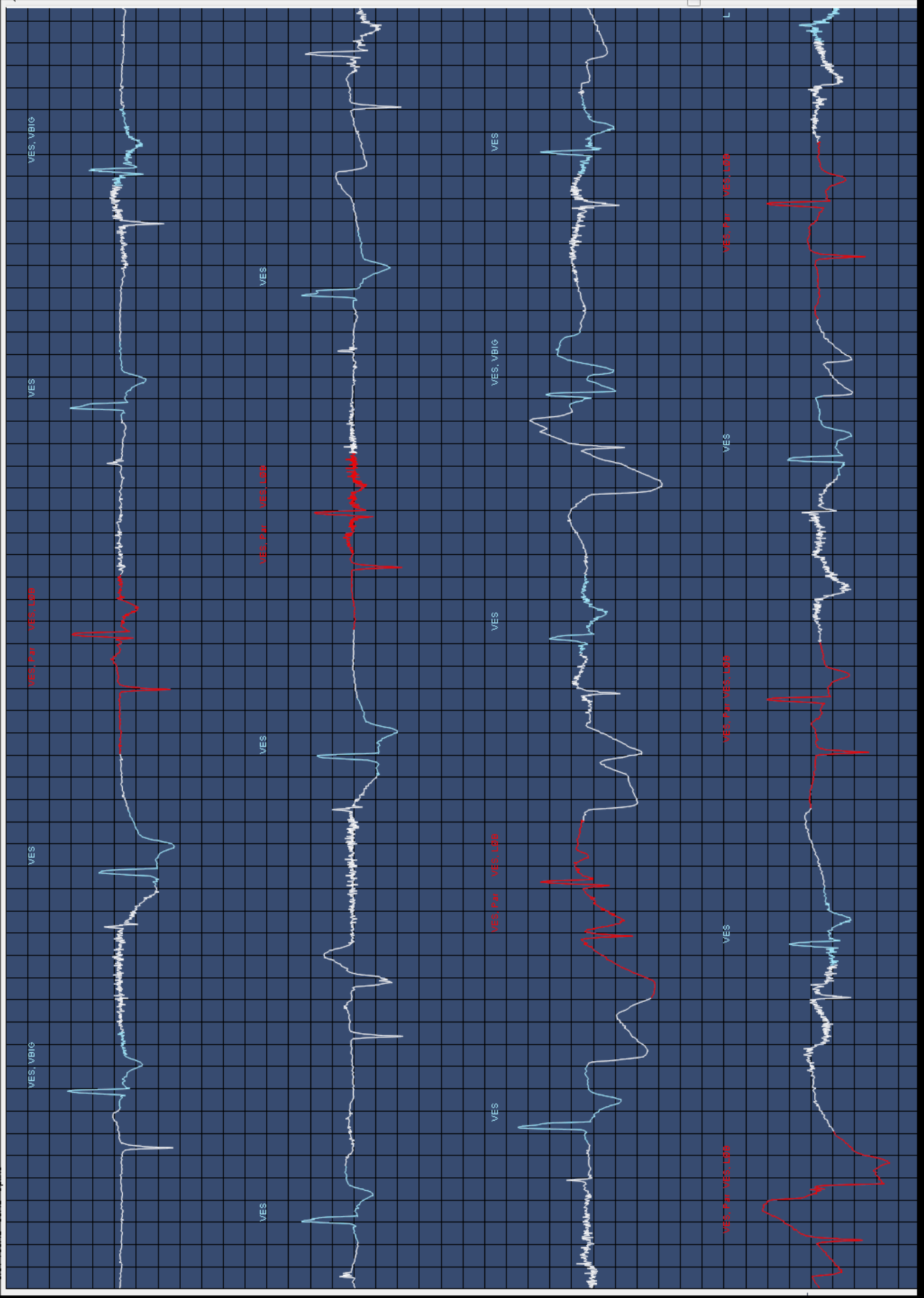

Supplement: Supplementary file 2 [file Data_Sheet_2.zip › EKG blindede/Subject 4 rest + max apnoea/4 max apnoea V2 no 2.pdf]

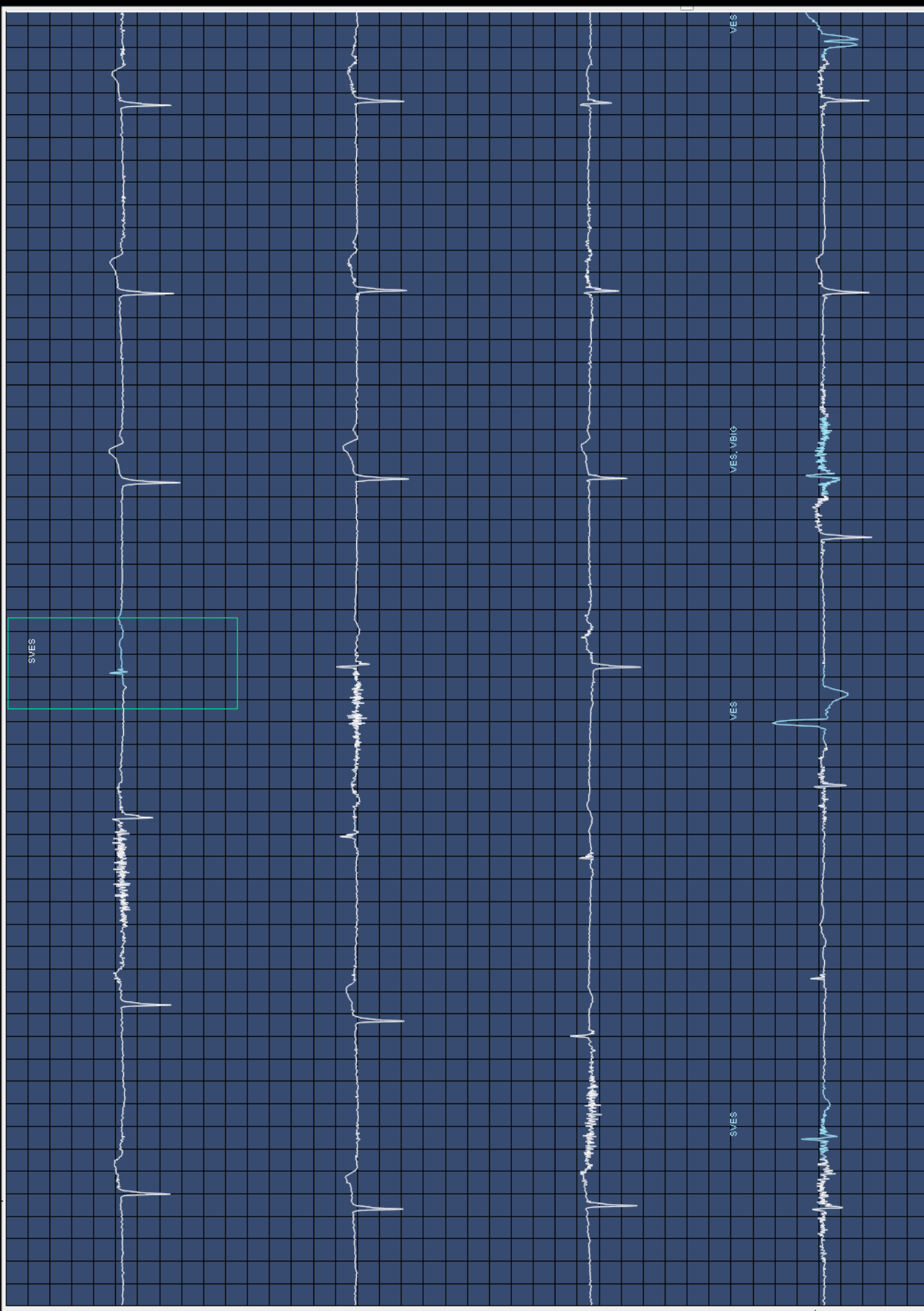

Supplement: Supplementary file 2 [file Data_Sheet_2.zip › EKG blindede/Subject 4 rest + max apnoea/4 max apnoea V2.pdf]

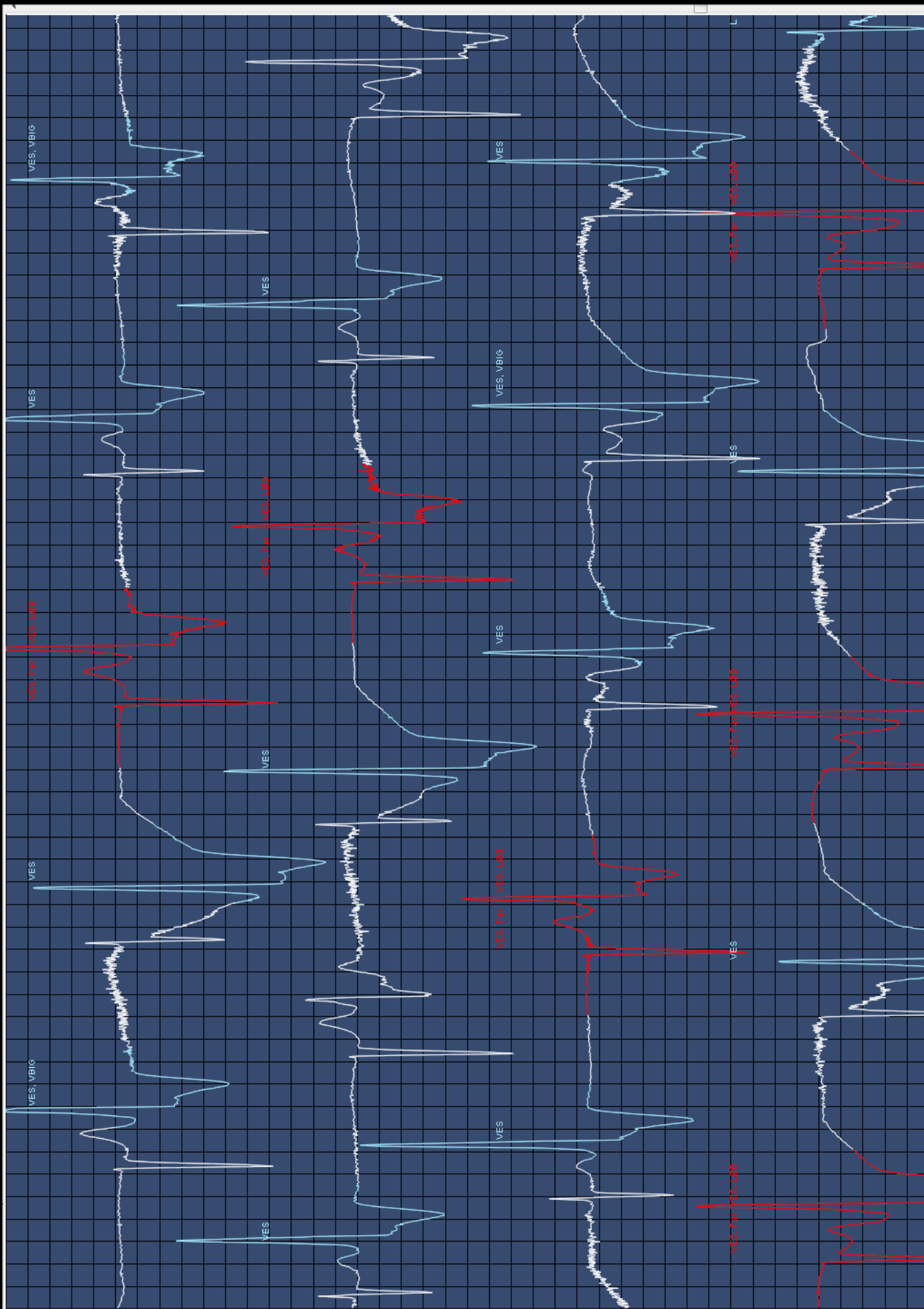

Supplement: Supplementary file 2 [file Data_Sheet_2.zip › EKG blindede/Subject 4 rest + max apnoea/4 max apnoea V3 no 2.pdf]

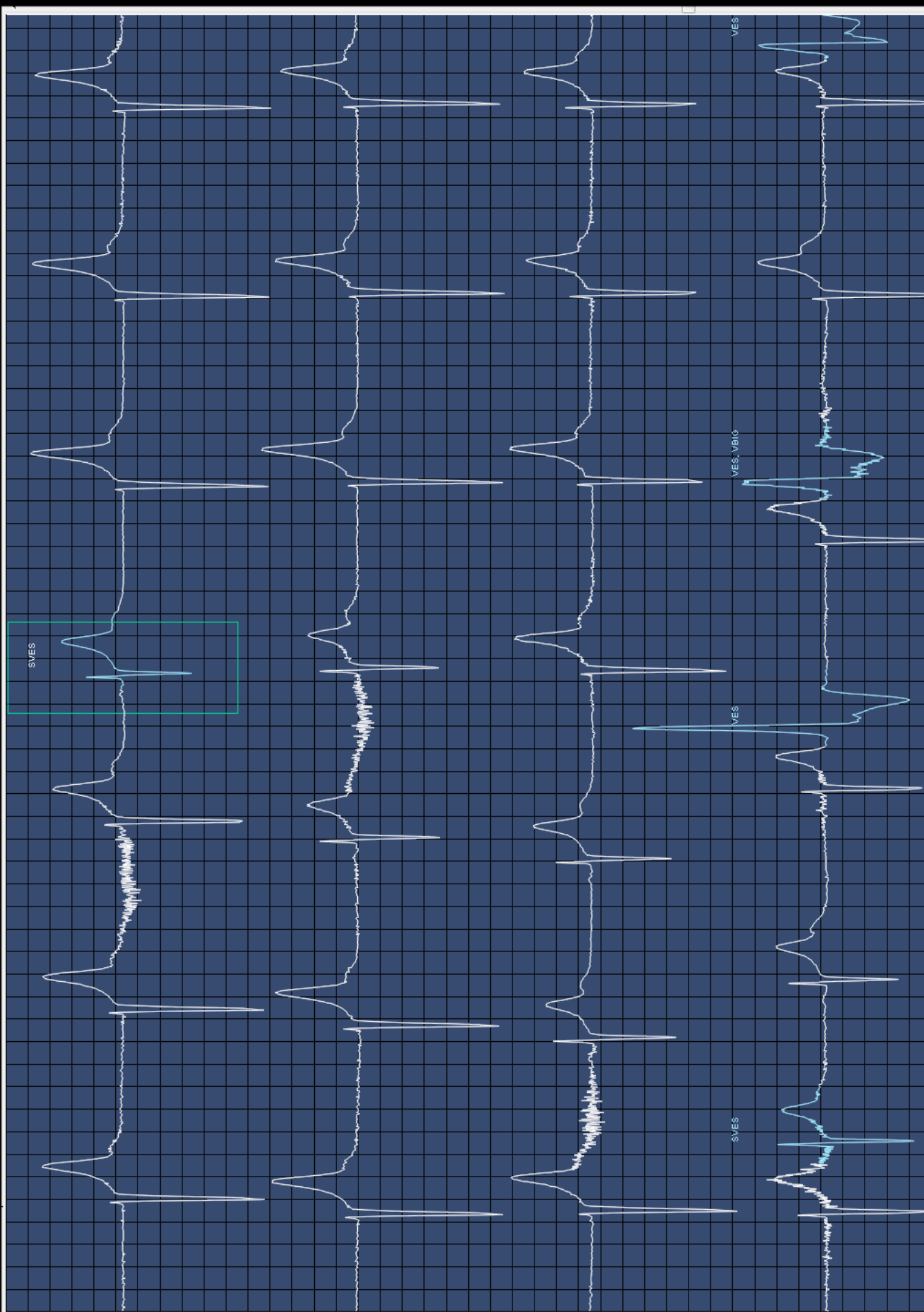

Supplement: Supplementary file 2 [file Data_Sheet_2.zip › EKG blindede/Subject 4 rest + max apnoea/4 max apnoea V3.pdf]

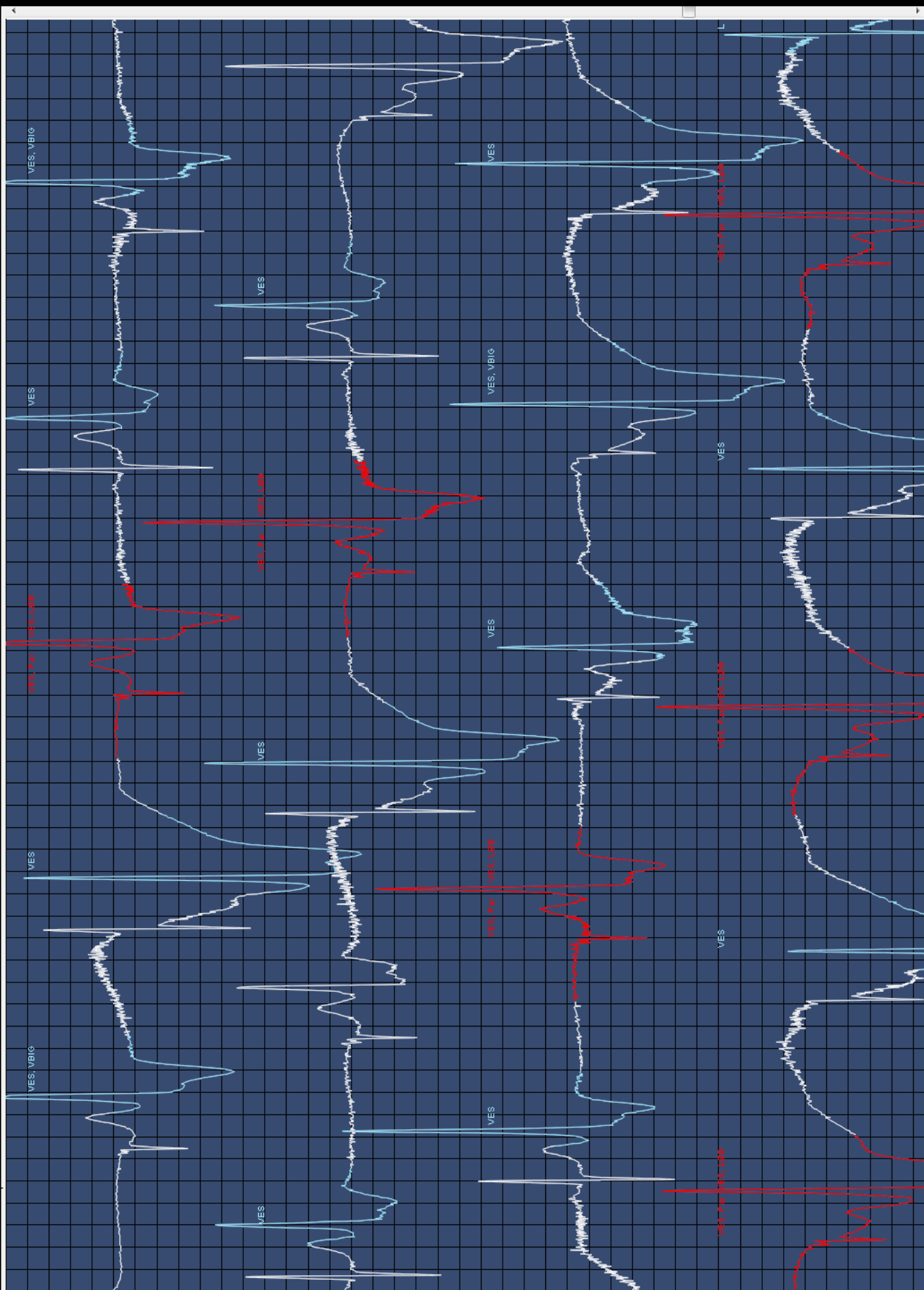

Supplement: Supplementary file 2 [file Data_Sheet_2.zip › EKG blindede/Subject 4 rest + max apnoea/4 max apnoea V4 no 2.pdf]

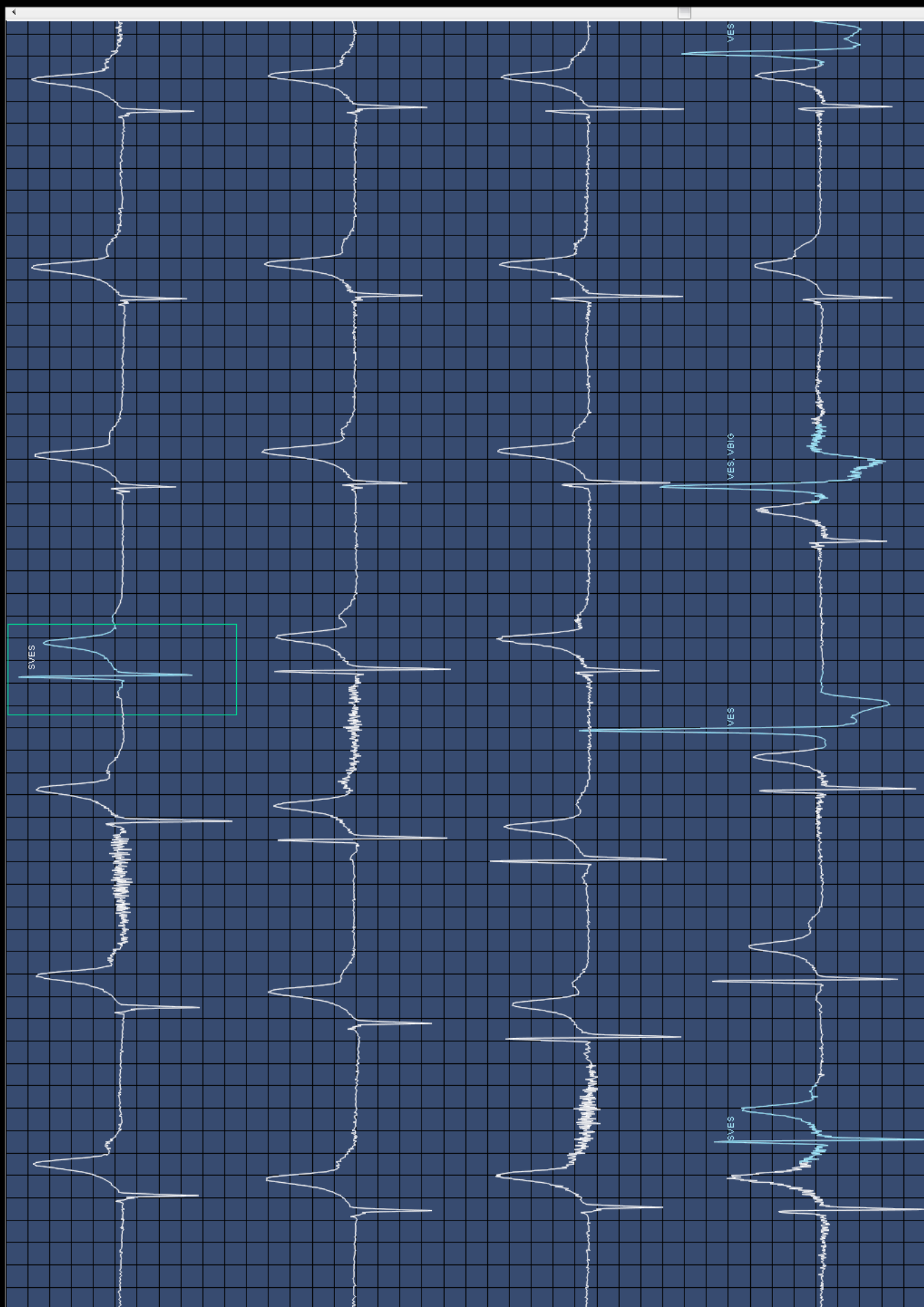

Supplement: Supplementary file 2 [file Data_Sheet_2.zip › EKG blindede/Subject 4 rest + max apnoea/4 max apnoea V4.pdf]

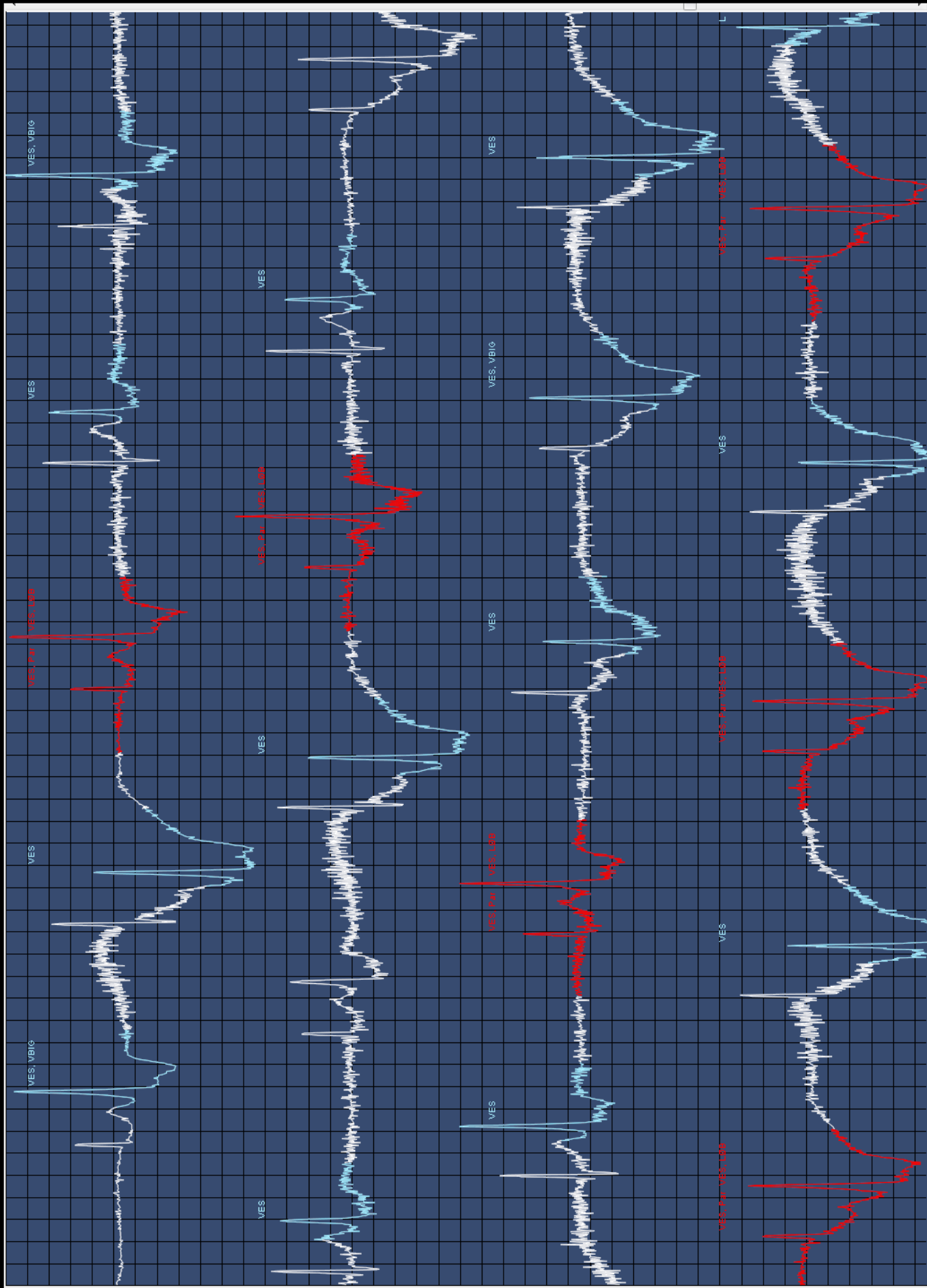

Supplement: Supplementary file 2 [file Data_Sheet_2.zip › EKG blindede/Subject 4 rest + max apnoea/4 max apnoea V5 no 2.pdf]

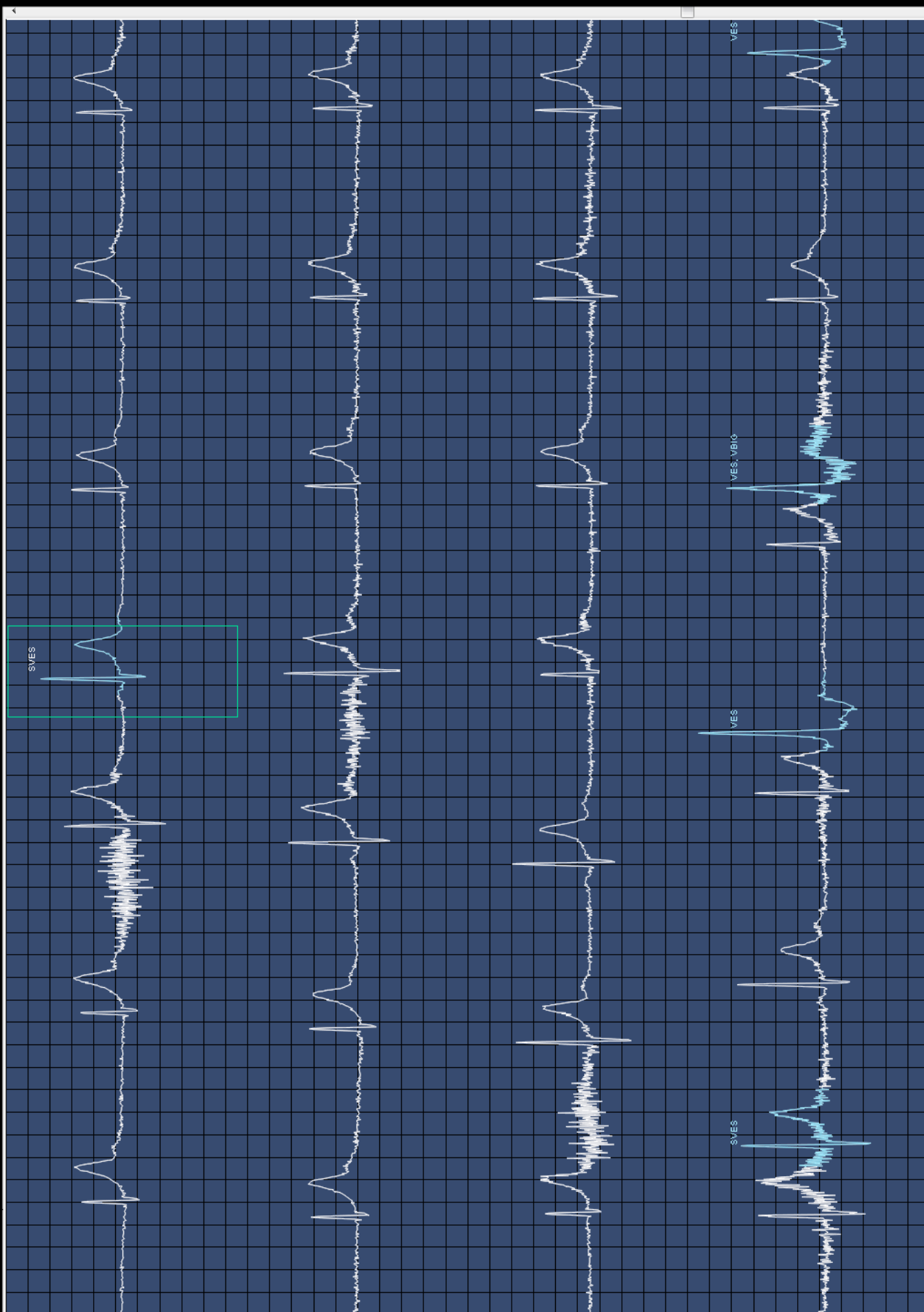

Supplement: Supplementary file 2 [file Data_Sheet_2.zip › EKG blindede/Subject 4 rest + max apnoea/4 max apnoea V5.pdf]

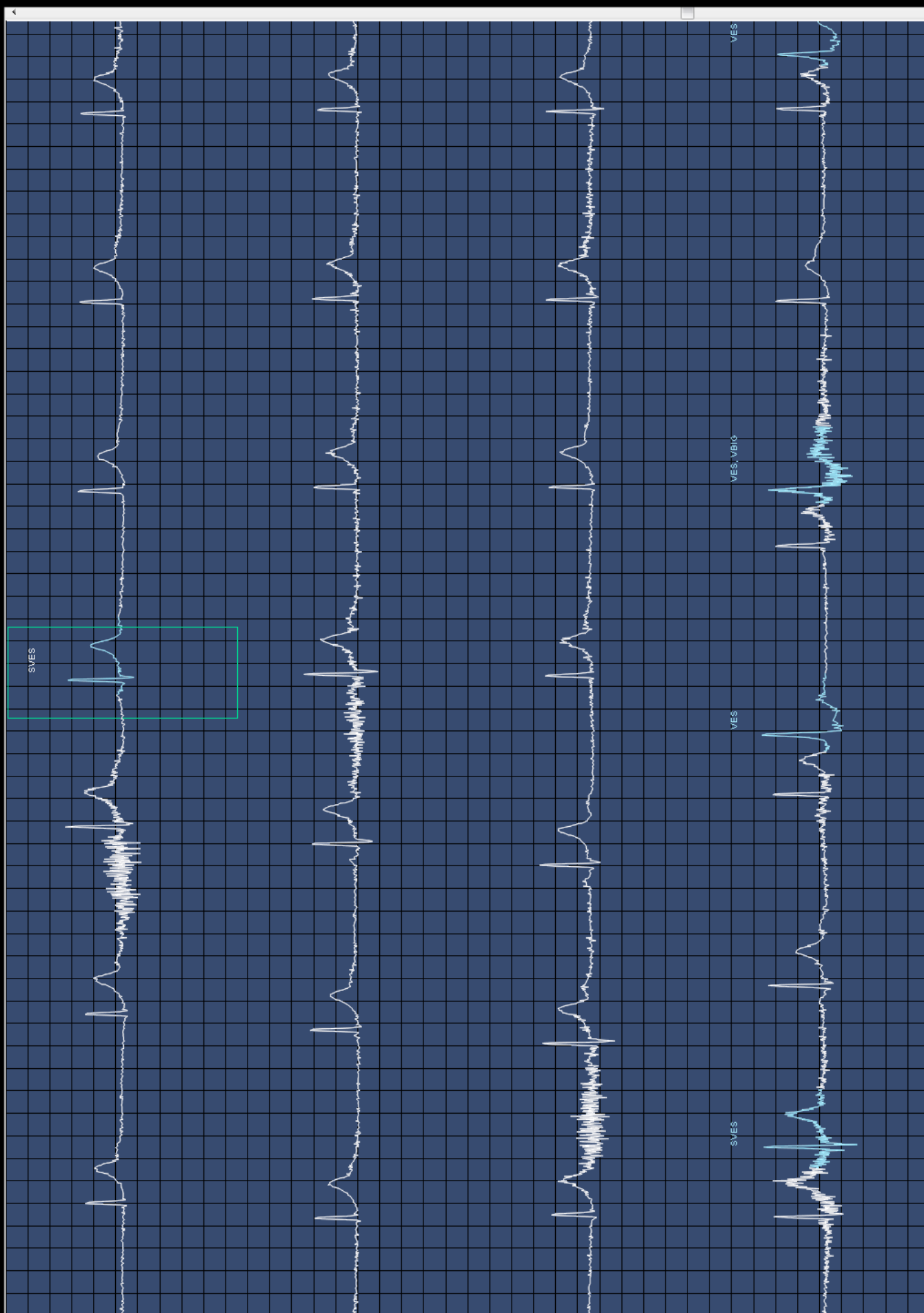

Supplement: Supplementary file 2 [file Data_Sheet_2.zip › EKG blindede/Subject 4 rest + max apnoea/4 max apnoea V6.pdf]

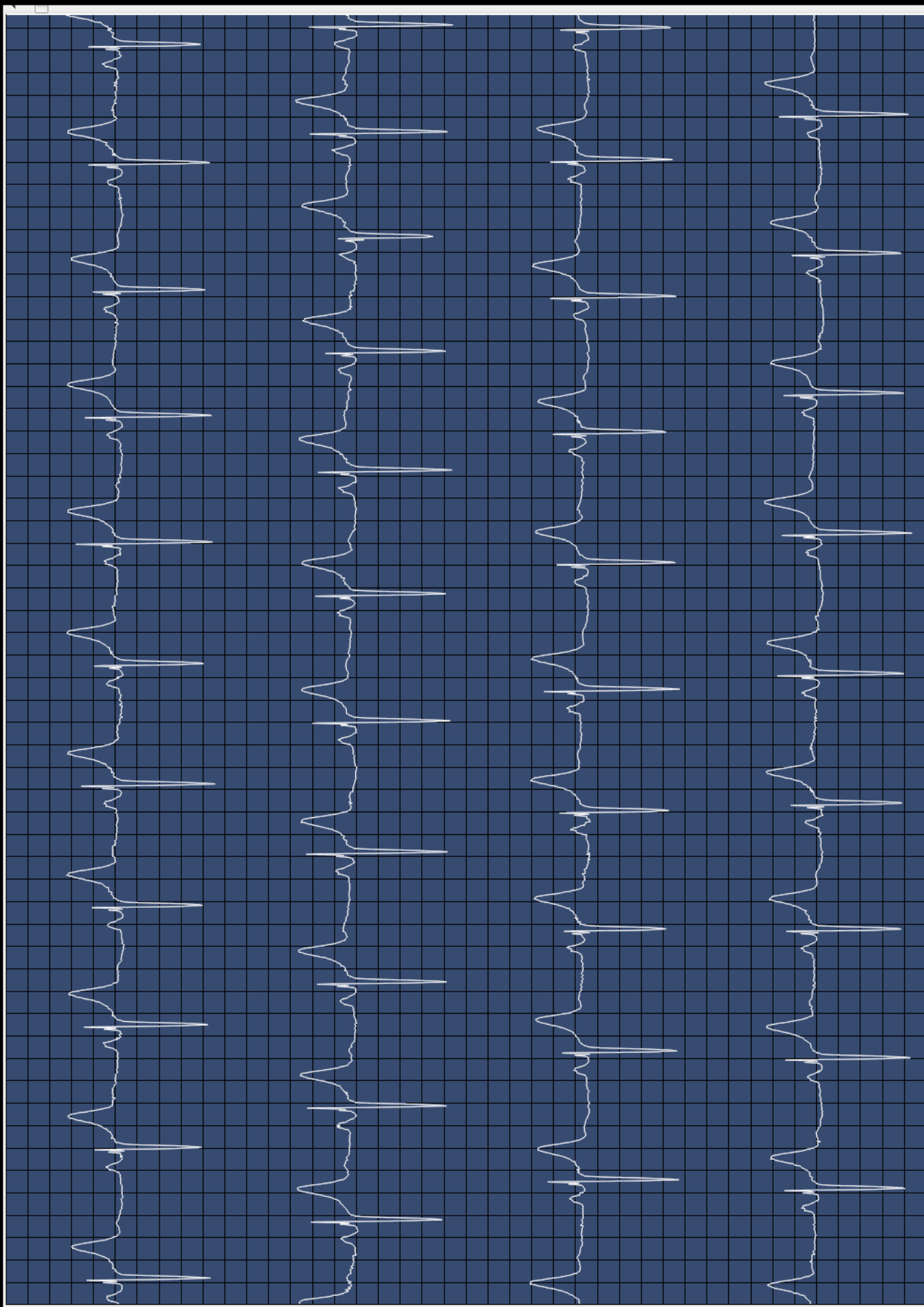

Supplement: Supplementary file 2 [file Data_Sheet_2.zip › EKG blindede/Subject 4 rest + max apnoea/4 rest aVF.pdf]

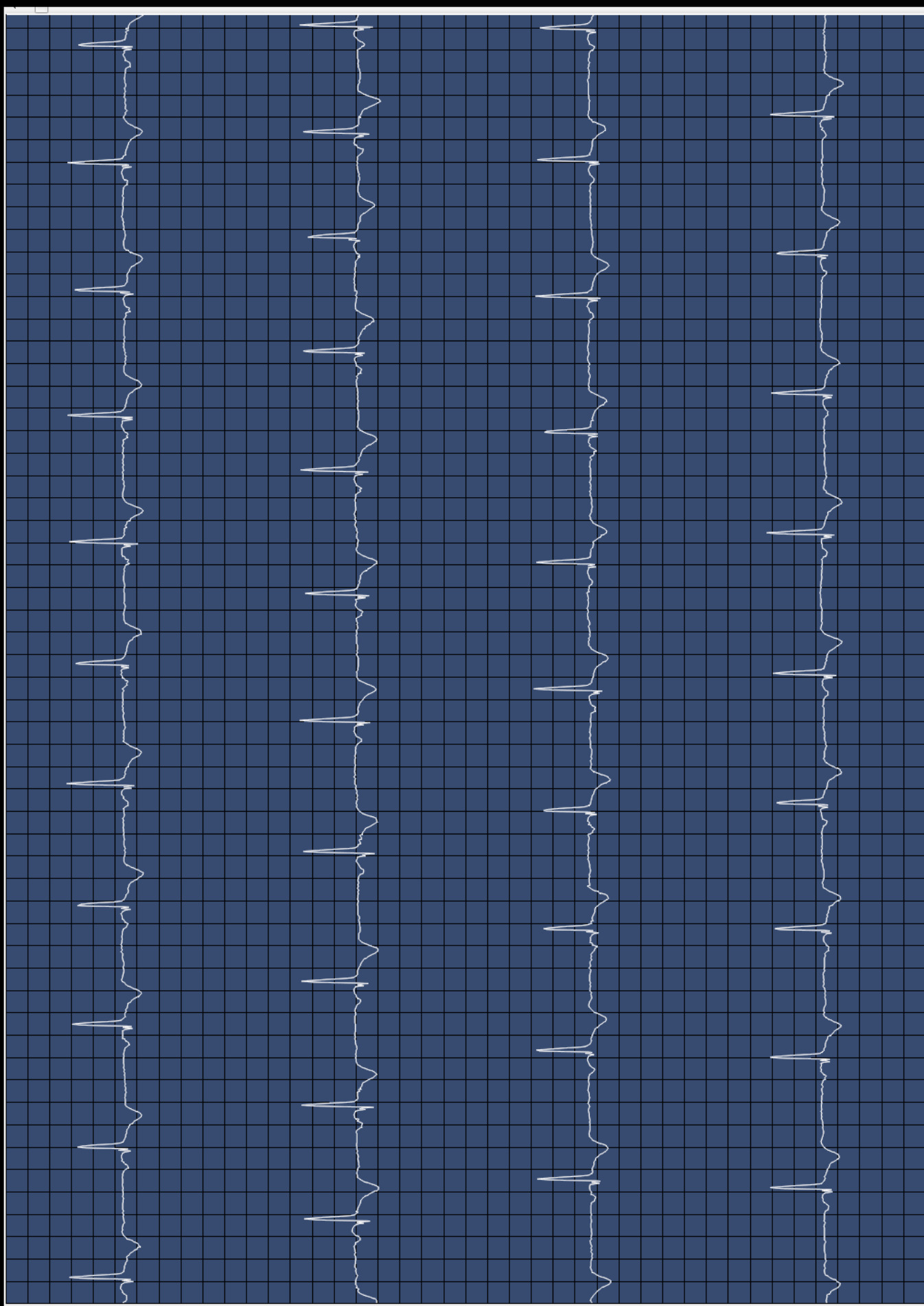

Supplement: Supplementary file 2 [file Data_Sheet_2.zip › EKG blindede/Subject 4 rest + max apnoea/4 rest aVL.pdf]

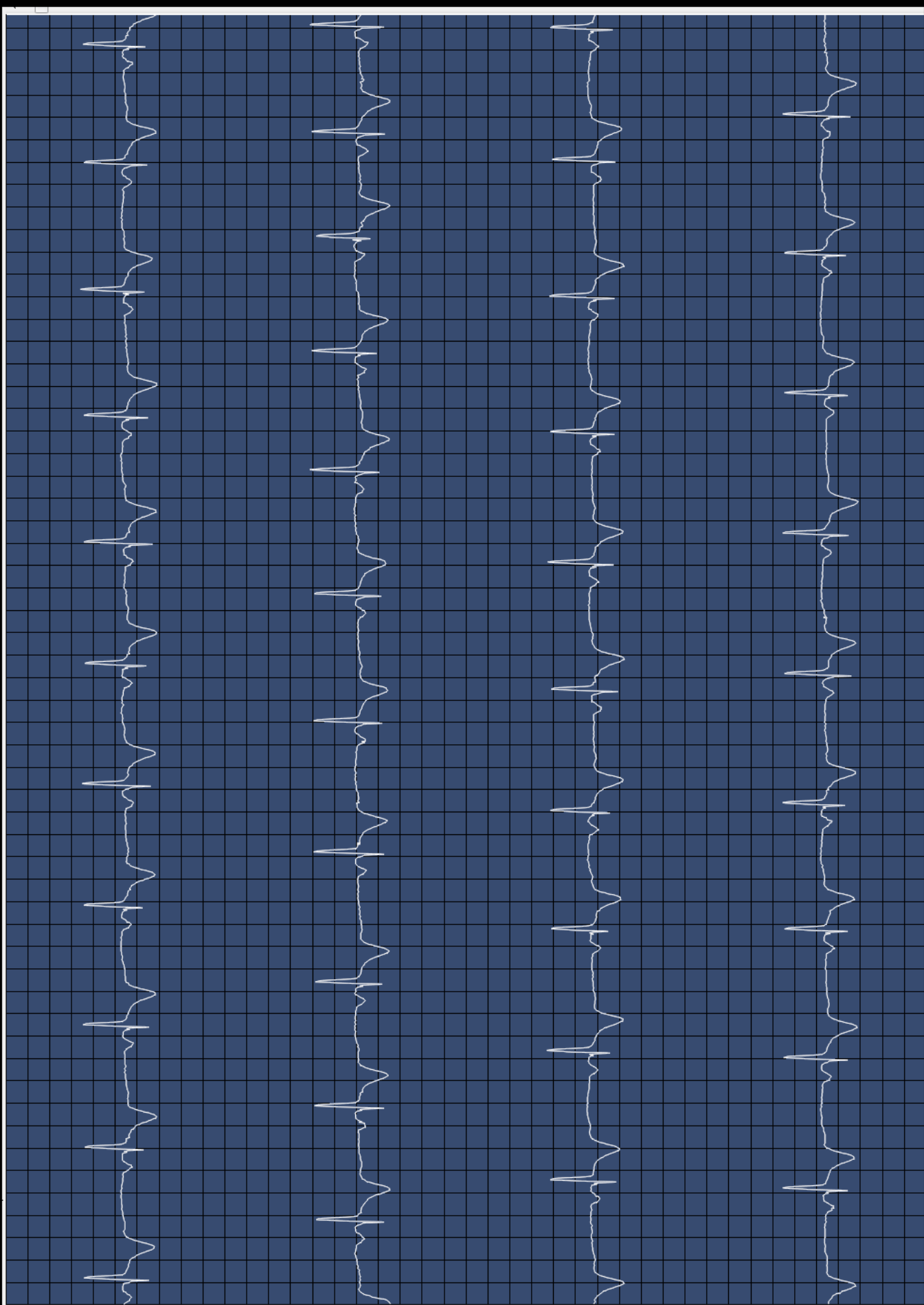

Supplement: Supplementary file 2 [file Data_Sheet_2.zip › EKG blindede/Subject 4 rest + max apnoea/4 rest aVR.pdf]

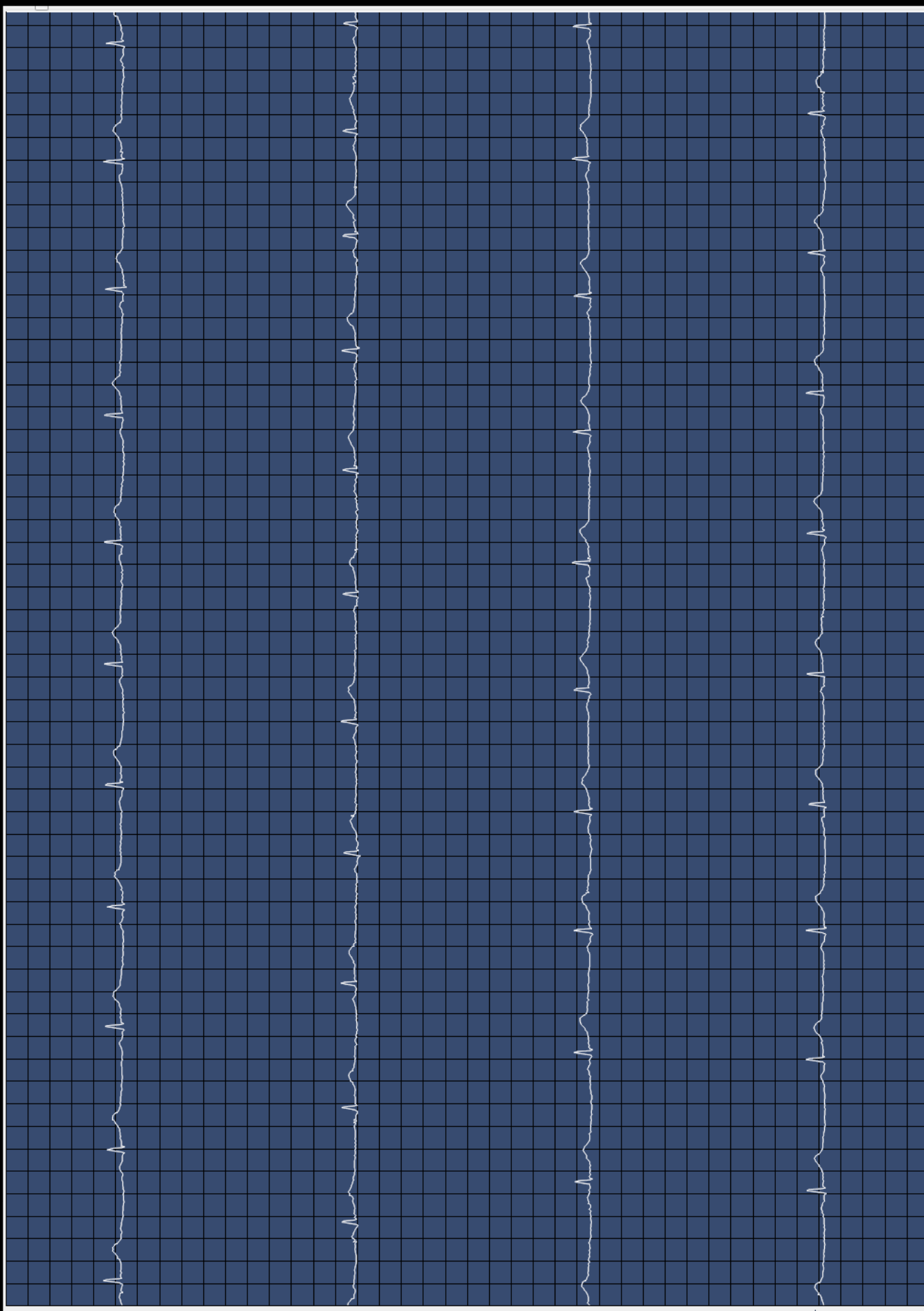

Supplement: Supplementary file 2 [file Data_Sheet_2.zip › EKG blindede/Subject 4 rest + max apnoea/4 rest I.pdf]

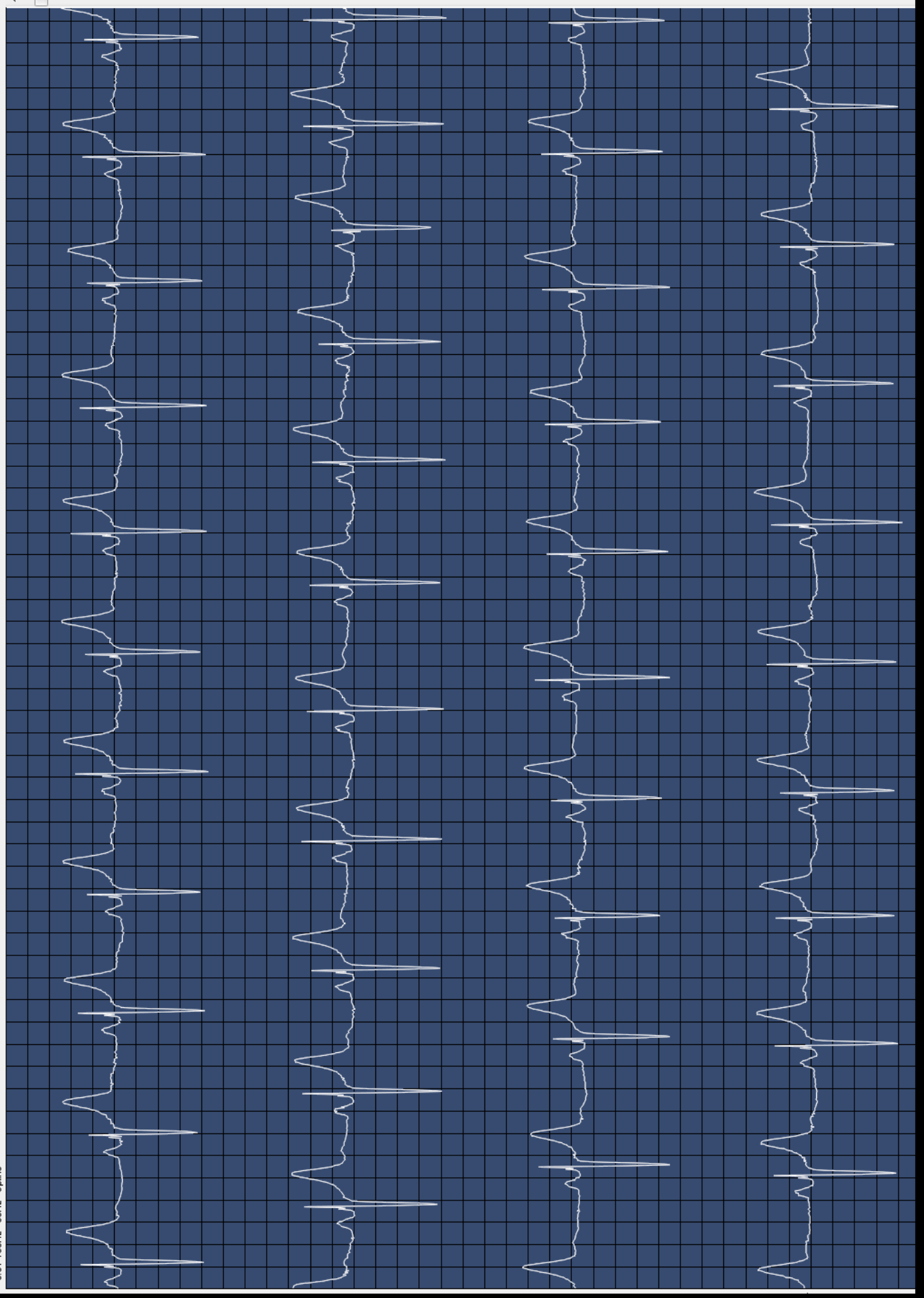

Supplement: Supplementary file 2 [file Data_Sheet_2.zip › EKG blindede/Subject 4 rest + max apnoea/4 rest II.pdf]

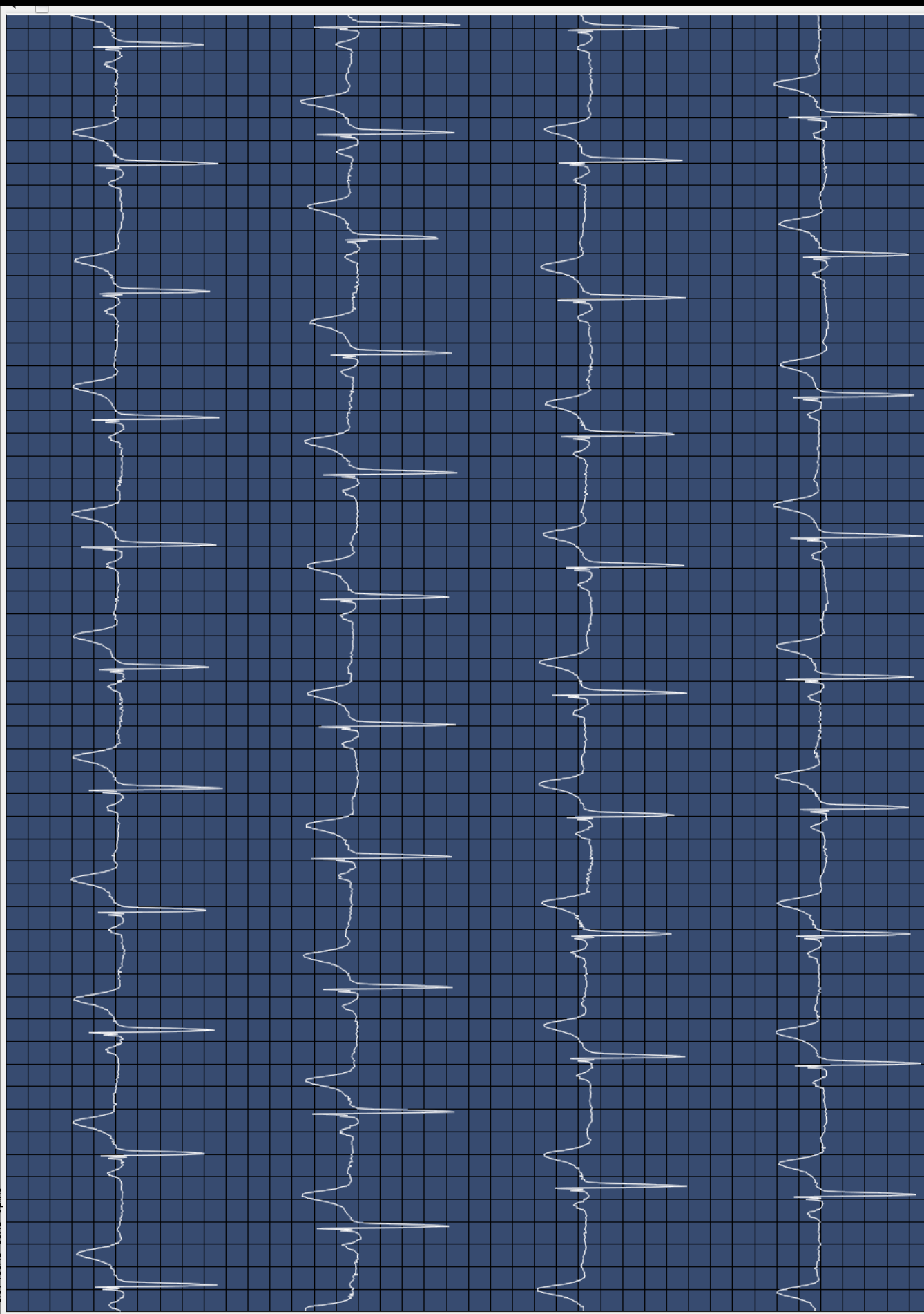

Supplement: Supplementary file 2 [file Data_Sheet_2.zip › EKG blindede/Subject 4 rest + max apnoea/4 rest III.pdf]

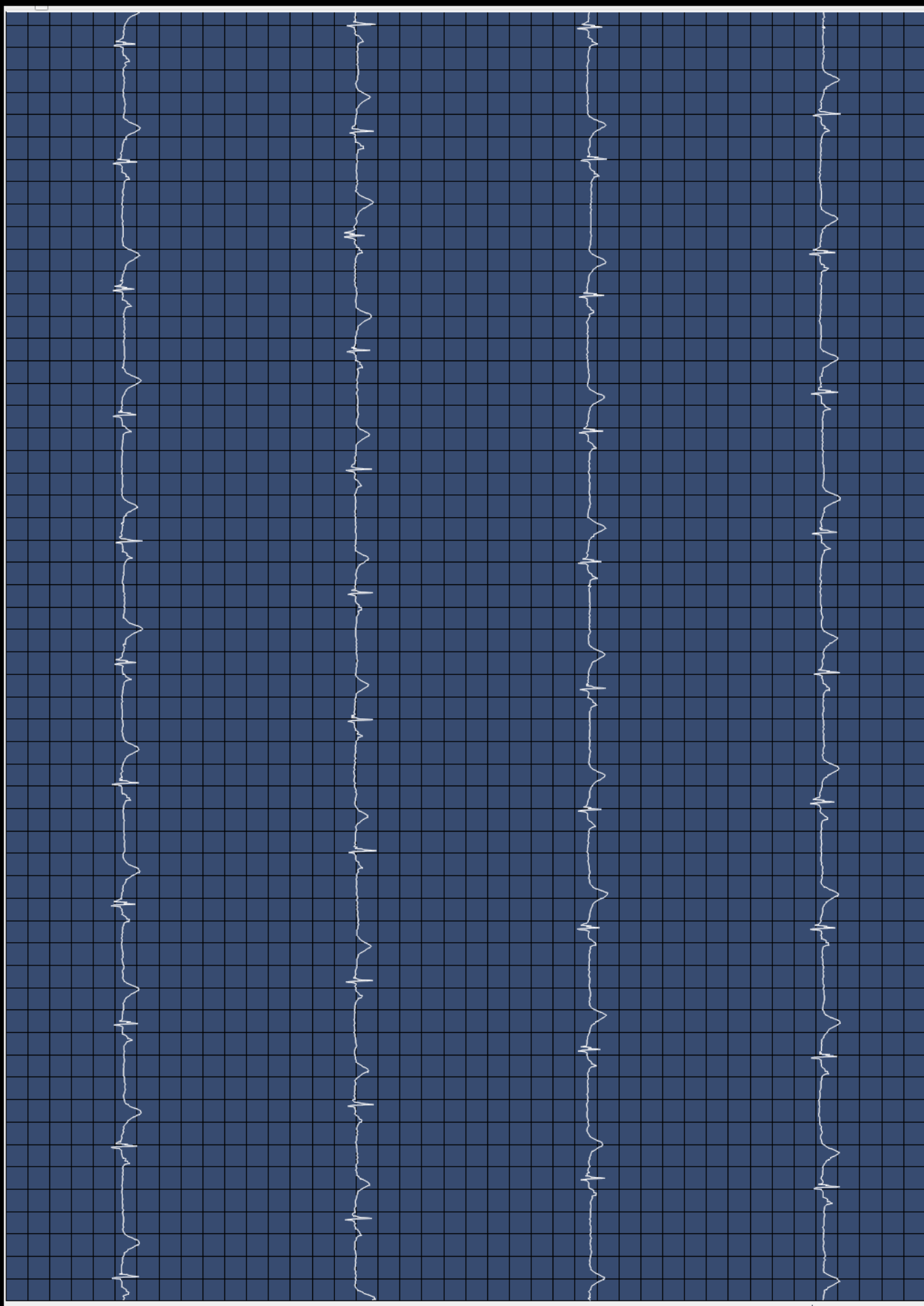

Supplement: Supplementary file 2 [file Data_Sheet_2.zip › EKG blindede/Subject 4 rest + max apnoea/4 rest V1.pdf]

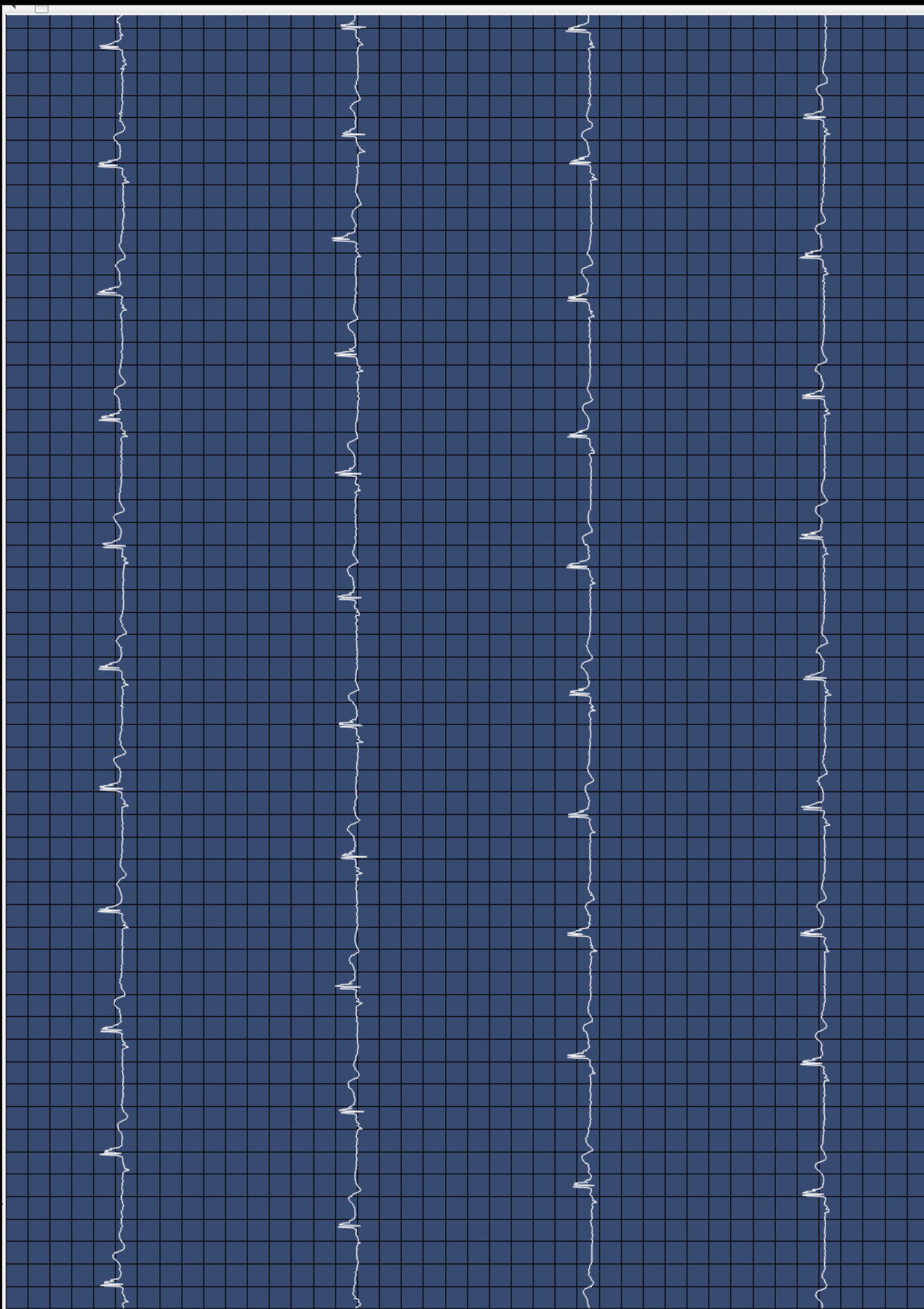

Supplement: Supplementary file 2 [file Data_Sheet_2.zip › EKG blindede/Subject 4 rest + max apnoea/4 rest V2.pdf]

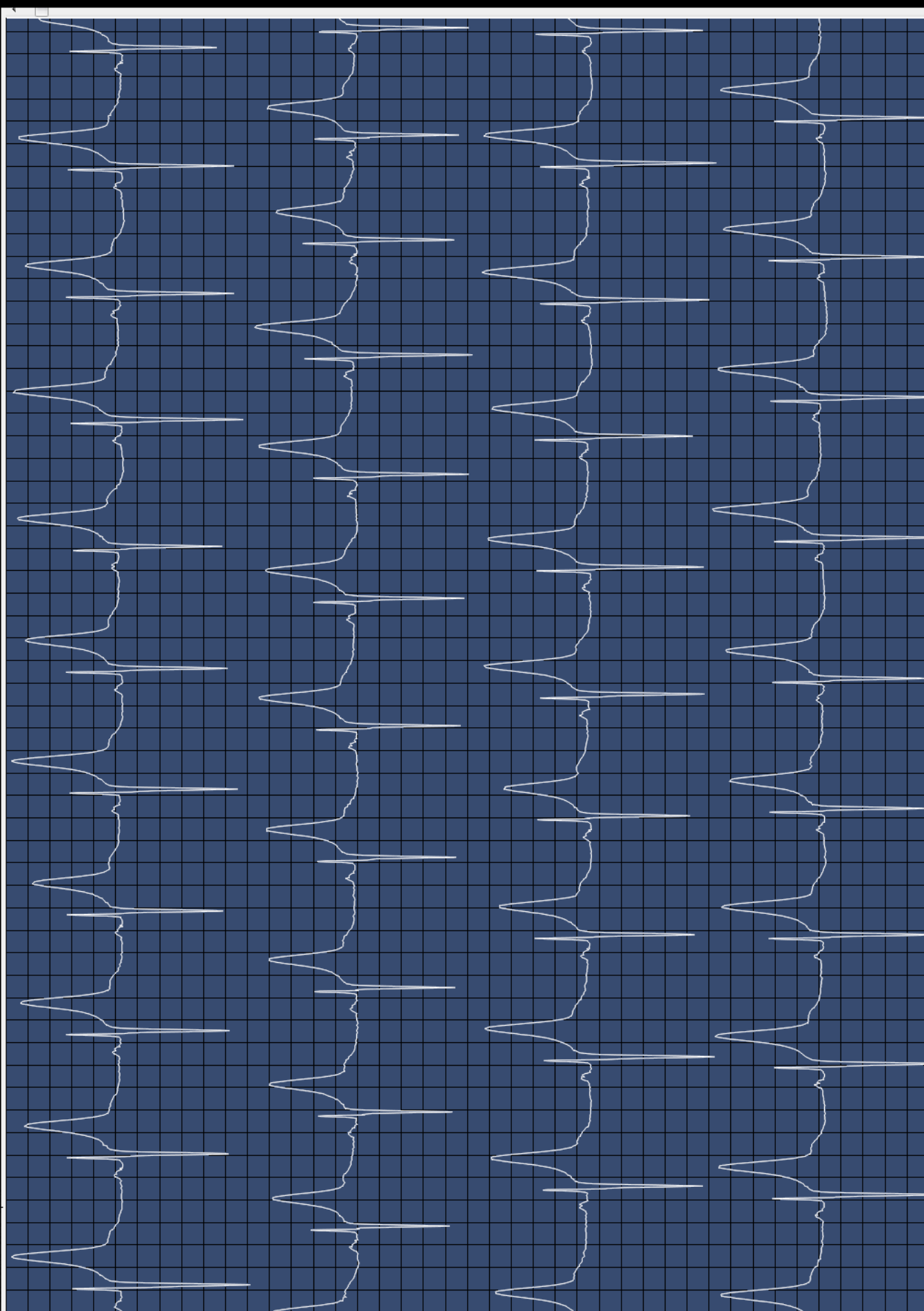

Supplement: Supplementary file 2 [file Data_Sheet_2.zip › EKG blindede/Subject 4 rest + max apnoea/4 rest V3.pdf]

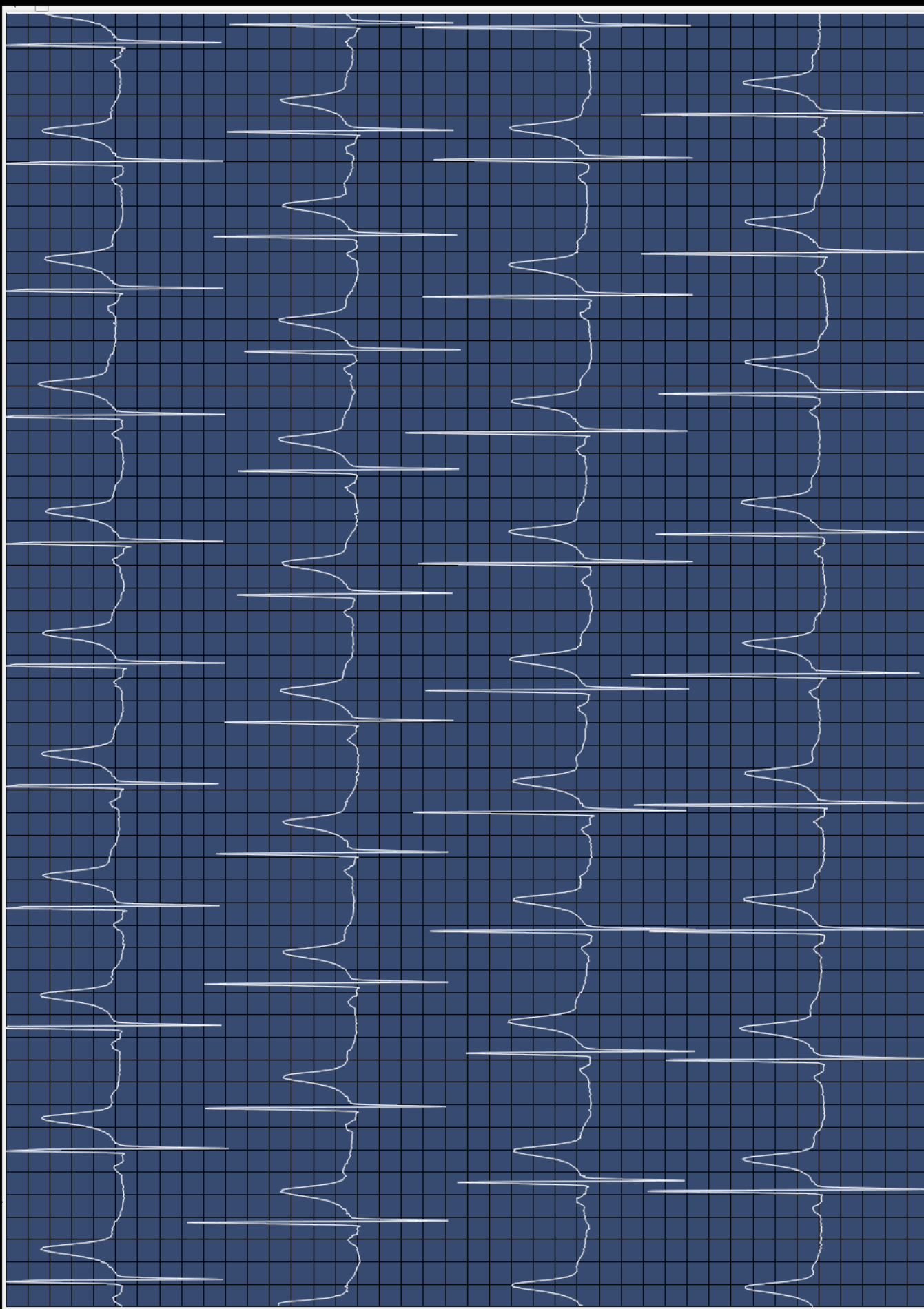

Supplement: Supplementary file 2 [file Data_Sheet_2.zip › EKG blindede/Subject 4 rest + max apnoea/4 rest V4.pdf]

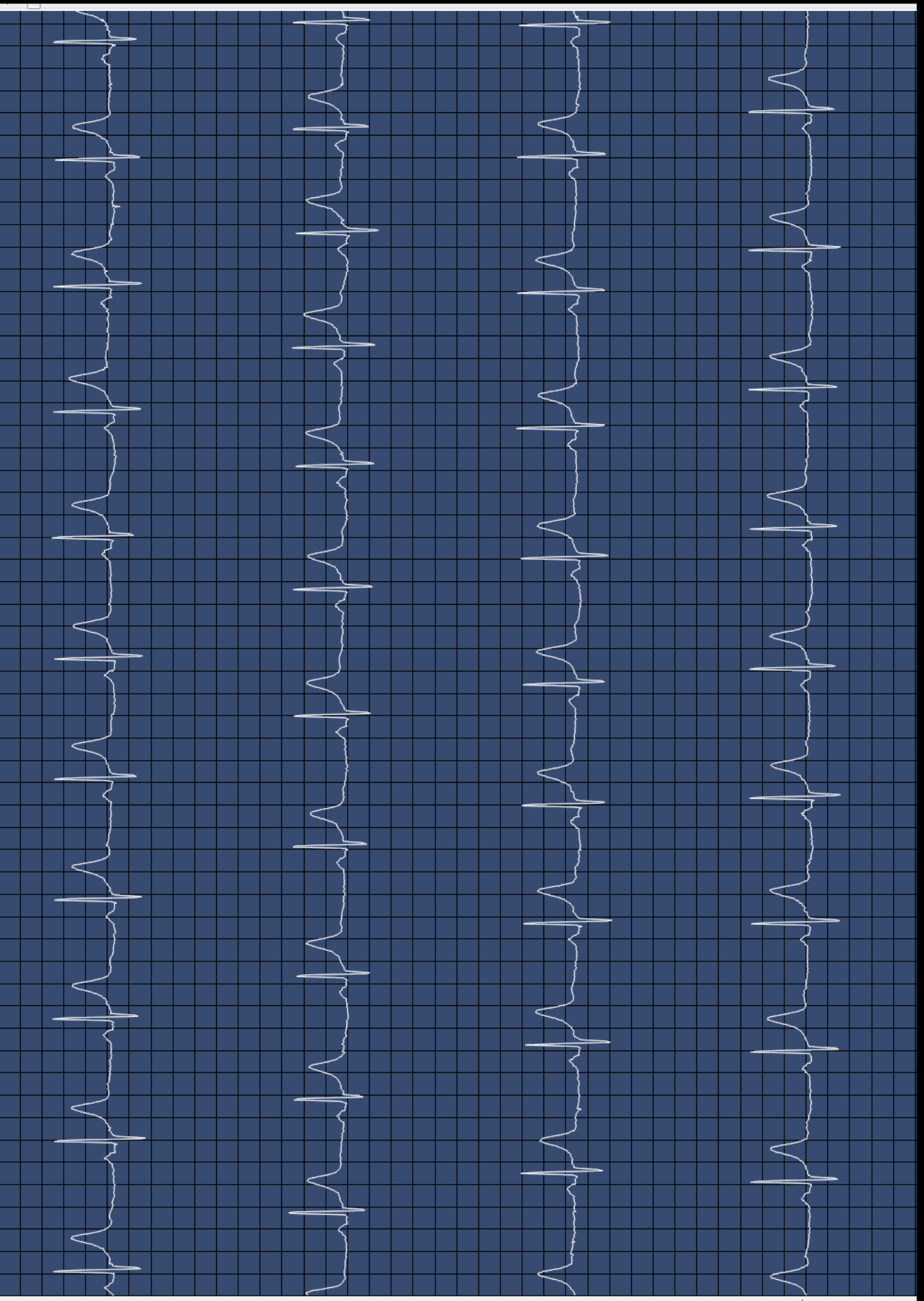

Supplement: Supplementary file 2 [file Data_Sheet_2.zip › EKG blindede/Subject 4 rest + max apnoea/4 rest V6.pdf]

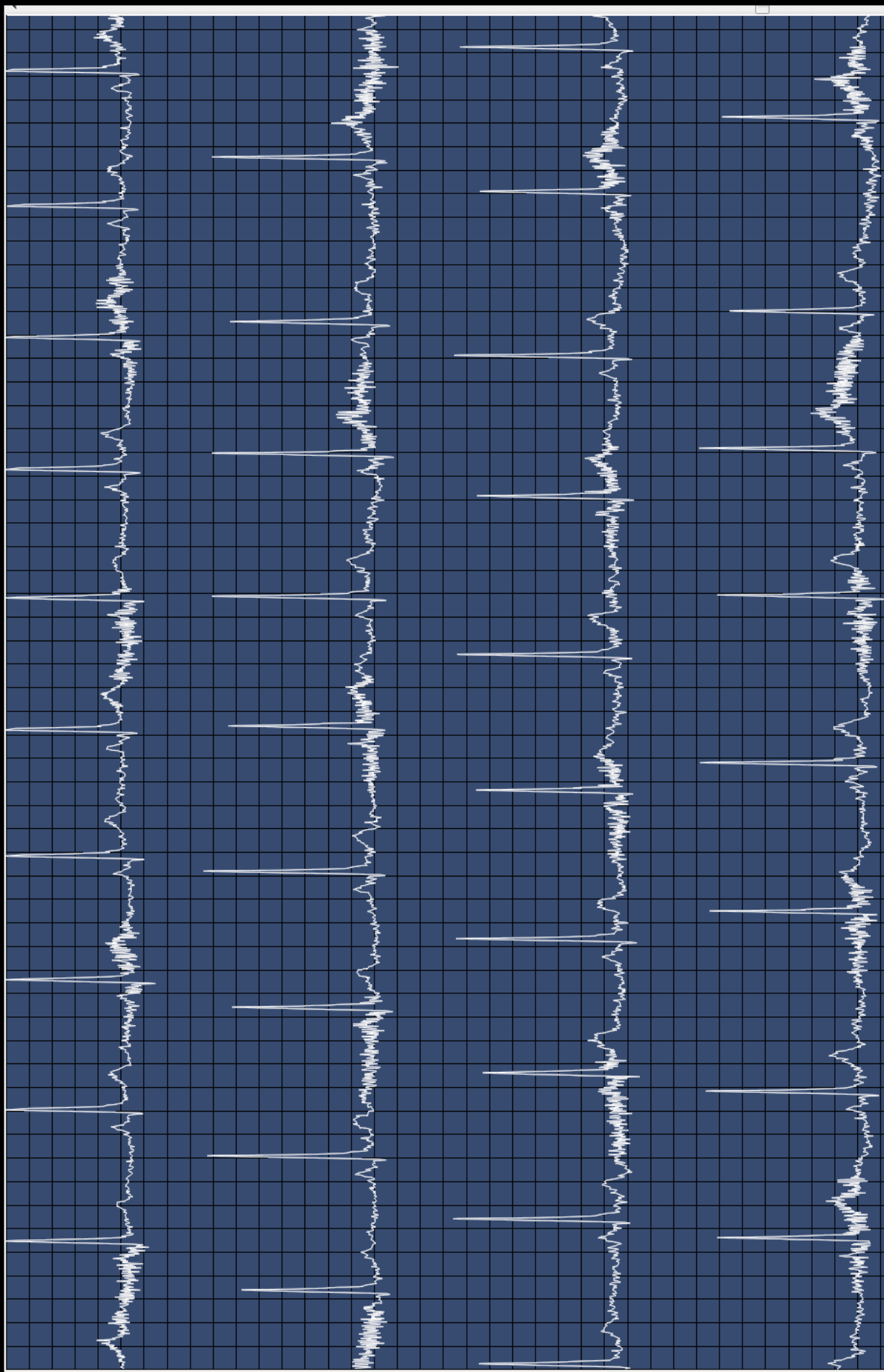

Supplement: Supplementary file 2 [file Data_Sheet_2.zip › EKG blindede/Subject 5 rest + max apnoea/5 max apnoea aVF.pdf]

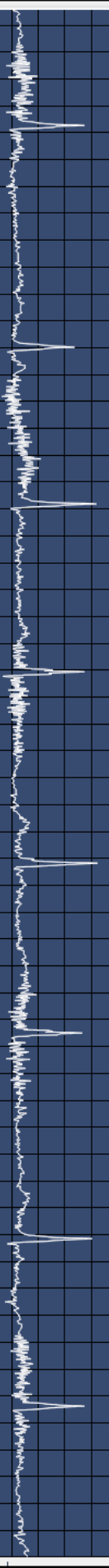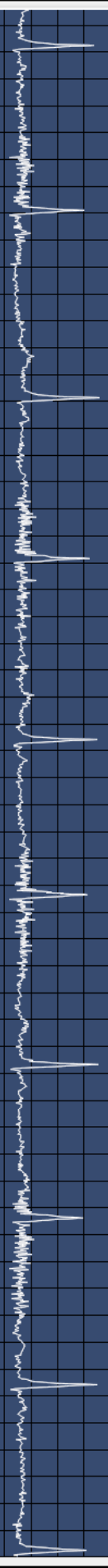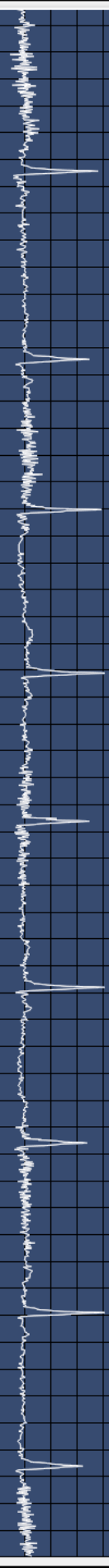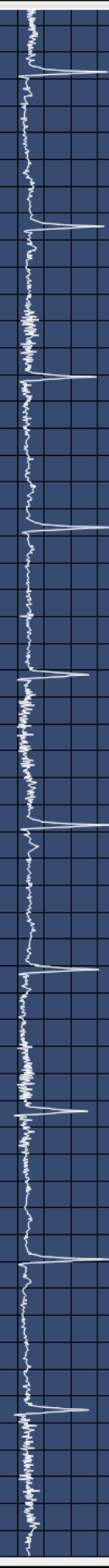

Supplement: Supplementary file 2 [file Data_Sheet_2.zip › EKG blindede/Subject 5 rest + max apnoea/5 max apnoea aVL.pdf]
