# Supplementary material for: Extreme Hypoxia Causing Brady-Arrythmias During Apnea in Elite Breath-Hold Divers
Source: Front Physiol. 2021 Dec 3;12:712573. doi: 10.3389/fphys.2021.712573 (PMC8678416; doi:10.3389/fphys.2021.712573)
Supplement: Supplementary file 2 [file Data_Sheet_2.zip › EKG blindede/Subject 1 rest + max apnoea/1 rest V2.pdf]

GE CardioSoft (Supervisor) : [redacted] Hvile-EKG / 26.08.2020 - 18:03:01

Testoversigt | Full-disclosure EKG

V2 | L 00:10 | 25mm/s | 20mm/mV | 4 Linjer | ☑ Arytmi i farver

0.01-150Hz 50Hz Spline

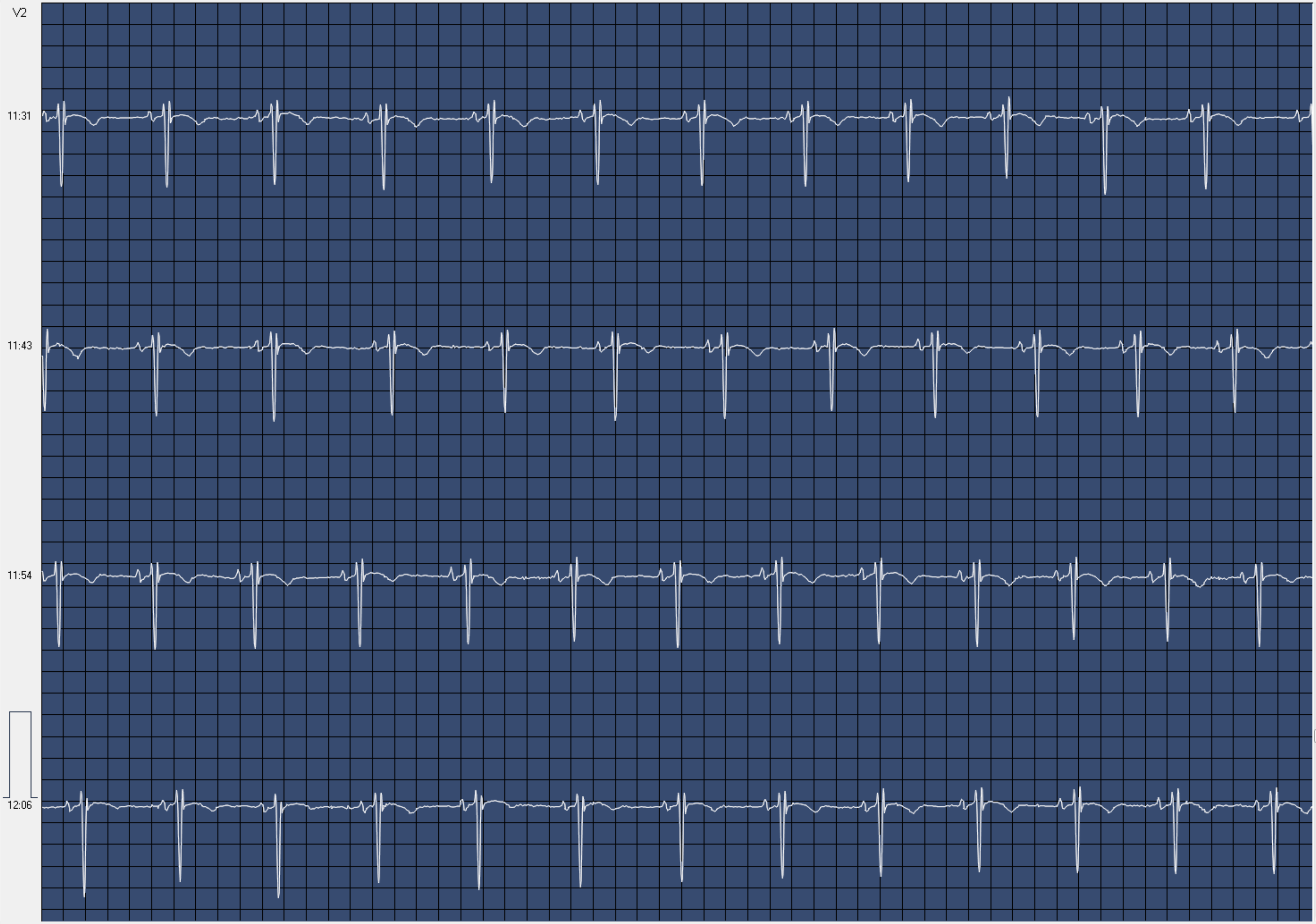

Ny test
Lokal database
MUSE browser
Udskriv
Sammenlign
Tolkning
Hjælp
Startskærm
