# Supplementary material for: Extreme Hypoxia Causing Brady-Arrythmias During Apnea in Elite Breath-Hold Divers
Source: Front Physiol. 2021 Dec 3;12:712573. doi: 10.3389/fphys.2021.712573 (PMC8678416; doi:10.3389/fphys.2021.712573)
Supplement: Supplementary file 2 [file Data_Sheet_2.zip › EKG blindede/Subject 4 rest + max apnoea/4 rest V5.pdf]

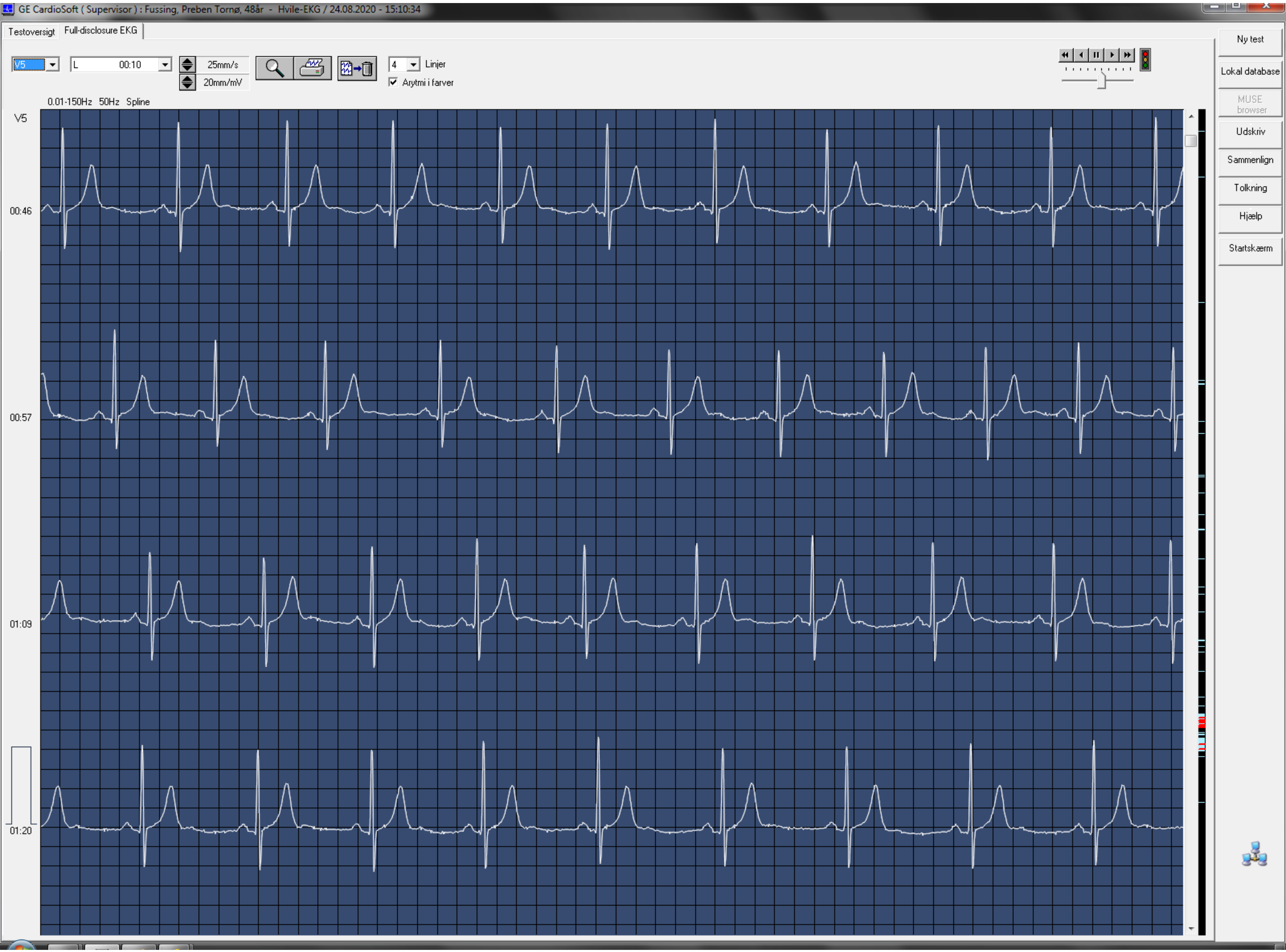

GE CardioSoft (Supervisor) : Fussing, Preben Tornø, 48år - Hvile-EKG / 24.08.2020 - 15:10:34
Testoversigt
Full-disclosure EKG
V5
L
00:10
25mm/s
20mm/mV
4
Linjer
Arytmi i farver
0.01-150Hz 50Hz Spline
V5
00:46
00:57
01:09
01:20
Ny test
Lokal database
MUSE browser
Udskriv
Sammenlign
Tolkning
Hjælp
Startskærm
