# Supplementary figures and images for: Extreme Hypoxia Causing Brady-Arrythmias During Apnea in Elite Breath-Hold Divers (part 2 of 2)
Source: Front Physiol. 2021 Dec 3;12:712573. doi: 10.3389/fphys.2021.712573 (PMC8678416; doi:10.3389/fphys.2021.712573)

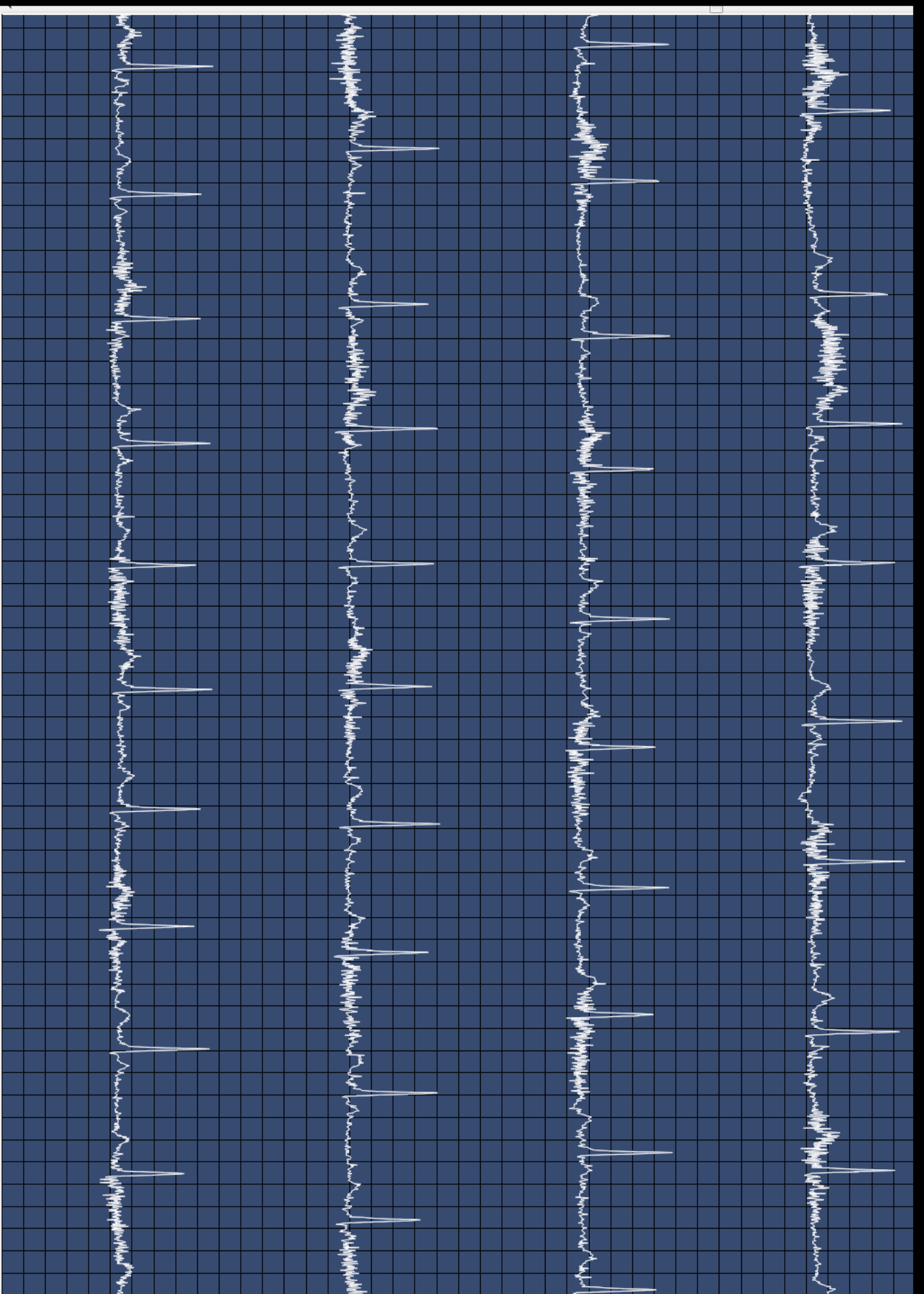

Supplement: Supplementary file 2 [file Data_Sheet_2.zip › EKG blindede/Subject 5 rest + max apnoea/5 max apnoea aVR.pdf]

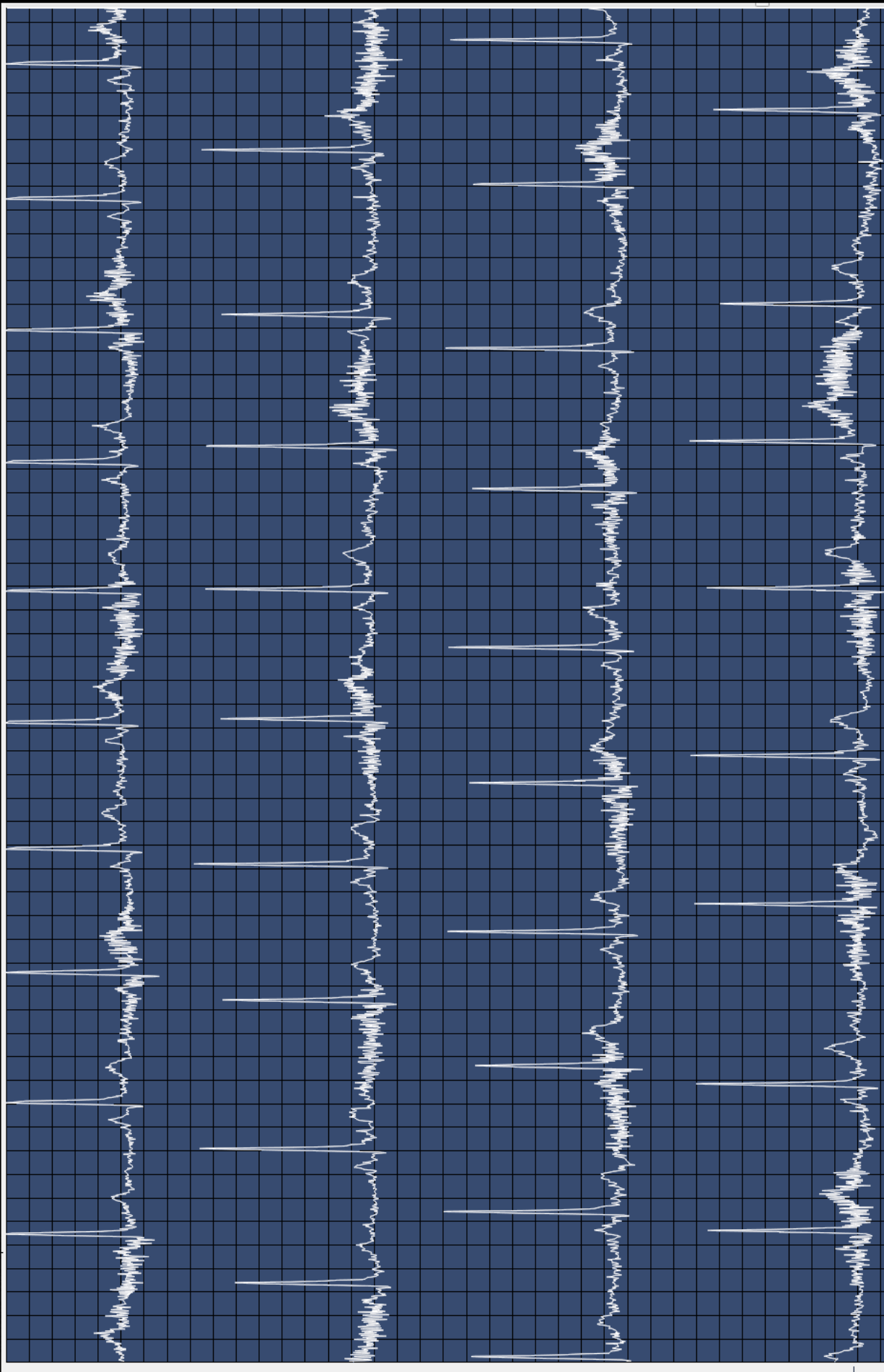

Supplement: Supplementary file 2 [file Data_Sheet_2.zip › EKG blindede/Subject 5 rest + max apnoea/5 max apnoea II.pdf]

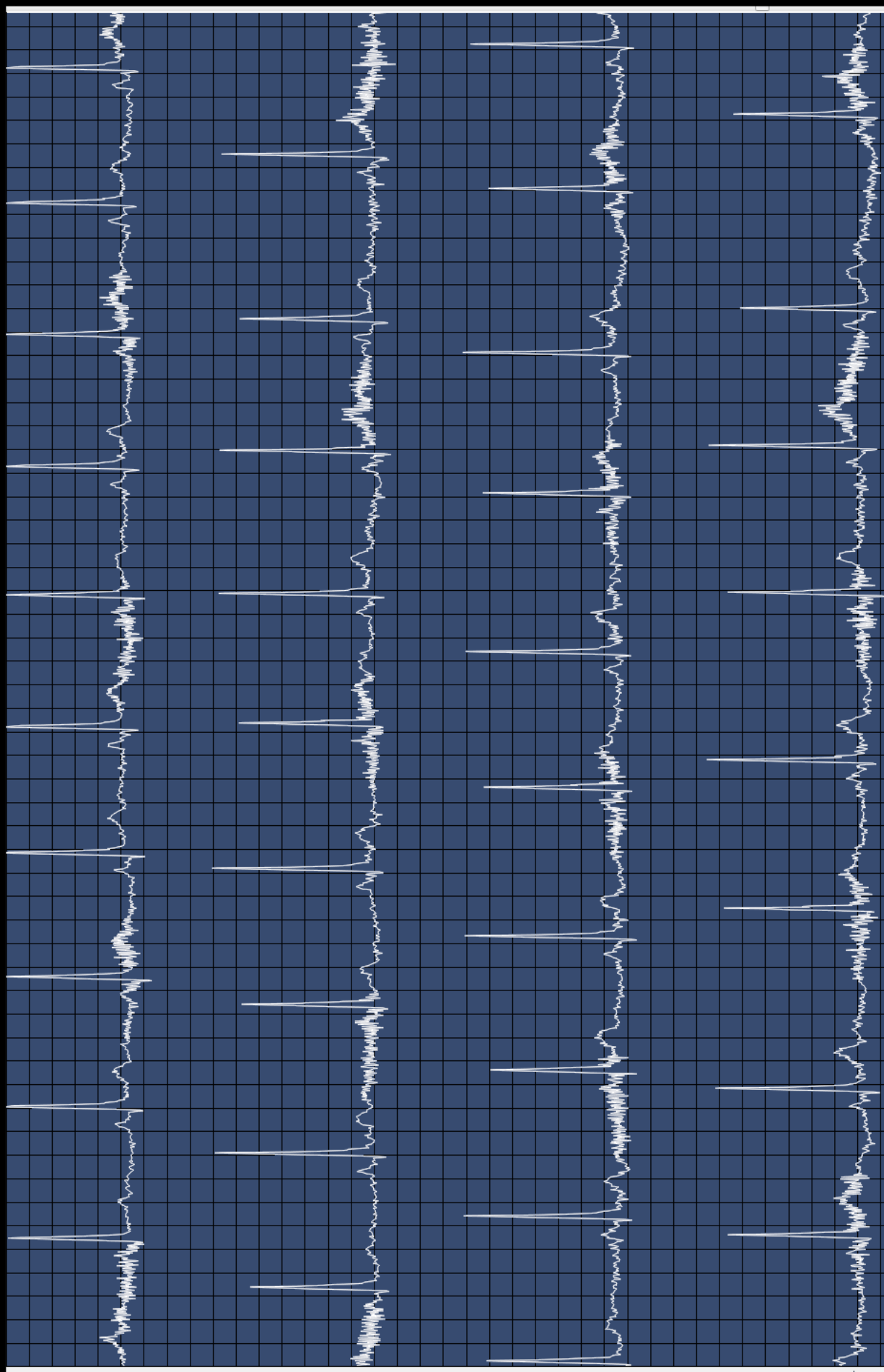

Supplement: Supplementary file 2 [file Data_Sheet_2.zip › EKG blindede/Subject 5 rest + max apnoea/5 max apnoea III.pdf]

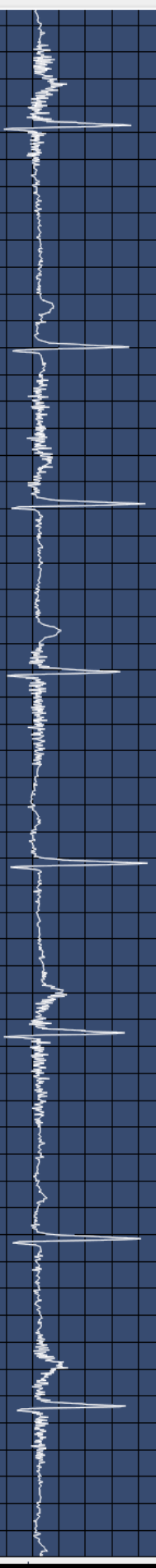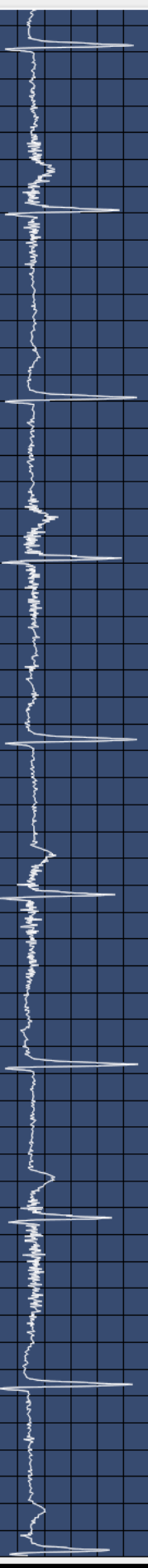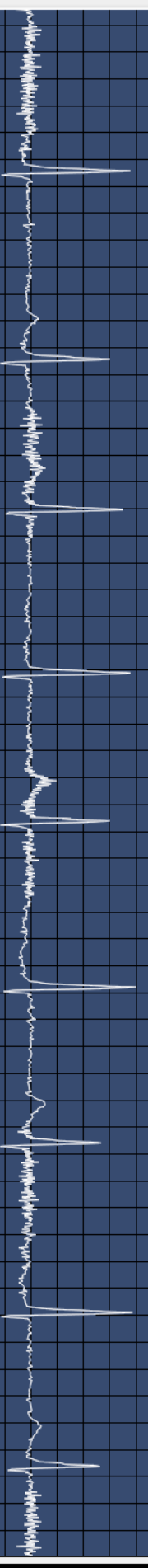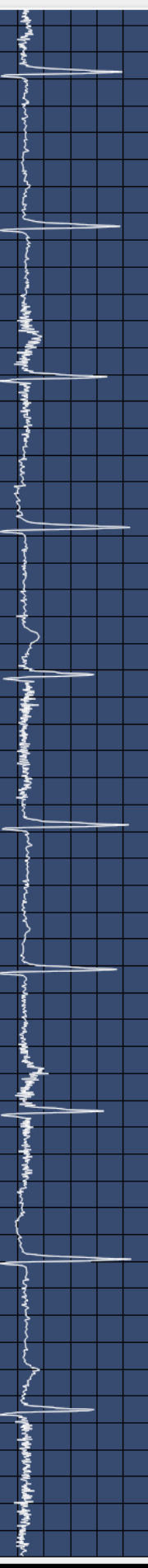

Supplement: Supplementary file 2 [file Data_Sheet_2.zip › EKG blindede/Subject 5 rest + max apnoea/5 max apnoea V1.pdf]

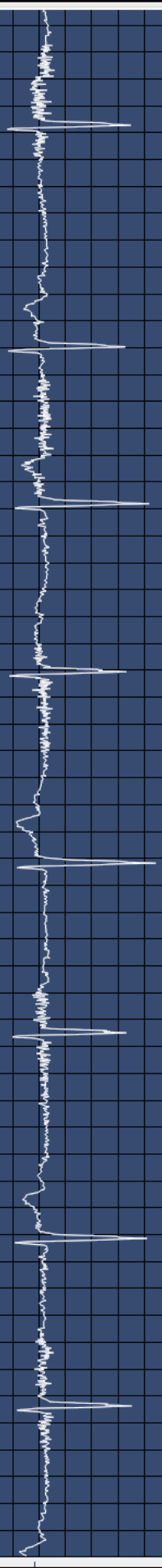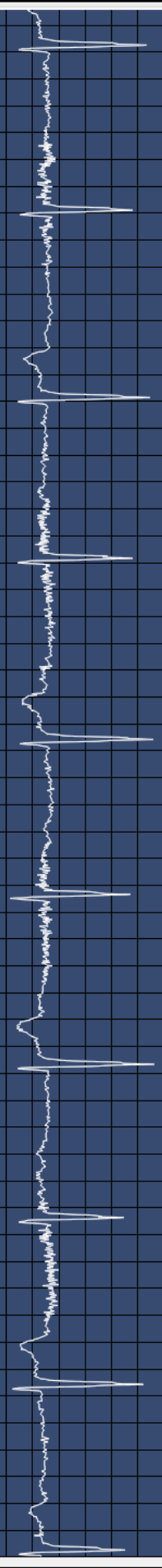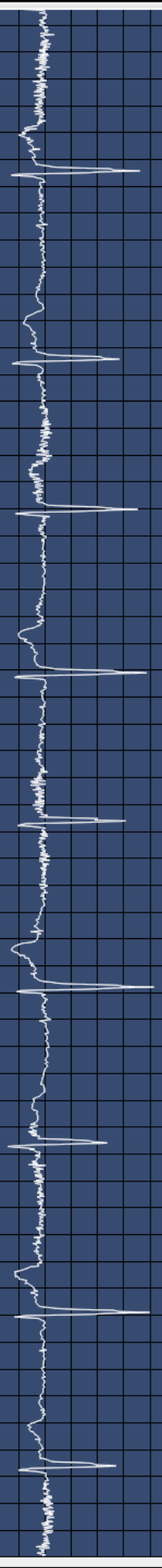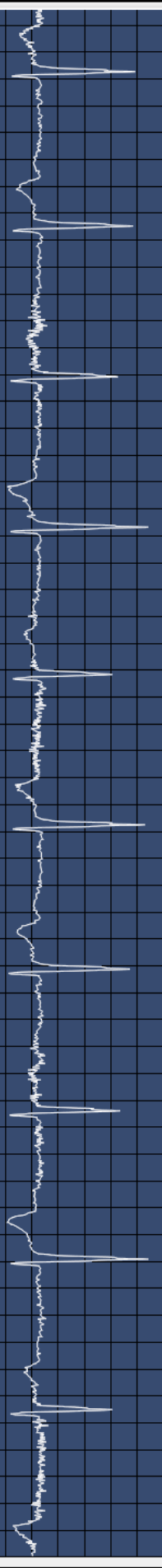

Supplement: Supplementary file 2 [file Data_Sheet_2.zip › EKG blindede/Subject 5 rest + max apnoea/5 max apnoea V2.pdf]

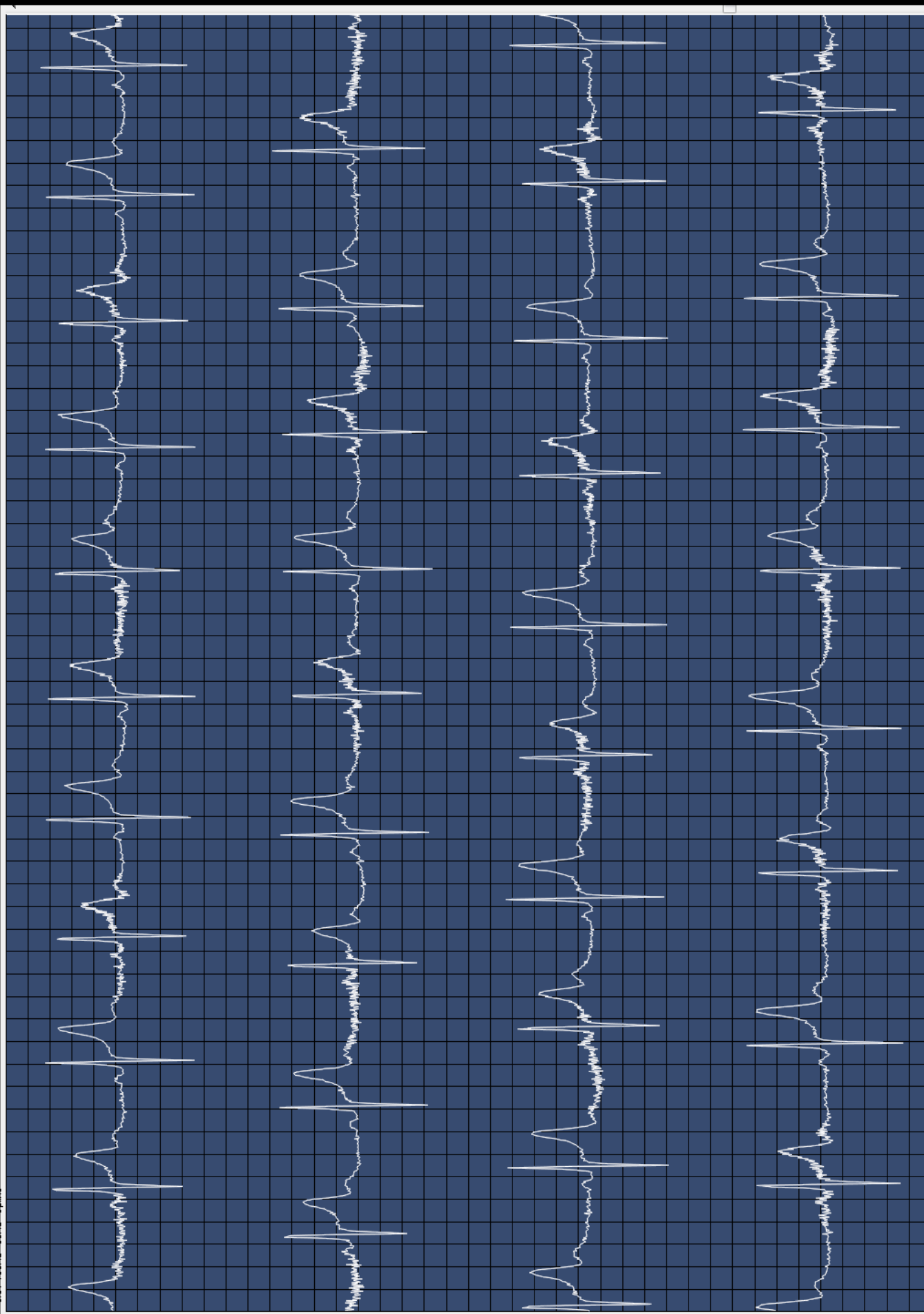

Supplement: Supplementary file 2 [file Data_Sheet_2.zip › EKG blindede/Subject 5 rest + max apnoea/5 max apnoea V3.pdf]

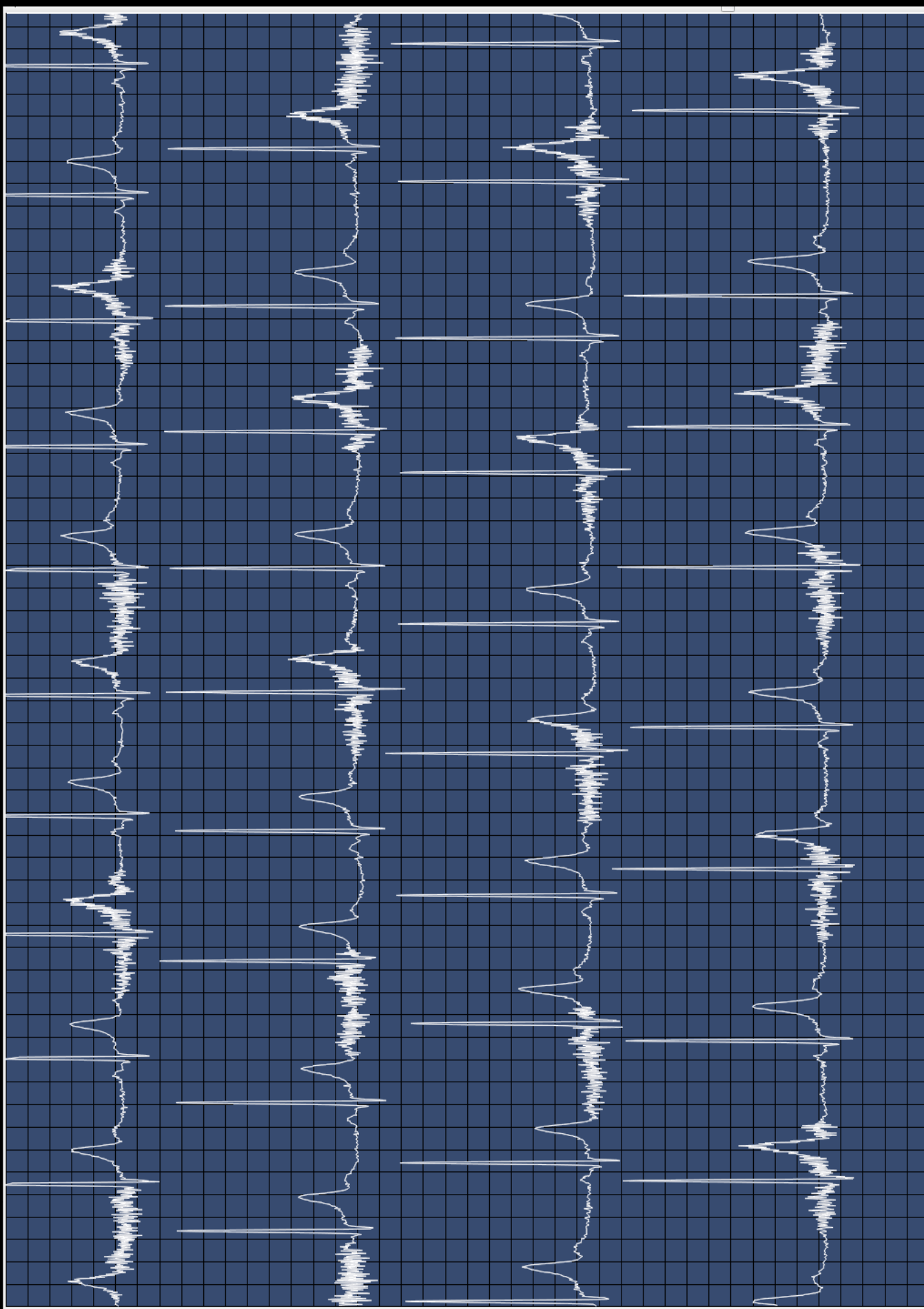

Supplement: Supplementary file 2 [file Data_Sheet_2.zip › EKG blindede/Subject 5 rest + max apnoea/5 max apnoea V4.pdf]

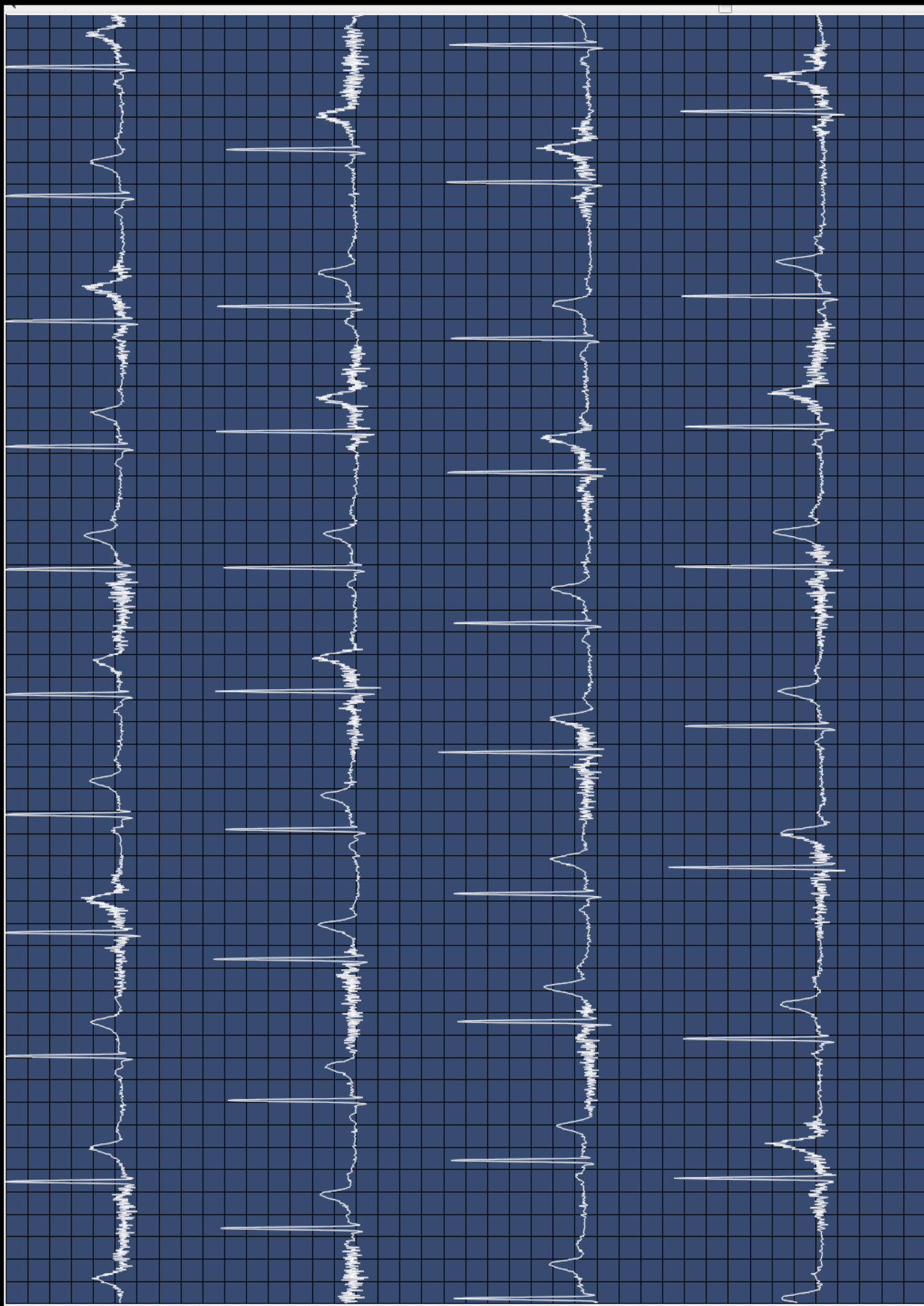

Supplement: Supplementary file 2 [file Data_Sheet_2.zip › EKG blindede/Subject 5 rest + max apnoea/5 max apnoea V5.pdf]

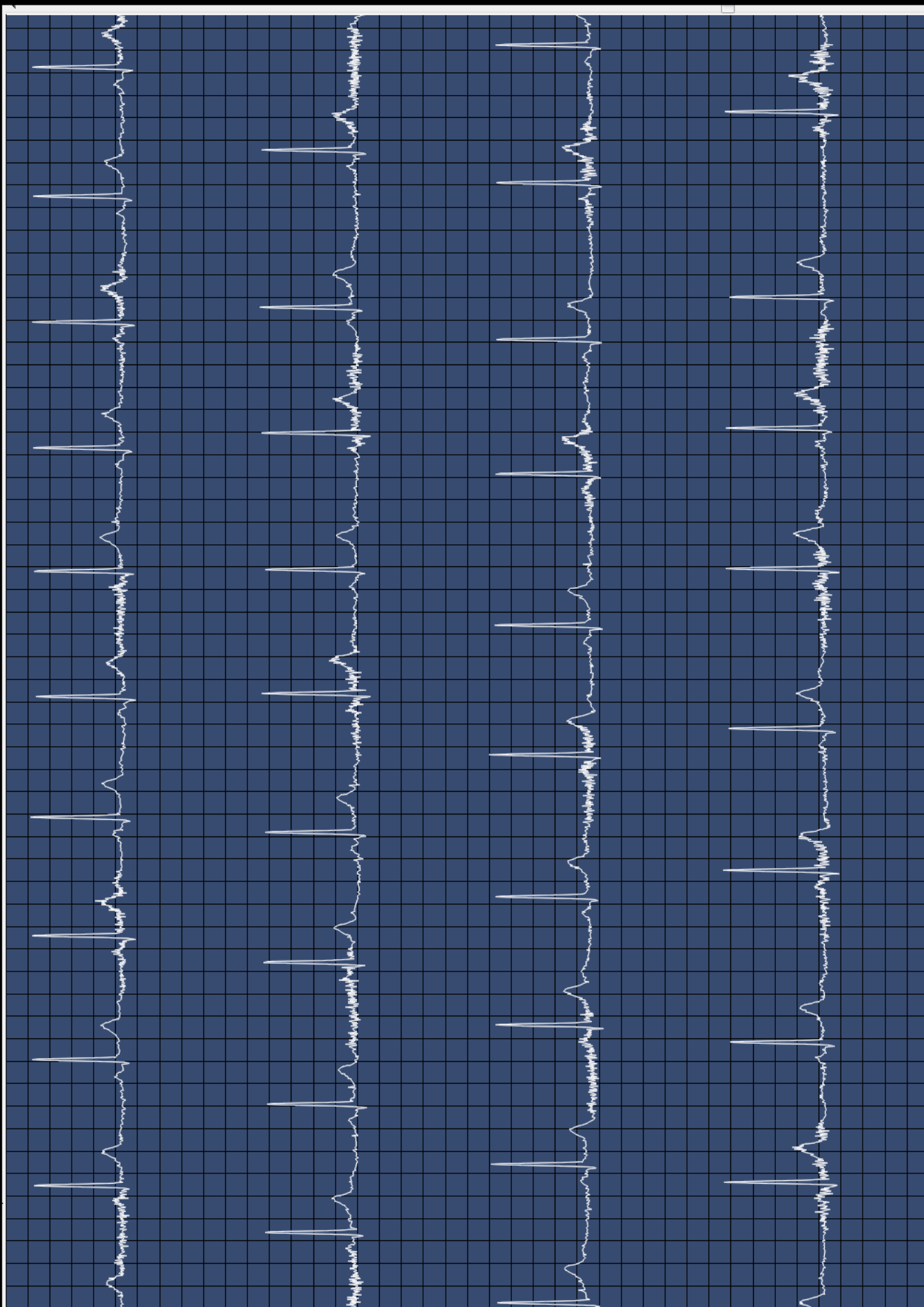

Supplement: Supplementary file 2 [file Data_Sheet_2.zip › EKG blindede/Subject 5 rest + max apnoea/5 max apnoea V6.pdf]

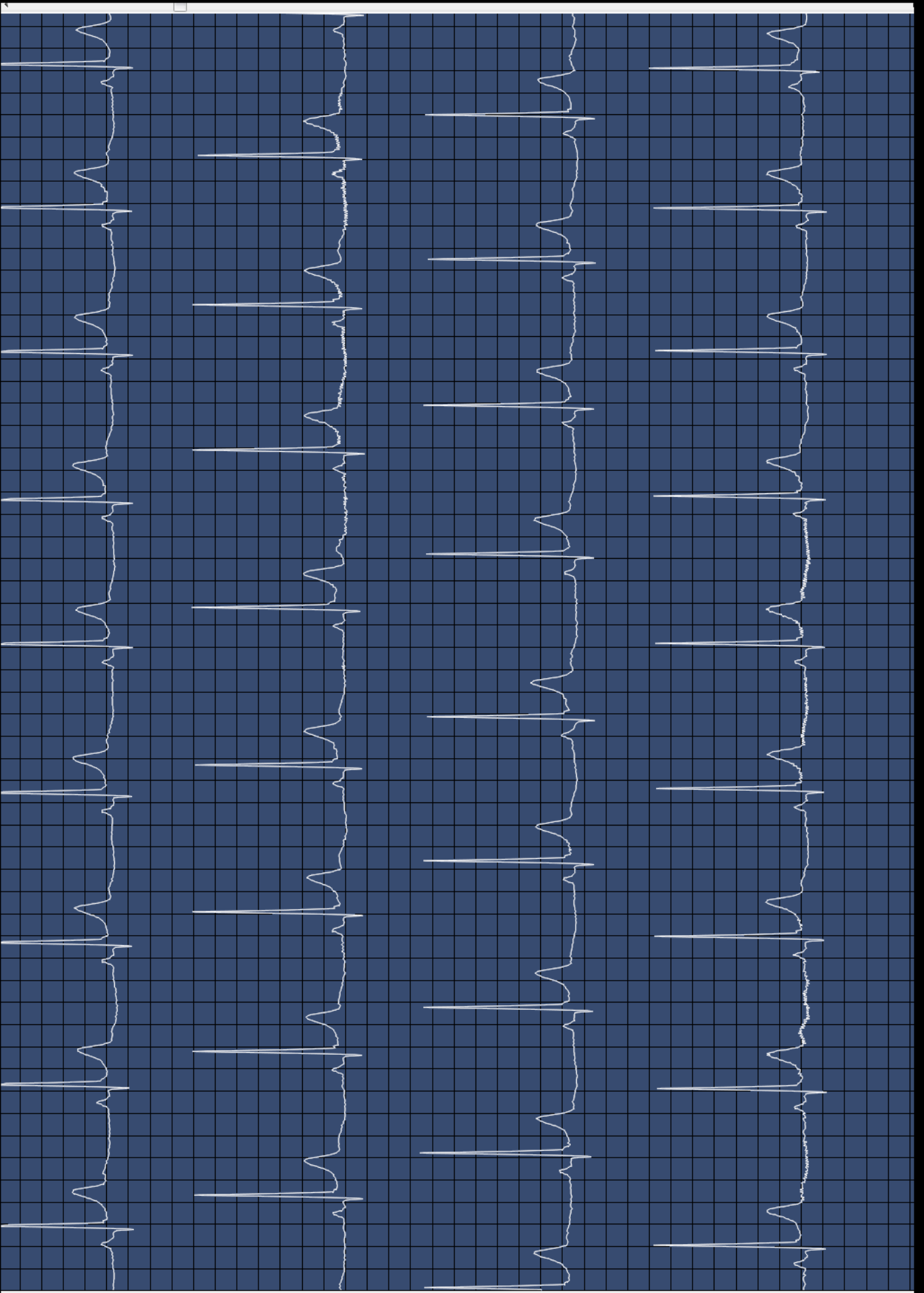

Supplement: Supplementary file 2 [file Data_Sheet_2.zip › EKG blindede/Subject 5 rest + max apnoea/5 rest aVF.pdf]

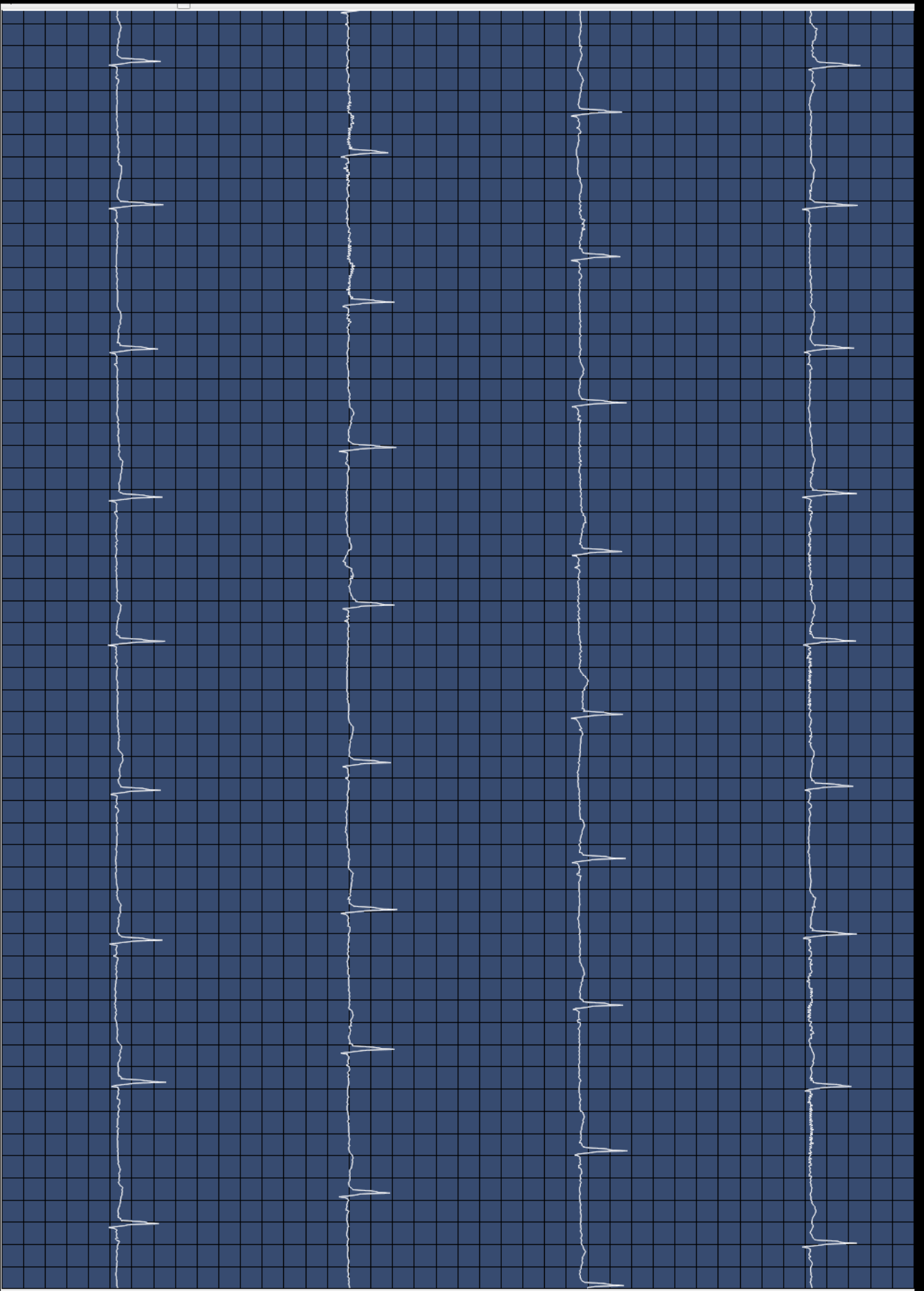

Supplement: Supplementary file 2 [file Data_Sheet_2.zip › EKG blindede/Subject 5 rest + max apnoea/5 rest aVL.pdf]

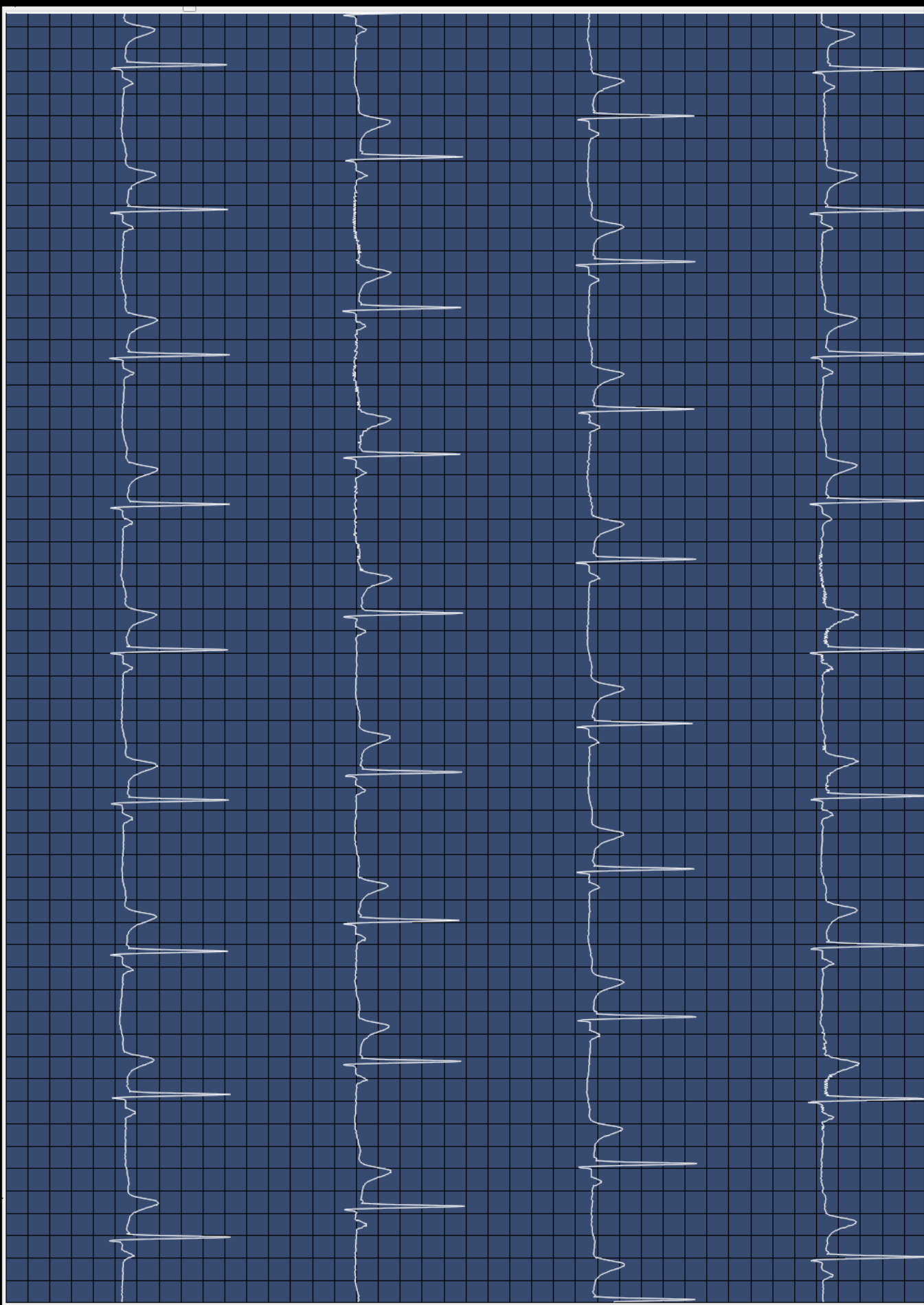

Supplement: Supplementary file 2 [file Data_Sheet_2.zip › EKG blindede/Subject 5 rest + max apnoea/5 rest aVR.pdf]

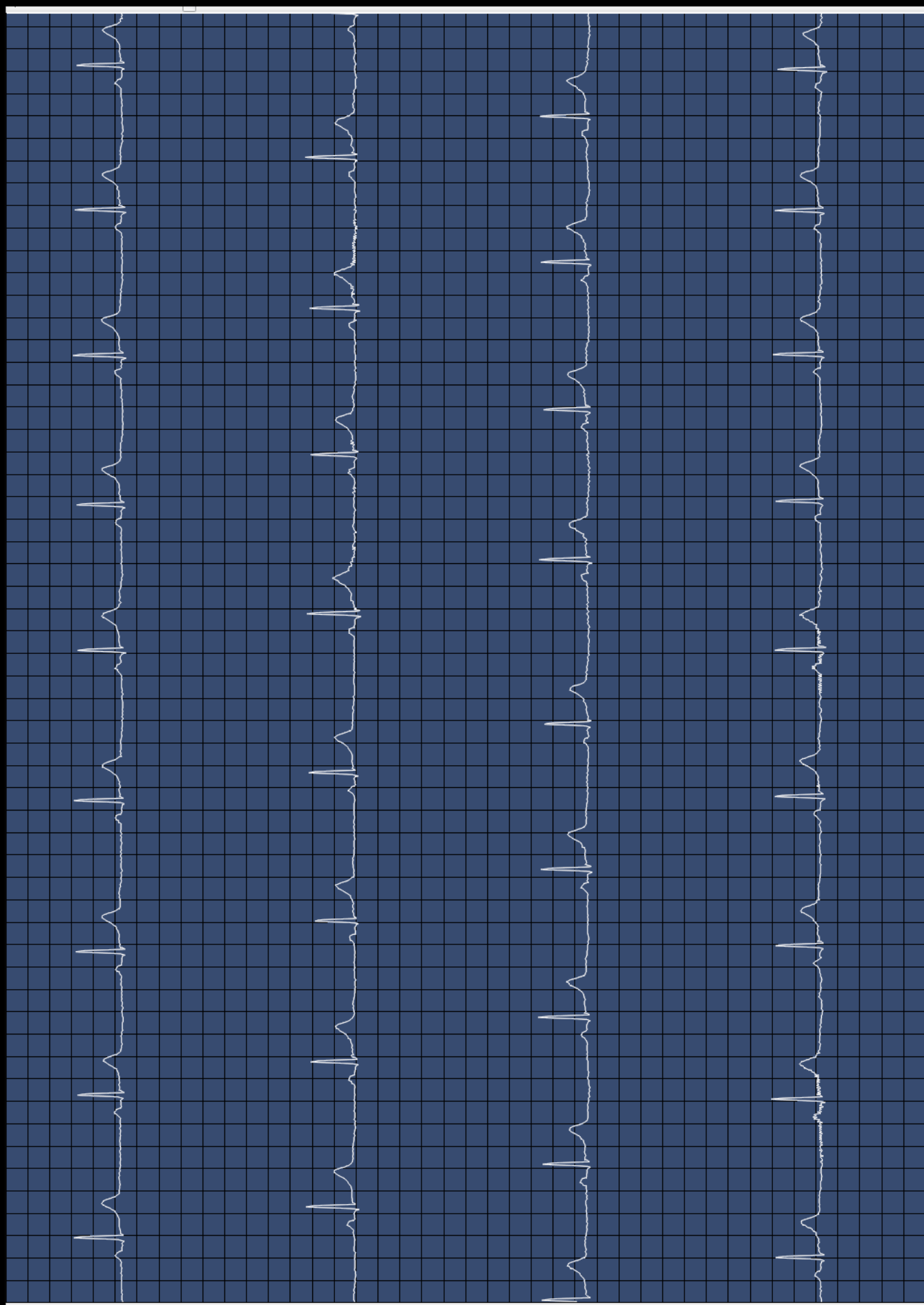

Supplement: Supplementary file 2 [file Data_Sheet_2.zip › EKG blindede/Subject 5 rest + max apnoea/5 rest I.pdf]

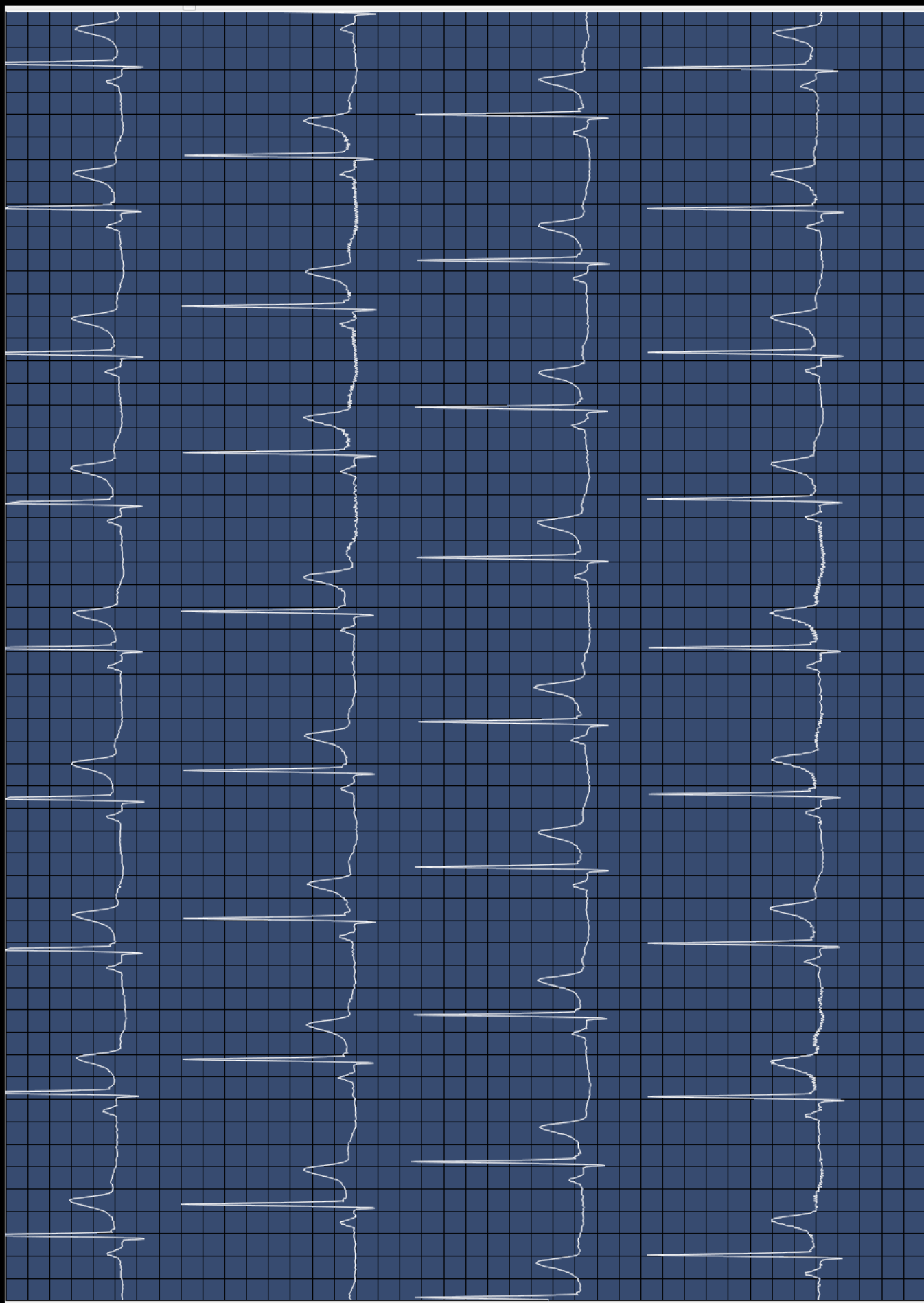

Supplement: Supplementary file 2 [file Data_Sheet_2.zip › EKG blindede/Subject 5 rest + max apnoea/5 rest II.pdf]

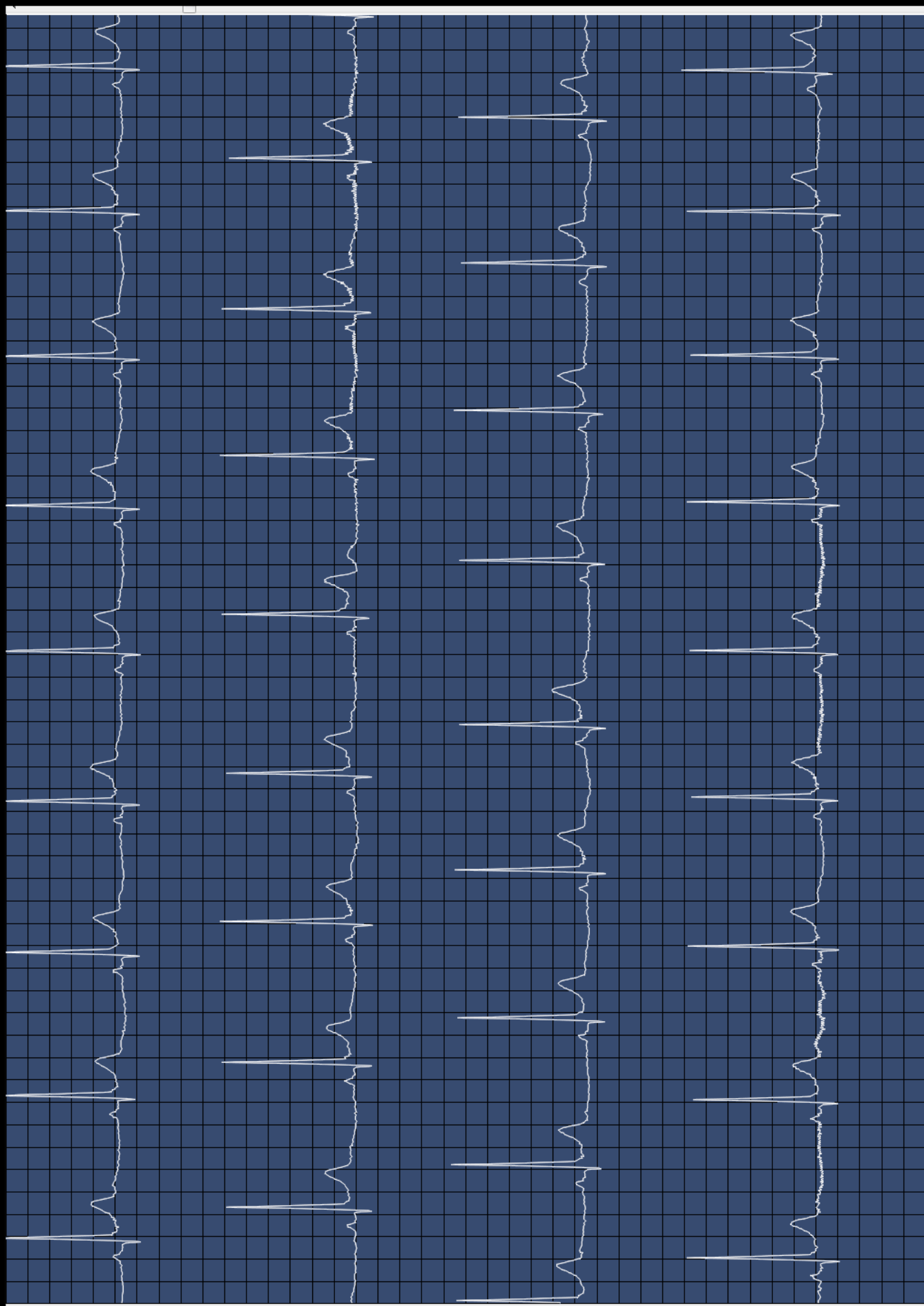

Supplement: Supplementary file 2 [file Data_Sheet_2.zip › EKG blindede/Subject 5 rest + max apnoea/5 rest III.pdf]

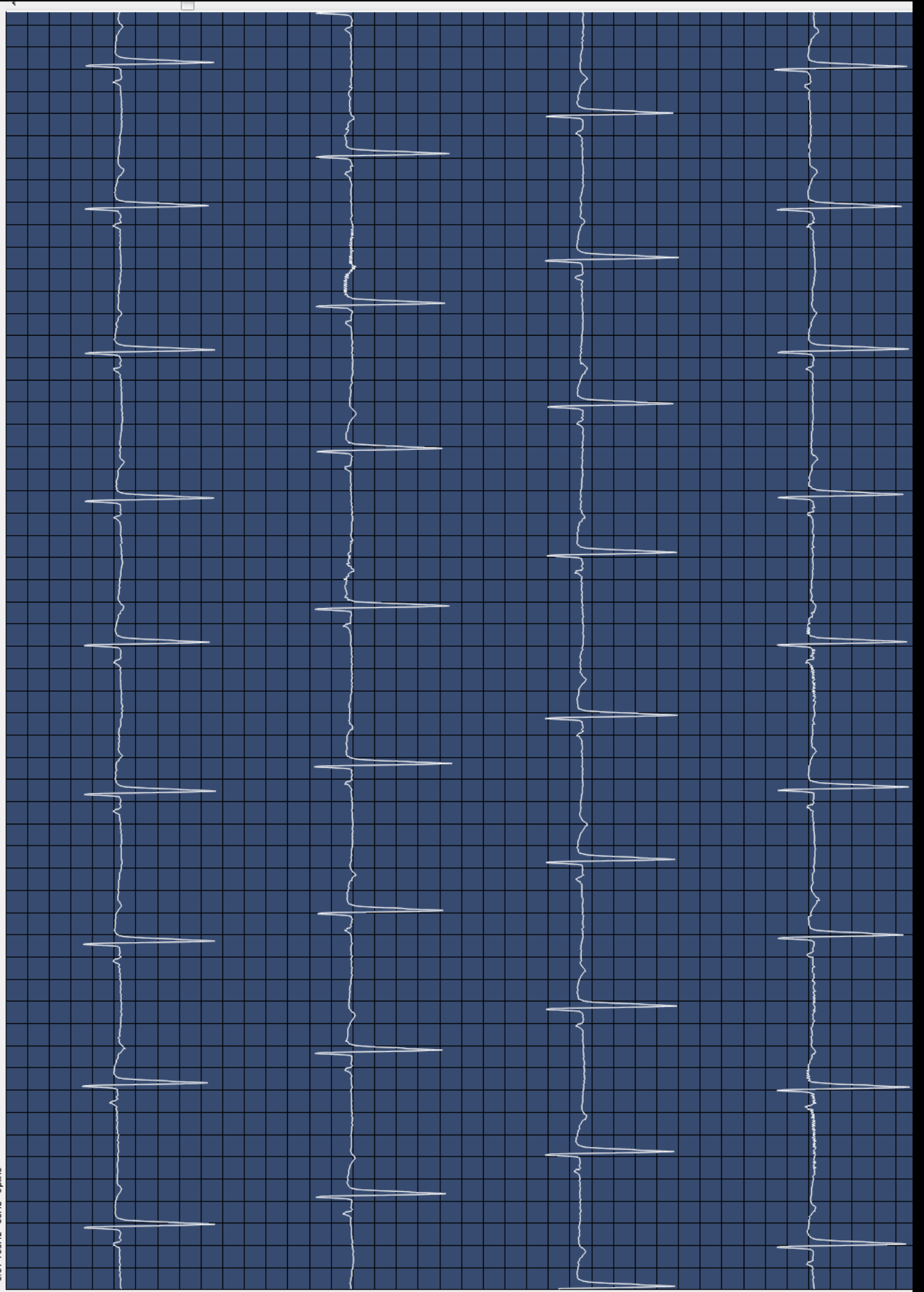

Supplement: Supplementary file 2 [file Data_Sheet_2.zip › EKG blindede/Subject 5 rest + max apnoea/5 rest V1.pdf]

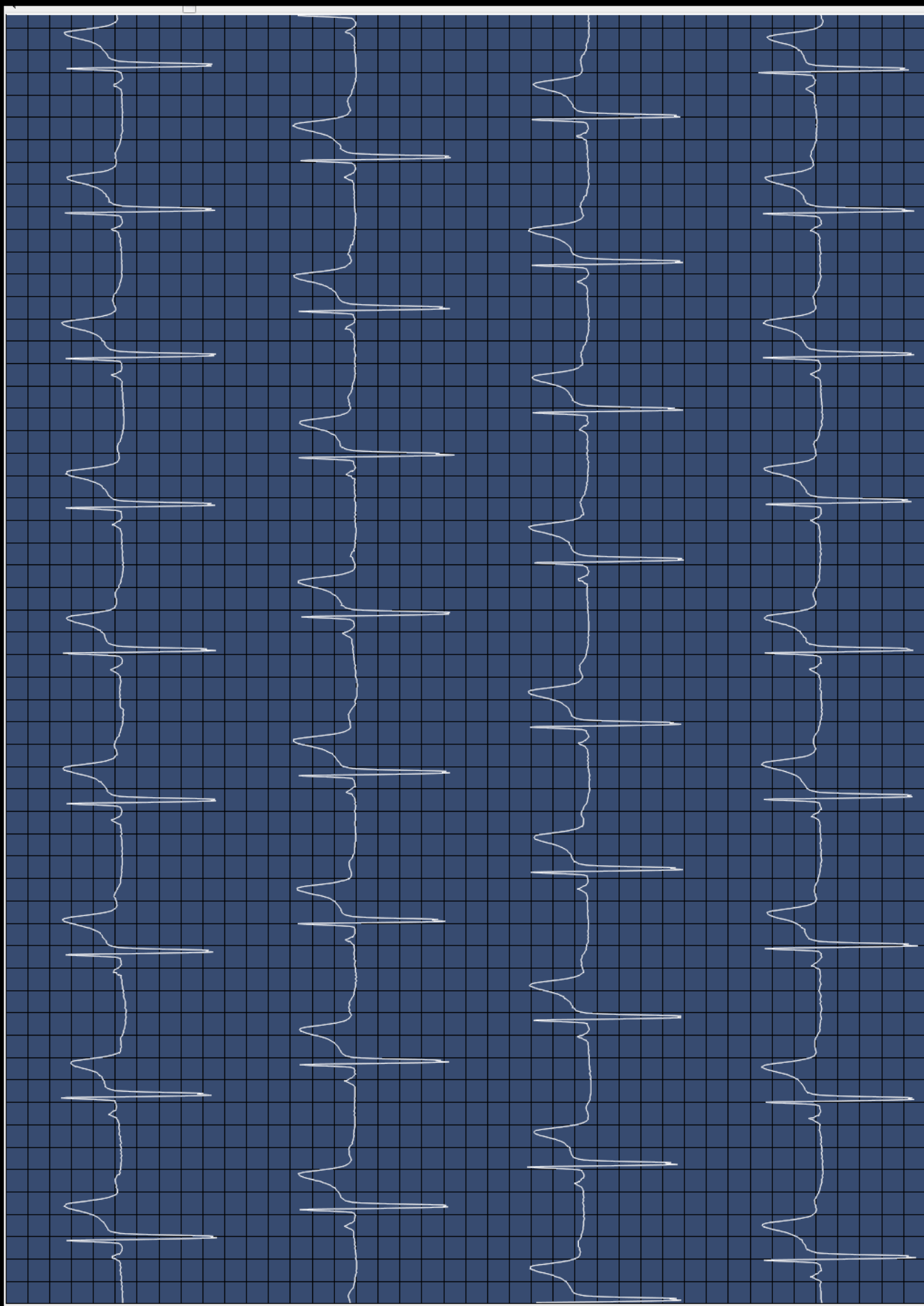

Supplement: Supplementary file 2 [file Data_Sheet_2.zip › EKG blindede/Subject 5 rest + max apnoea/5 rest V2.pdf]

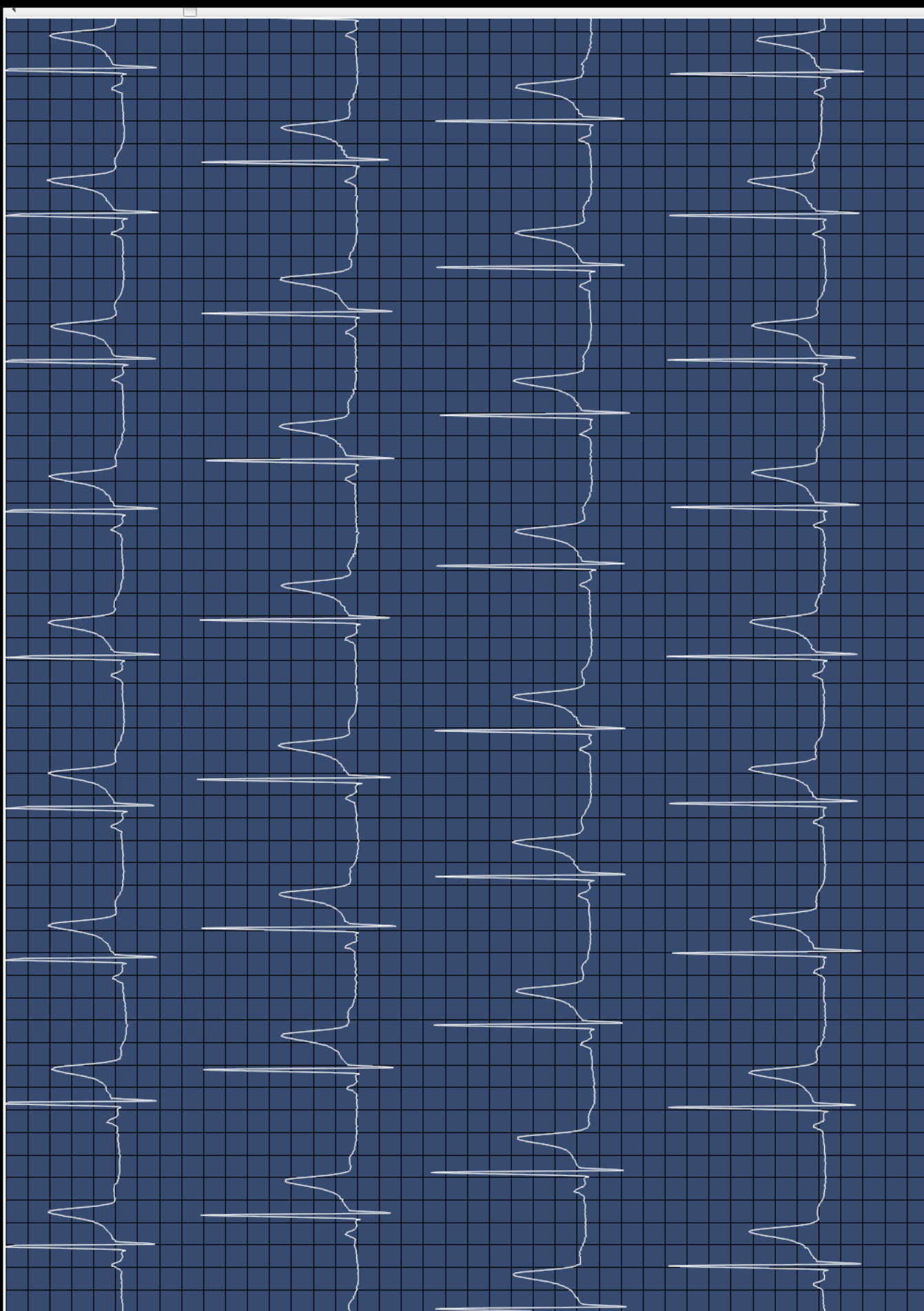

Supplement: Supplementary file 2 [file Data_Sheet_2.zip › EKG blindede/Subject 5 rest + max apnoea/5 rest V3.pdf]

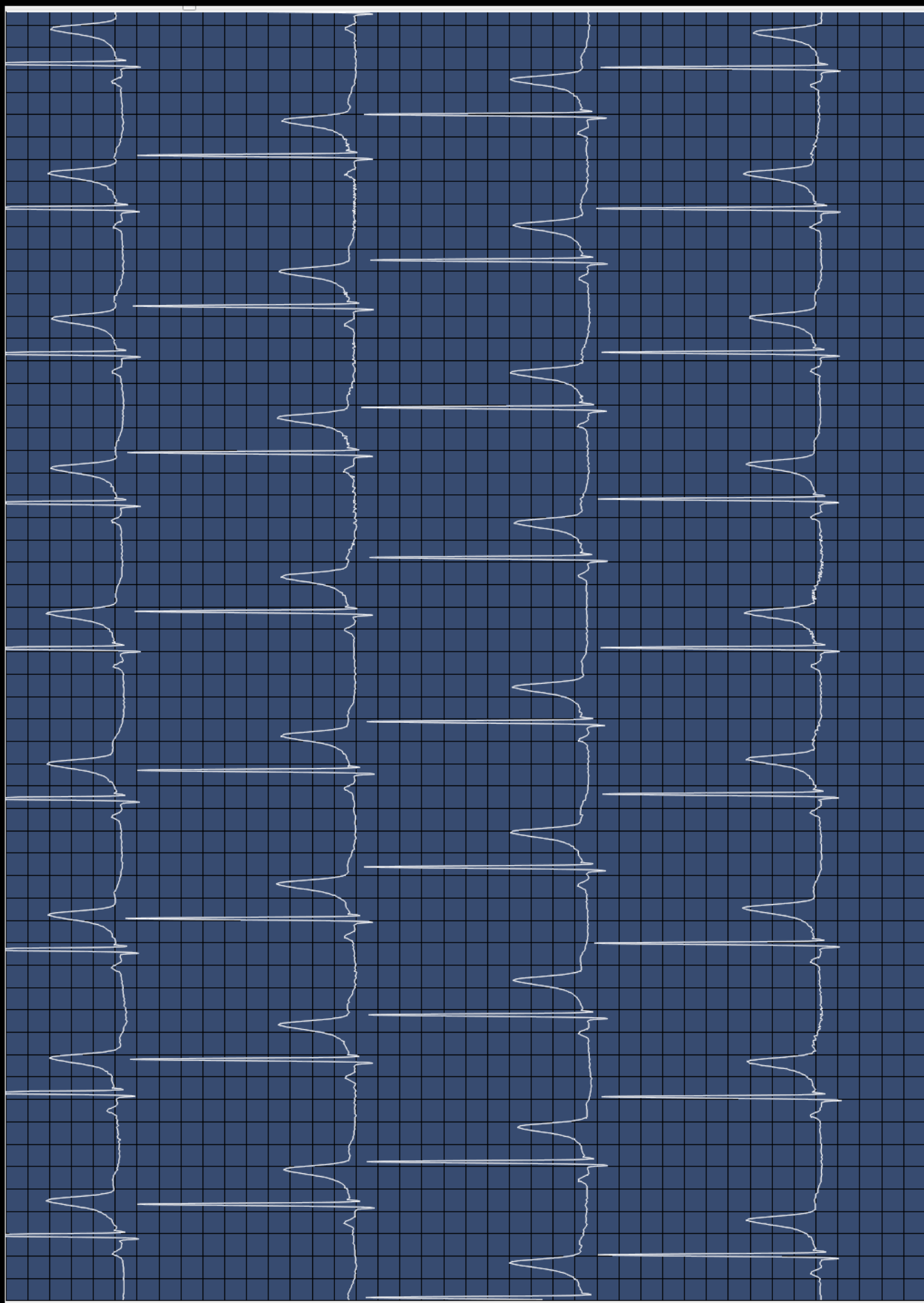

Supplement: Supplementary file 2 [file Data_Sheet_2.zip › EKG blindede/Subject 5 rest + max apnoea/5 rest V4.pdf]

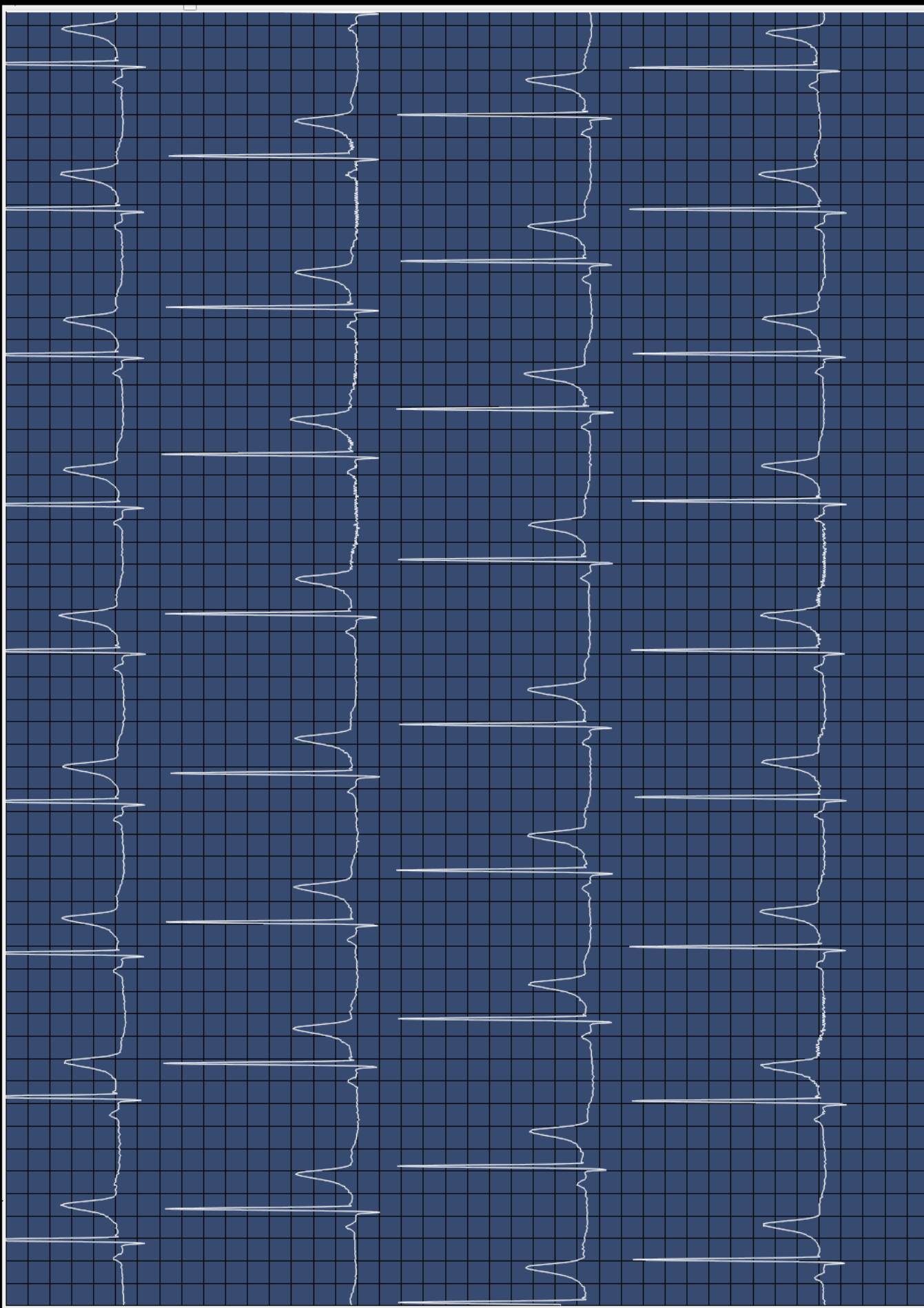

Supplement: Supplementary file 2 [file Data_Sheet_2.zip › EKG blindede/Subject 5 rest + max apnoea/5 rest V5.pdf]

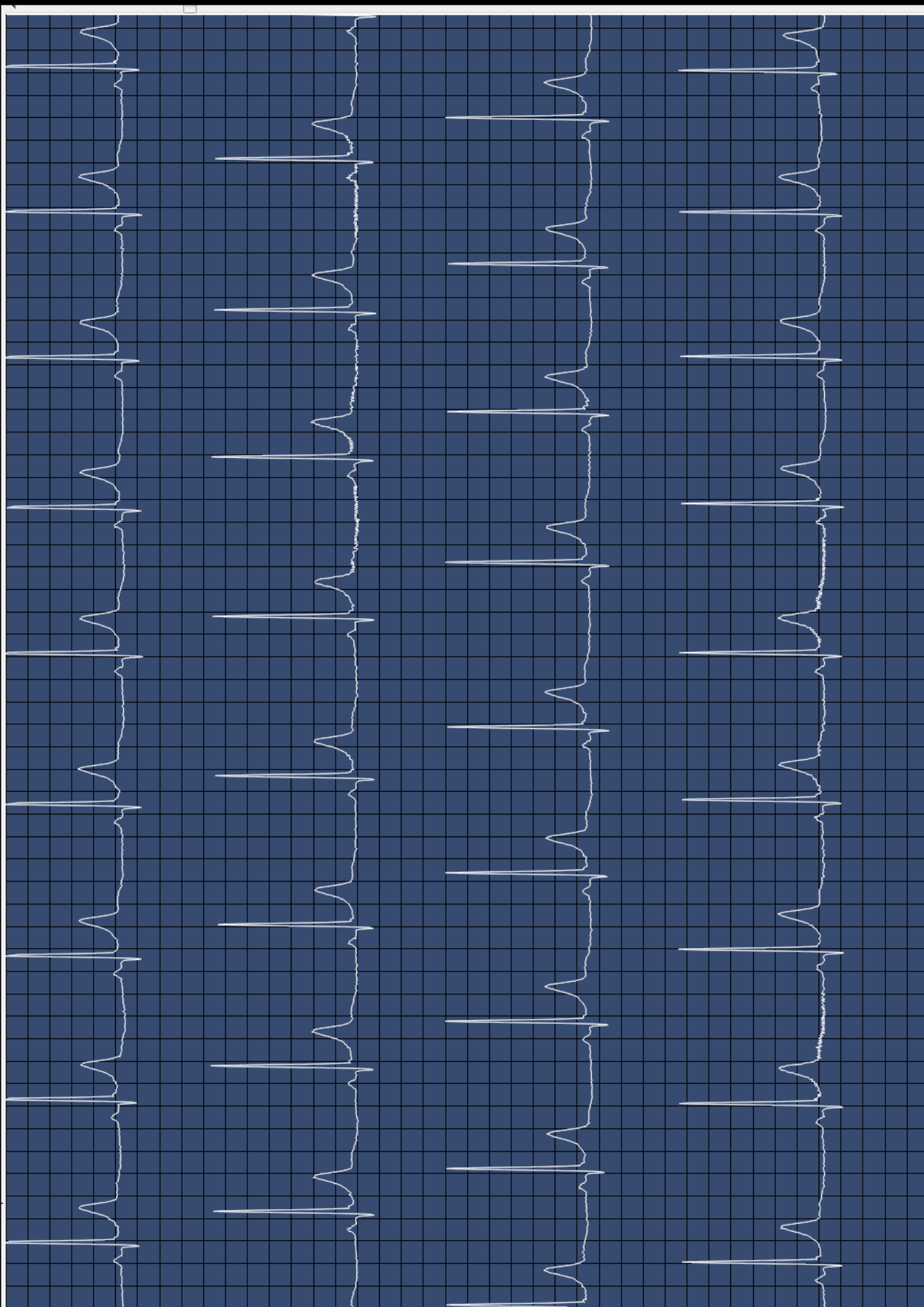

Supplement: Supplementary file 2 [file Data_Sheet_2.zip › EKG blindede/Subject 5 rest + max apnoea/5 rest V6.pdf]
