# Supplementary material for: Extreme Hypoxia Causing Brady-Arrythmias During Apnea in Elite Breath-Hold Divers
Source: Front Physiol. 2021 Dec 3;12:712573. doi: 10.3389/fphys.2021.712573 (PMC8678416; doi:10.3389/fphys.2021.712573)
Supplement: Supplementary file 3 [file Table_1.docx]

Table S1. Characteristics of diving mammals compared to non diving (terrestrial) mammals.

| Characteristic | ***Diving mammal*** | ***Non diving mammal*** |
| --- | --- | --- |
| Oxygen storage capacity | Marine mammals reduce cardiac output, and limit peripheral blood flow during diving, causing apnea, bradycardia, and peripheral vasoconstriction (the dive response), which reduces convective oxygen transport to muscles resulting in tissue hypoxia (Elsner and Gooden 1983) and appears to limit aerobic scope (Davis et al. 1991).High altitude otters compared to lowland otters increase Hb concentration, but also increase NO and have reduction in serum albumin(Crait et al. 2012; Davis and Kanatous 1999). | Terrestrial animals (dogs, racehorses) rely on high mitochondrial volume densities in skeletal muscles and cardiorespiratory adaptations (increased ventilation and cardiac output) that enhance convective and diffusive oxygen transport (Hoppeler et al. 1981). |
| Energy consumption | Weddell seals capacity to fuel metabolism using poly unsaturated fatty acids seems oxygen conserving (Trumble and Kanatous 2012). | Weddell seal pup has increased polyunsaturated fatty acids recovered from lipids associated with their skeletal muscle whereas saturated fatty acids are decreased when compared with adults. In adult seals, the fatty acids recovered were primarily monounsaturated fatty acids(Trumble et al. 2010). |
| Oxygen diffusion capacity | Harbor seals: Myoglobin concentration, citrate synthase activity, and β-hydroxyacyl-CoA dehydrogenase were 1.1- to 2.3-fold greater in the swimming vs. nonswimming muscles.Total mitochondrial volume density per volume of fiber of 9.7% (Kanatous et al. 1999) | The swimming muscles of Steller sea lions (Eumetopias jubatus,n = 27) and Northern fur seals (Callorhinus ursinus,n = 5)have average mitochondrial volume densities of 6.2 and 8.8%, respectively as compared to non-swimming muscles(Kanatous et al. 1999). |
| Michaelis Konstant | Marine mammals do not possess enhanced anaerobic capacity to maintain ATP synthesis under the hypoxic conditions of diving or unusually high anaerobic enzyme activities compared with terrestrial mammals (Castellini et al. 1981) |  |
| Antioxidant capacity:Animals that routinely face high changes in oxygen availability and/or consumption seem to show a general strategy to prevent oxidative damage by having either appropriate high constitutive antioxidant defences and/or the ability to undergo arrested states, where depressed metabolic rates minimize the oxidative challenge (Wilhelm et al. 2002). | Samples of ringed seal heart, muscle and kidney subjected to oxidative stress by addition of xanthine oxidase. Production of superoxide radical (O(2)(.-)), lipid peroxidation (as determined by the presence of thiobarbituric acid reactive substances, TBARS) and antioxidant capacity (AOX) were in pig tissues less O(2)(.-) and TBARS compared with ringed seal tissues. These results show that ringed seal muscle, heart and kidney can be induced in vitro to generate reactive oxygene species, and suggest that the living seal's protective defenses may depend upon O(2)(.-) production, similar to the protective effect of experimental preconditioning, or on enhanced intermediate scavenging, as evidenced by the larger AOX found in ringed seal tissues (Zenteno-Savin et al. 2002). | Prolonged fasting, sleep apnea, hypoxemia and ischemia/reperfusion increase oxidant production and oxidative stress in terrestrial mammals. In seals, however, neither prolonged fasting nor apnea-induced hypoxemia or ischemia/reperfusion increase systemic or local oxidative damage(Vazquez-Medina et al. 2012). |

References

Castellini, M. A., Kooyman, G. L., and Somero, G. N.

1981 Glycolytic enzyme activities in tissues of marine and terrestrial mammals. Pp. 242-252: Physiological Zoology.

Crait, J. R., Prange, H. D., Marshall, N. A., Harlow, H. J., Cotton, C. J., and Ben-David, M.

2012 High-altitude diving in river otters: coping with combined hypoxic stresses. *J.Exp.Biol.* 215:256-263.

Davis, R. W., Castellini, M. A., Williams, T. M., and Kooyman, G. L.

1991 Fuel homeostasis in the harbor seal during submerged swimming. *J.Comp Physiol B* 160:627-635.

Davis, R. W. and Kanatous, S. B.

1999 Convective oxygen transport and tissue oxygen consumption in Weddell seals during aerobic dives. *J.Exp.Biol.* 202:1091-1113.

Elsner, R. and Gooden, B.

1983 Diving and asphyxia. A comparative study of animals and man. *Monogr Physiol Soc.* 40:1-168.

Hoppeler, H., Mathieu, O., Krauer, R., Claassen, H., Armstrong, R. B., and Weibel, E. R.

1981 Design of the mammalian respiratory system. VI Distribution of mitochondria and capillaries in various muscles. *Respir.Physiol* 44:87-111.

Kanatous, S. B., DiMichele, L. V., Cowan, D. F., and Davis, R. W.

1999 High aerobic capacities in the skeletal muscles of pinnipeds: adaptations to diving hypoxia. *J.Appl.Physiol (1985.)* 86:1247-1256.

Trumble, S. J. and Kanatous, S. B.

2012 Fatty Acid use in Diving Mammals: More than Merely Fuel. *Front Physiol* 3:184.

Trumble, S. J., Noren, S. R., Cornick, L. A., Hawke, T. J., and Kanatous, S. B.

2010 Age-related differences in skeletal muscle lipid profiles of Weddell seals: clues to developmental changes. *J.Exp.Biol.* 213:1676-1684.

Vazquez-Medina, J. P., Zenteno-Savin, T., Elsner, R., and Ortiz, R. M.

2012 Coping with physiological oxidative stress: a review of antioxidant strategies in seals. *J.Comp Physiol B* 182:741-750.

Wilhelm, F. D., Sell, F., Ribeiro, L., Ghislandi, M., Carrasquedo, F., Fraga, C. G., Wallauer, J. P., Simoes-Lopes, P. C., and Uhart, M. M.

2002 Comparison between the antioxidant status of terrestrial and diving mammals. *Comp Biochem.Physiol A Mol.Integr.Physiol* 133:885-892.

Zenteno-Savin, T., Clayton-Hernandez, E., and Elsner, R.

2002 Diving seals: are they a model for coping with oxidative stress? *Comp Biochem.Physiol C.Toxicol.Pharmacol.* 133:527-536.
