# Supplementary material for: Extreme Hypoxia Causing Brady-Arrythmias During Apnea in Elite Breath-Hold Divers
Source: Front Physiol. 2021 Dec 3;12:712573. doi: 10.3389/fphys.2021.712573 (PMC8678416; doi:10.3389/fphys.2021.712573)
Supplement: Supplementary file 2 [file 3 max apnoea V5.pdf › EKG blindede/Subject 3 rest + max apnoea/3 max apnoea V5.pdf]

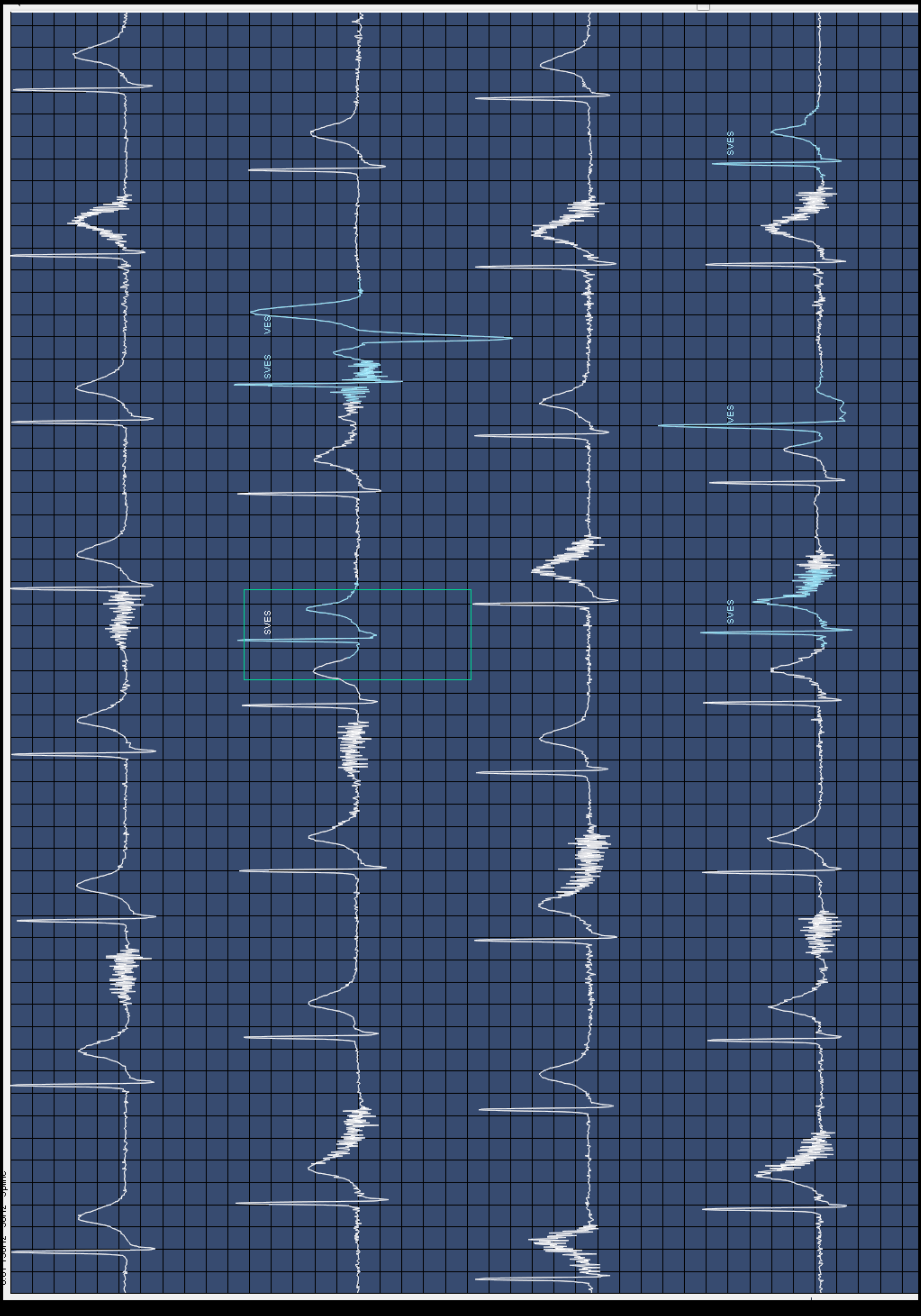

SVES
VES
SVES
SVES
VES
SVES
