# Supplementary material for: Extreme Hypoxia Causing Brady-Arrythmias During Apnea in Elite Breath-Hold Divers
Source: Front Physiol. 2021 Dec 3;12:712573. doi: 10.3389/fphys.2021.712573 (PMC8678416; doi:10.3389/fphys.2021.712573)
Supplement: Supplementary file 2 [file 4 max apnoea V6 no 2.pdf › EKG blindede/Subject 4 rest + max apnoea/4 max apnoea V6 no 2.pdf]

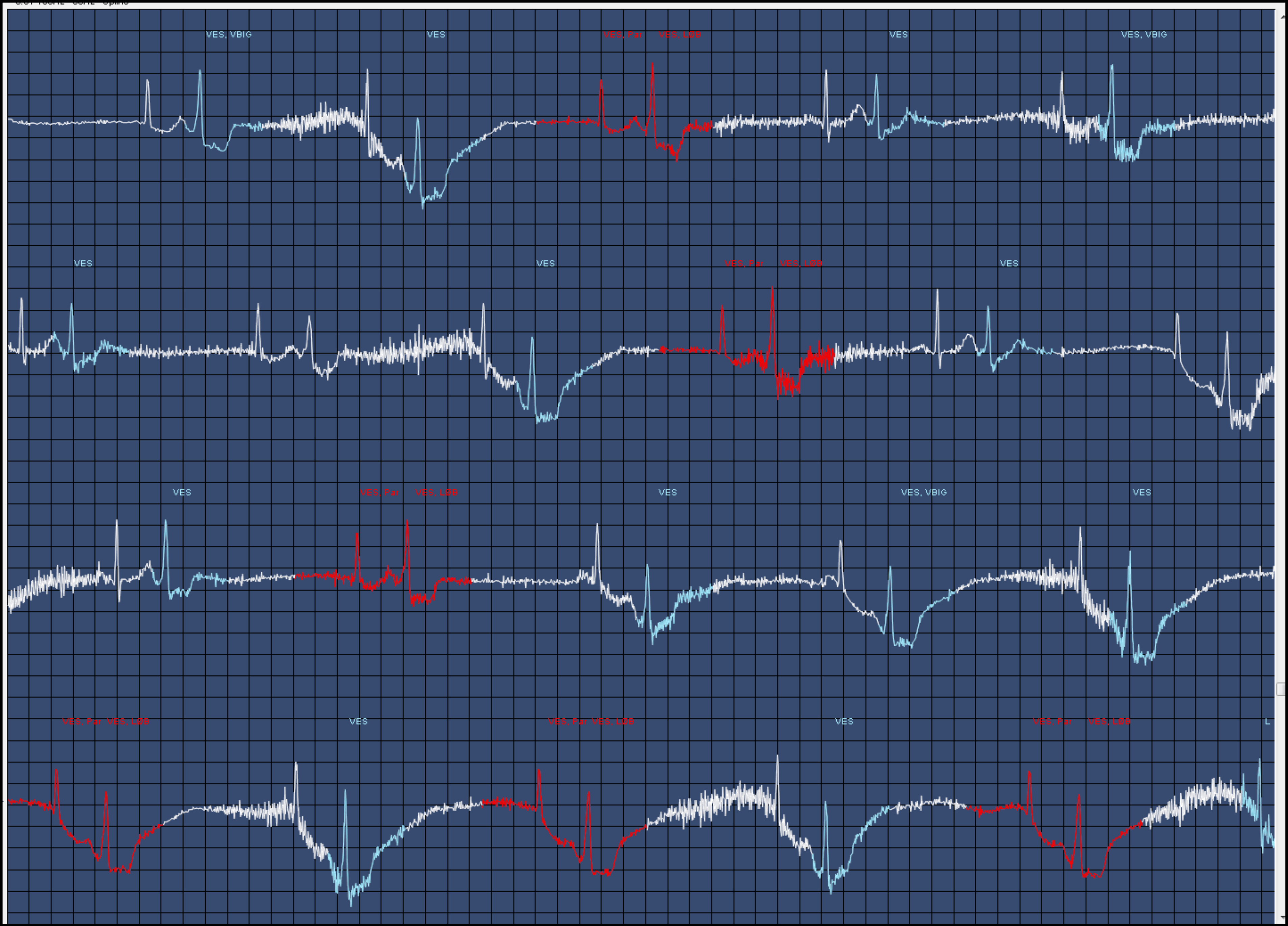

VES, VBIG
VES
VES, Par
VES, LBB
VES
VES, VBIG
VES
VES
VES, Par
VES, LBB
VES
VES
VES, Par
VES, LBB
VES
VES, VBIG
VES
VES, Par VES, LBB
VES
VES, Par VES, LBB
VES
VES, Par
VES, LBB
